# Supplementary figures and images for: Comparative analysis of the effects of cyclophosphamide and dexamethasone on intestinal immunity and microbiota in delayed hypersensitivity mice
Source: PLoS One. 2024 Oct 17;19(10):e0312147. doi: 10.1371/journal.pone.0312147 (PMC11486373; doi:10.1371/journal.pone.0312147)

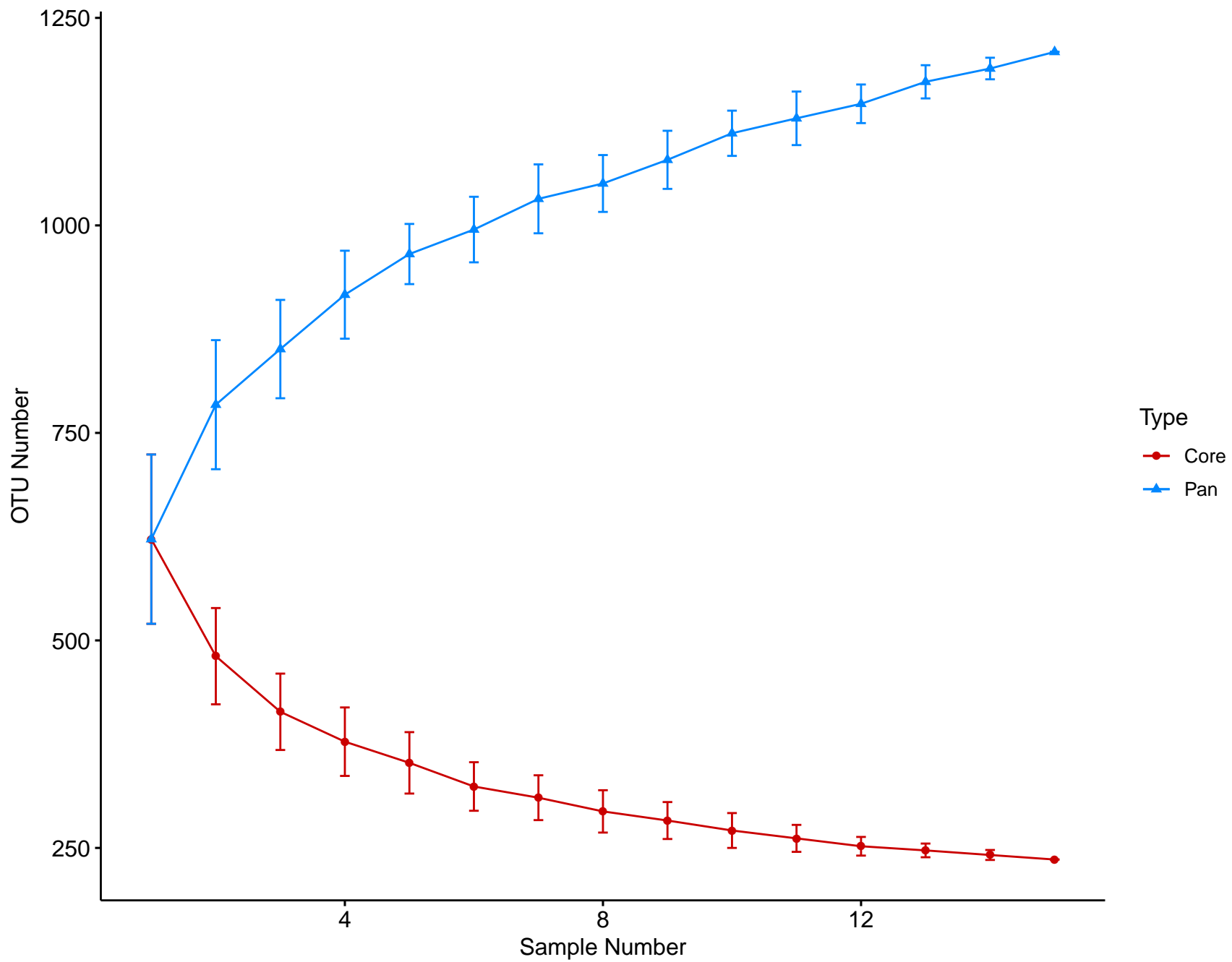

Supplement: S2 File — (ZIP) [file pone.0312147.s002.zip › 3_AlphaDiversity/Accumulation/accumulation.pdf]

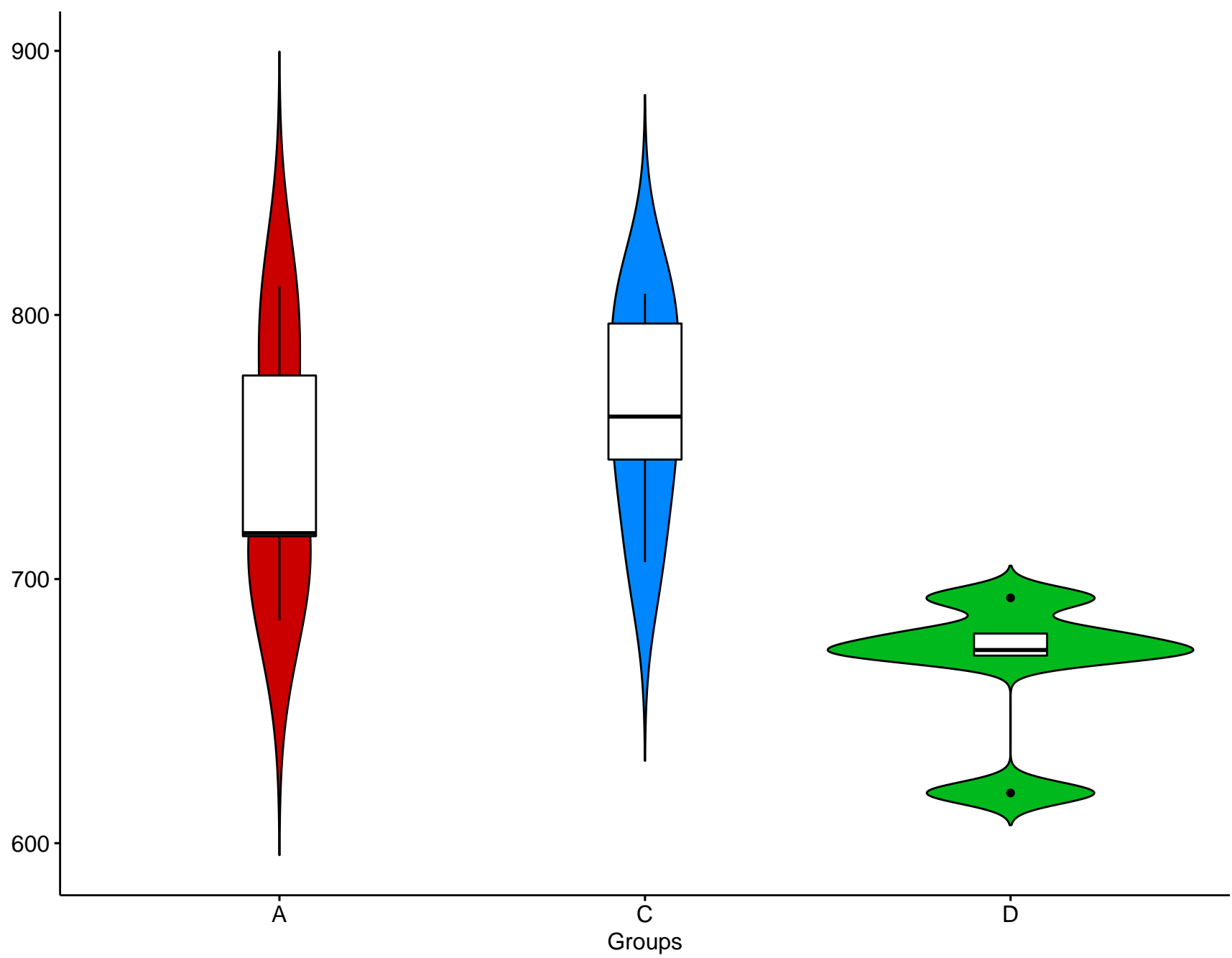

Supplement: S2 File — (ZIP) [file pone.0312147.s002.zip › 3_AlphaDiversity/AlphaIndex/ace_alpha_diversity_boxplot.pdf]

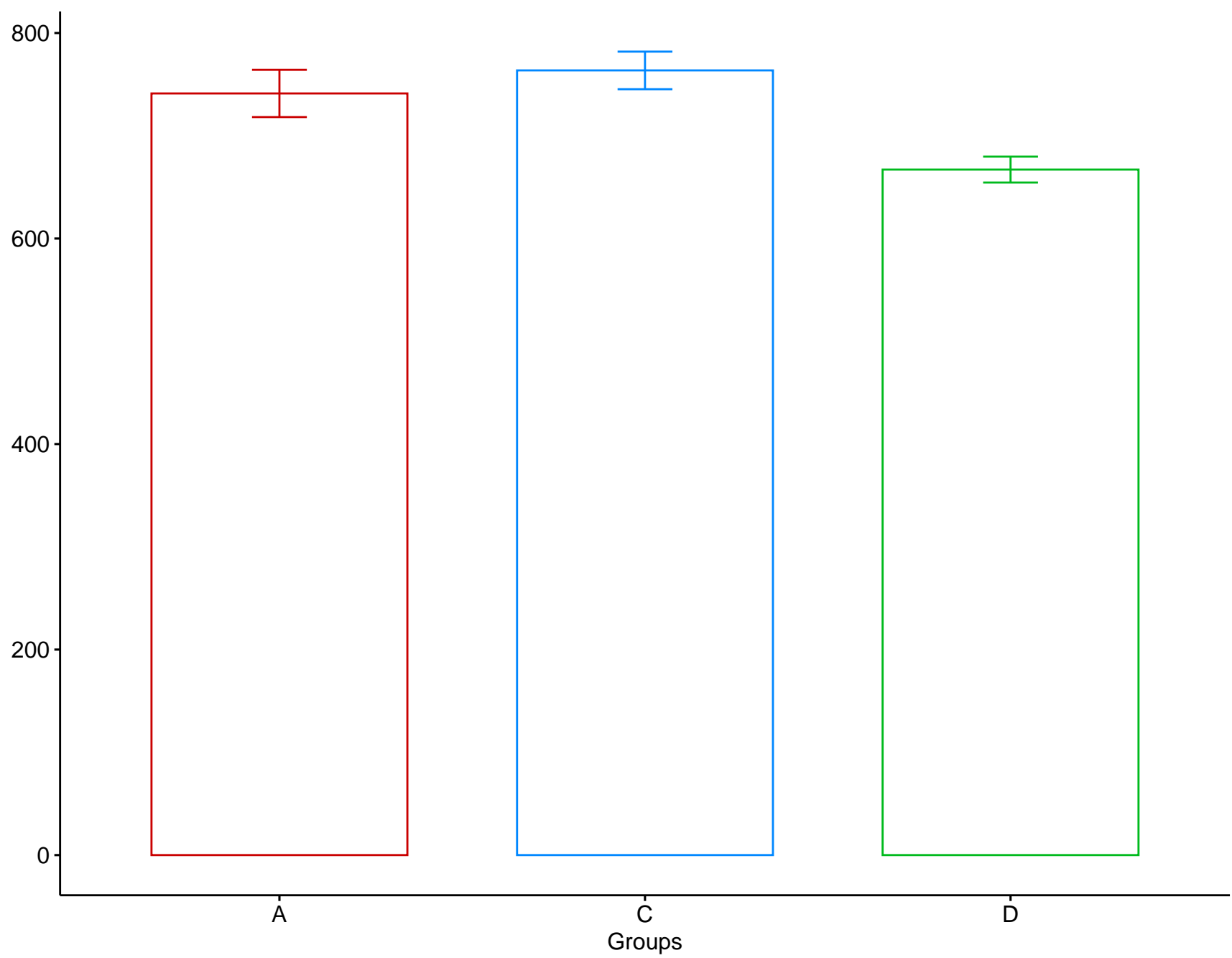

Supplement: S2 File — (ZIP) [file pone.0312147.s002.zip › 3_AlphaDiversity/AlphaIndex/ace_alpha_diversity_meanse.pdf]

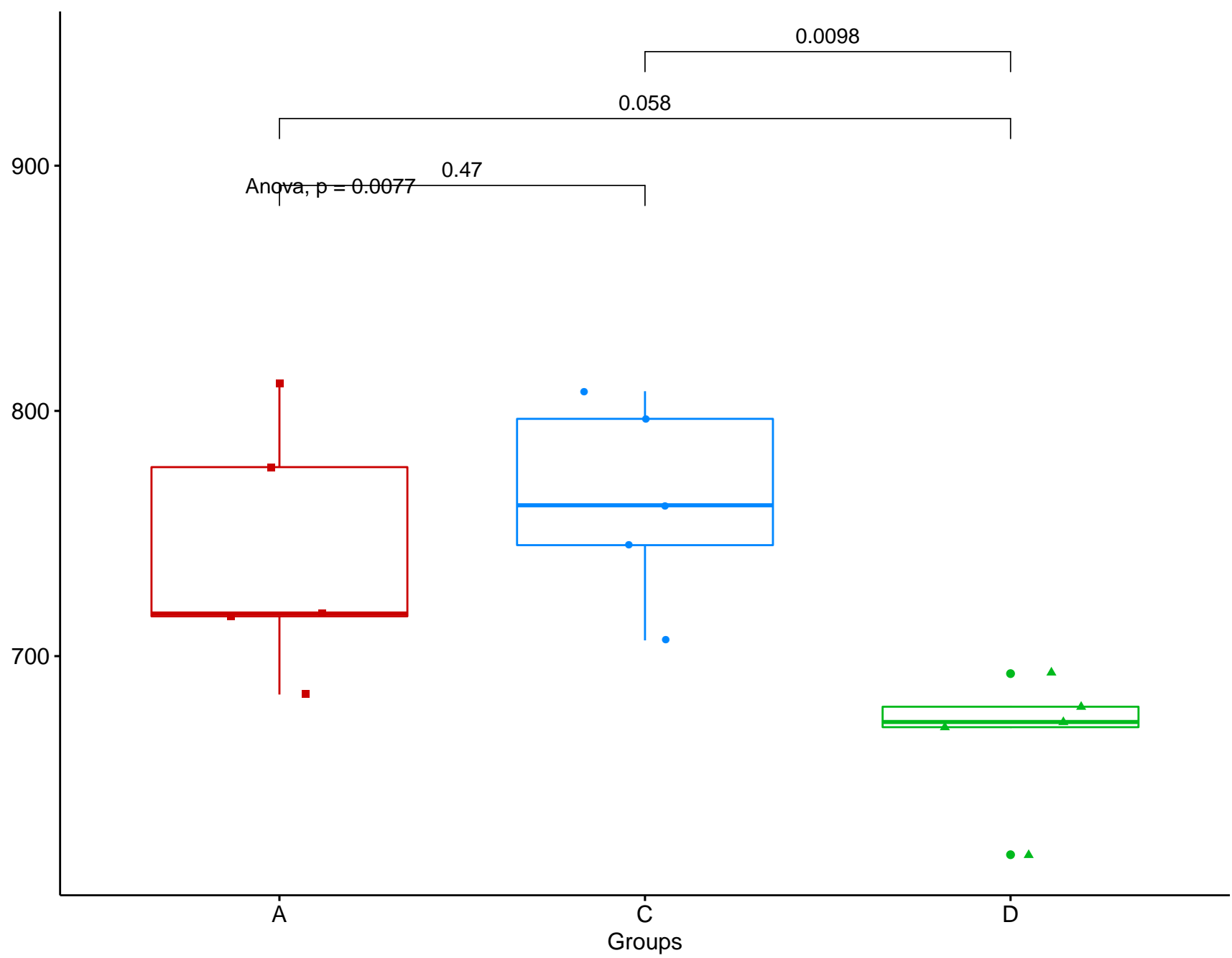

Supplement: S2 File — (ZIP) [file pone.0312147.s002.zip › 3_AlphaDiversity/AlphaIndex/ace_alpha_diversity_test.pdf]

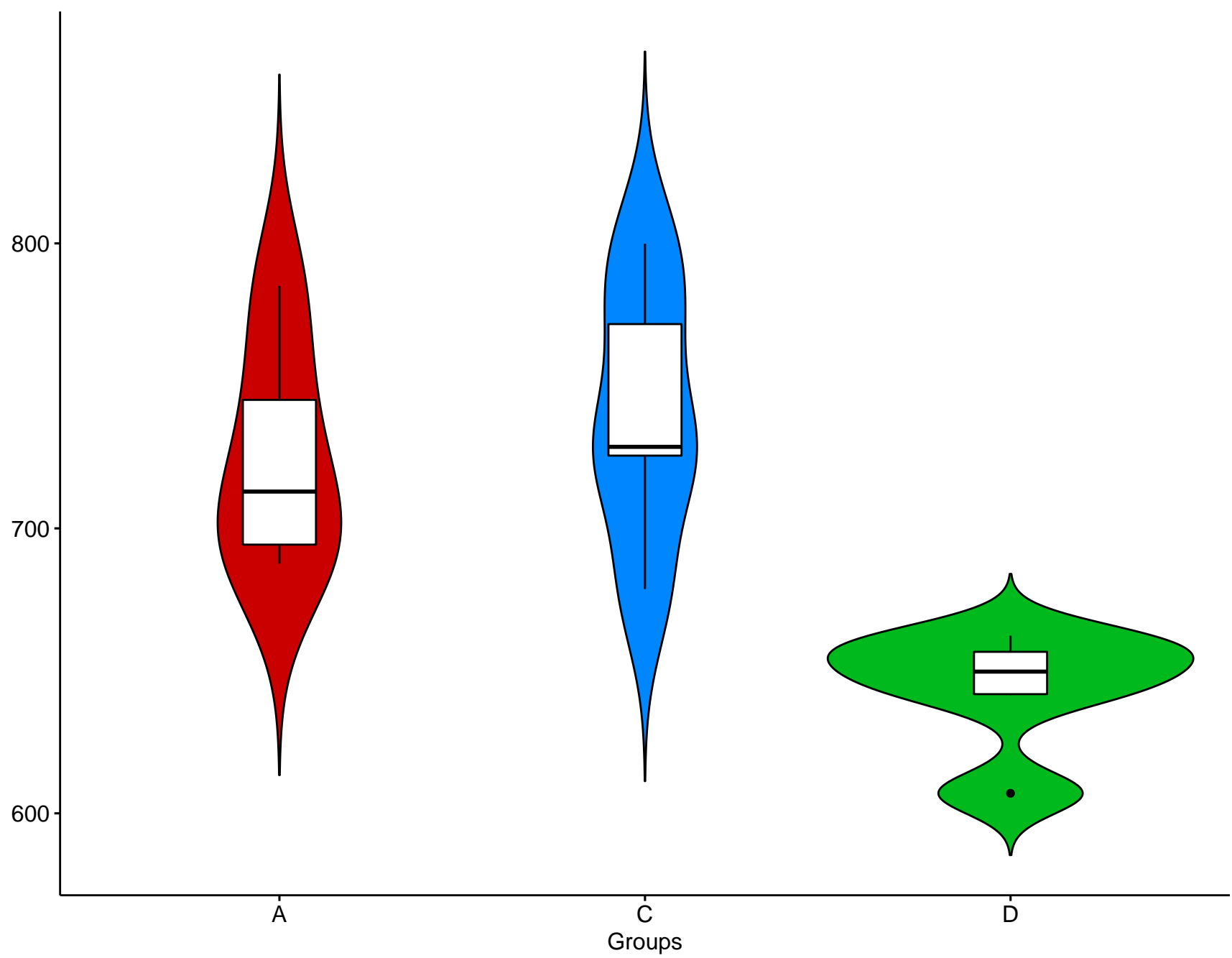

Supplement: S2 File — (ZIP) [file pone.0312147.s002.zip › 3_AlphaDiversity/AlphaIndex/chao_alpha_diversity_boxplot.pdf]

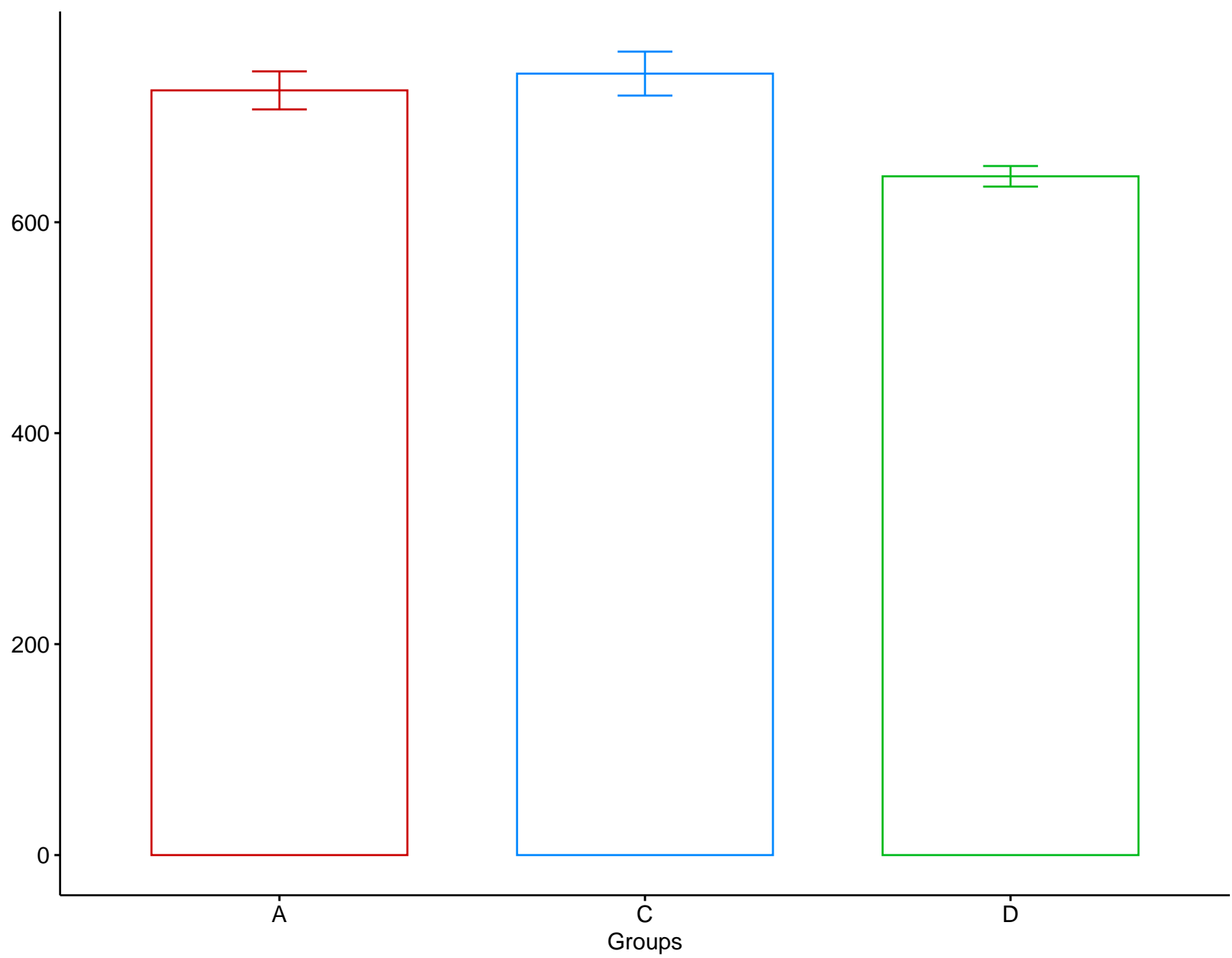

Supplement: S2 File — (ZIP) [file pone.0312147.s002.zip › 3_AlphaDiversity/AlphaIndex/chao_alpha_diversity_meanse.pdf]

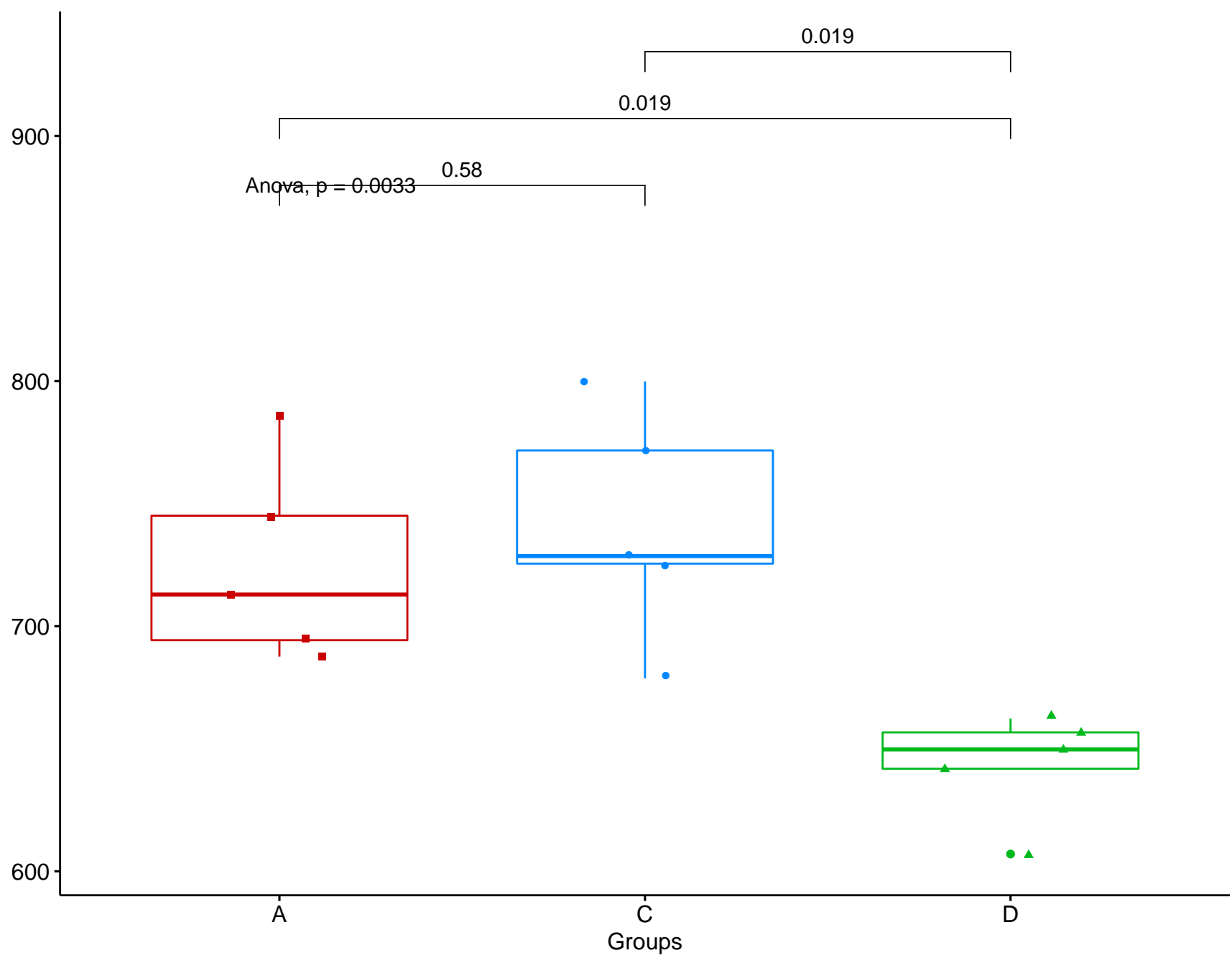

Supplement: S2 File — (ZIP) [file pone.0312147.s002.zip › 3_AlphaDiversity/AlphaIndex/chao_alpha_diversity_test.pdf]

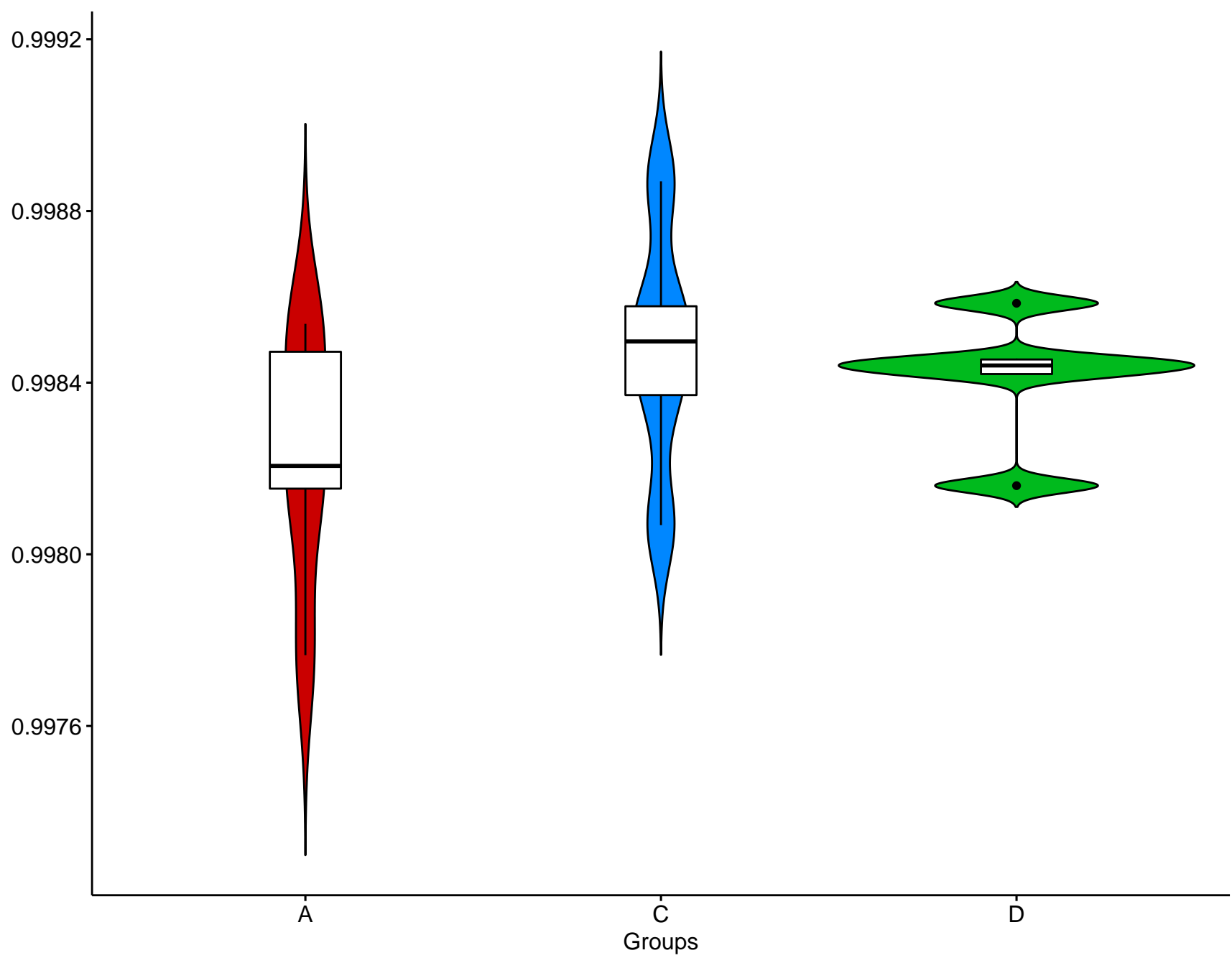

Supplement: S2 File — (ZIP) [file pone.0312147.s002.zip › 3_AlphaDiversity/AlphaIndex/coverage_alpha_diversity_boxplot.pdf]

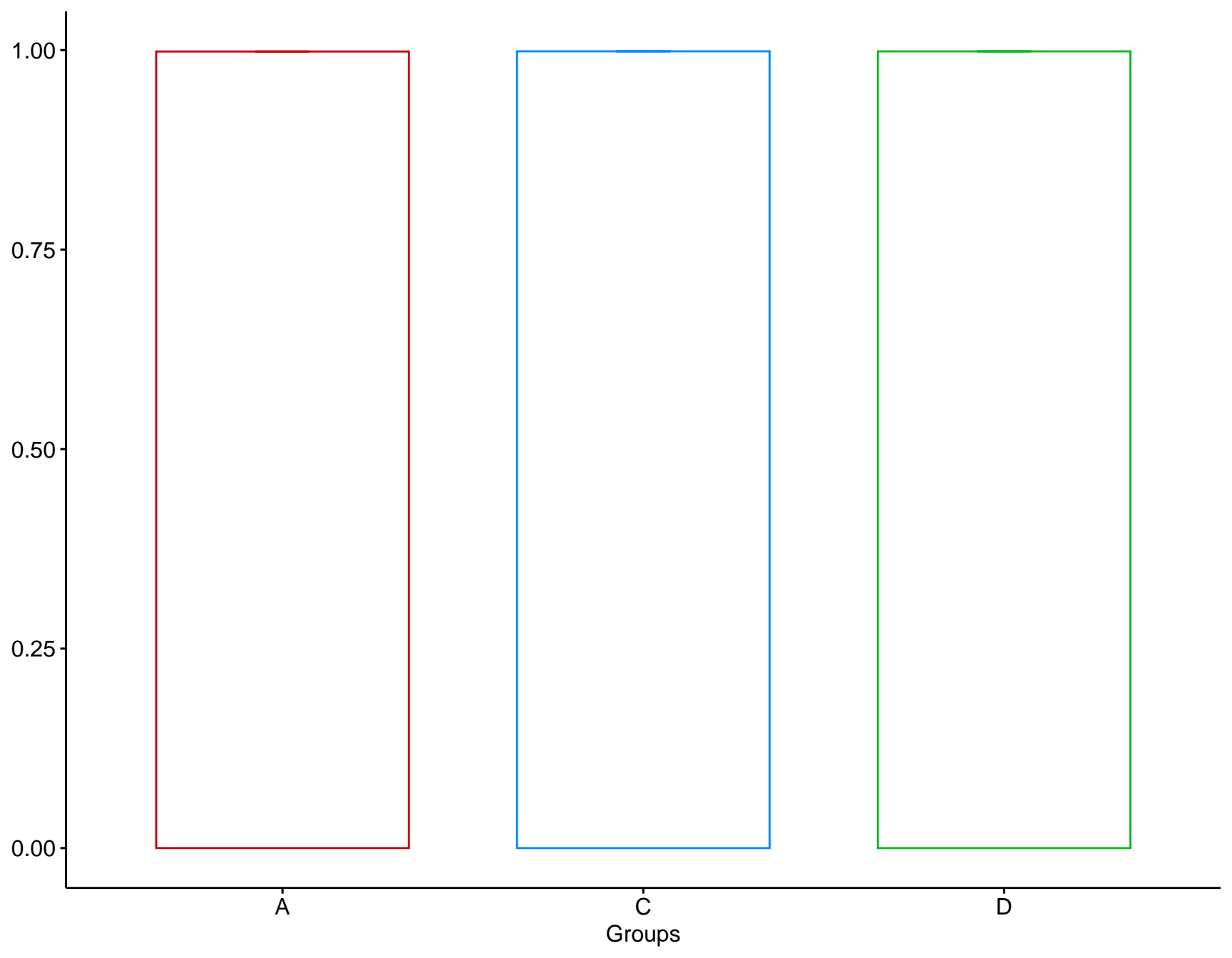

Supplement: S2 File — (ZIP) [file pone.0312147.s002.zip › 3_AlphaDiversity/AlphaIndex/coverage_alpha_diversity_meanse.pdf]

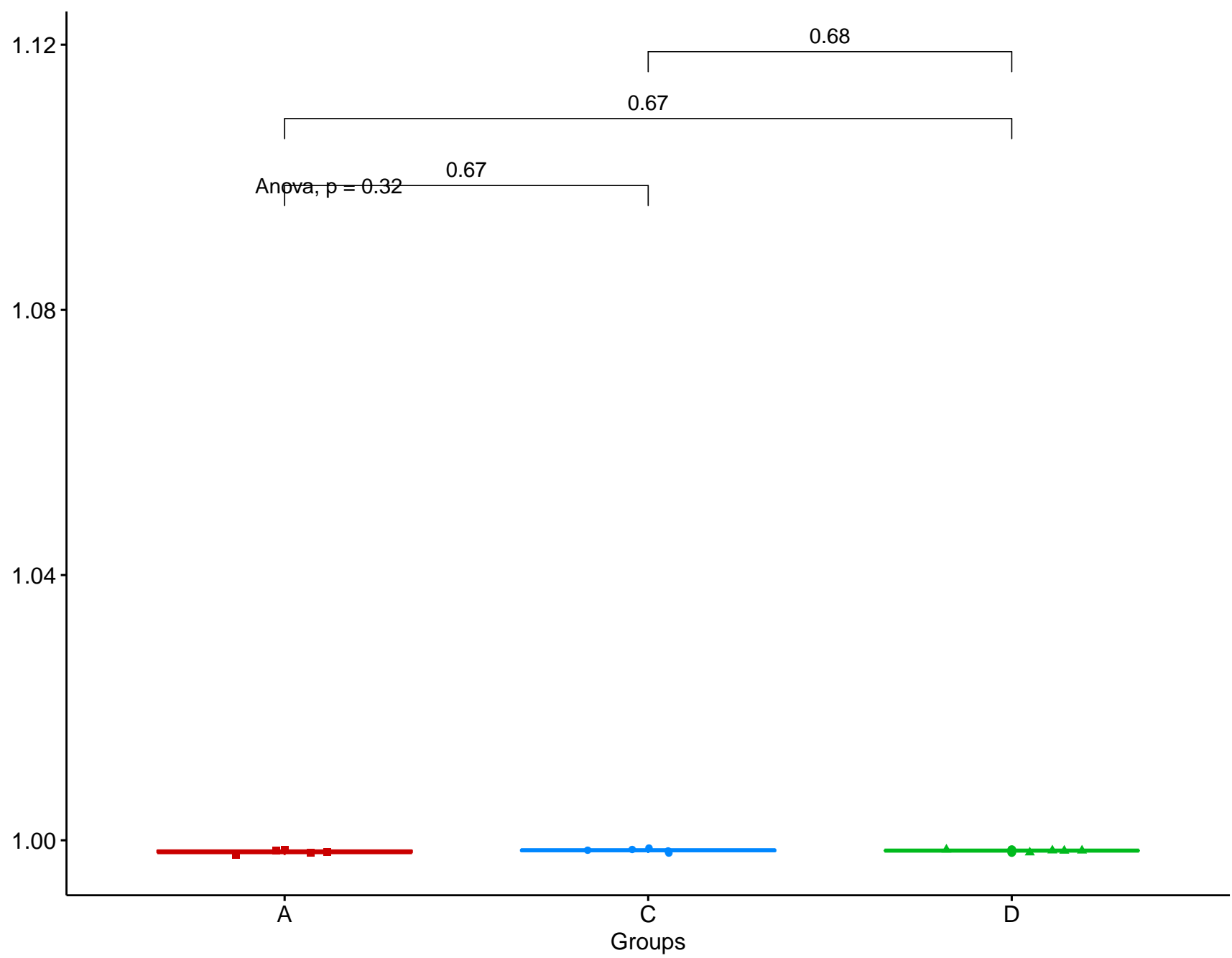

Supplement: S2 File — (ZIP) [file pone.0312147.s002.zip › 3_AlphaDiversity/AlphaIndex/coverage_alpha_diversity_test.pdf]

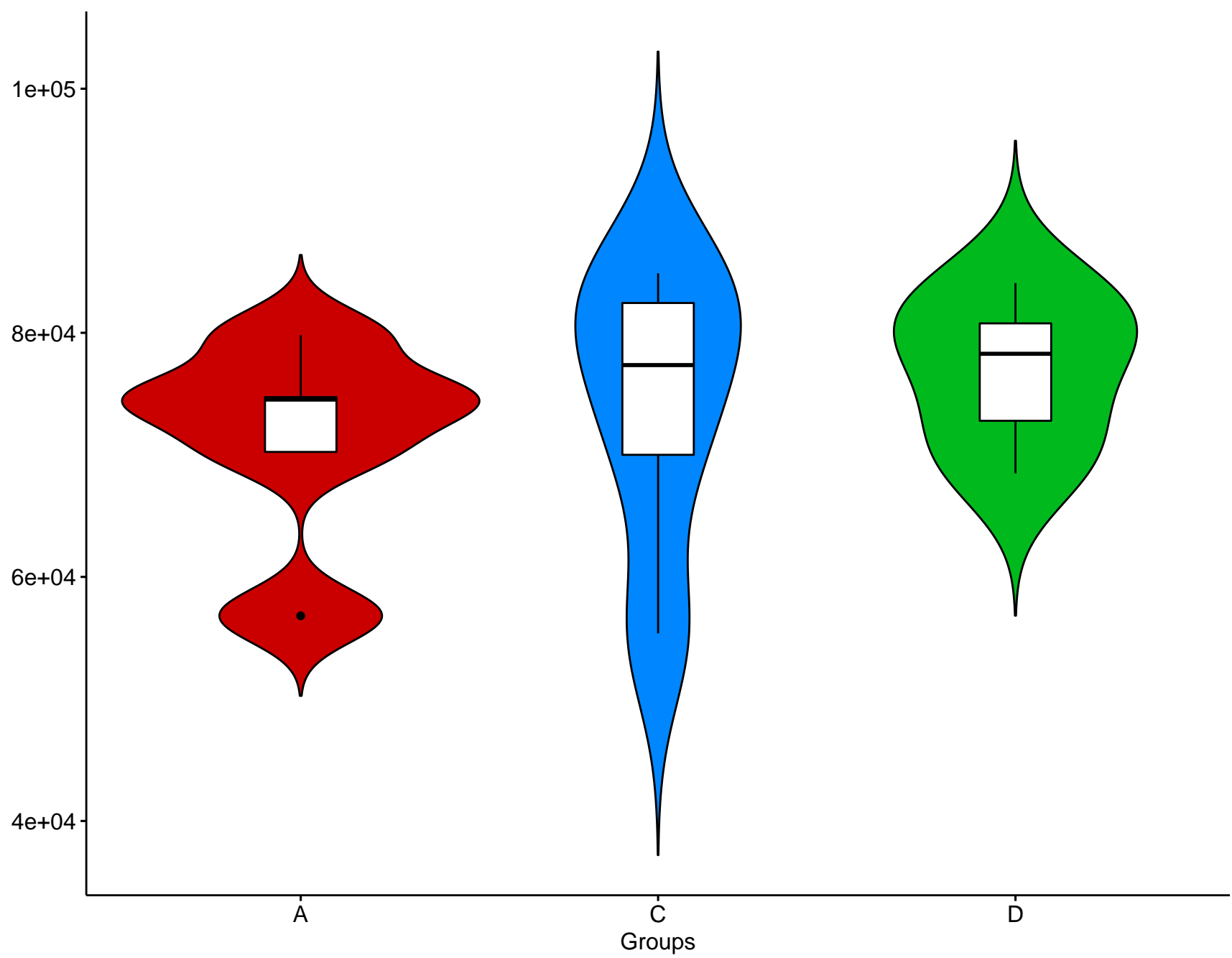

Supplement: S2 File — (ZIP) [file pone.0312147.s002.zip › 3_AlphaDiversity/AlphaIndex/number_alpha_diversity_boxplot.pdf]

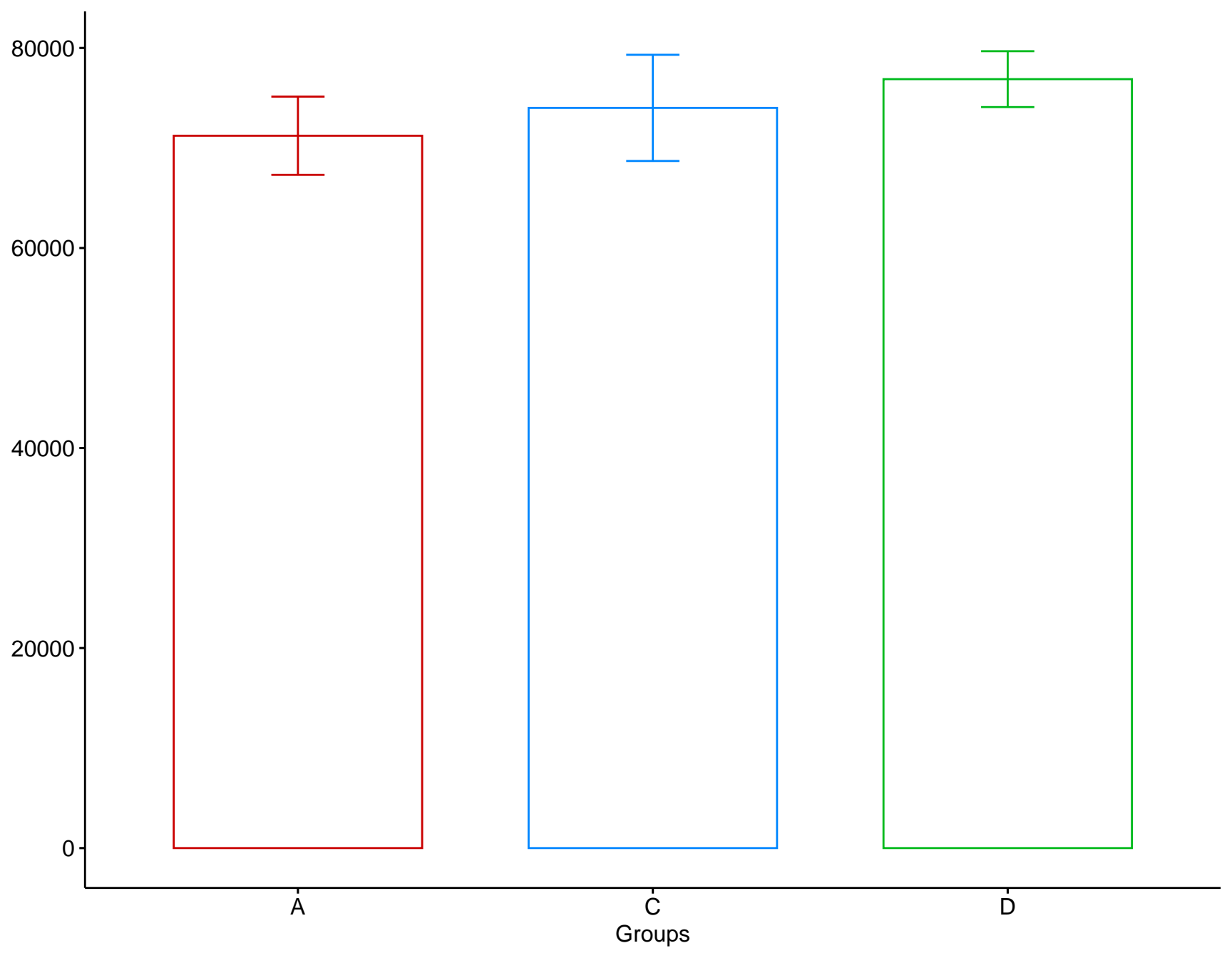

Supplement: S2 File — (ZIP) [file pone.0312147.s002.zip › 3_AlphaDiversity/AlphaIndex/number_alpha_diversity_meanse.pdf]

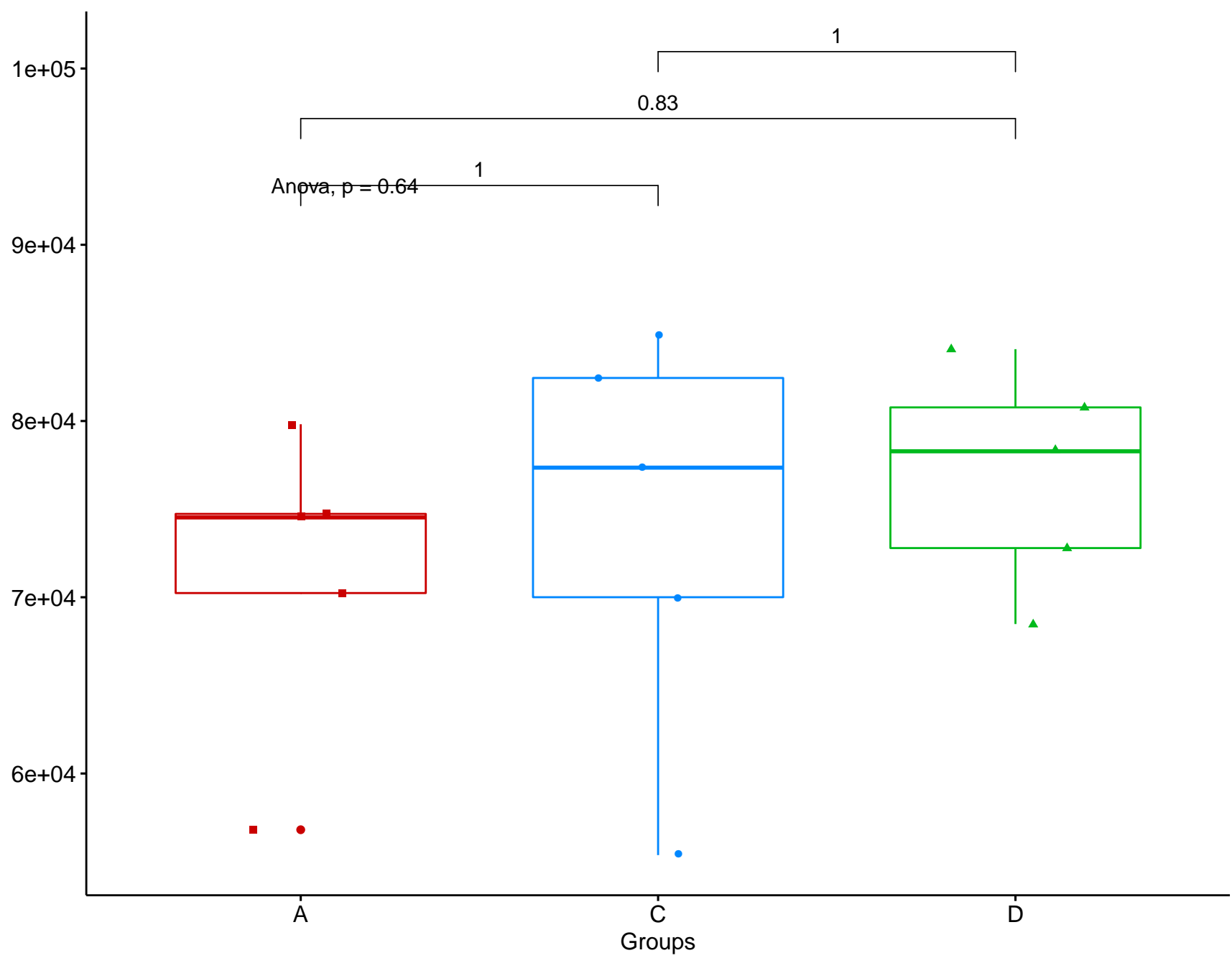

Supplement: S2 File — (ZIP) [file pone.0312147.s002.zip › 3_AlphaDiversity/AlphaIndex/number_alpha_diversity_test.pdf]

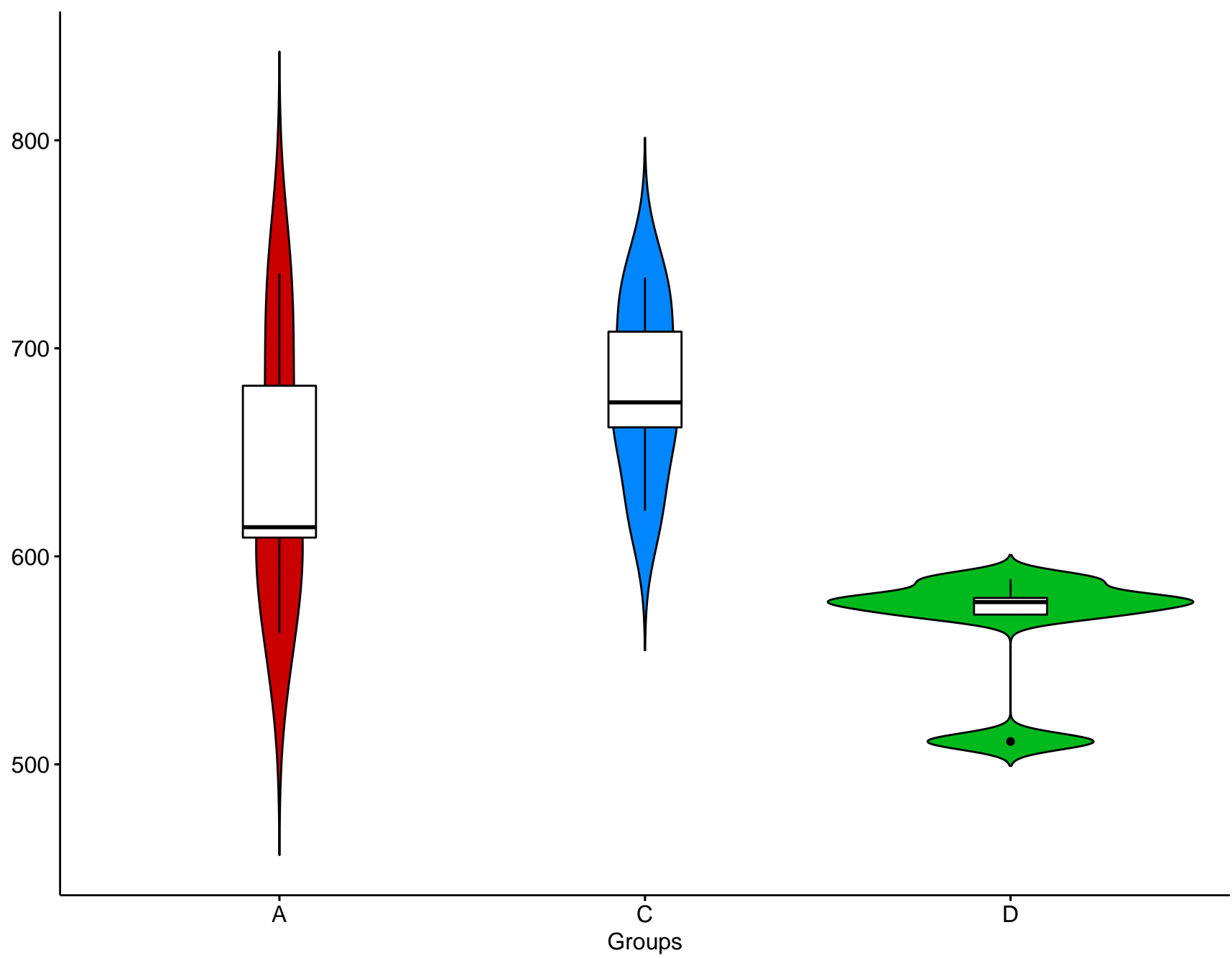

Supplement: S2 File — (ZIP) [file pone.0312147.s002.zip › 3_AlphaDiversity/AlphaIndex/otus_alpha_diversity_boxplot.pdf]

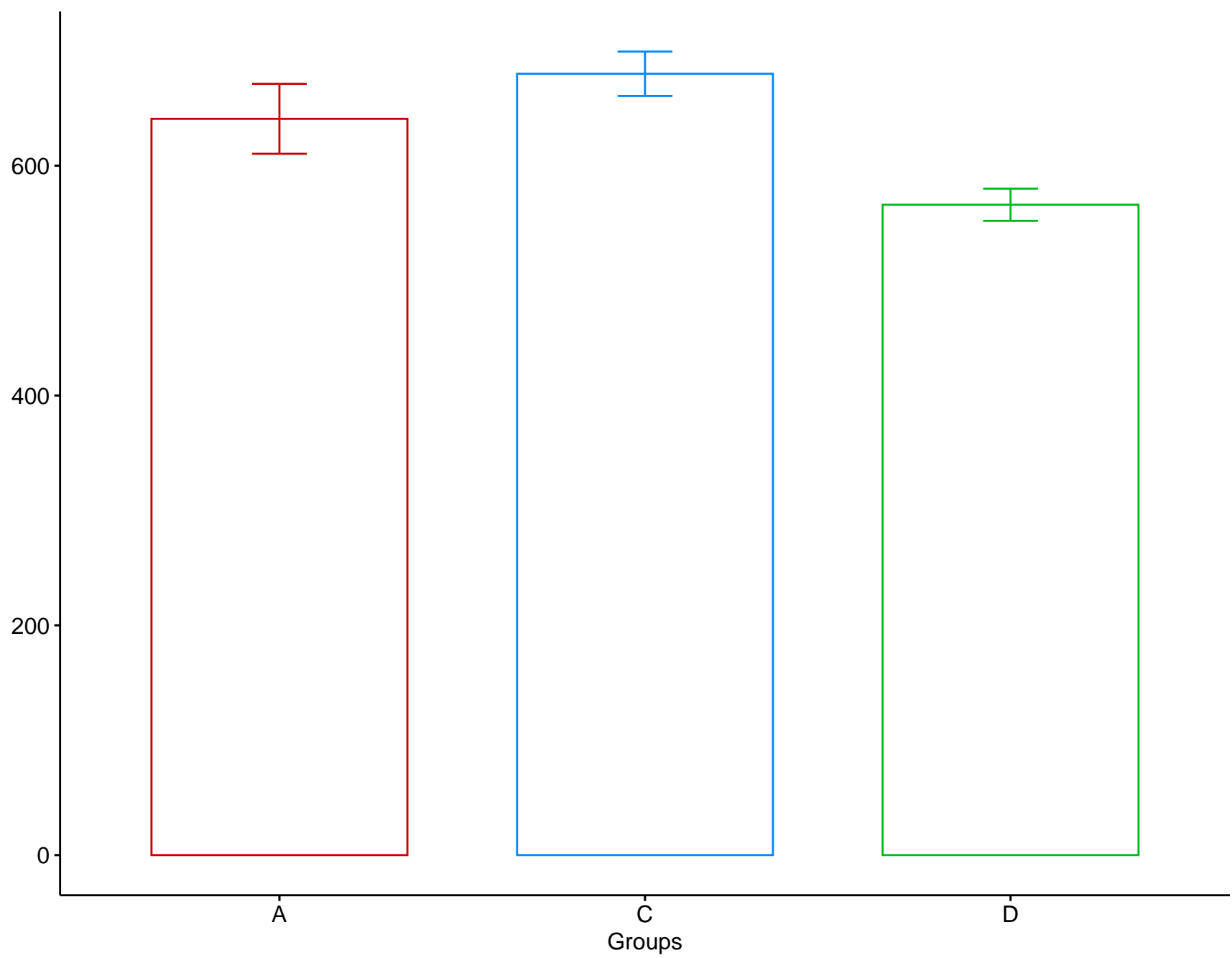

Supplement: S2 File — (ZIP) [file pone.0312147.s002.zip › 3_AlphaDiversity/AlphaIndex/otus_alpha_diversity_meanse.pdf]

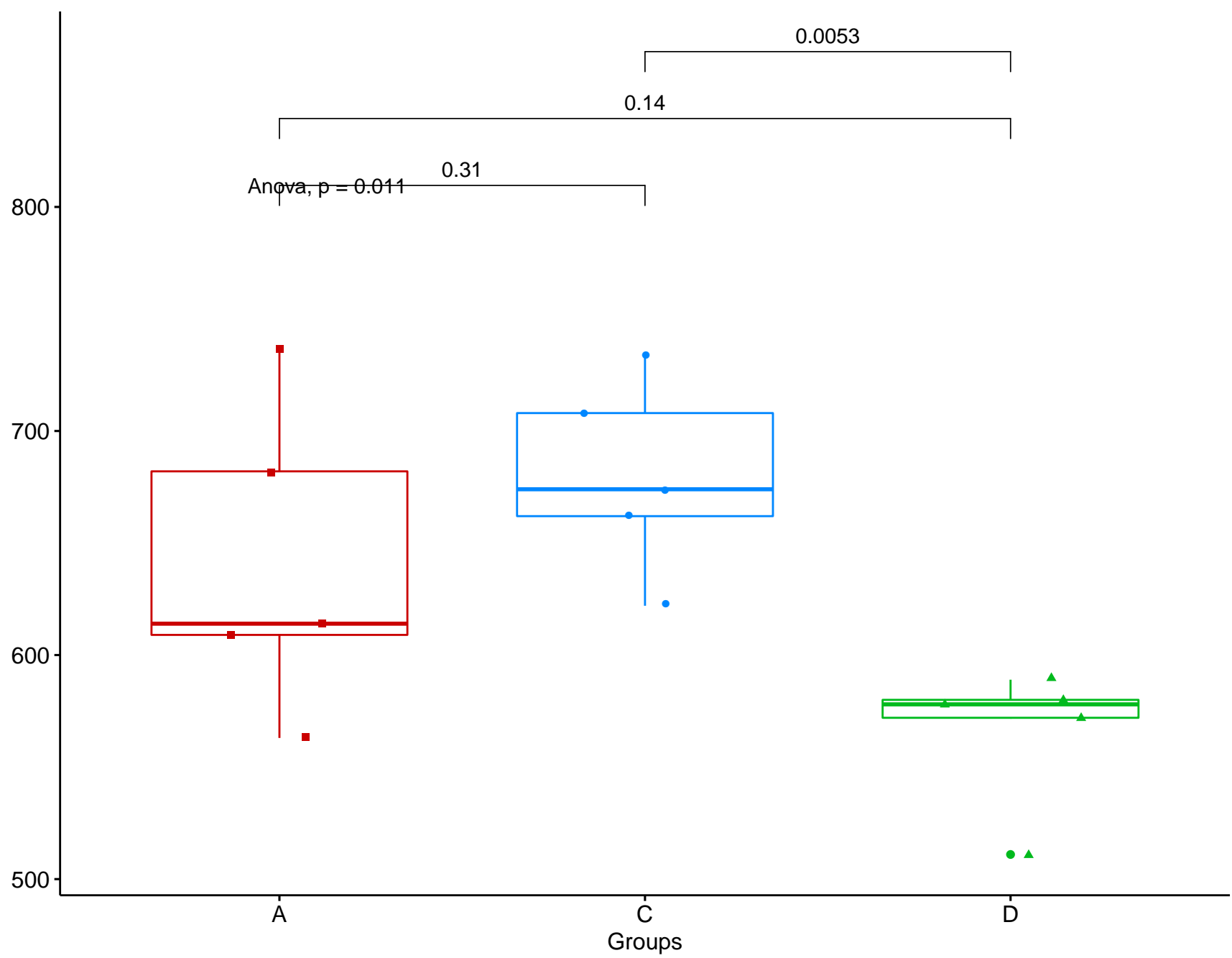

Supplement: S2 File — (ZIP) [file pone.0312147.s002.zip › 3_AlphaDiversity/AlphaIndex/otus_alpha_diversity_test.pdf]

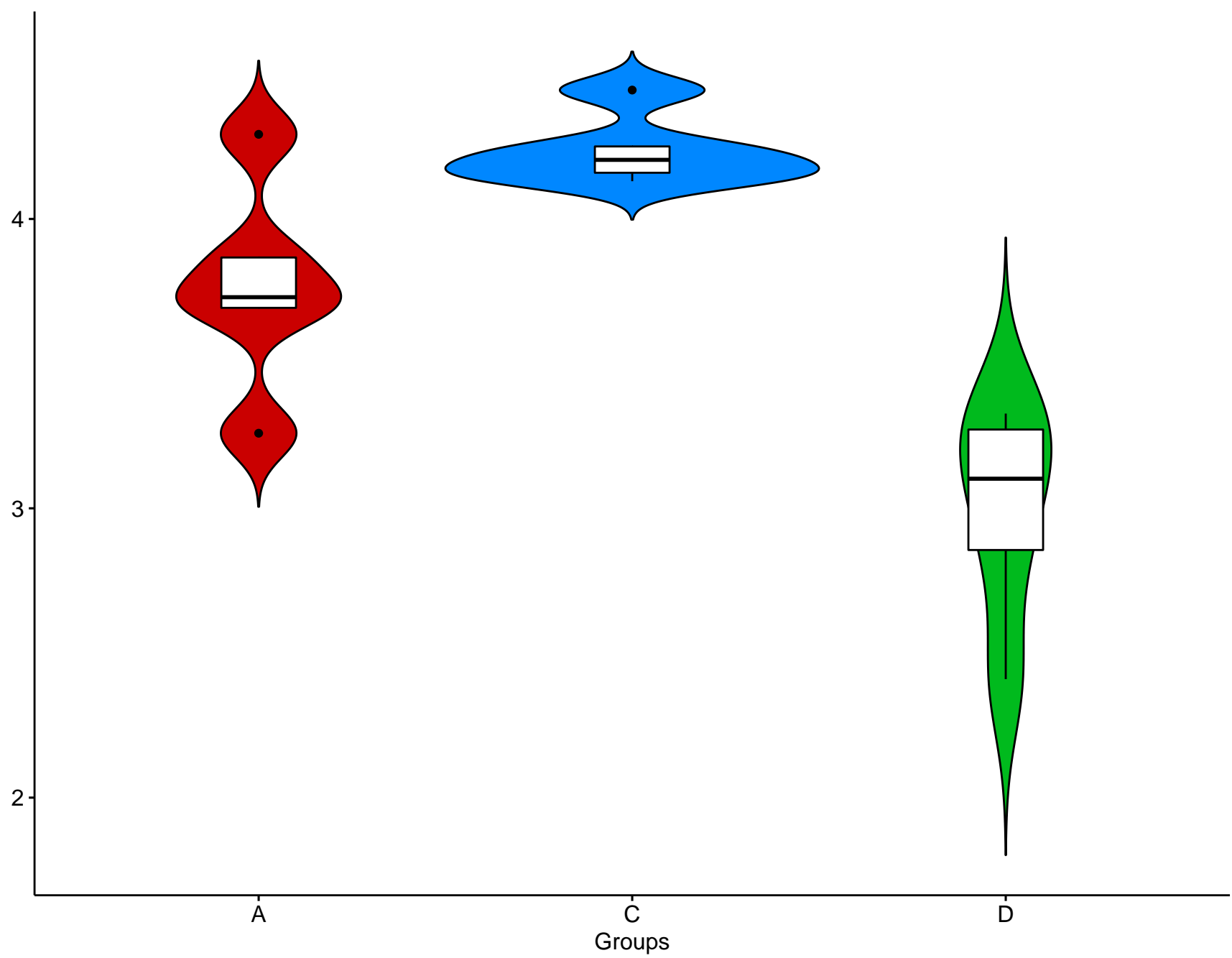

Supplement: S2 File — (ZIP) [file pone.0312147.s002.zip › 3_AlphaDiversity/AlphaIndex/shannon_alpha_diversity_boxplot.pdf]

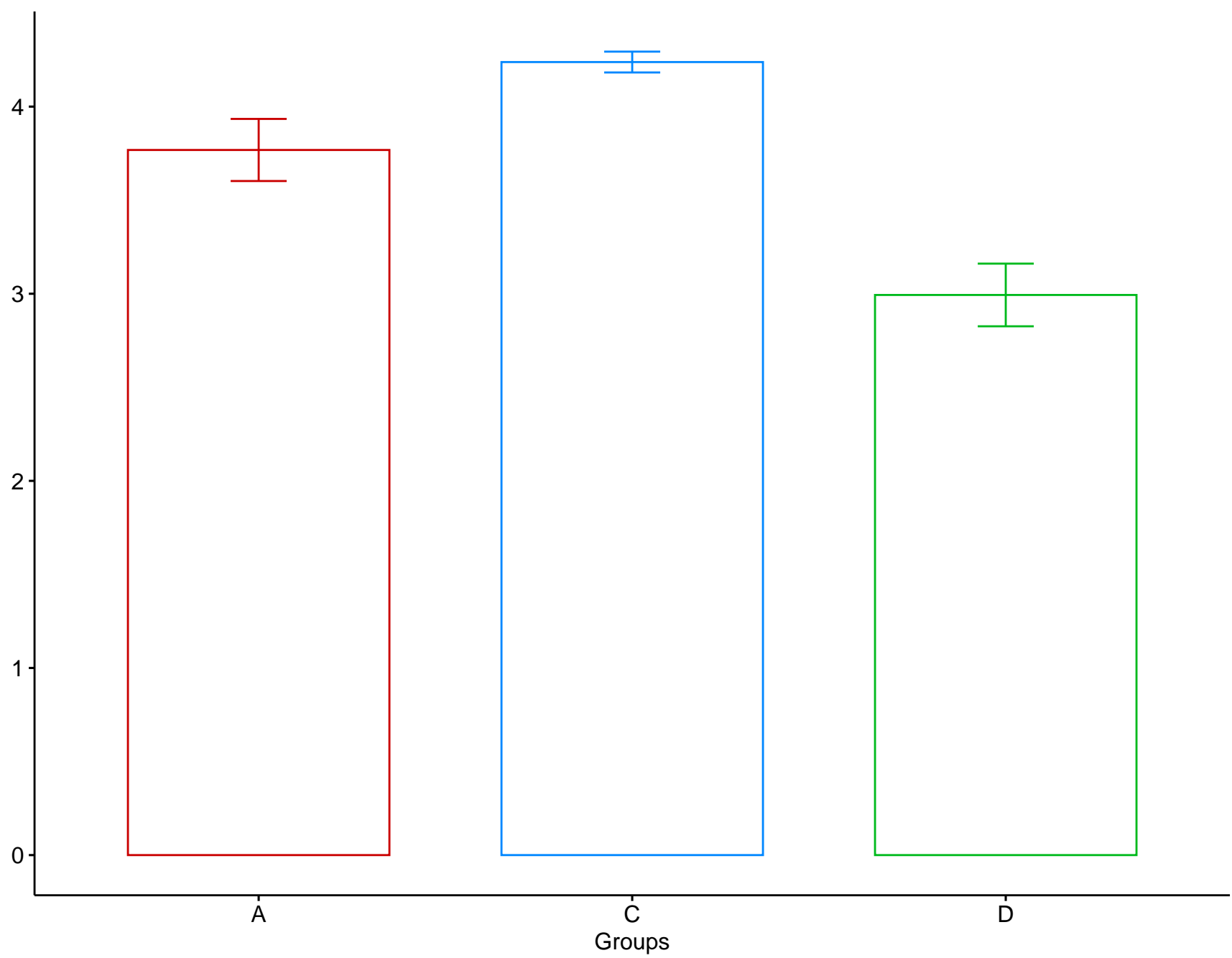

Supplement: S2 File — (ZIP) [file pone.0312147.s002.zip › 3_AlphaDiversity/AlphaIndex/shannon_alpha_diversity_meanse.pdf]

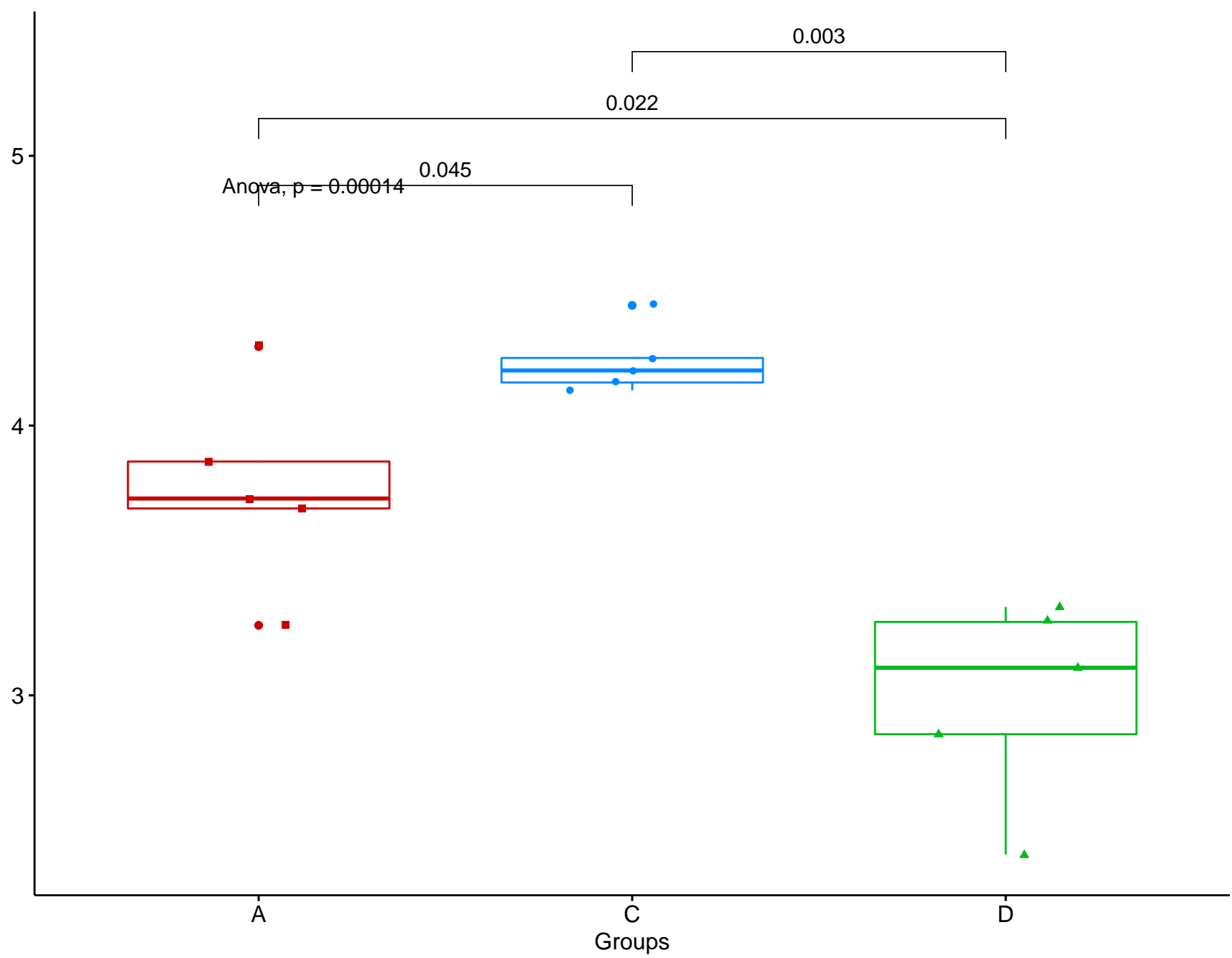

Supplement: S2 File — (ZIP) [file pone.0312147.s002.zip › 3_AlphaDiversity/AlphaIndex/shannon_alpha_diversity_test.pdf]

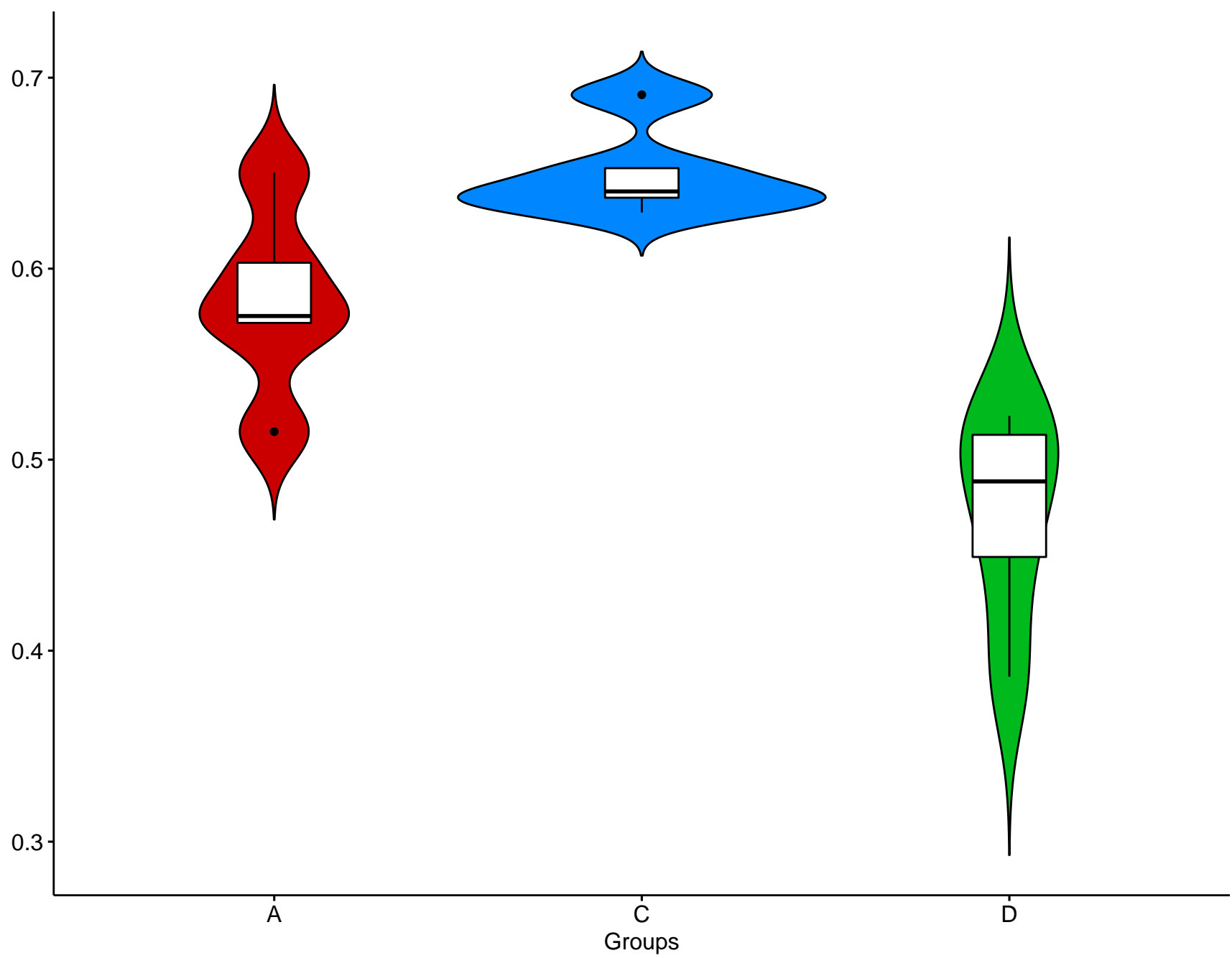

Supplement: S2 File — (ZIP) [file pone.0312147.s002.zip › 3_AlphaDiversity/AlphaIndex/shannoneven_alpha_diversity_boxplot.pdf]

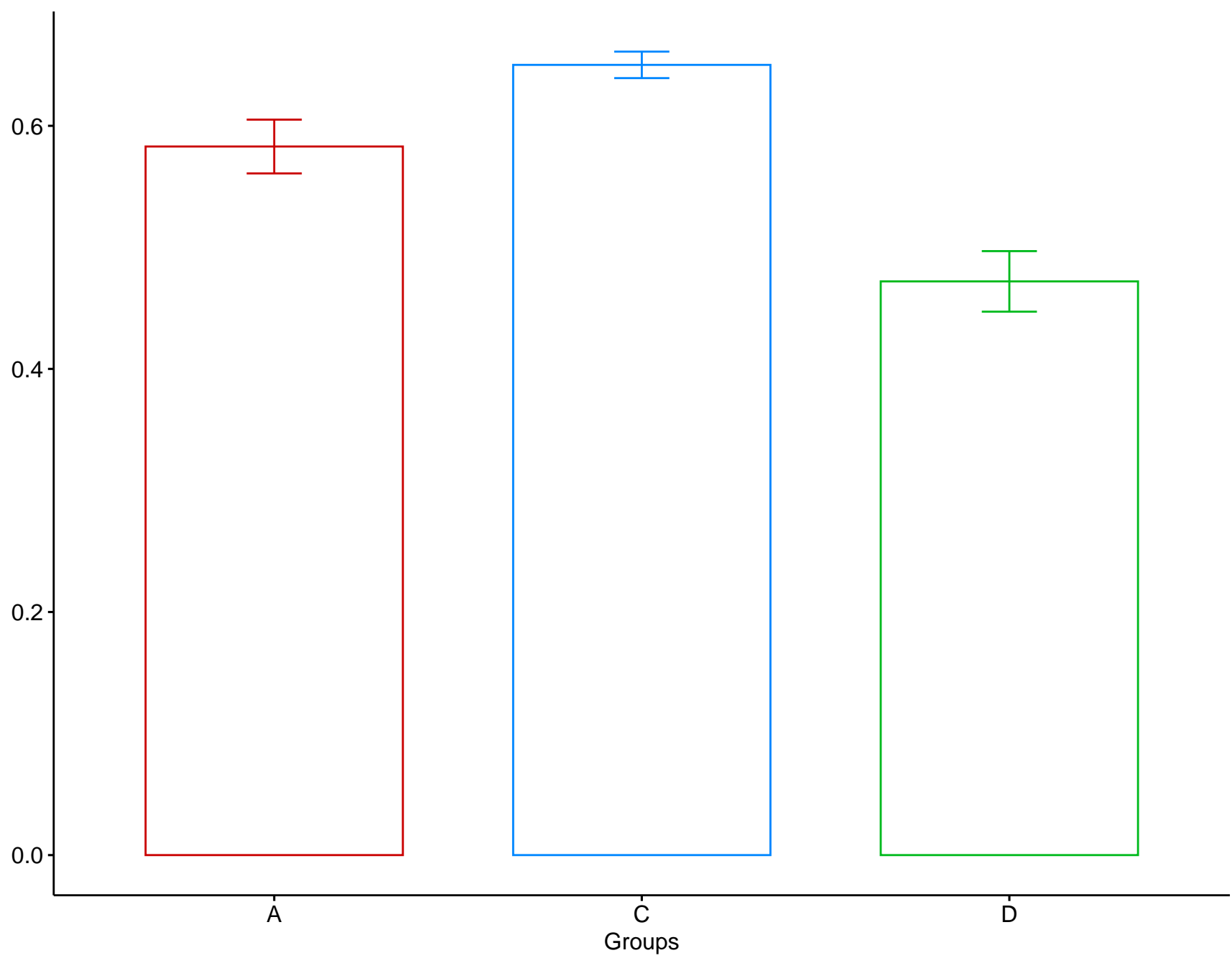

Supplement: S2 File — (ZIP) [file pone.0312147.s002.zip › 3_AlphaDiversity/AlphaIndex/shannoneven_alpha_diversity_meanse.pdf]

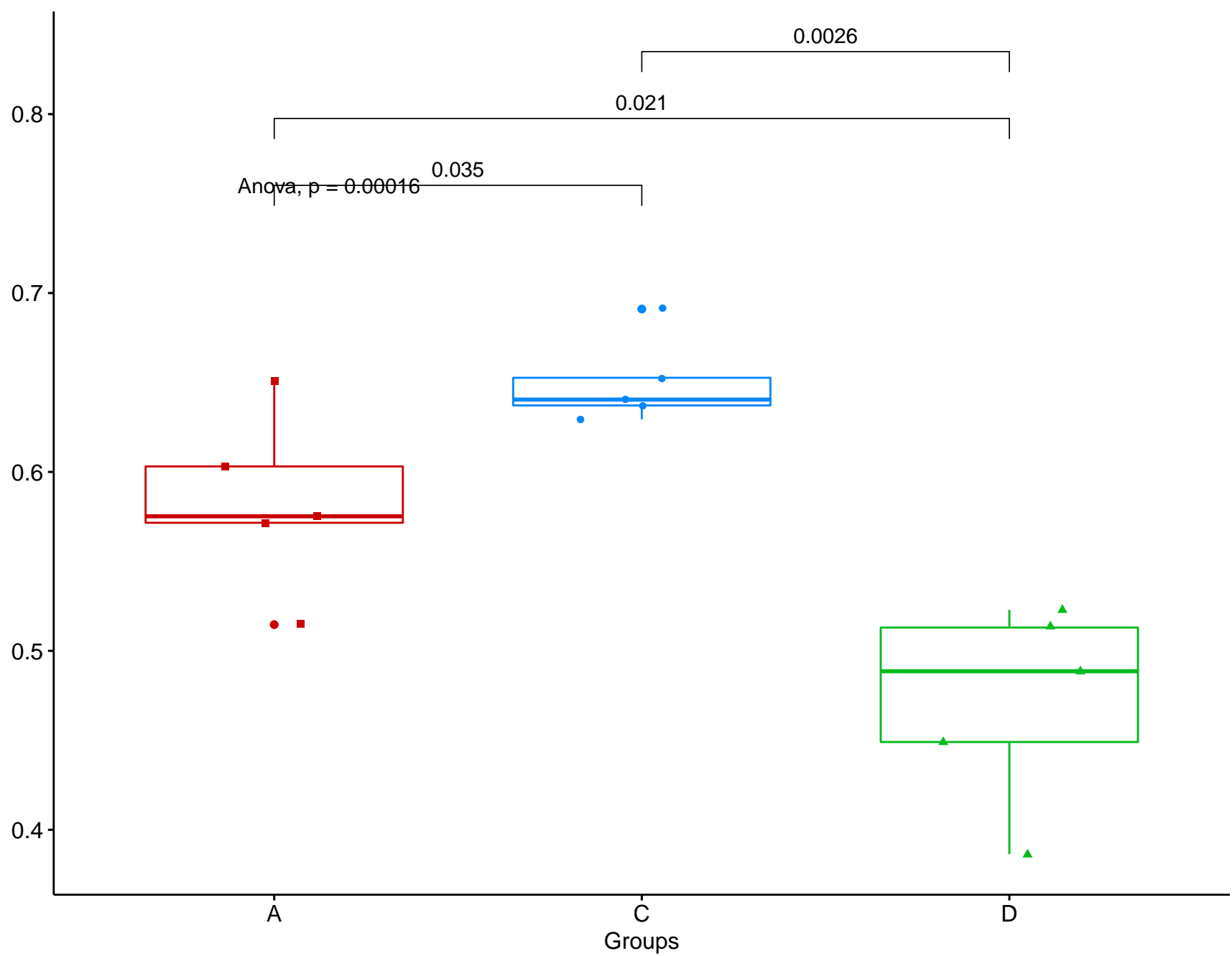

Supplement: S2 File — (ZIP) [file pone.0312147.s002.zip › 3_AlphaDiversity/AlphaIndex/shannoneven_alpha_diversity_test.pdf]

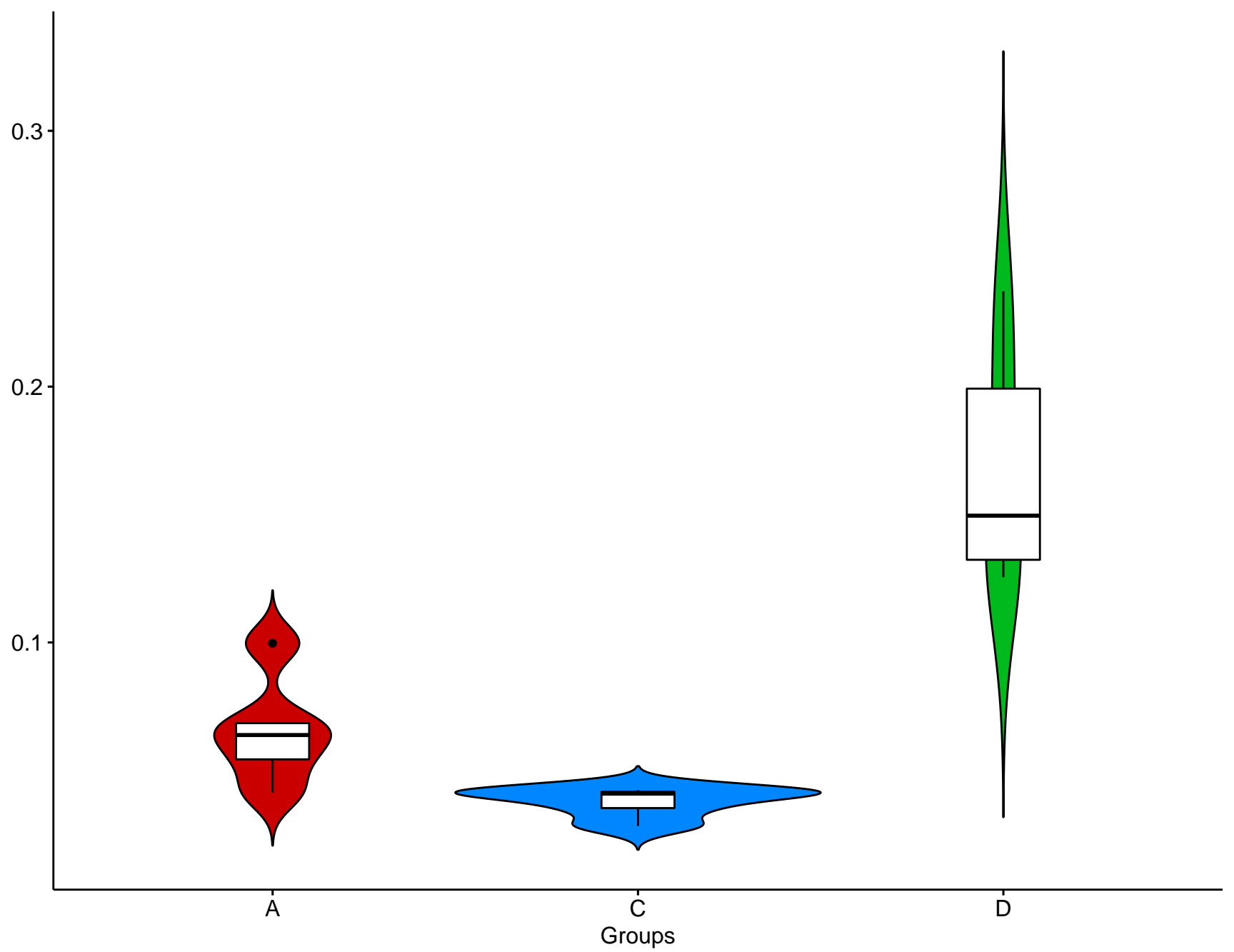

Supplement: S2 File — (ZIP) [file pone.0312147.s002.zip › 3_AlphaDiversity/AlphaIndex/simpson_alpha_diversity_boxplot.pdf]

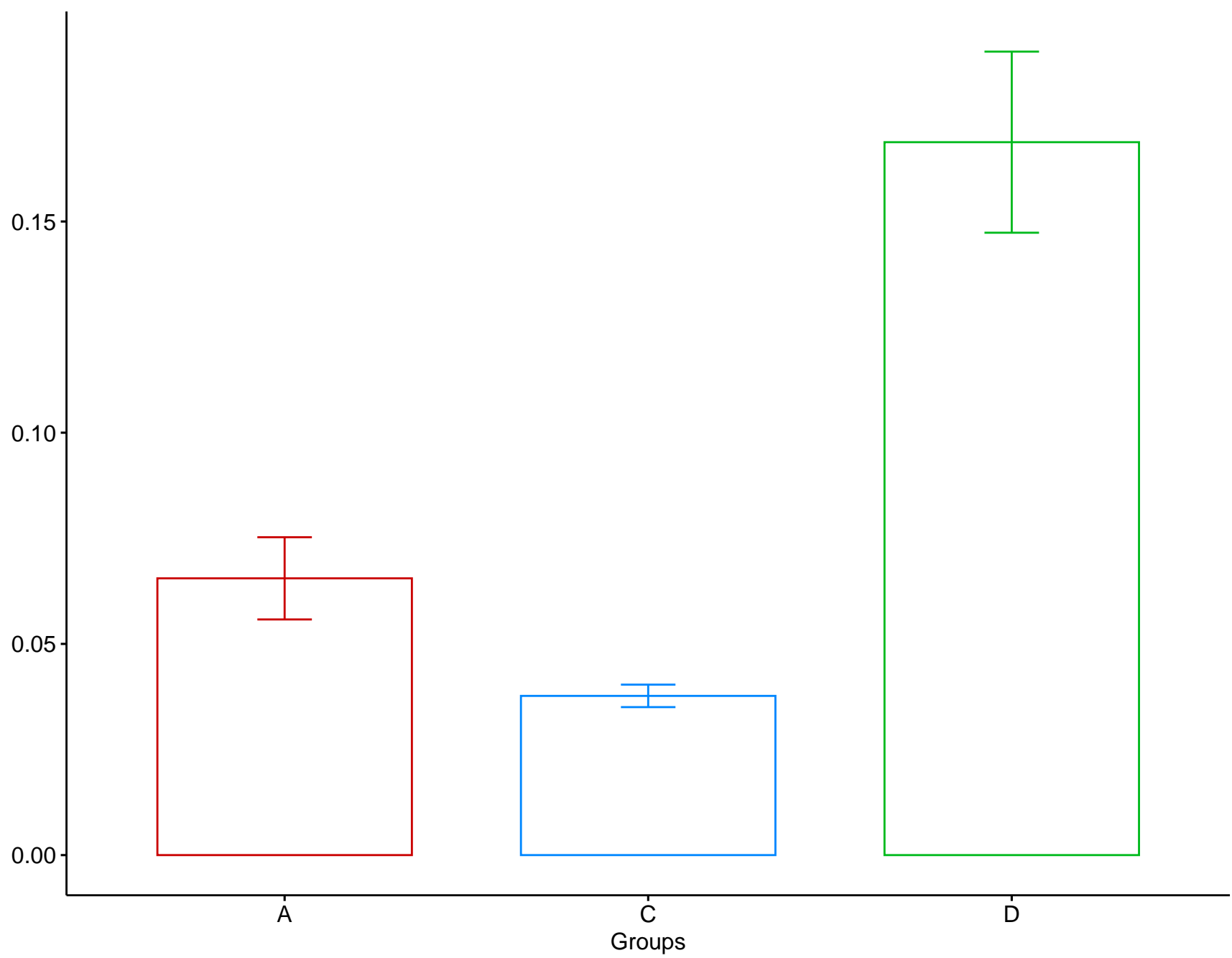

Supplement: S2 File — (ZIP) [file pone.0312147.s002.zip › 3_AlphaDiversity/AlphaIndex/simpson_alpha_diversity_meanse.pdf]

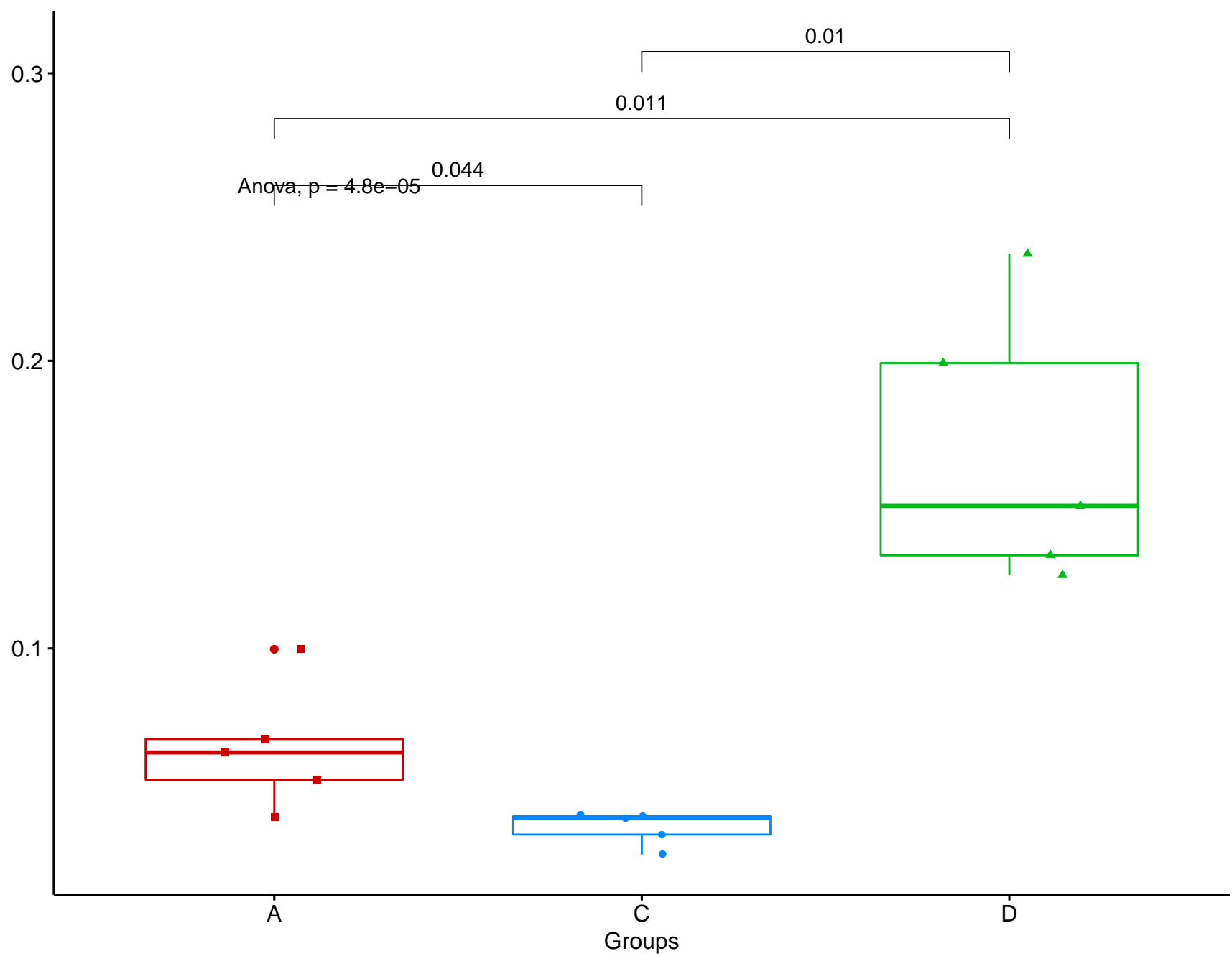

Supplement: S2 File — (ZIP) [file pone.0312147.s002.zip › 3_AlphaDiversity/AlphaIndex/simpson_alpha_diversity_test.pdf]

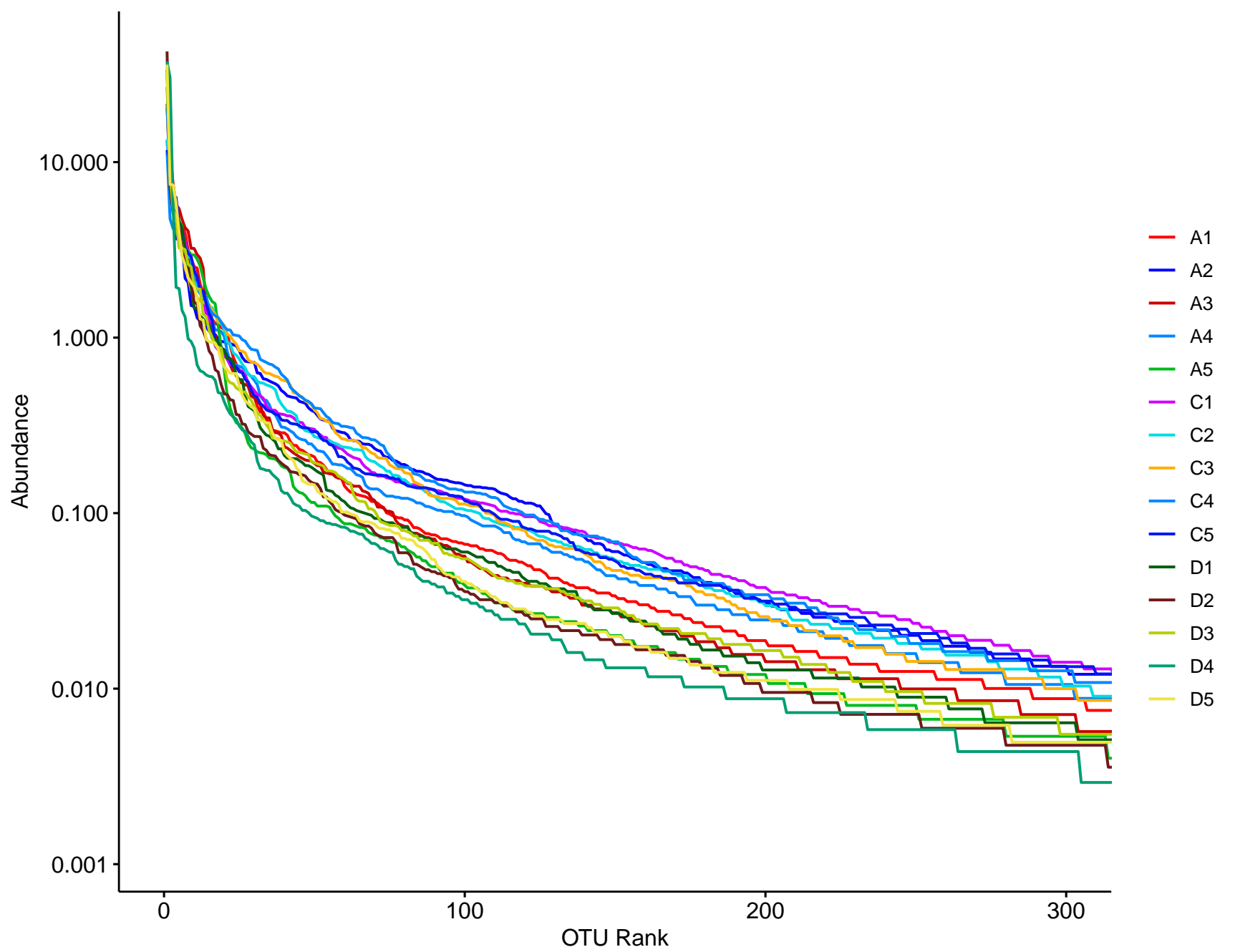

Supplement: S2 File — (ZIP) [file pone.0312147.s002.zip › 3_AlphaDiversity/RankAbundance/rank_abundance.pdf]

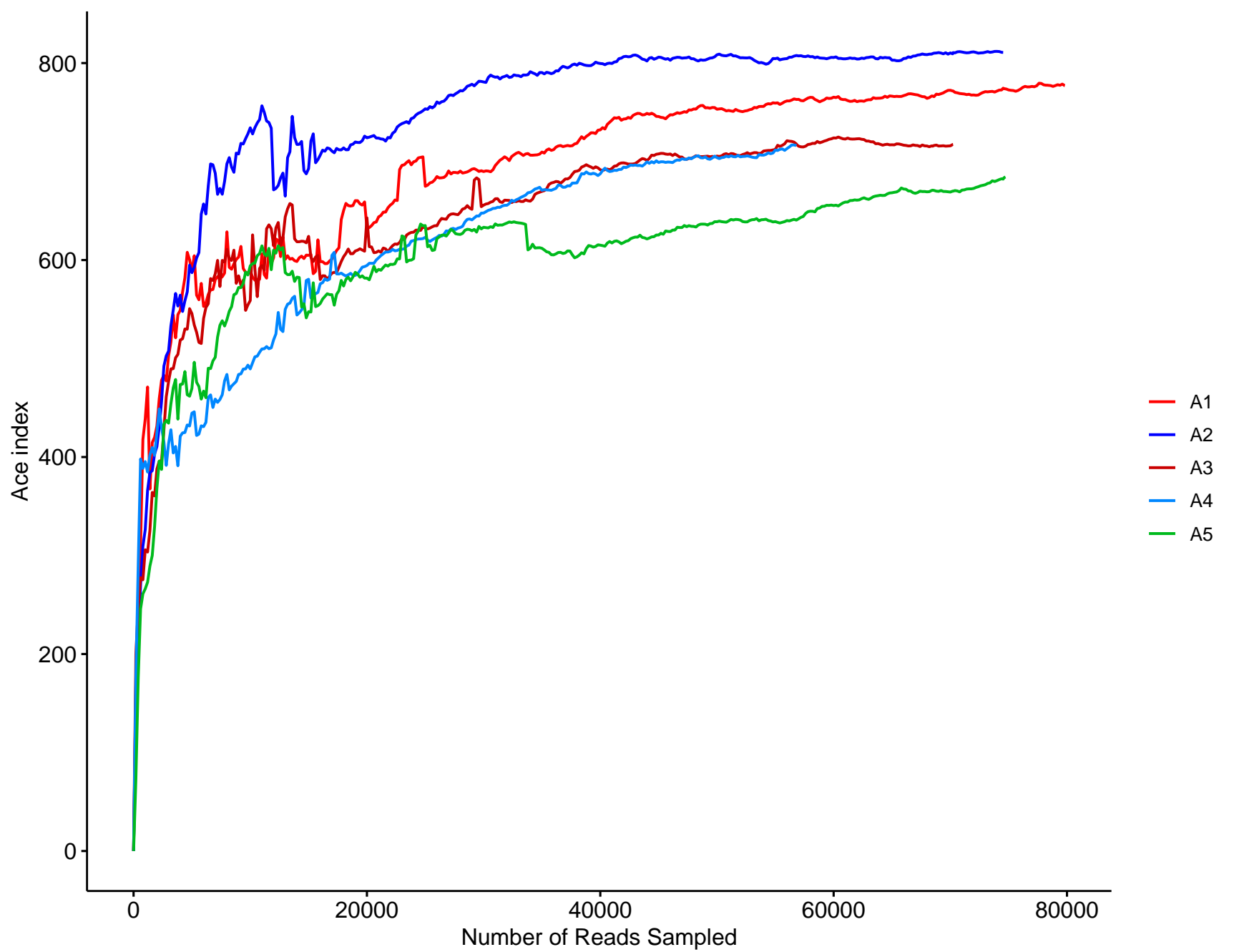

Supplement: S2 File — (ZIP) [file pone.0312147.s002.zip › 3_AlphaDiversity/Rarefaction/A.ace_rarefaction.pdf]

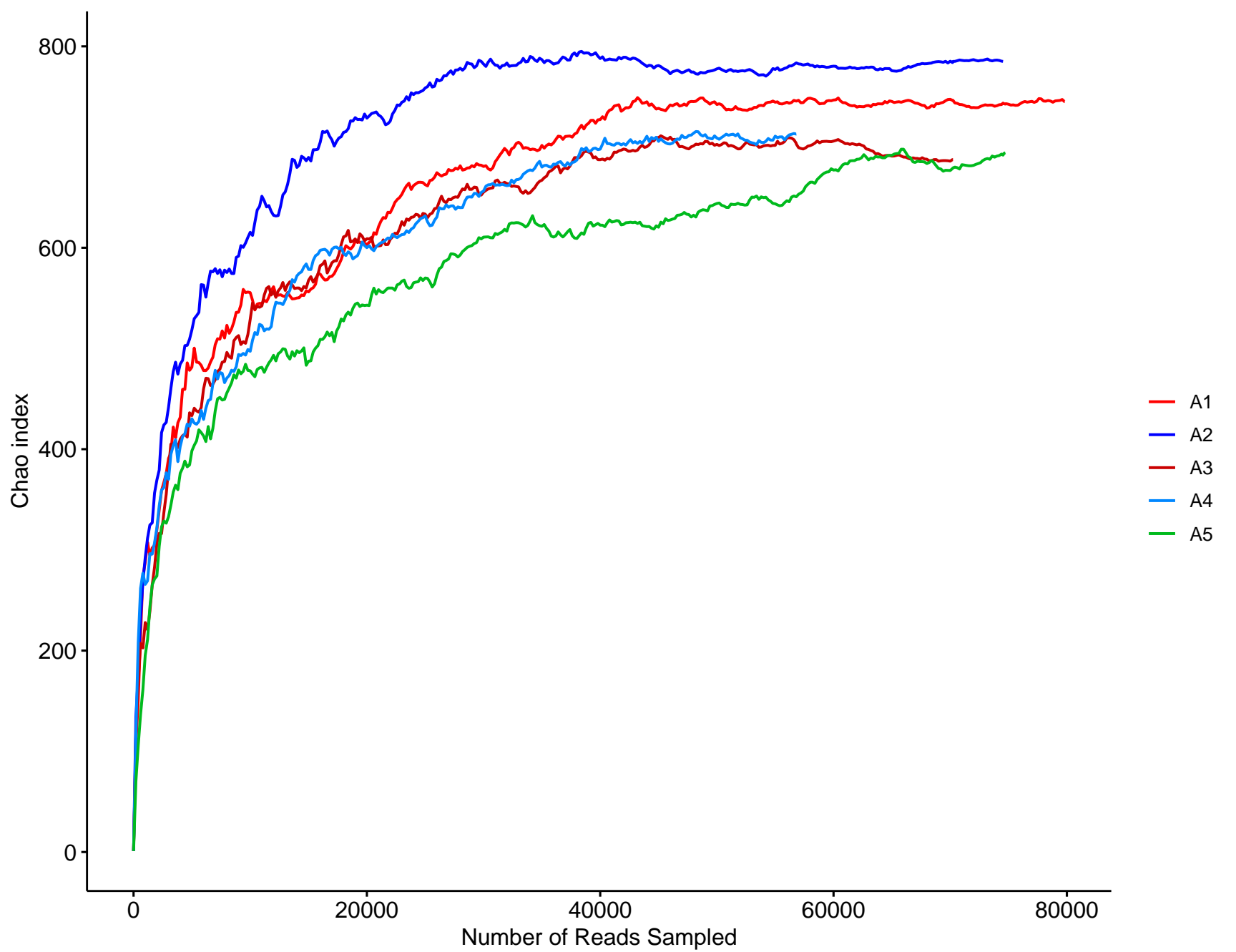

Supplement: S2 File — (ZIP) [file pone.0312147.s002.zip › 3_AlphaDiversity/Rarefaction/A.chao_rarefaction.pdf]

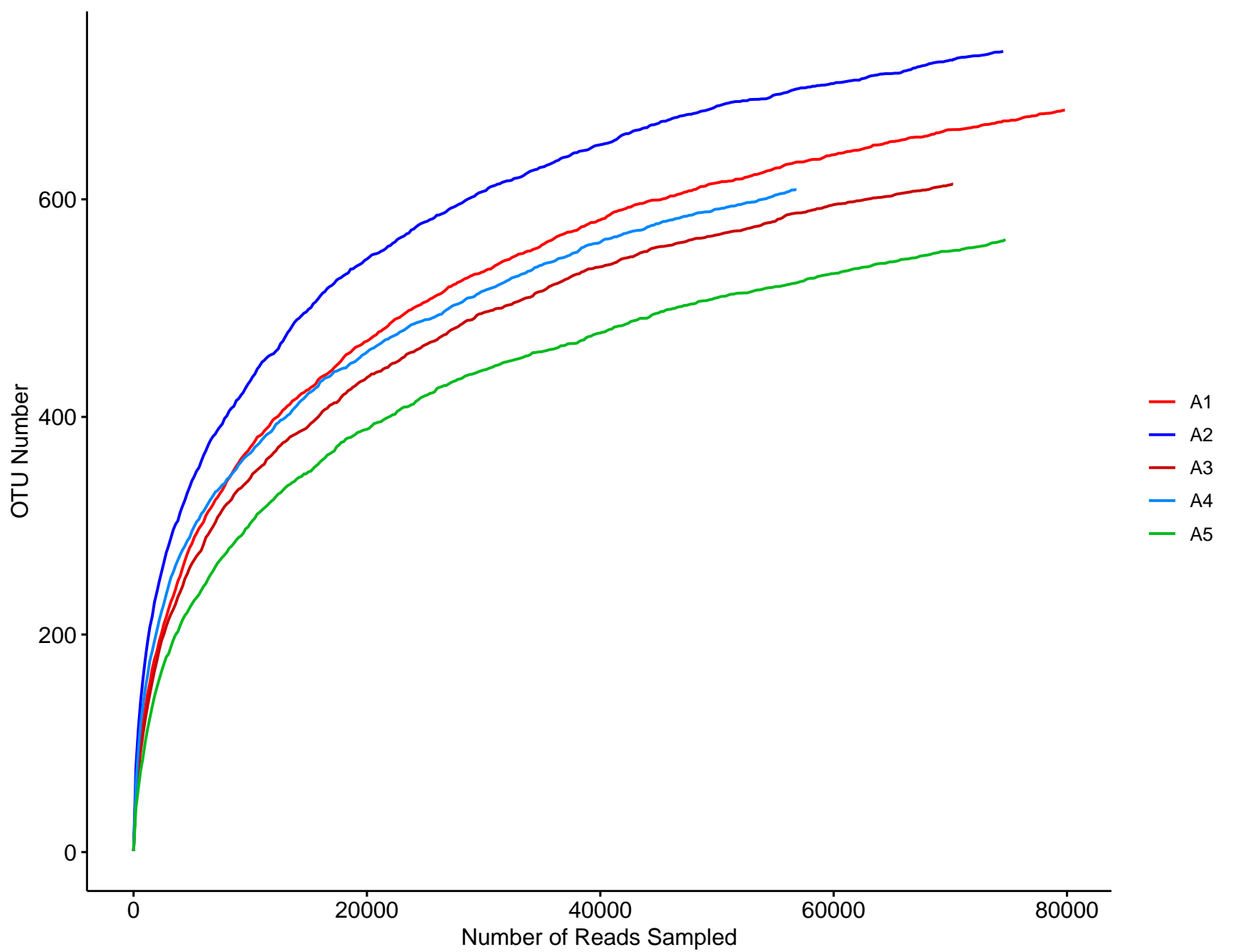

Supplement: S2 File — (ZIP) [file pone.0312147.s002.zip › 3_AlphaDiversity/Rarefaction/A.otu_rarefaction.pdf]

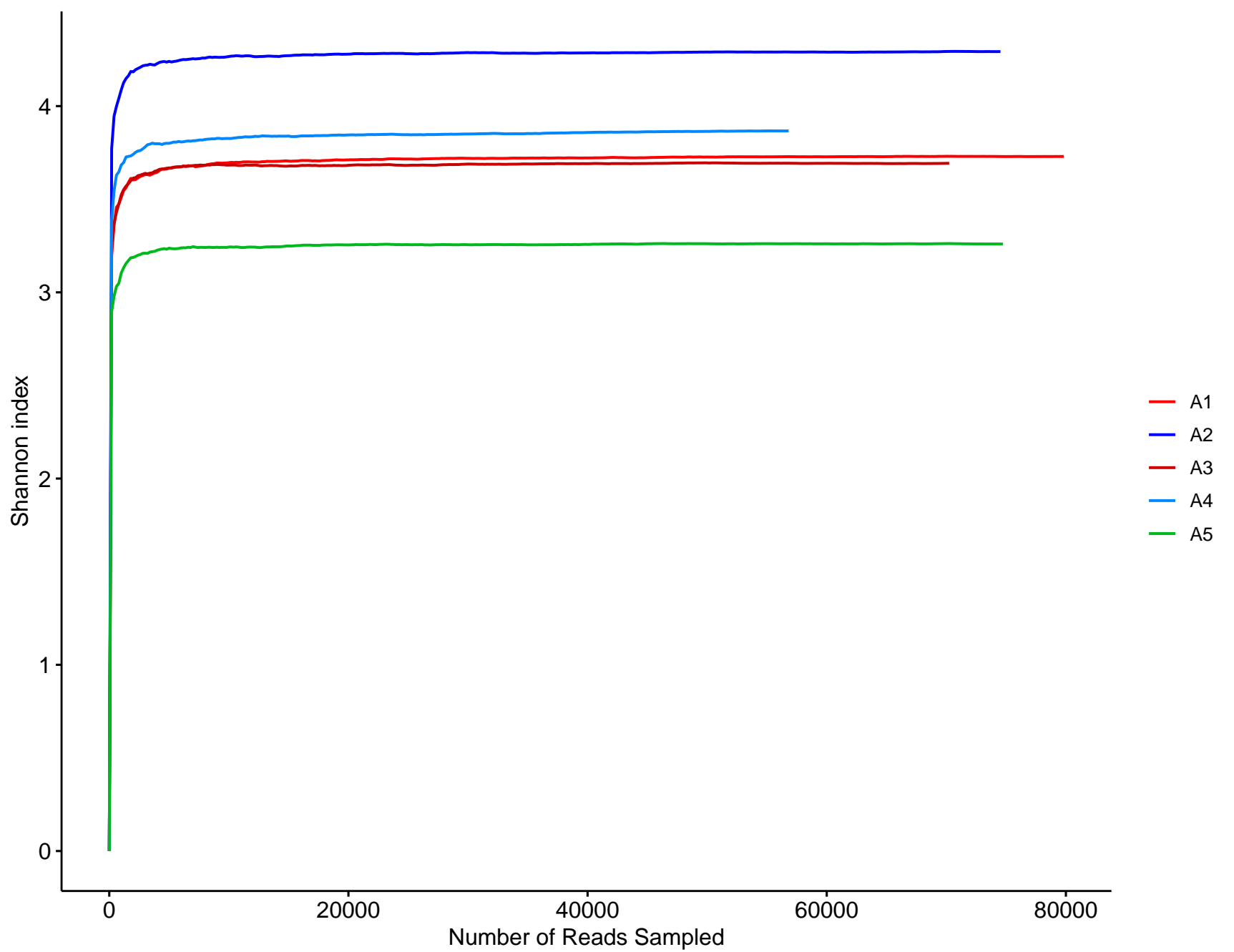

Supplement: S2 File — (ZIP) [file pone.0312147.s002.zip › 3_AlphaDiversity/Rarefaction/A.shannon_rarefaction.pdf]

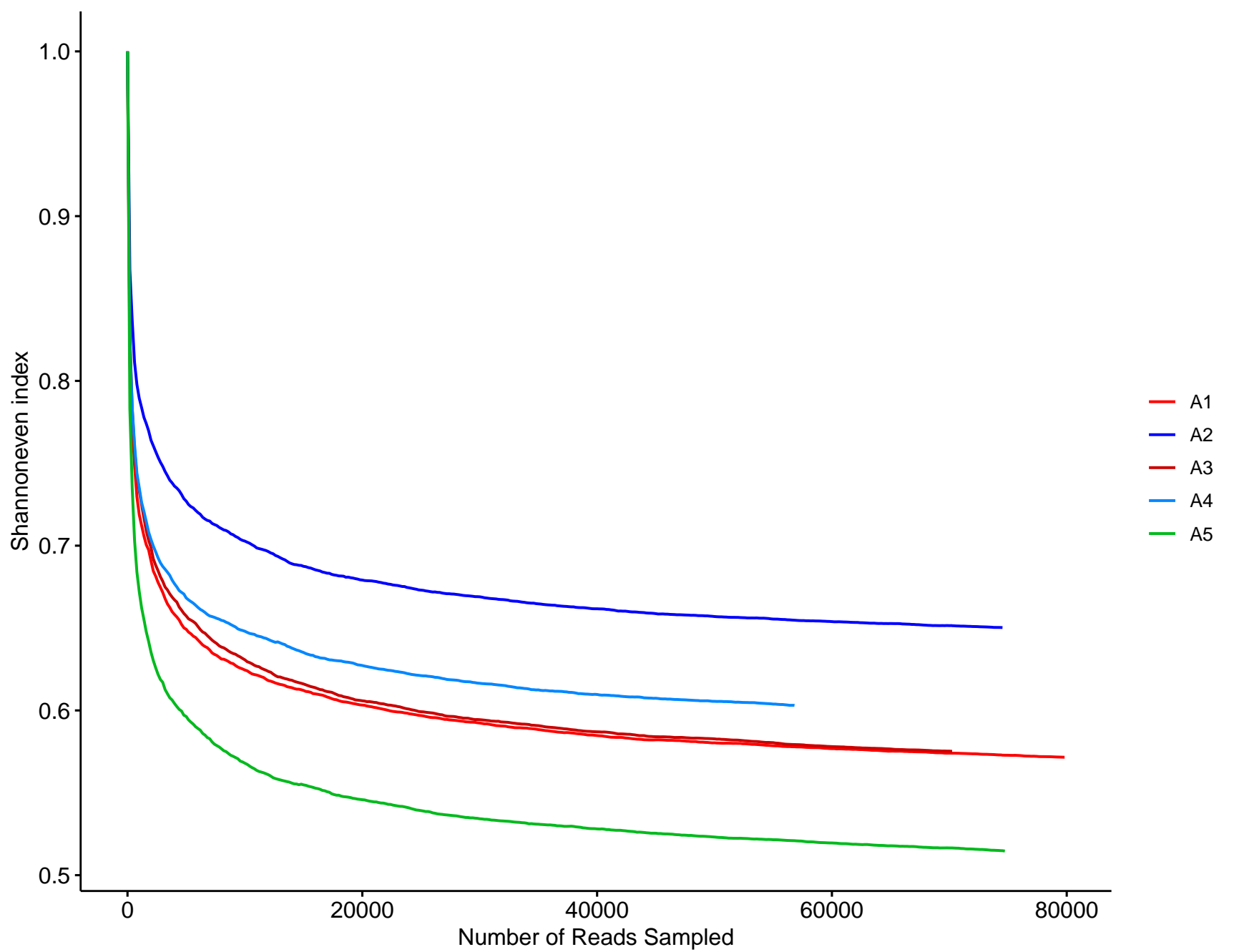

Supplement: S2 File — (ZIP) [file pone.0312147.s002.zip › 3_AlphaDiversity/Rarefaction/A.shannoneven_rarefaction.pdf]

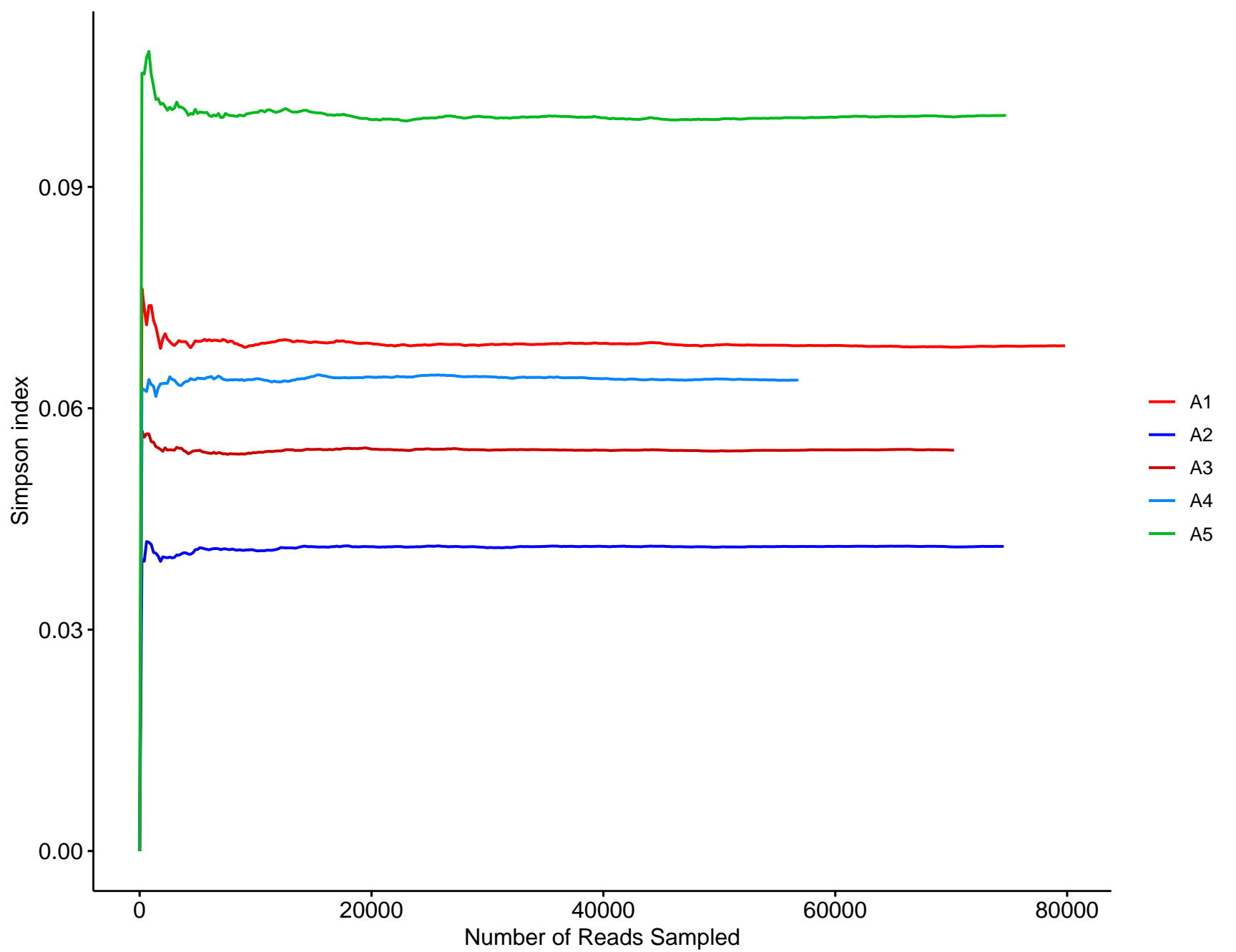

Supplement: S2 File — (ZIP) [file pone.0312147.s002.zip › 3_AlphaDiversity/Rarefaction/A.simpson_rarefaction.pdf]

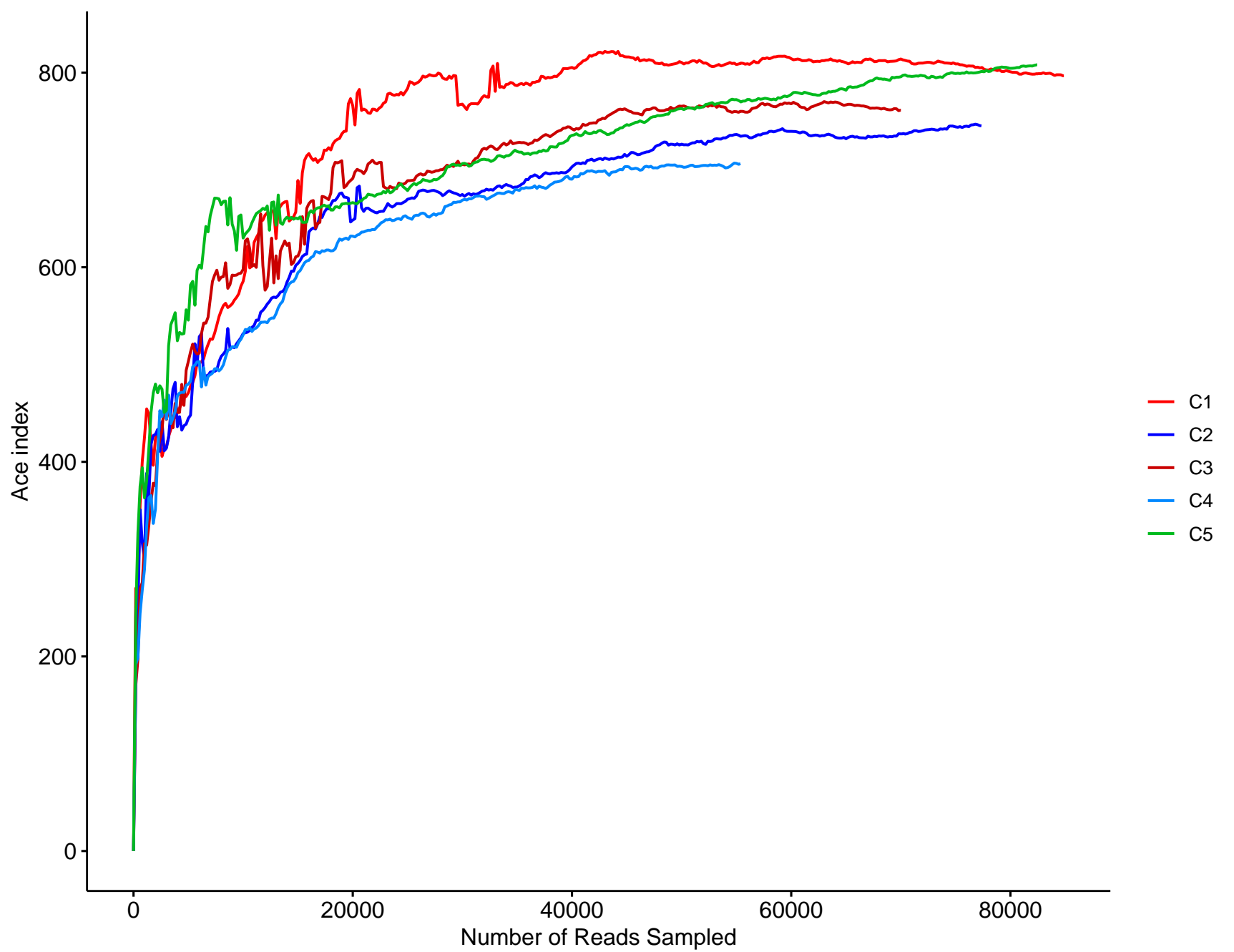

Supplement: S2 File — (ZIP) [file pone.0312147.s002.zip › 3_AlphaDiversity/Rarefaction/C.ace_rarefaction.pdf]

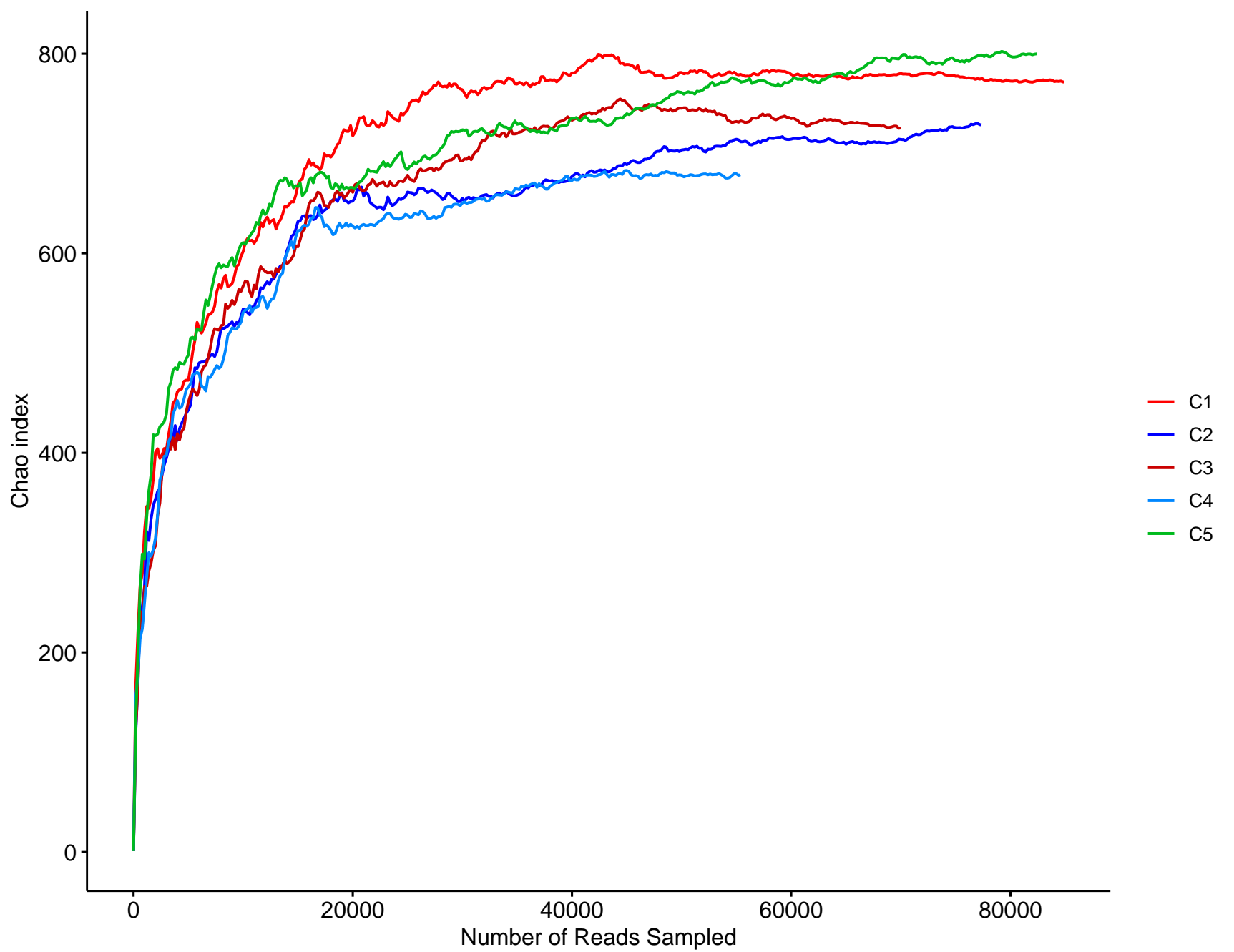

Supplement: S2 File — (ZIP) [file pone.0312147.s002.zip › 3_AlphaDiversity/Rarefaction/C.chao_rarefaction.pdf]

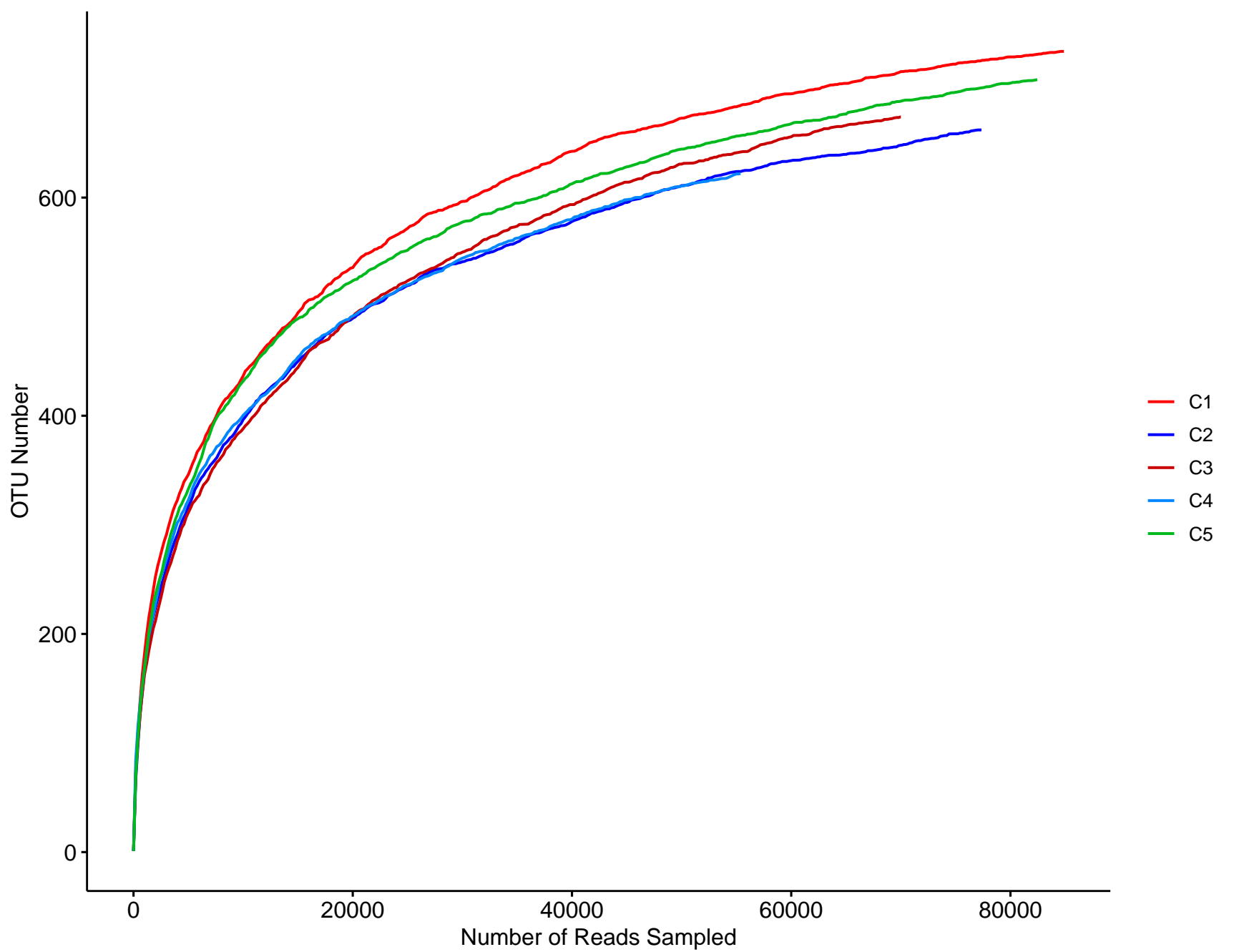

Supplement: S2 File — (ZIP) [file pone.0312147.s002.zip › 3_AlphaDiversity/Rarefaction/C.otu_rarefaction.pdf]

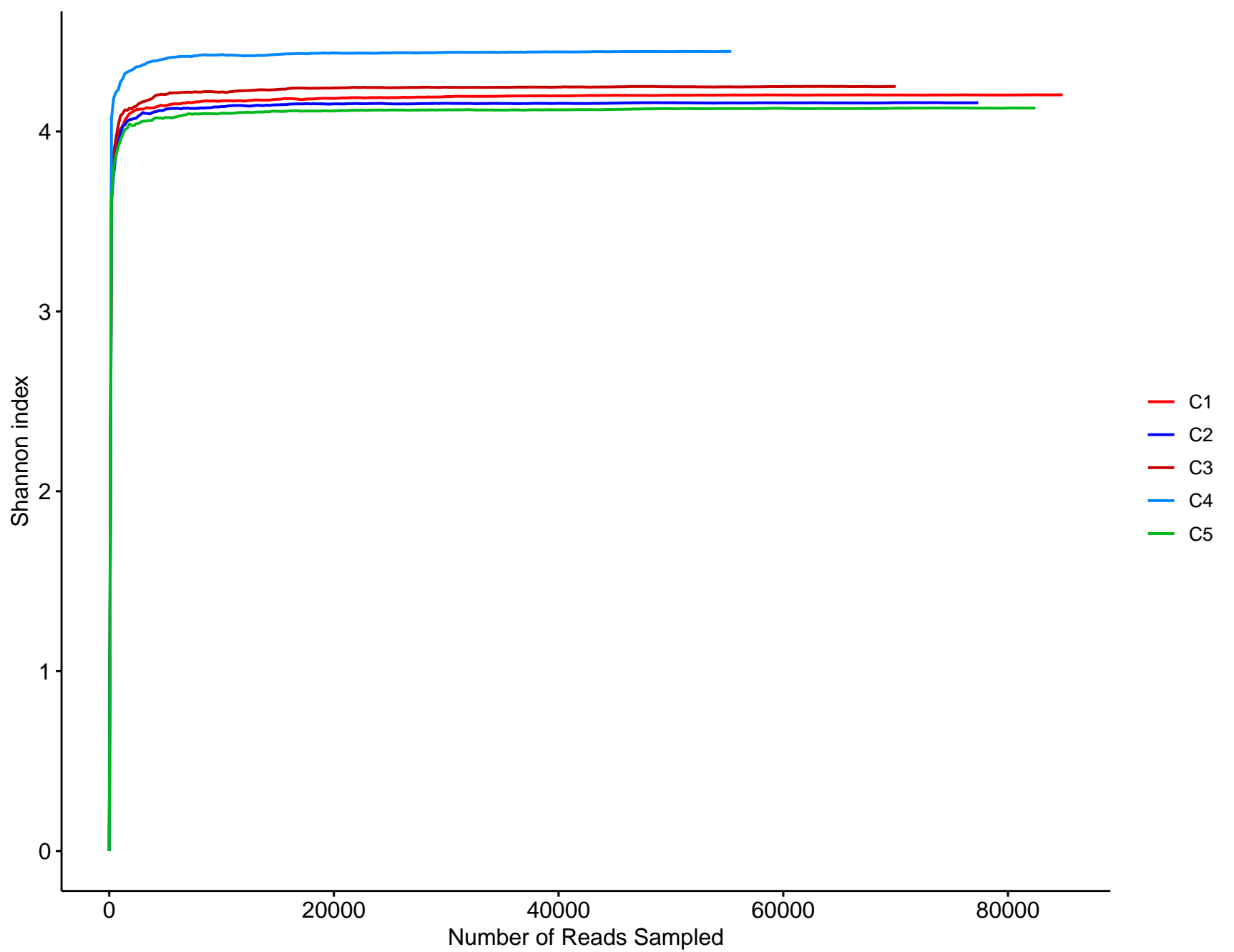

Supplement: S2 File — (ZIP) [file pone.0312147.s002.zip › 3_AlphaDiversity/Rarefaction/C.shannon_rarefaction.pdf]

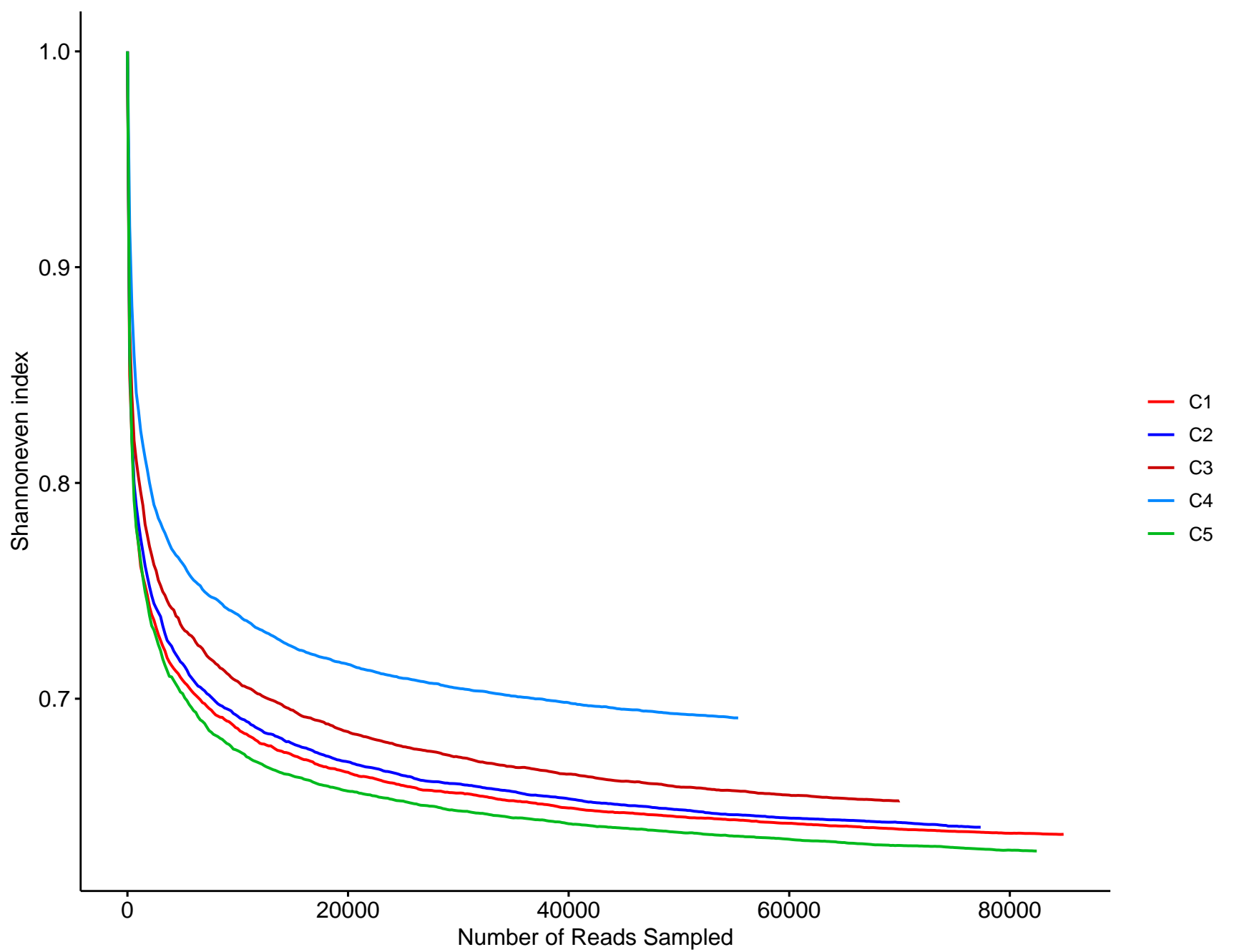

Supplement: S2 File — (ZIP) [file pone.0312147.s002.zip › 3_AlphaDiversity/Rarefaction/C.shannoneven_rarefaction.pdf]

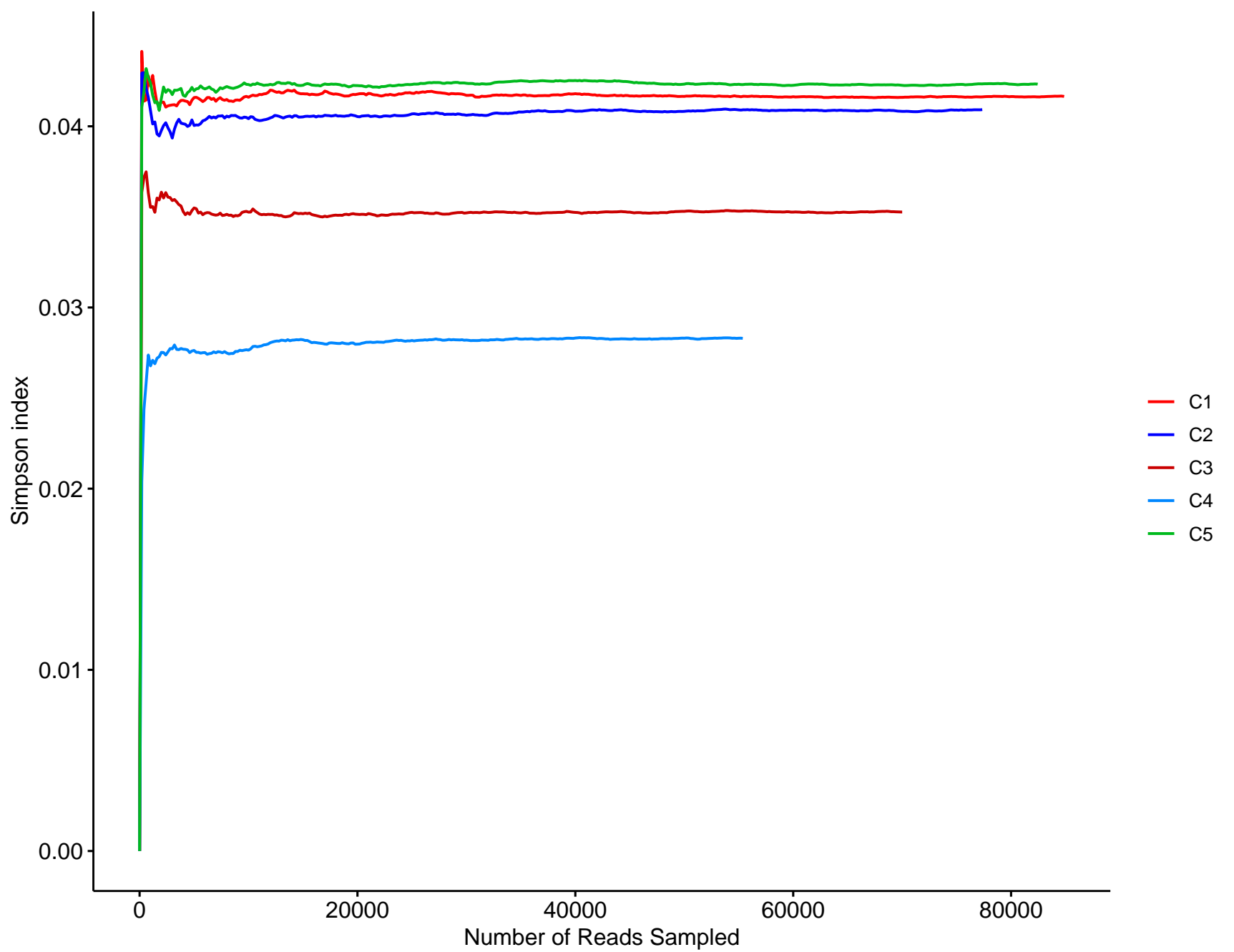

Supplement: S2 File — (ZIP) [file pone.0312147.s002.zip › 3_AlphaDiversity/Rarefaction/C.simpson_rarefaction.pdf]

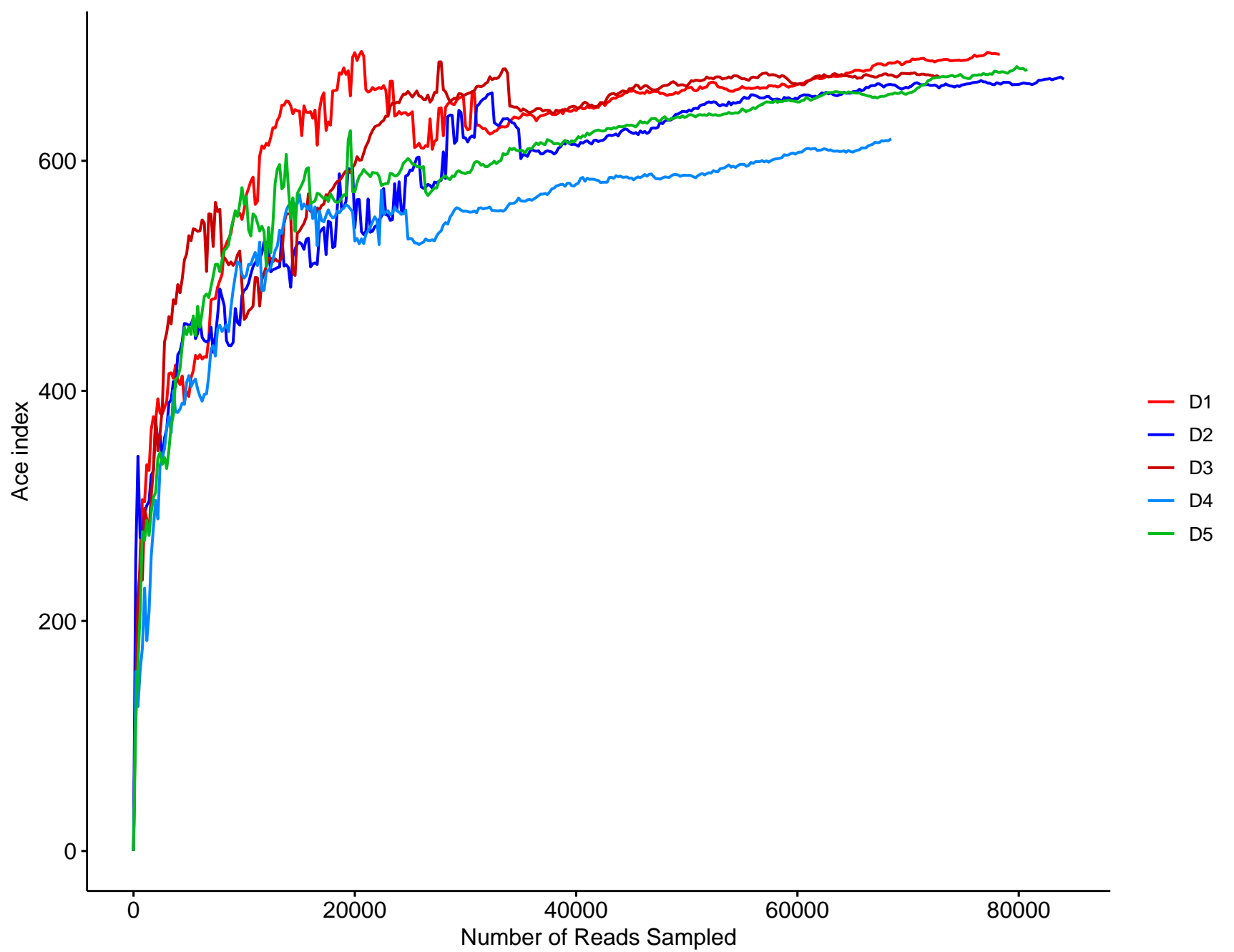

Supplement: S2 File — (ZIP) [file pone.0312147.s002.zip › 3_AlphaDiversity/Rarefaction/D.ace_rarefaction.pdf]

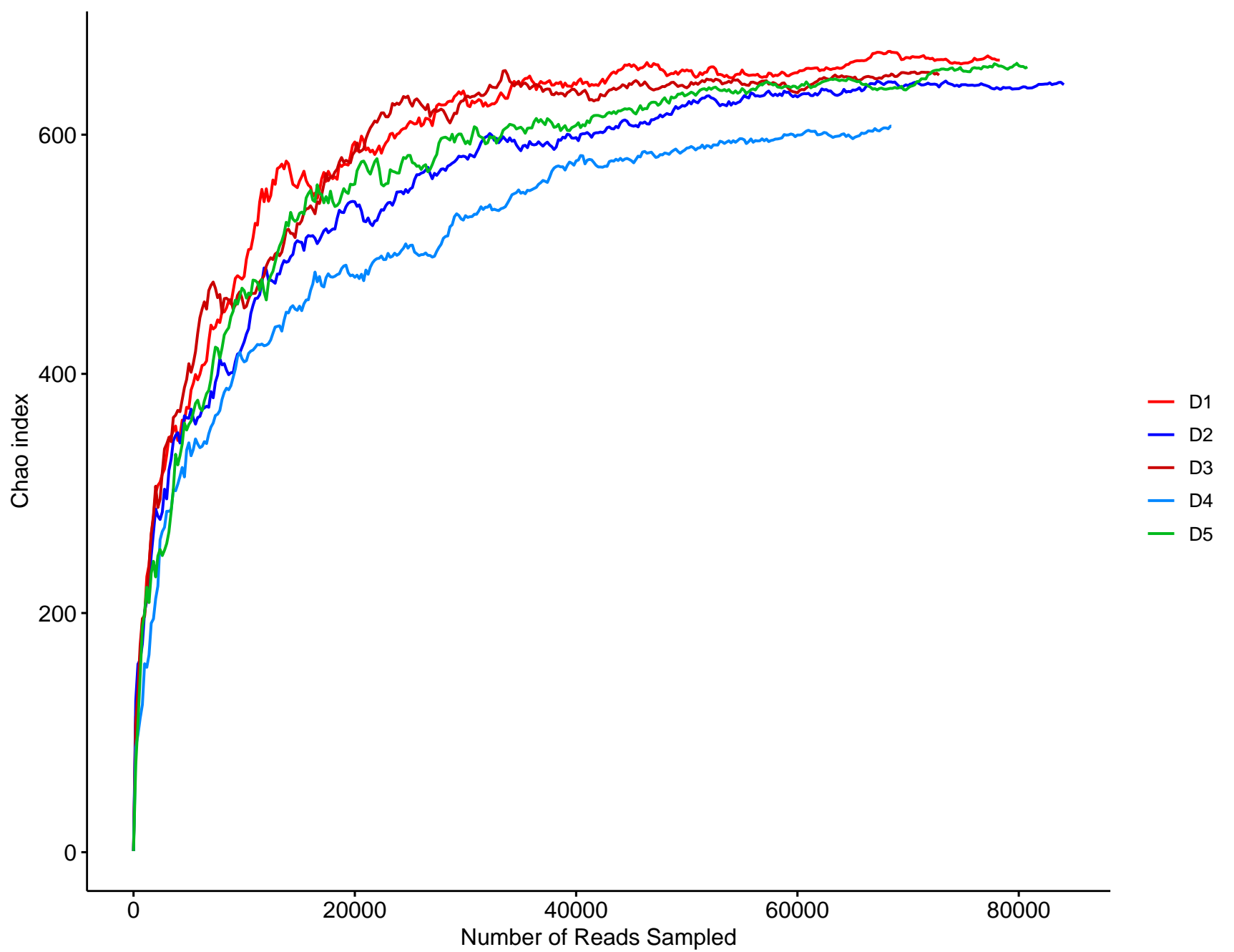

Supplement: S2 File — (ZIP) [file pone.0312147.s002.zip › 3_AlphaDiversity/Rarefaction/D.chao_rarefaction.pdf]

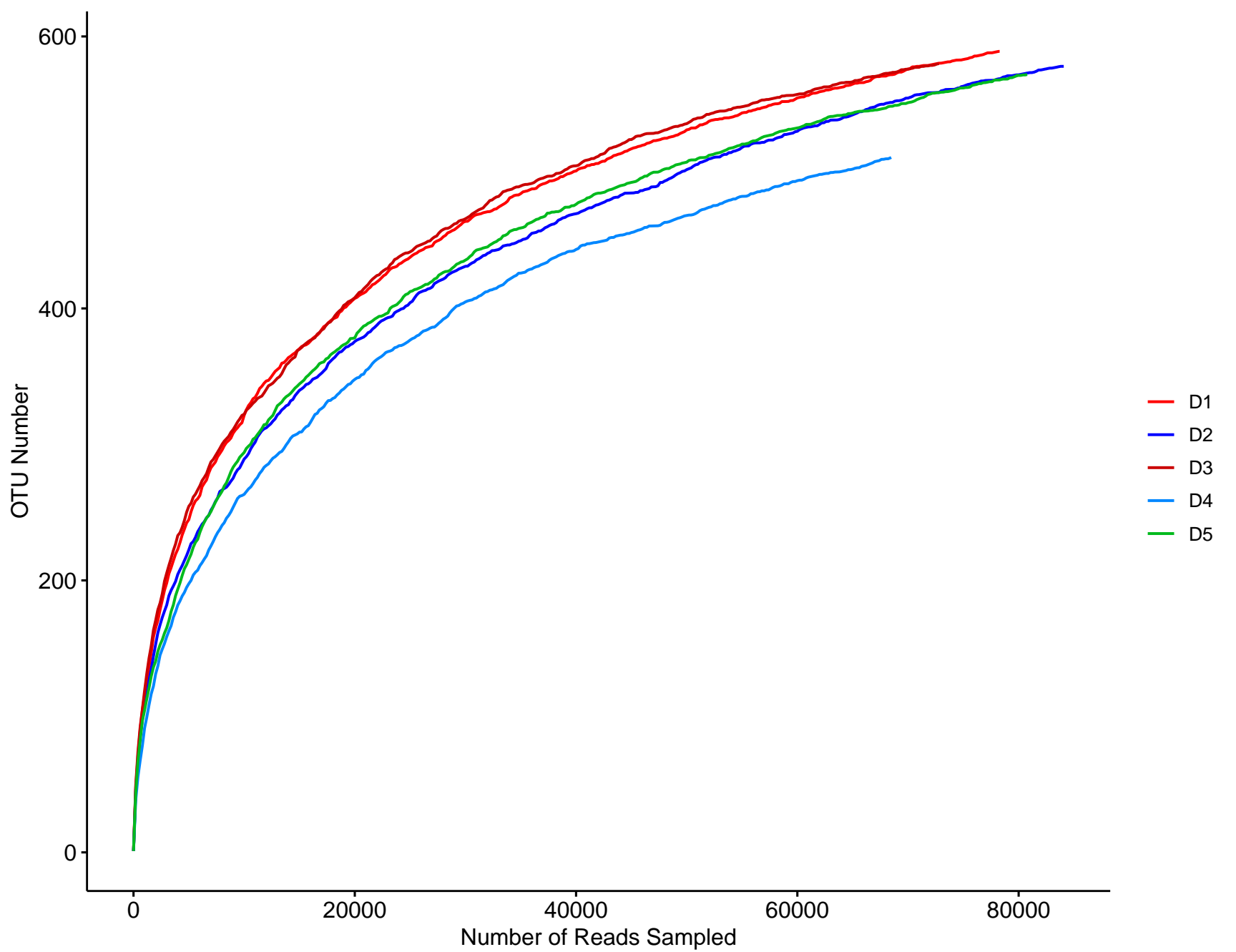

Supplement: S2 File — (ZIP) [file pone.0312147.s002.zip › 3_AlphaDiversity/Rarefaction/D.otu_rarefaction.pdf]

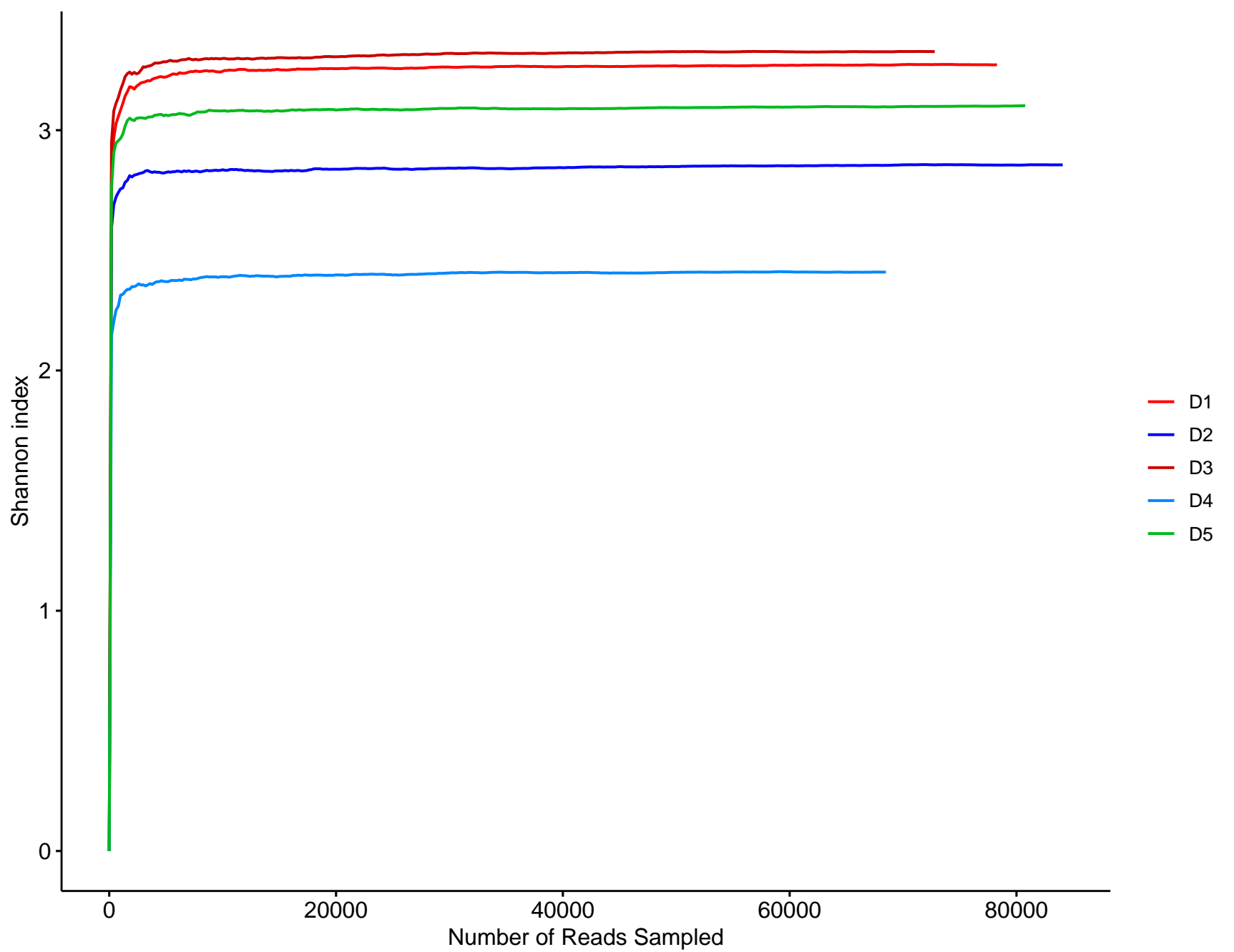

Supplement: S2 File — (ZIP) [file pone.0312147.s002.zip › 3_AlphaDiversity/Rarefaction/D.shannon_rarefaction.pdf]

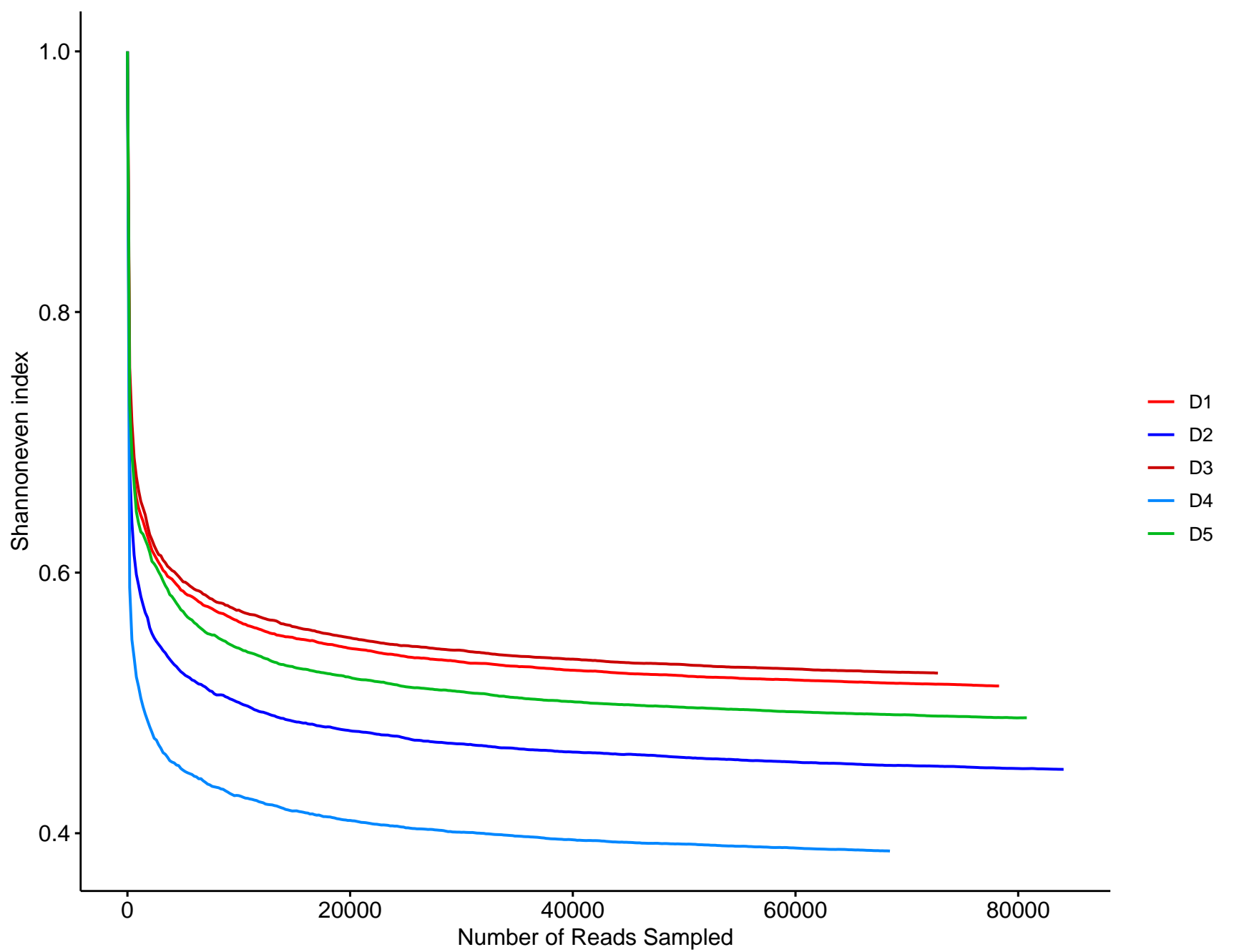

Supplement: S2 File — (ZIP) [file pone.0312147.s002.zip › 3_AlphaDiversity/Rarefaction/D.shannoneven_rarefaction.pdf]

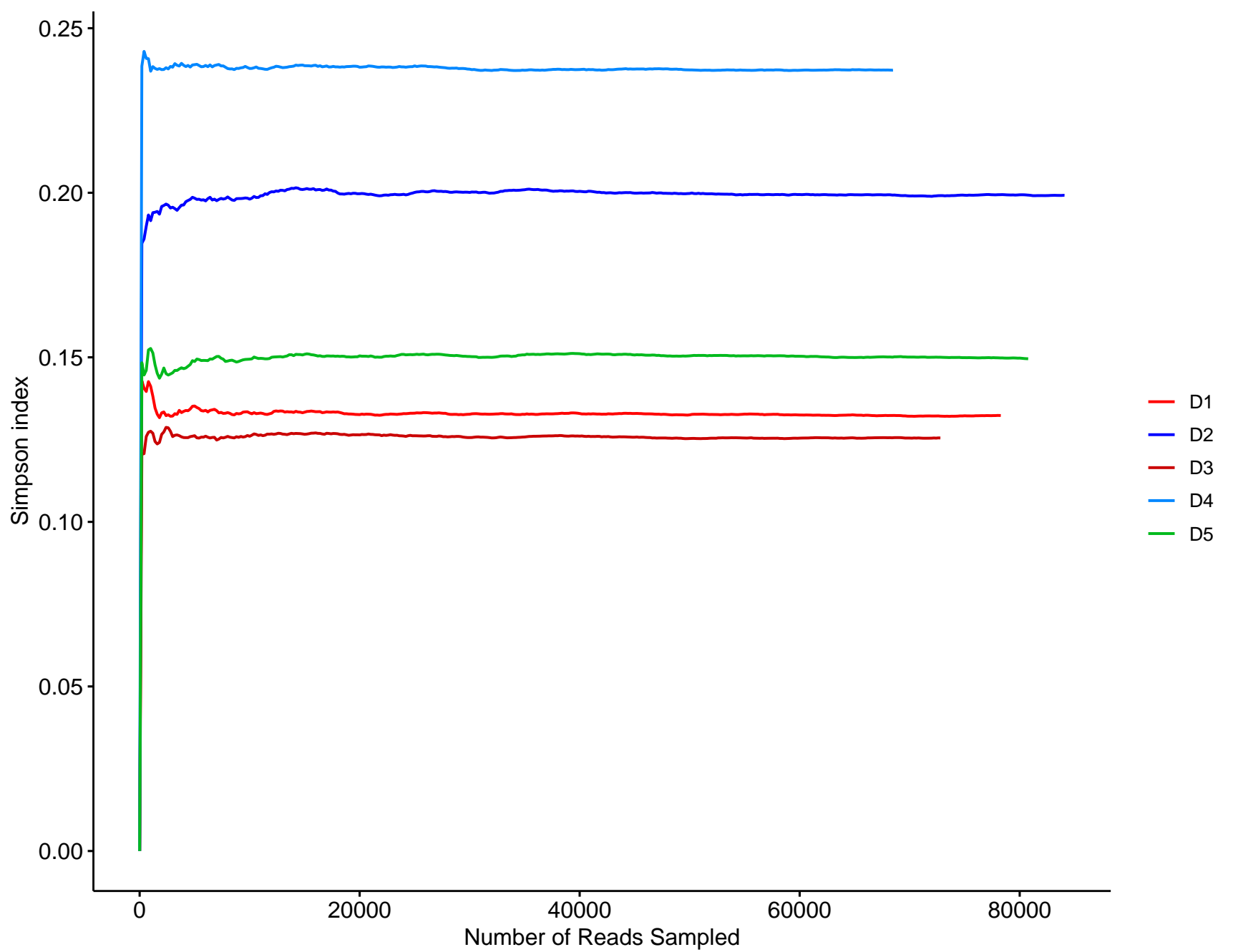

Supplement: S2 File — (ZIP) [file pone.0312147.s002.zip › 3_AlphaDiversity/Rarefaction/D.simpson_rarefaction.pdf]

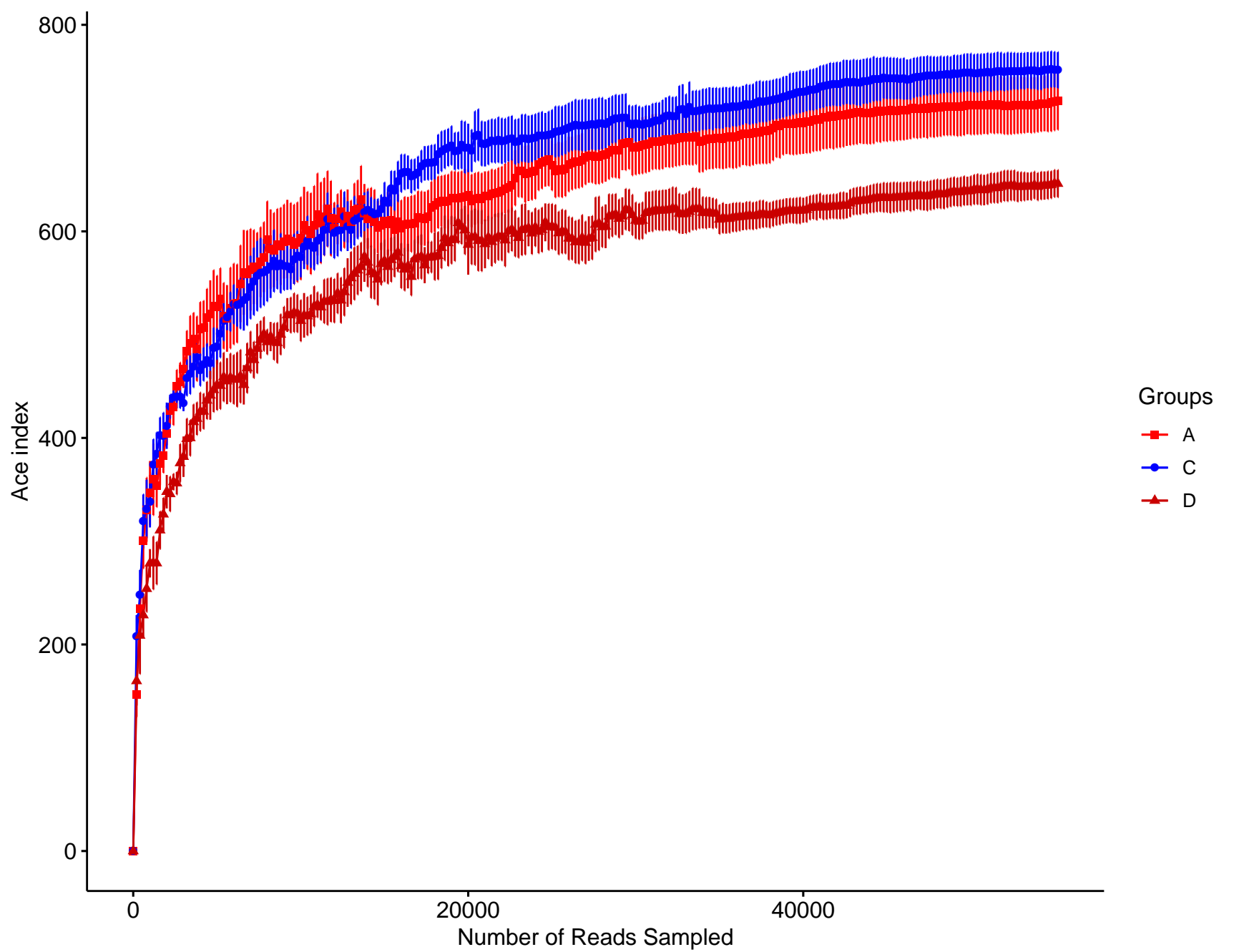

Supplement: S2 File — (ZIP) [file pone.0312147.s002.zip › 3_AlphaDiversity/Rarefaction/ace_rarefaction.groups.pdf]

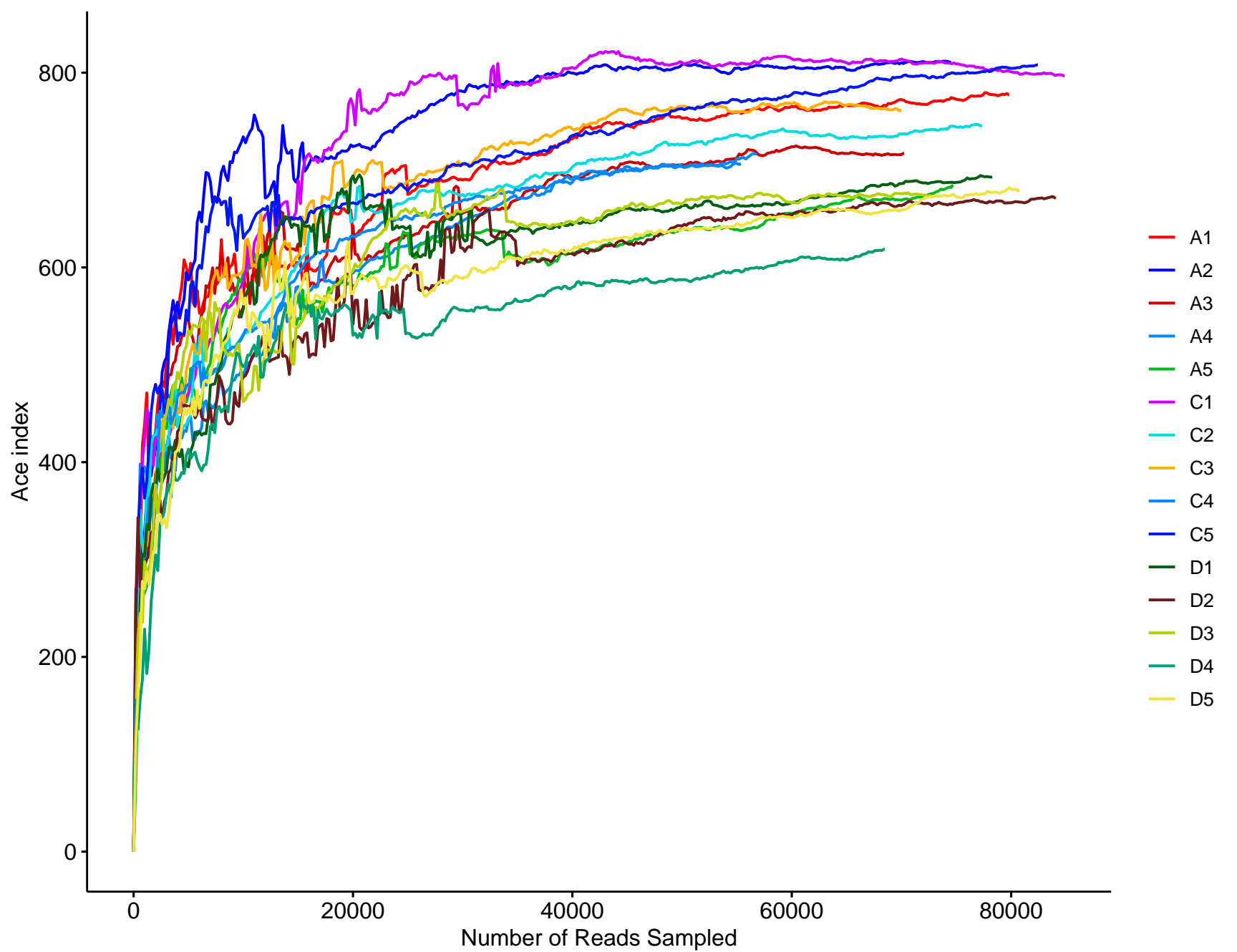

Supplement: S2 File — (ZIP) [file pone.0312147.s002.zip › 3_AlphaDiversity/Rarefaction/ace_rarefaction.pdf]

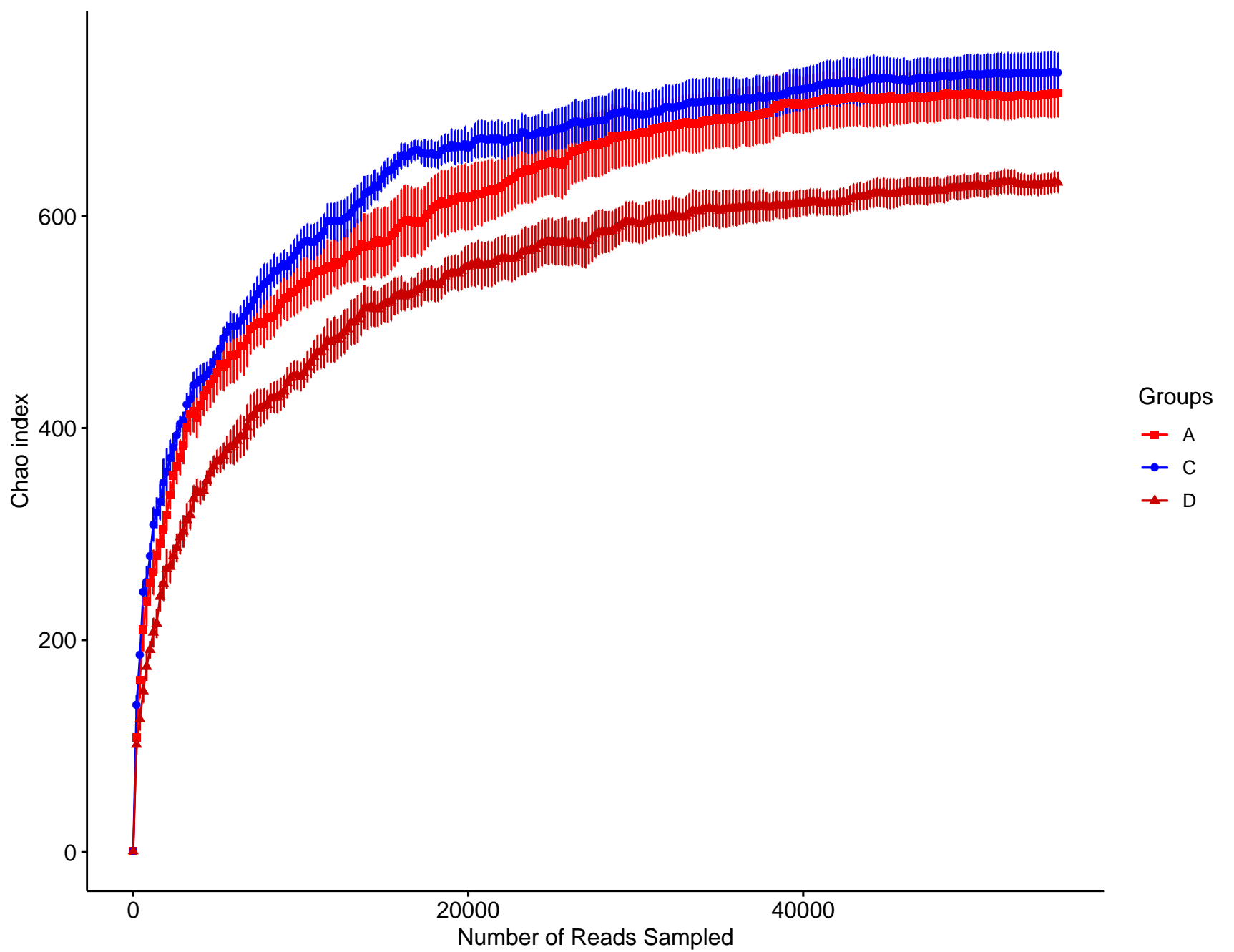

Supplement: S2 File — (ZIP) [file pone.0312147.s002.zip › 3_AlphaDiversity/Rarefaction/chao_rarefaction.groups.pdf]

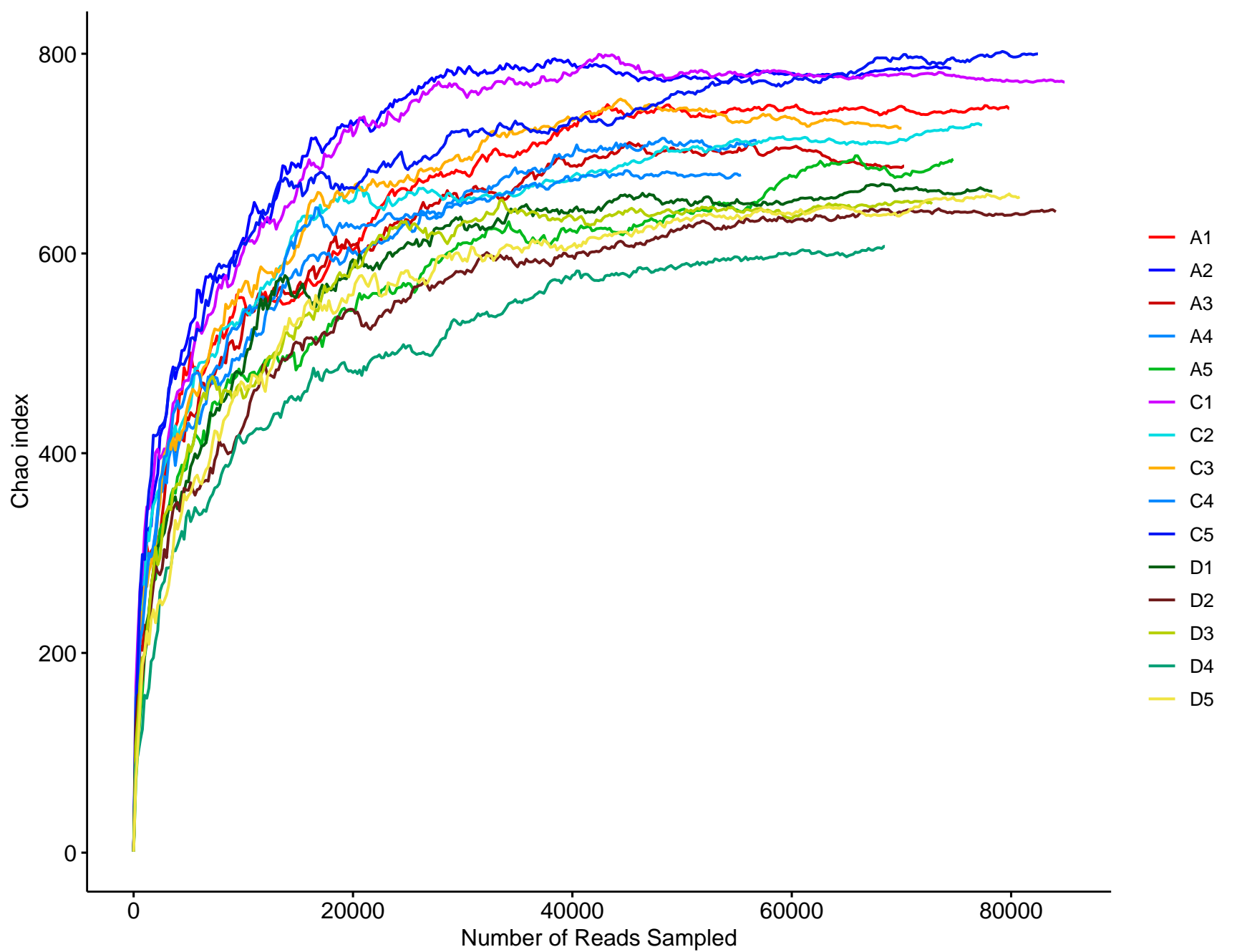

Supplement: S2 File — (ZIP) [file pone.0312147.s002.zip › 3_AlphaDiversity/Rarefaction/chao_rarefaction.pdf]

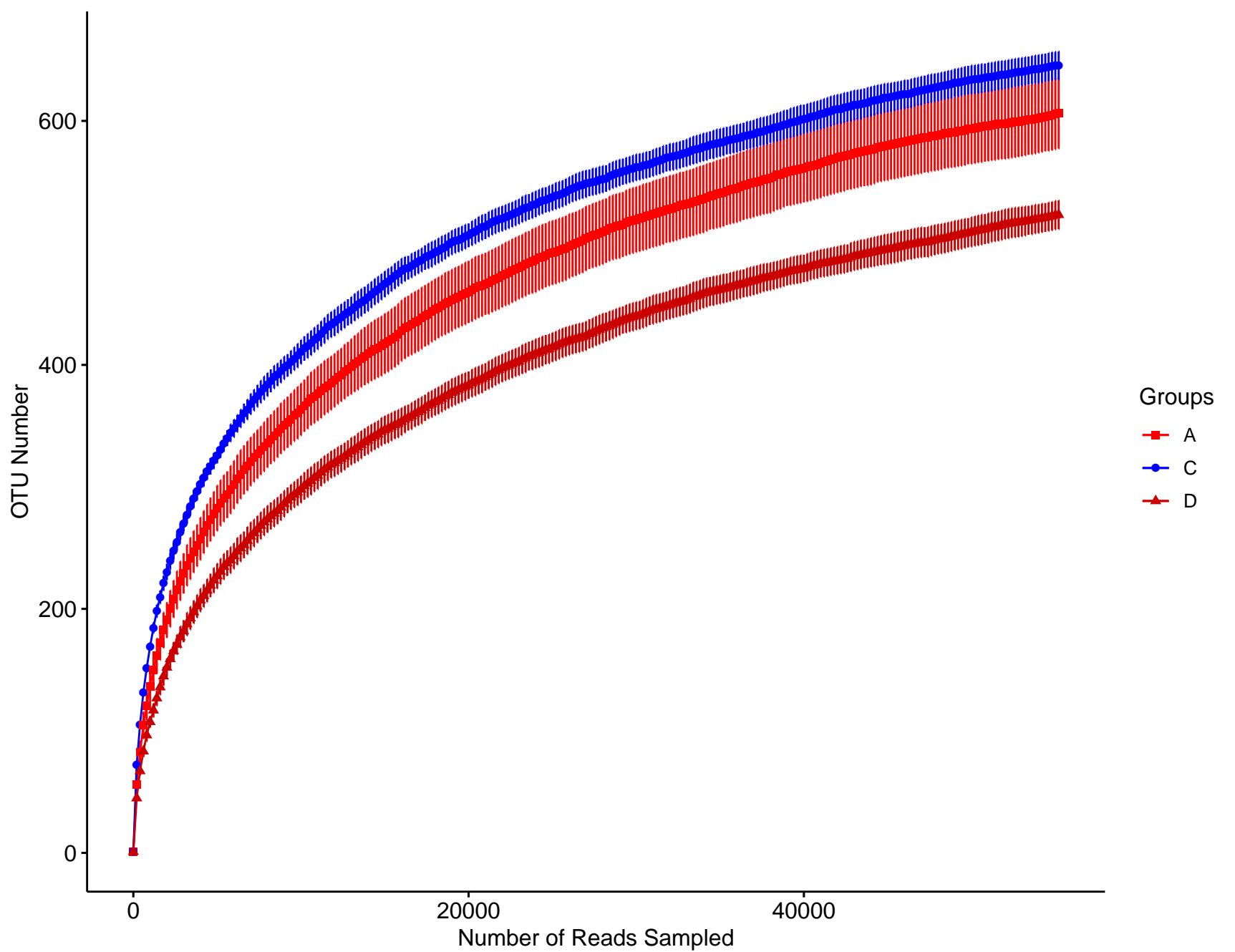

Supplement: S2 File — (ZIP) [file pone.0312147.s002.zip › 3_AlphaDiversity/Rarefaction/otu_rarefaction.groups.pdf]

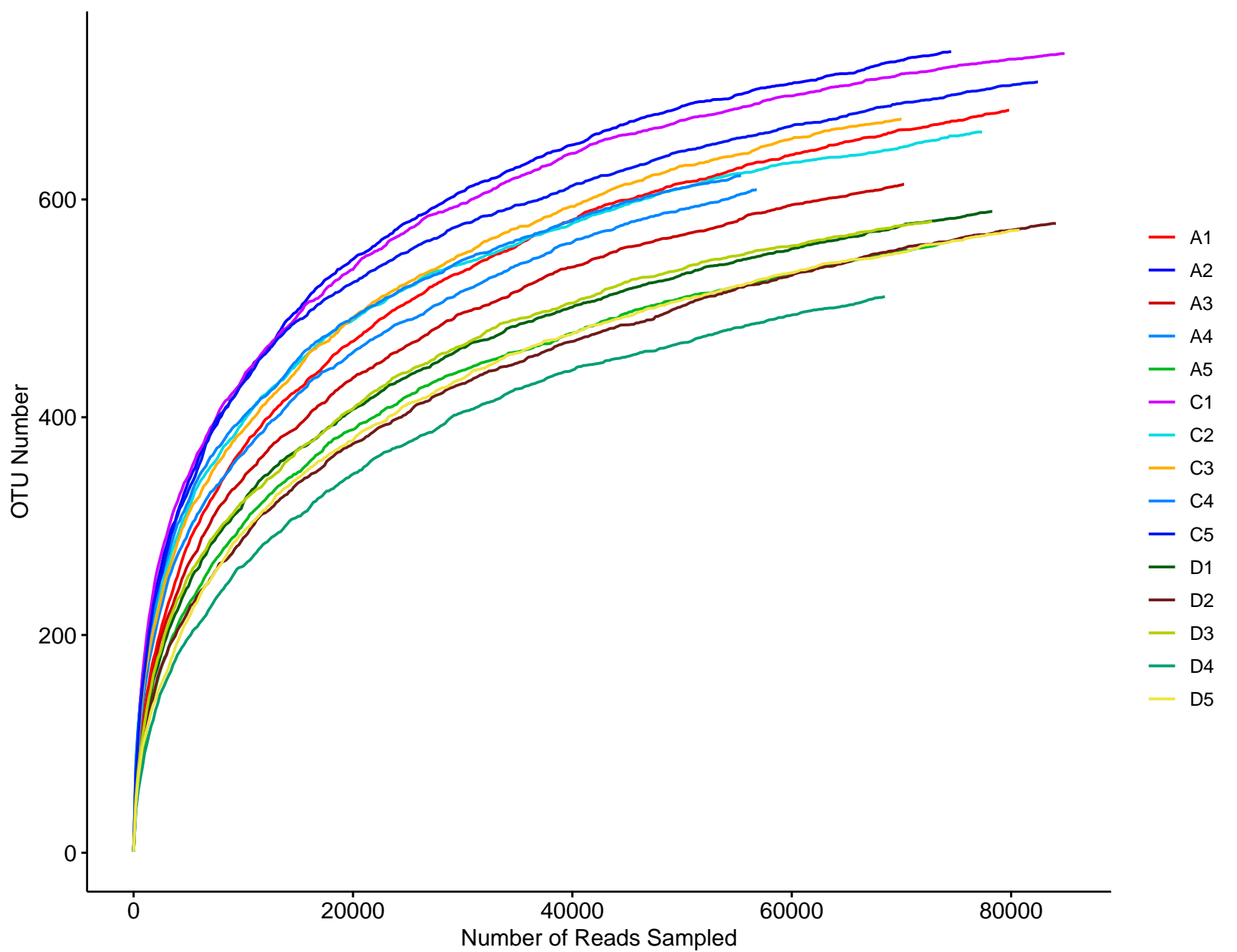

Supplement: S2 File — (ZIP) [file pone.0312147.s002.zip › 3_AlphaDiversity/Rarefaction/otu_rarefaction.pdf]

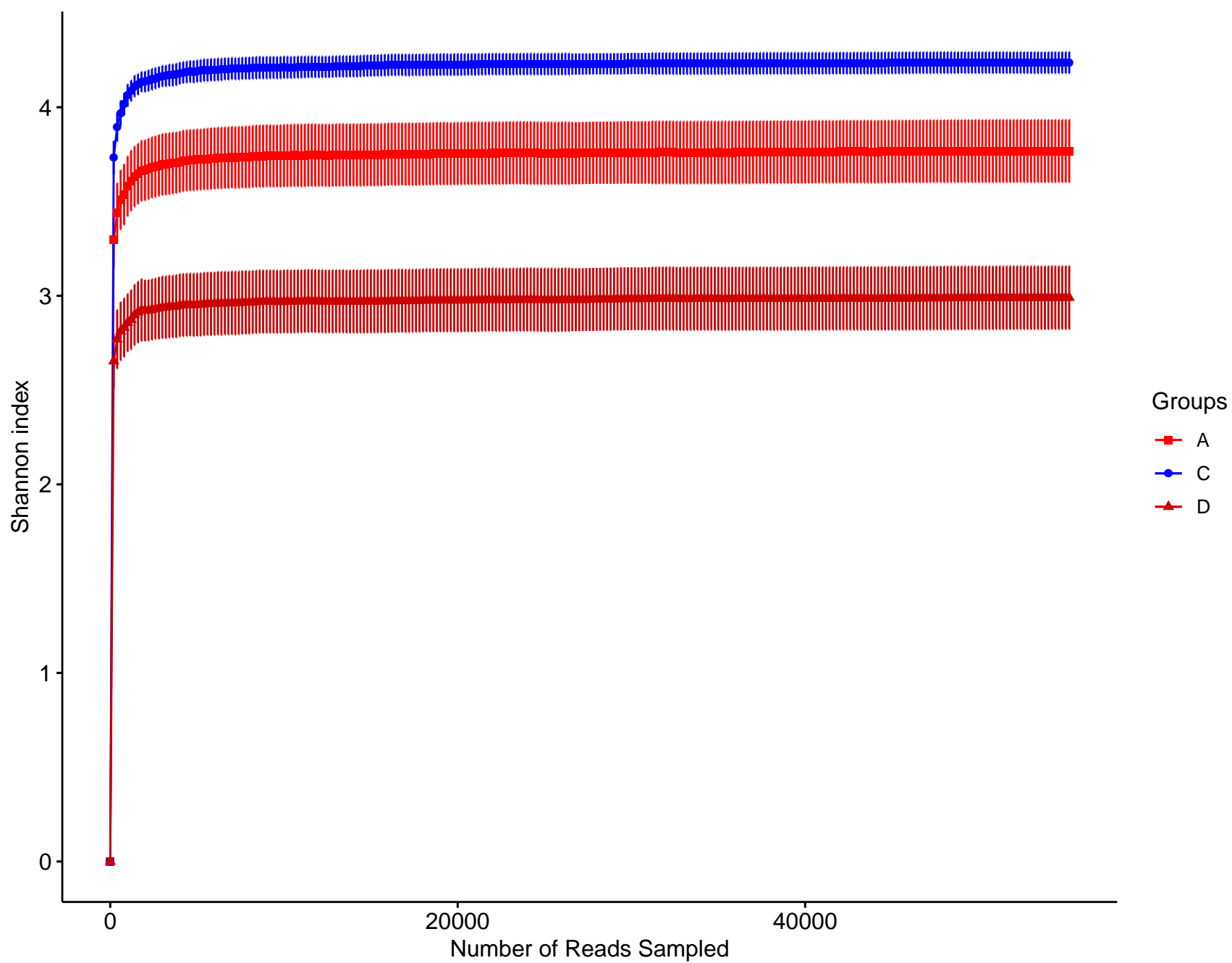

Supplement: S2 File — (ZIP) [file pone.0312147.s002.zip › 3_AlphaDiversity/Rarefaction/shannon_rarefaction.groups.pdf]

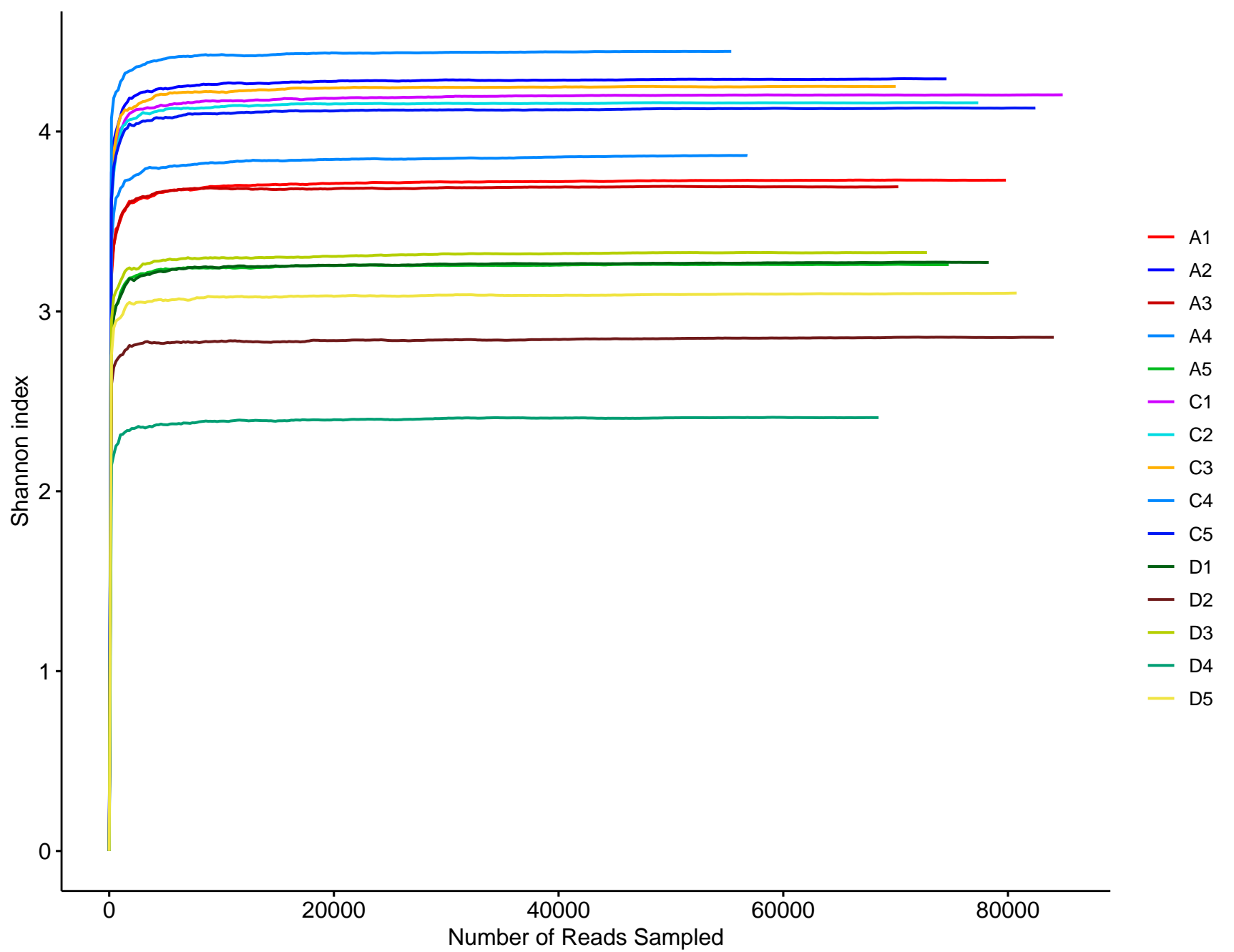

Supplement: S2 File — (ZIP) [file pone.0312147.s002.zip › 3_AlphaDiversity/Rarefaction/shannon_rarefaction.pdf]

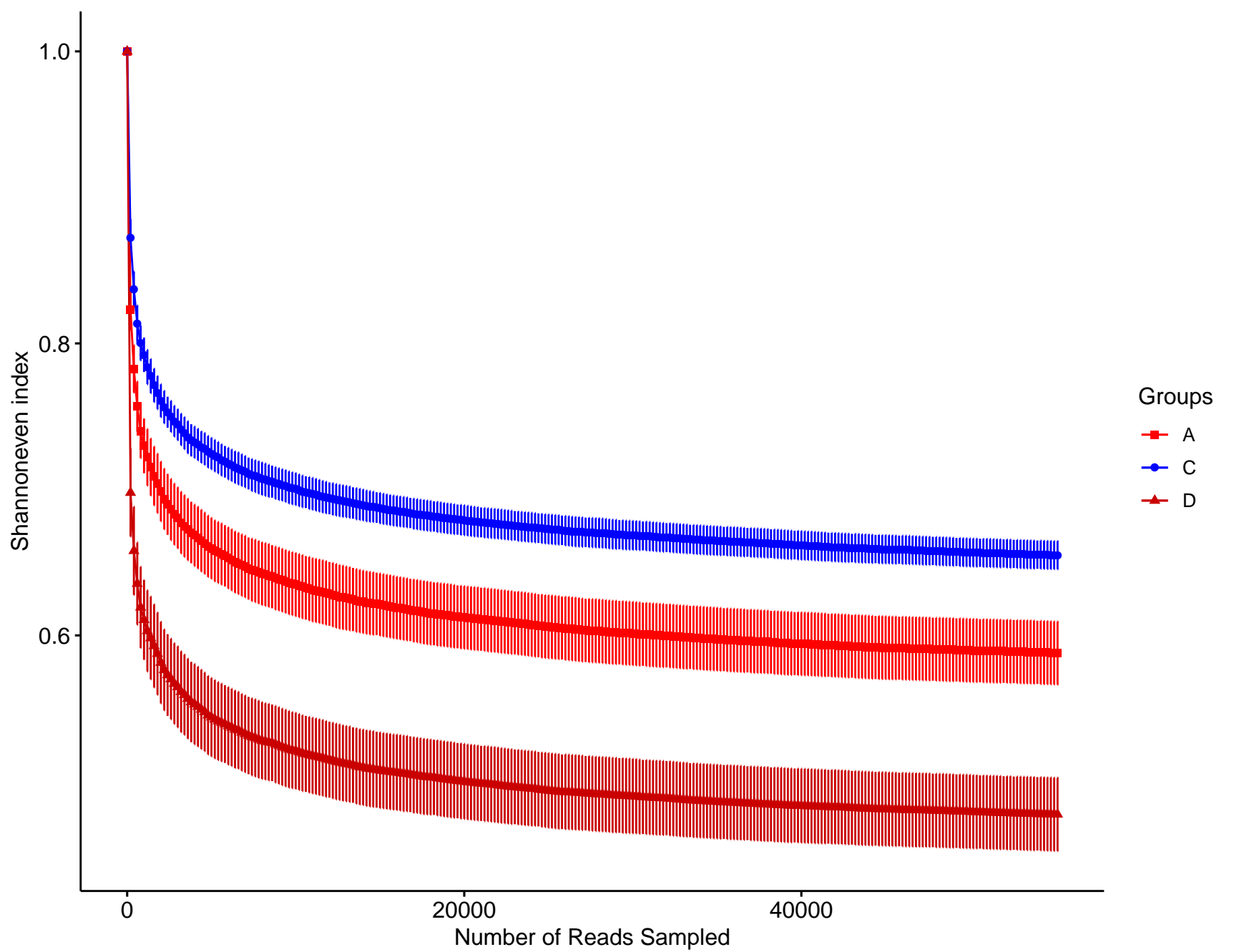

Supplement: S2 File — (ZIP) [file pone.0312147.s002.zip › 3_AlphaDiversity/Rarefaction/shannoneven_rarefaction.groups.pdf]

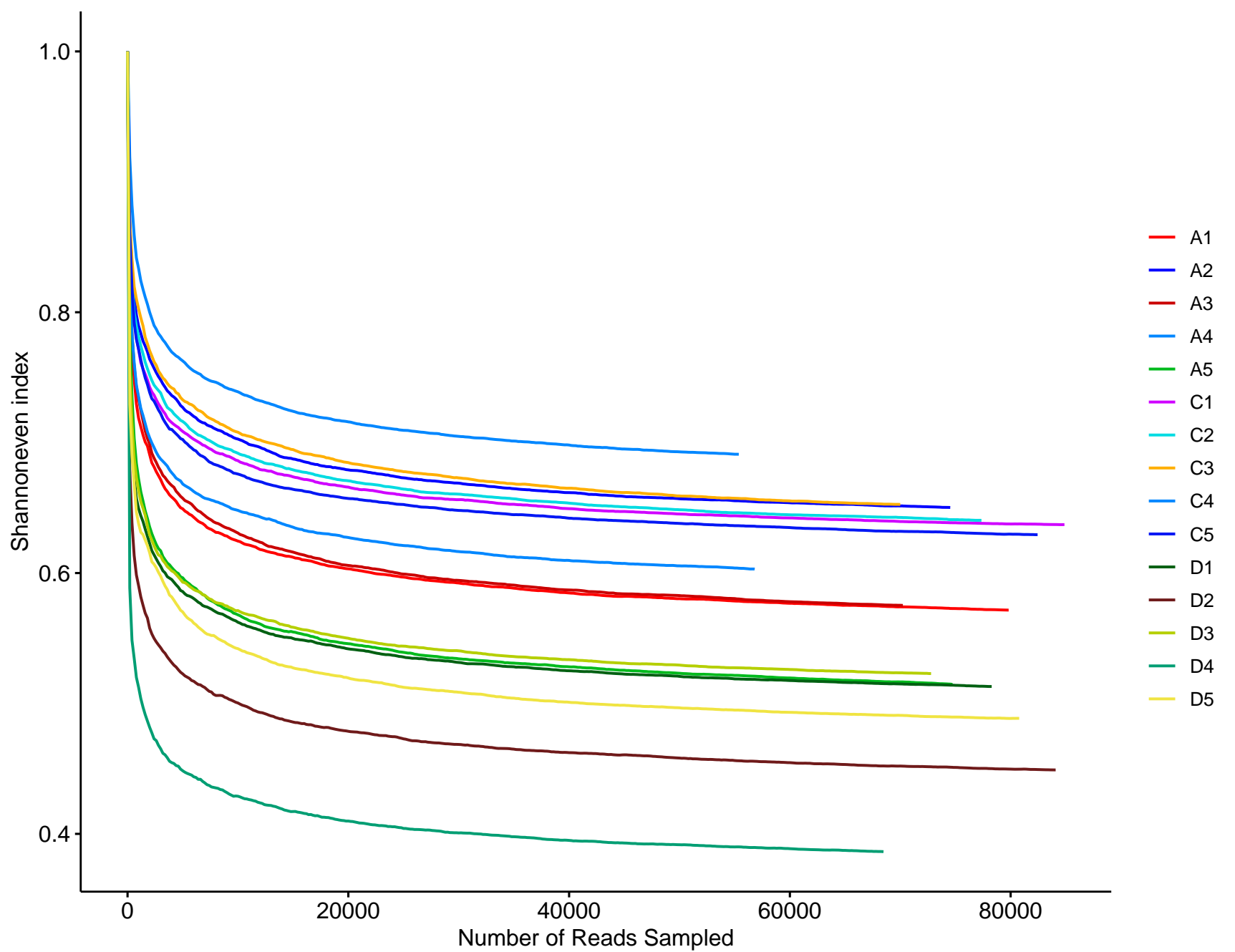

Supplement: S2 File — (ZIP) [file pone.0312147.s002.zip › 3_AlphaDiversity/Rarefaction/shannoneven_rarefaction.pdf]

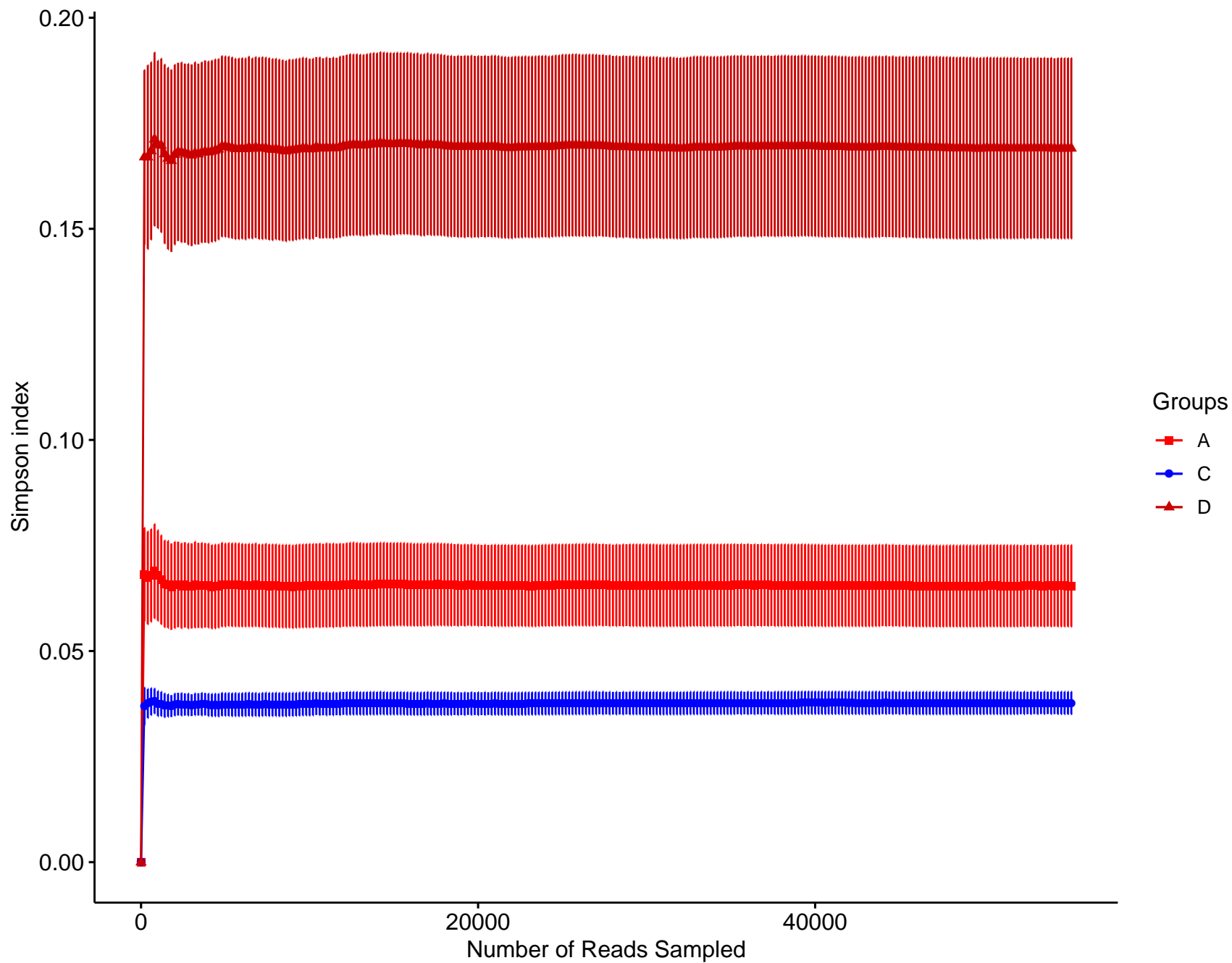

Supplement: S2 File — (ZIP) [file pone.0312147.s002.zip › 3_AlphaDiversity/Rarefaction/simpson_rarefaction.groups.pdf]

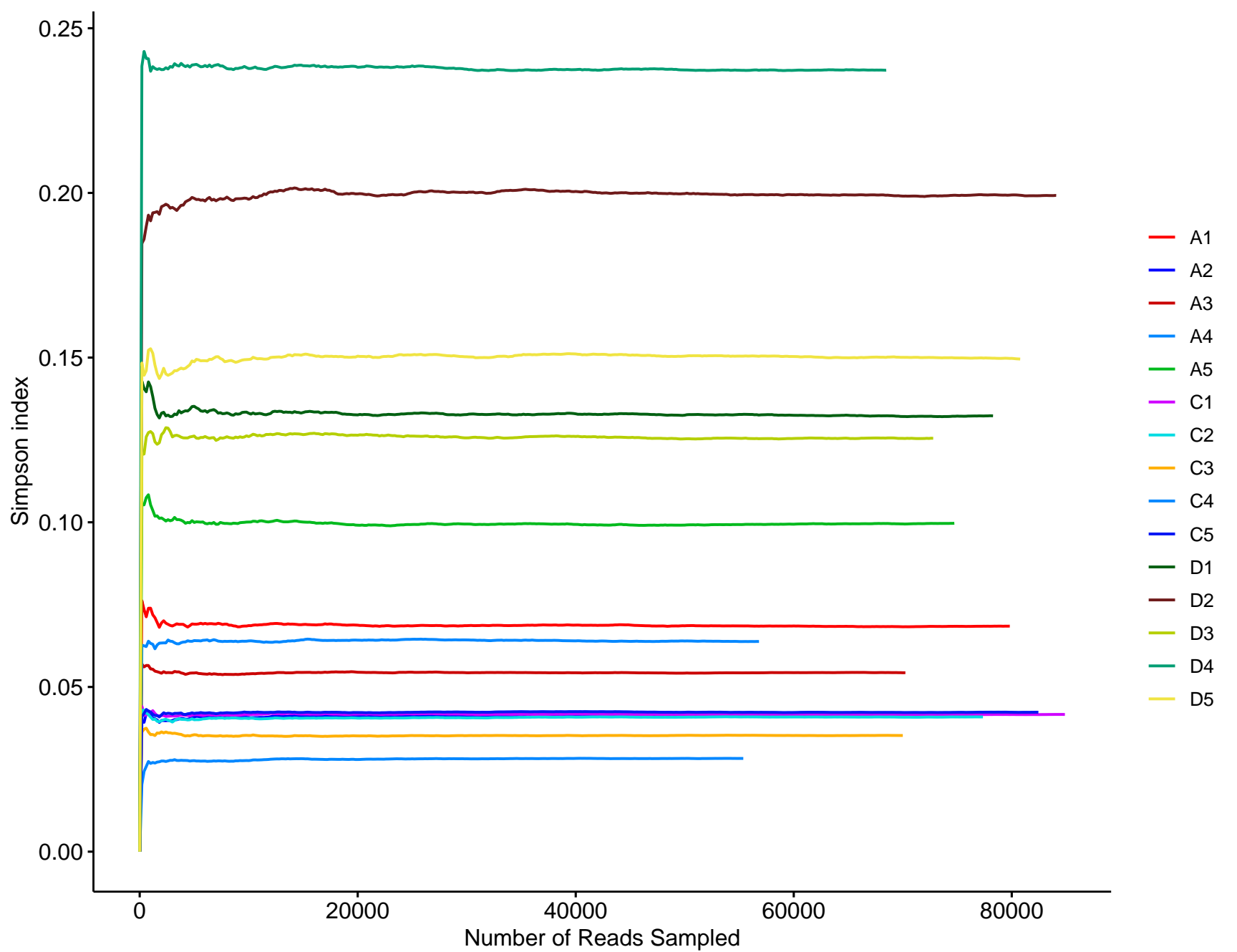

Supplement: S2 File — (ZIP) [file pone.0312147.s002.zip › 3_AlphaDiversity/Rarefaction/simpson_rarefaction.pdf]

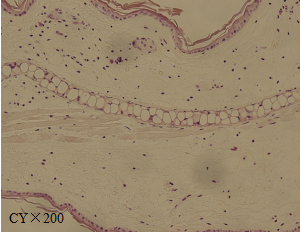

Supplement: S3 File — (ZIP) [file pone.0312147.s003.zip › Fig 1F/CY.png]

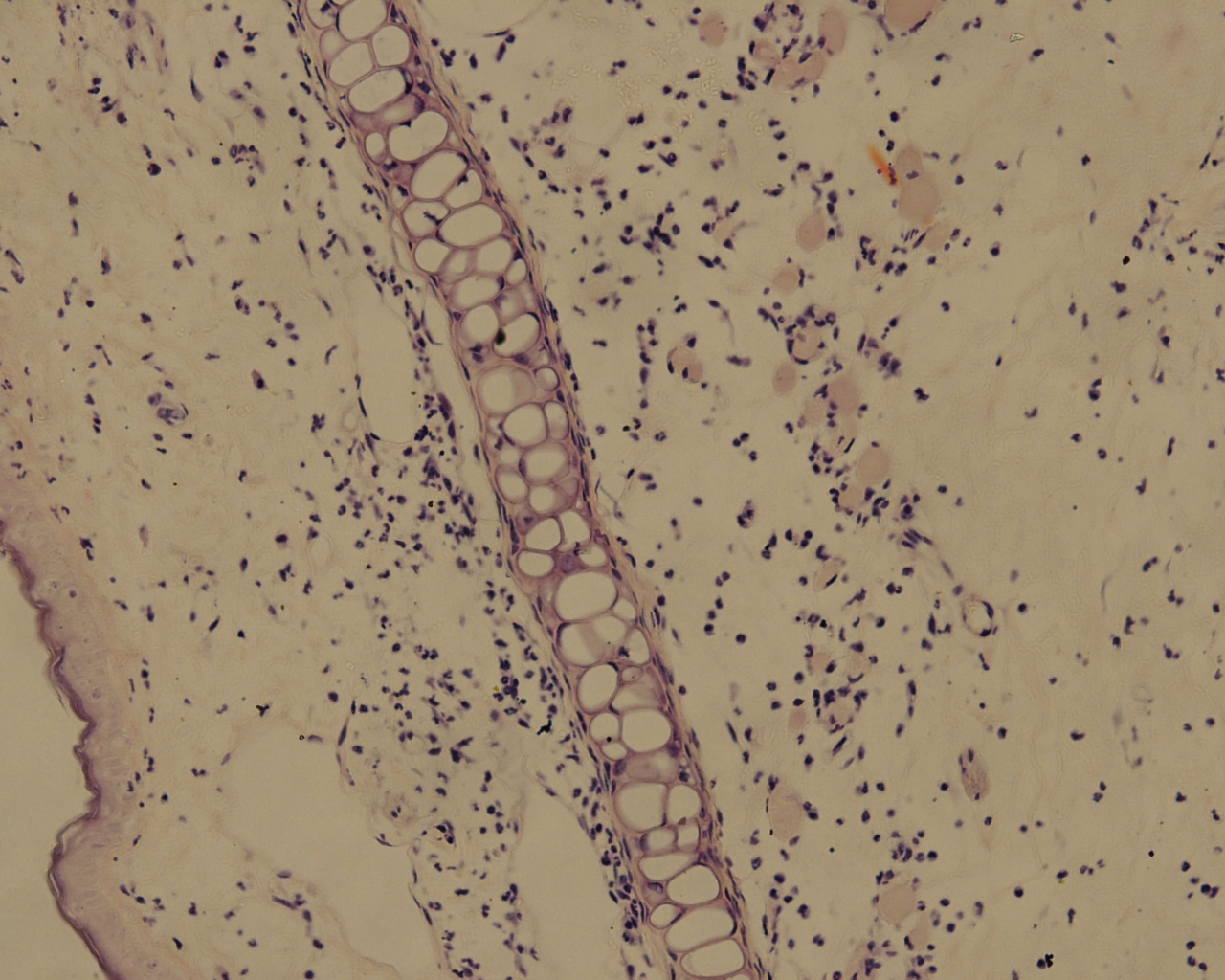

Supplement: S3 File — (ZIP) [file pone.0312147.s003.zip › Fig 1F/Control.png]

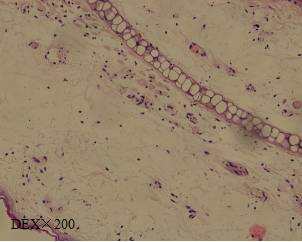

Supplement: S3 File — (ZIP) [file pone.0312147.s003.zip › Fig 1F/DEX.png]

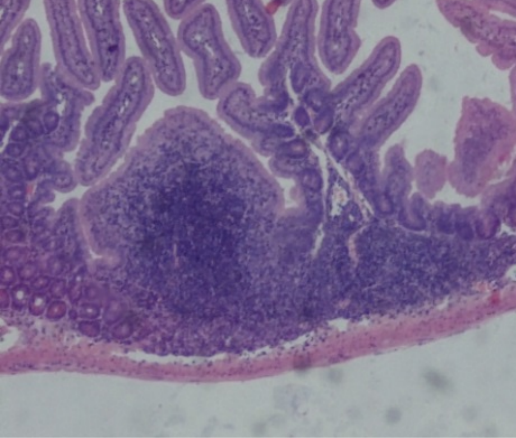

Supplement: S4 File — (ZIP) [file pone.0312147.s004.zip › Fig 1H/CY.png]

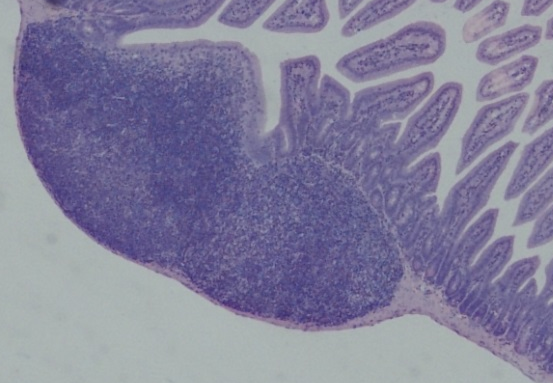

Supplement: S4 File — (ZIP) [file pone.0312147.s004.zip › Fig 1H/Control.png]

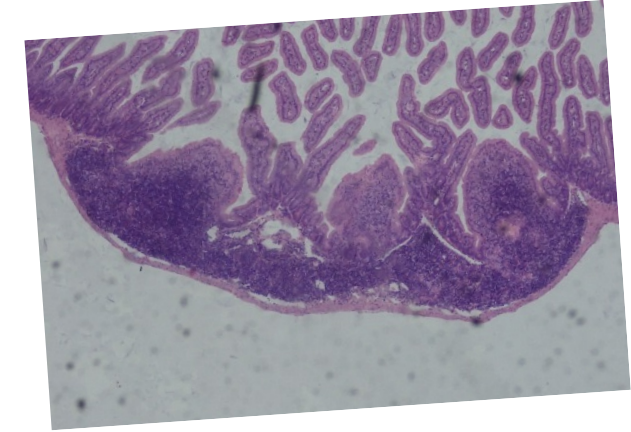

Supplement: S4 File — (ZIP) [file pone.0312147.s004.zip › Fig 1H/DEX.png]

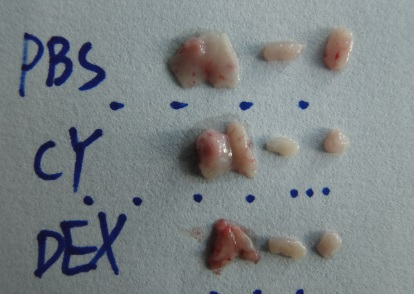

Supplement: S4 File — (ZIP) [file pone.0312147.s004.zip › Fig 1H/Fig 1C.png]

FACSDiva Version 6.2

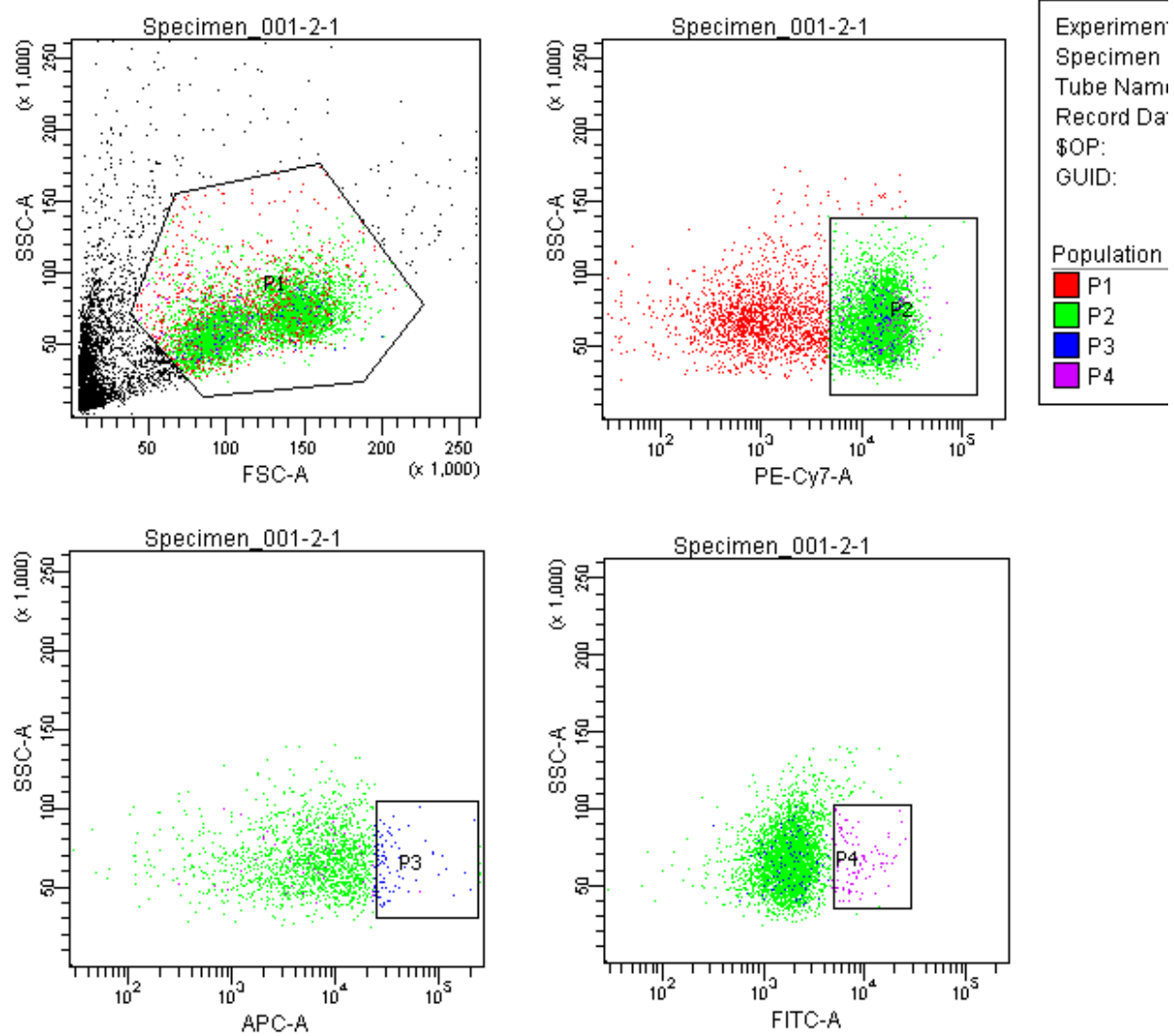

Supplement: S5 File — (ZIP) [file pone.0312147.s005.zip › Flow Cytometric Assessment/Global Sheet1_12052022165036.pdf]

FACSDiva Version 6.2

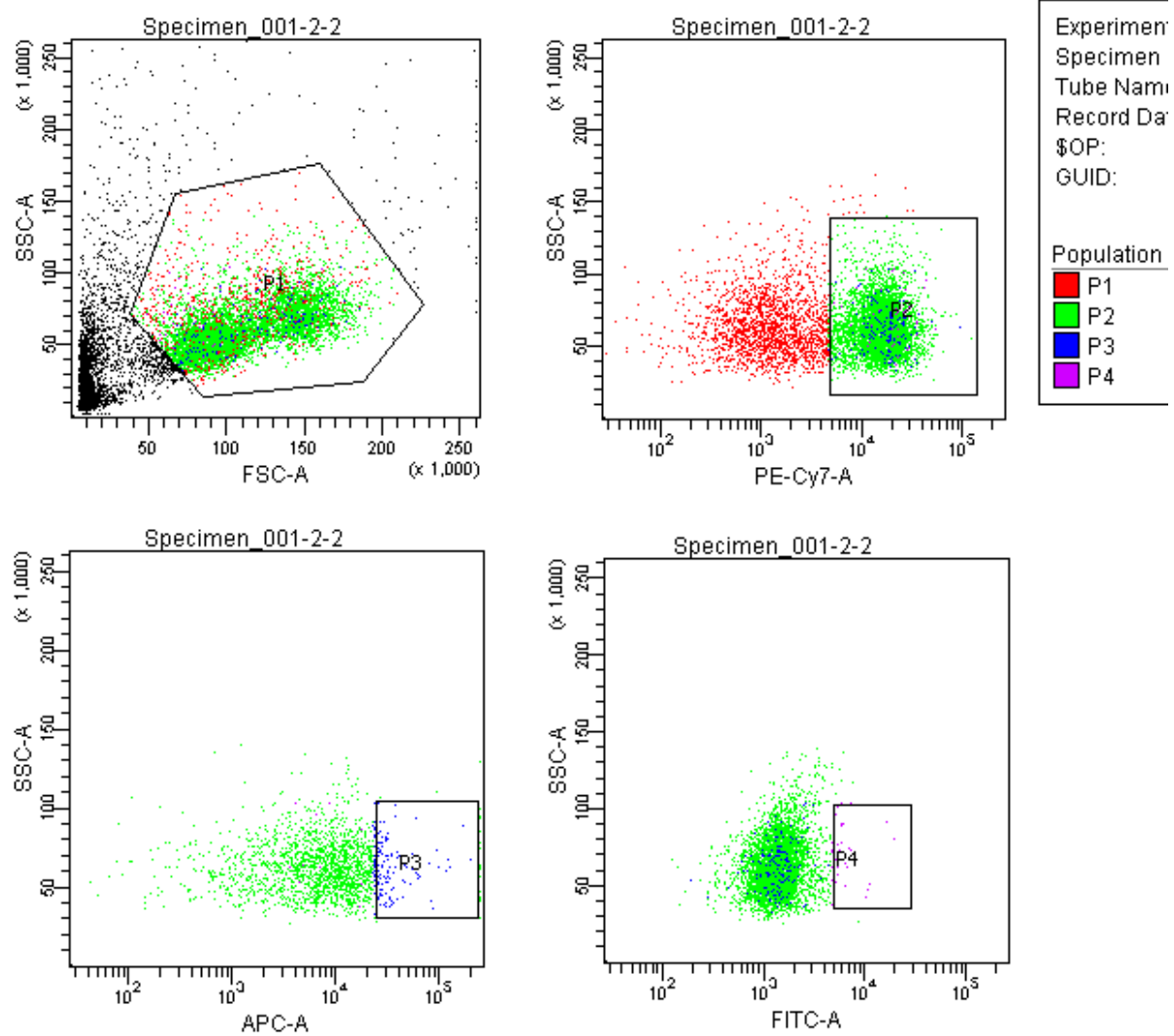

Supplement: S5 File — (ZIP) [file pone.0312147.s005.zip › Flow Cytometric Assessment/Global Sheet1_12052022165045.pdf]

FACSDiva Version 6.2

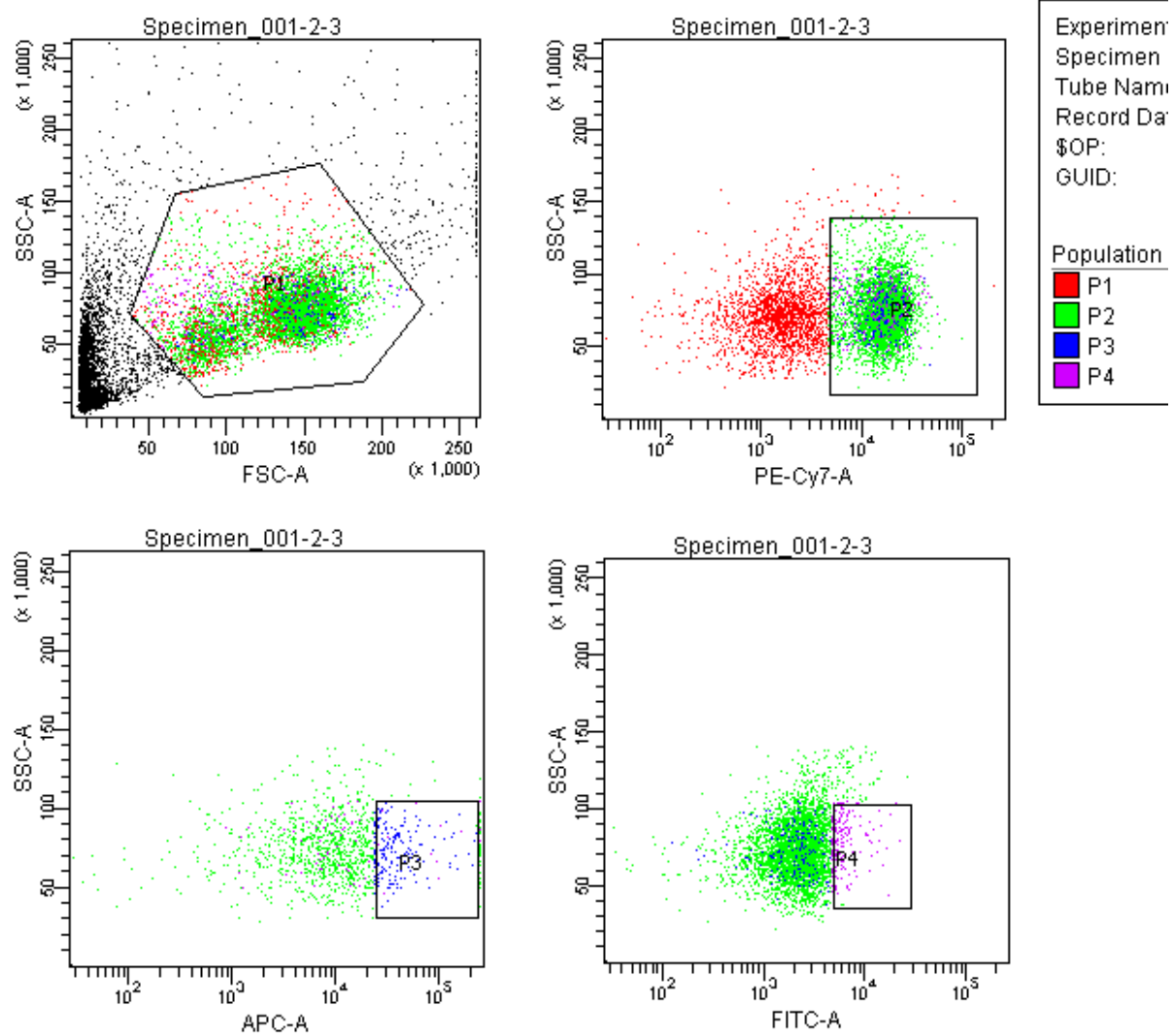

Supplement: S5 File — (ZIP) [file pone.0312147.s005.zip › Flow Cytometric Assessment/Global Sheet1_12052022165054.pdf]

FACSDiva Version 6.2

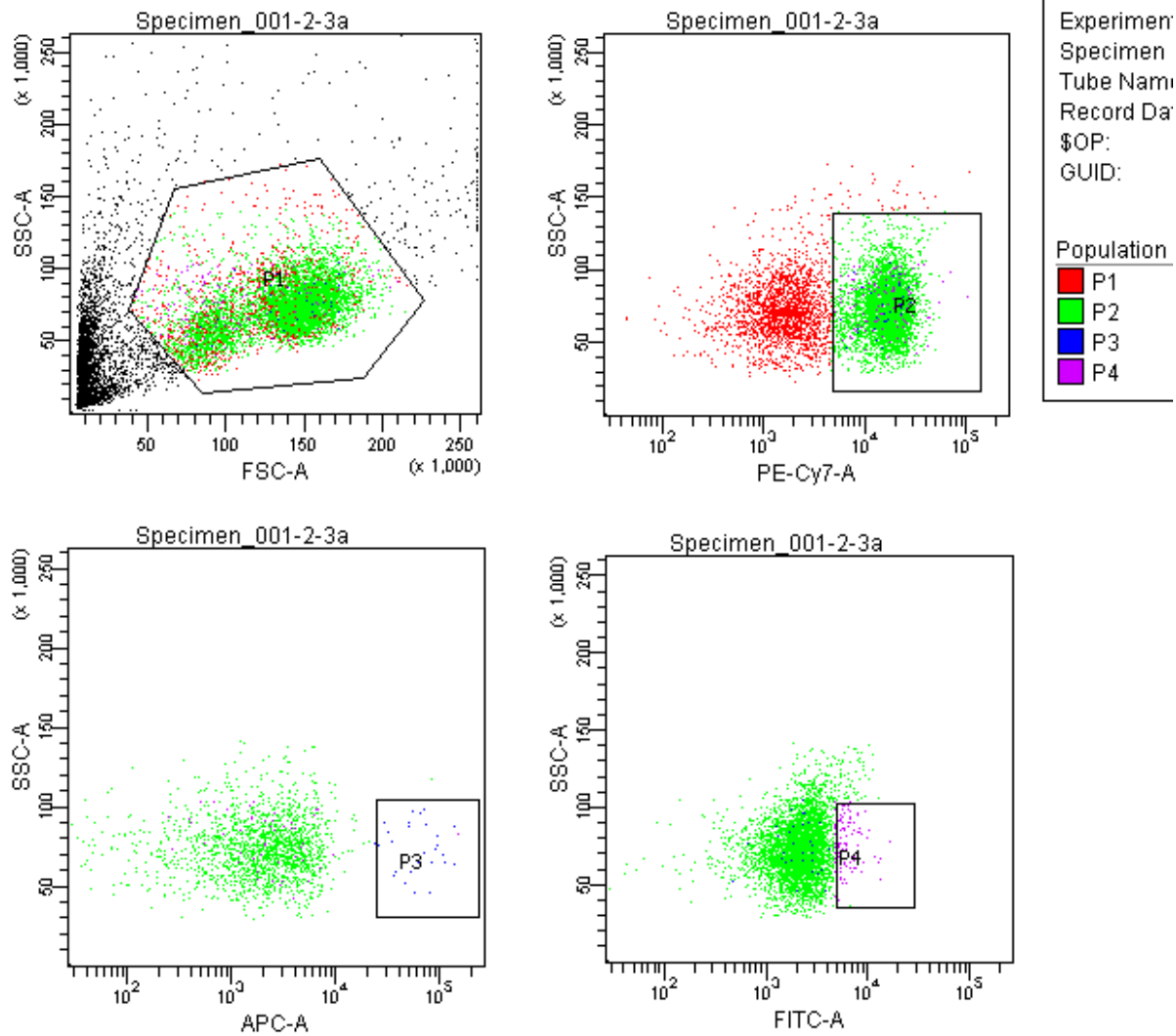

Supplement: S5 File — (ZIP) [file pone.0312147.s005.zip › Flow Cytometric Assessment/Global Sheet1_12052022165106.pdf]

FACSDiva Version 6.2

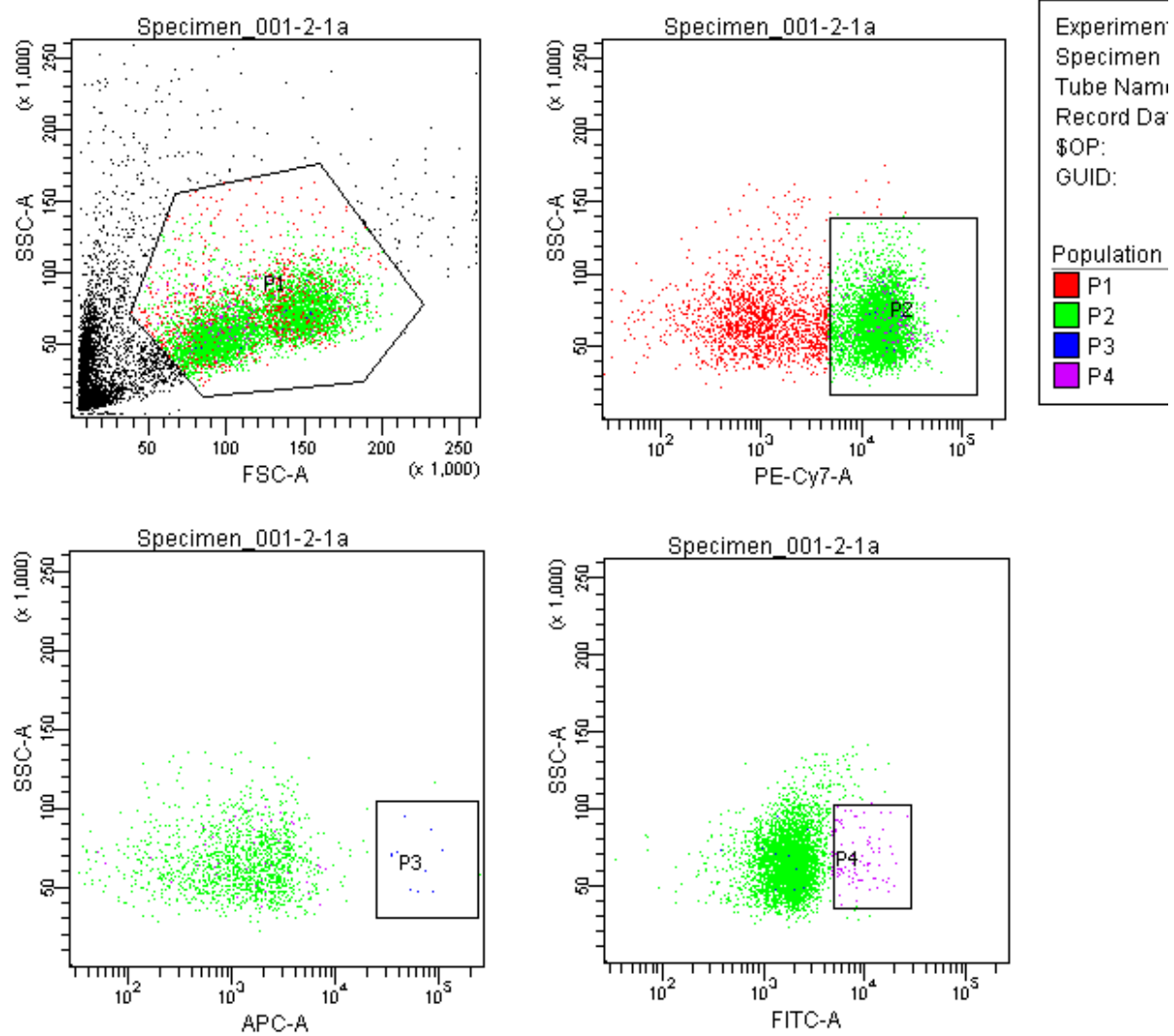

Supplement: S5 File — (ZIP) [file pone.0312147.s005.zip › Flow Cytometric Assessment/Global Sheet1_12052022165111.pdf]

FACSDiva Version 6.2

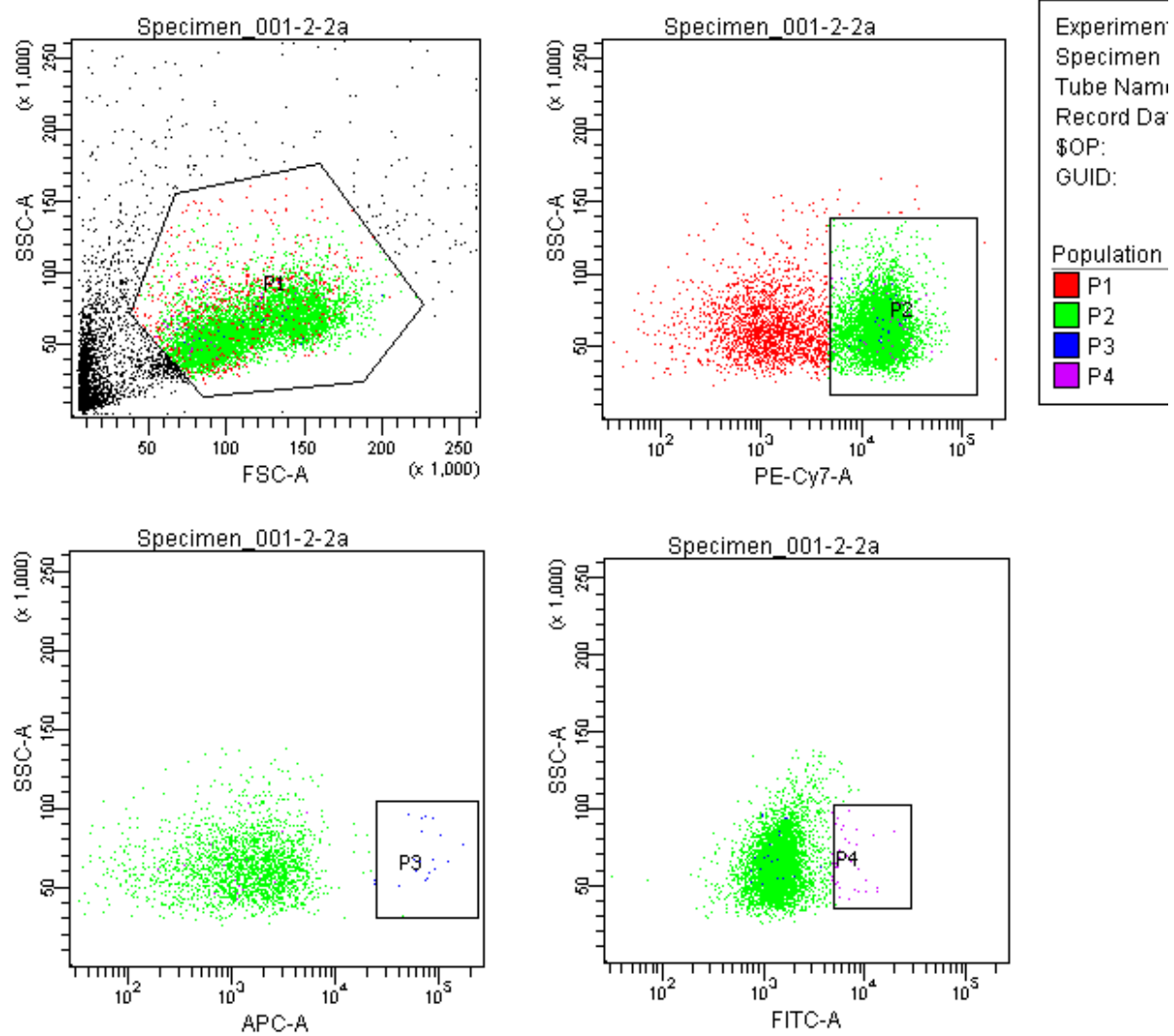

Supplement: S5 File — (ZIP) [file pone.0312147.s005.zip › Flow Cytometric Assessment/Global Sheet1_12052022165115.pdf]

FACSDiva Version 6.2

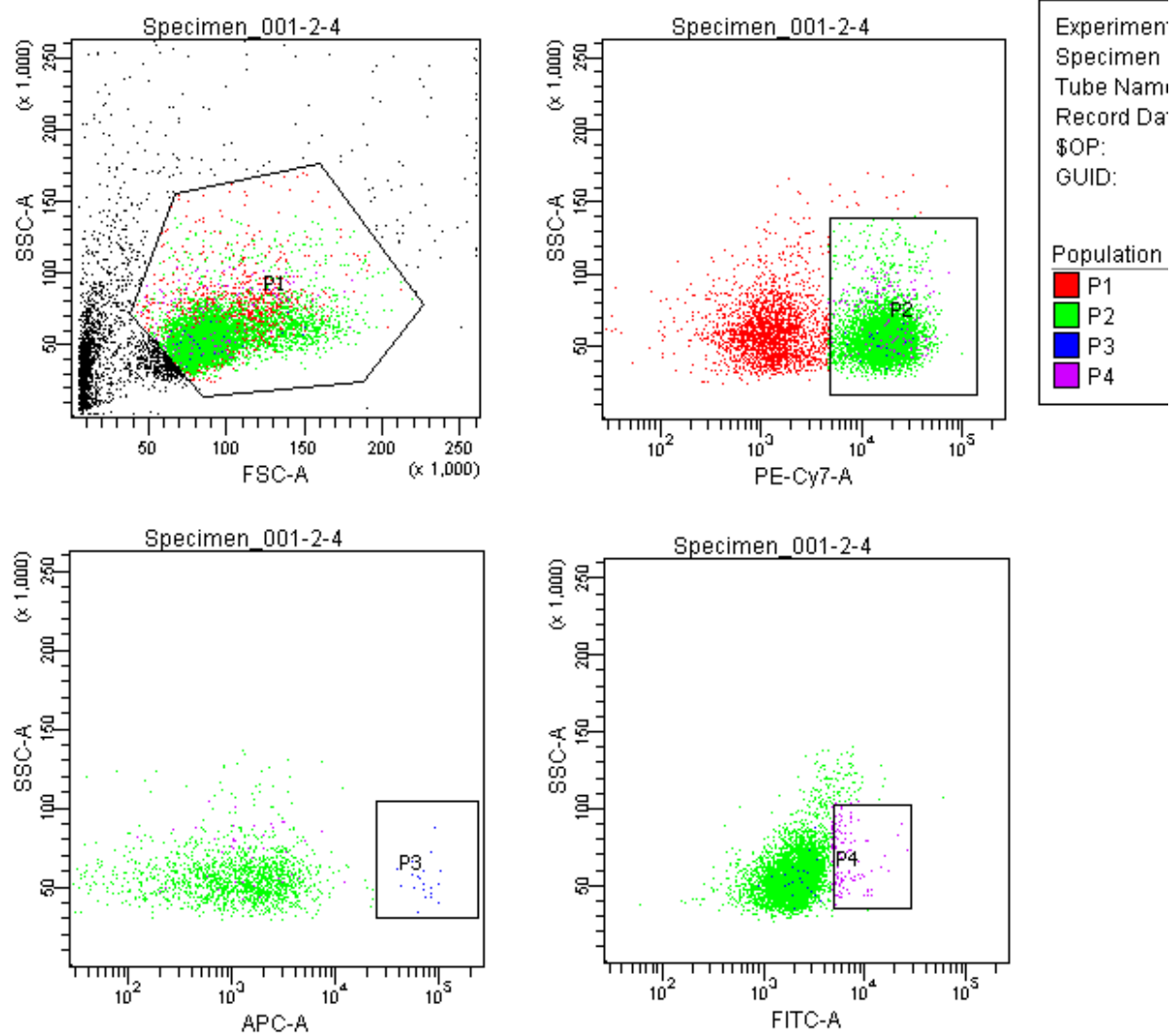

Supplement: S5 File — (ZIP) [file pone.0312147.s005.zip › Flow Cytometric Assessment/Global Sheet1_12052022165121.pdf]

FACSDiva Version 6.2

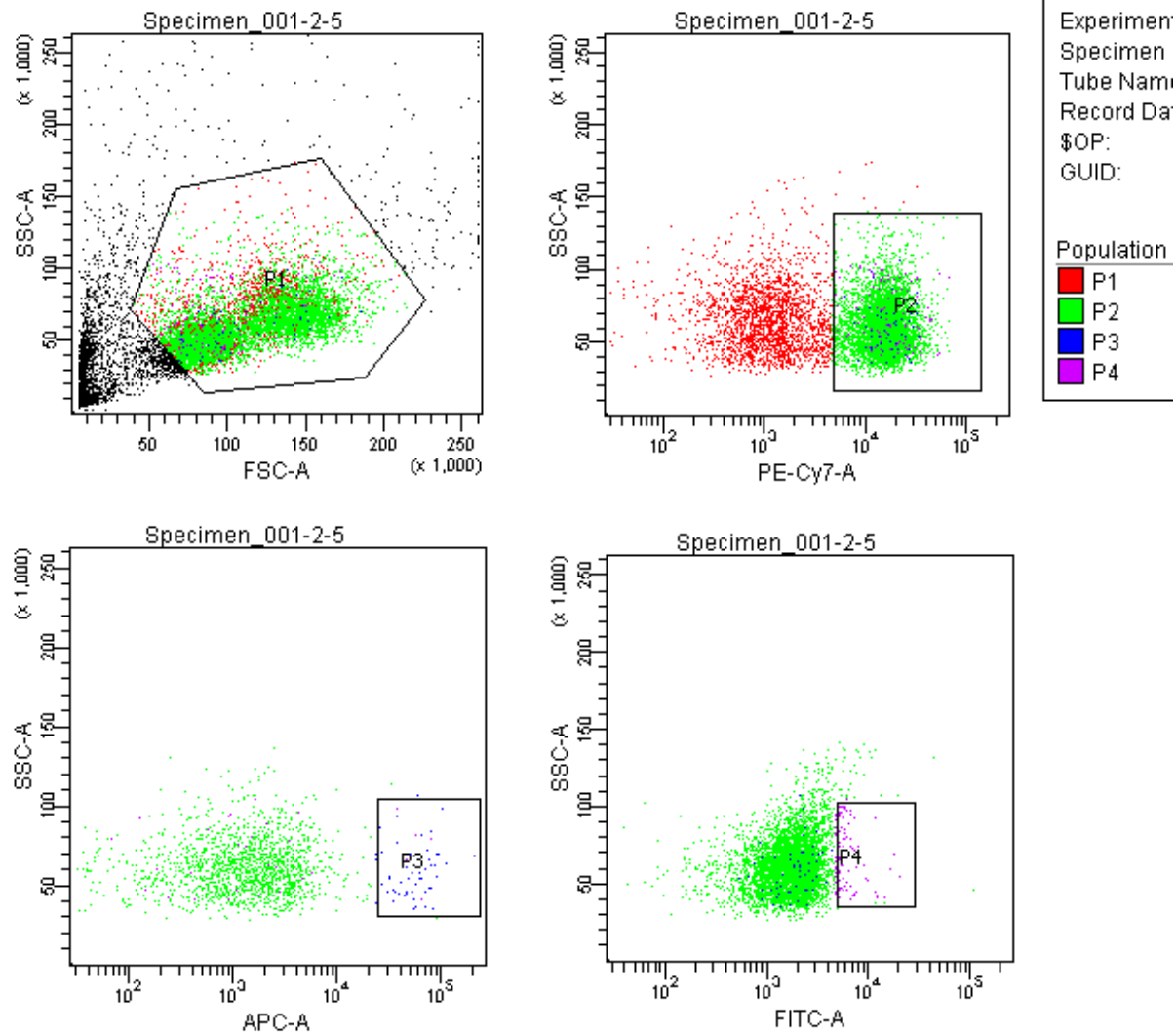

Supplement: S5 File — (ZIP) [file pone.0312147.s005.zip › Flow Cytometric Assessment/Global Sheet1_12052022165127.pdf]

FACSDiva Version 6.2

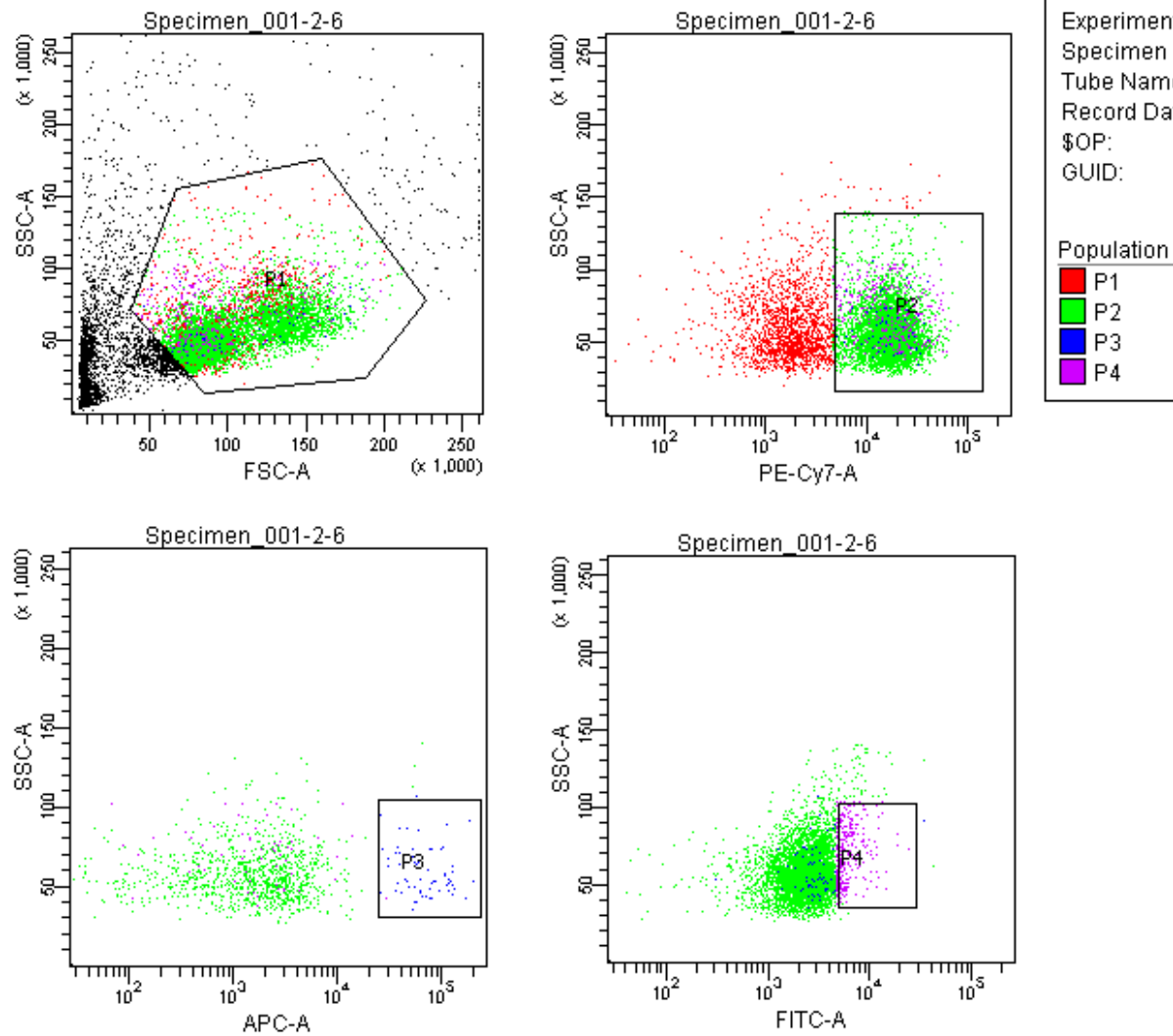

Supplement: S5 File — (ZIP) [file pone.0312147.s005.zip › Flow Cytometric Assessment/Global Sheet1_12052022165133.pdf]

FACSDiva Version 6.2

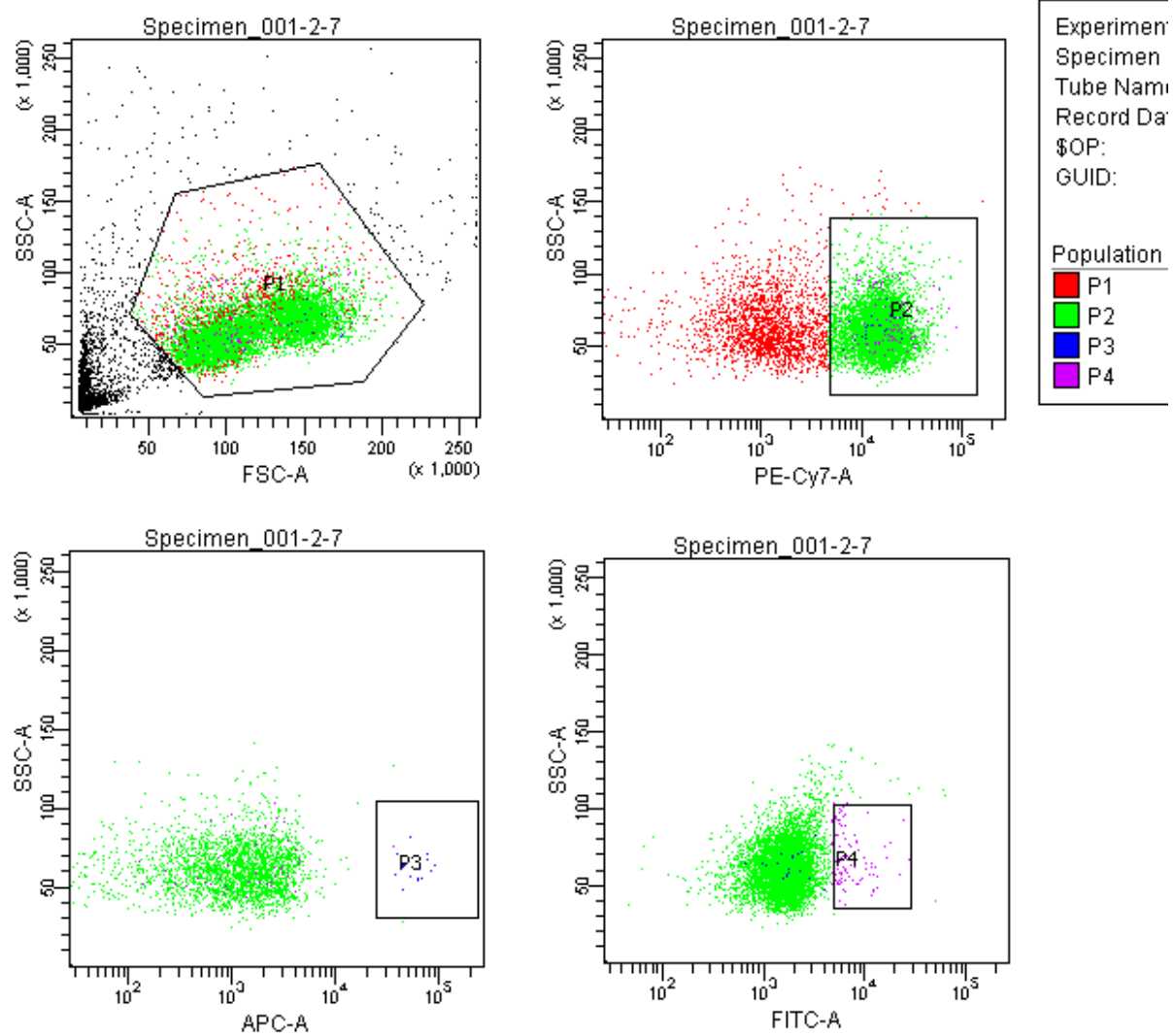

Supplement: S5 File — (ZIP) [file pone.0312147.s005.zip › Flow Cytometric Assessment/Global Sheet1_12052022165139.pdf]
