# Supplementary material for: Comparative analysis of the effects of cyclophosphamide and dexamethasone on intestinal immunity and microbiota in delayed hypersensitivity mice
Source: PLoS One. 2024 Oct 17;19(10):e0312147. doi: 10.1371/journal.pone.0312147 (PMC11486373; doi:10.1371/journal.pone.0312147)

FACSDiva Version 6.2

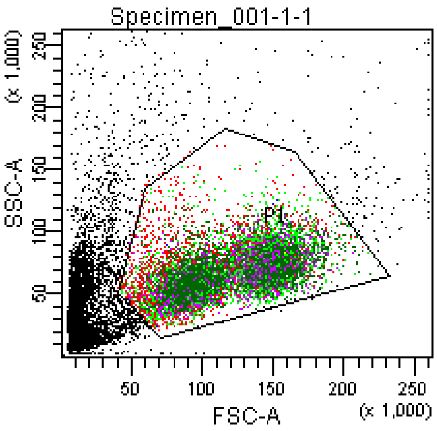

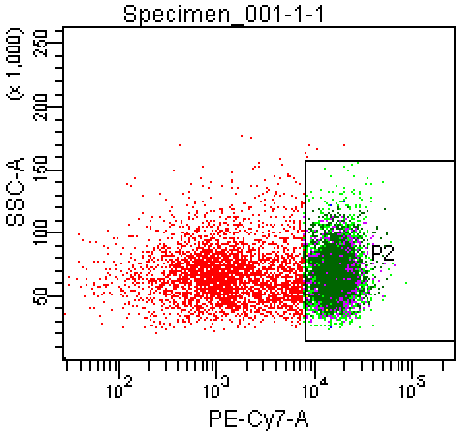

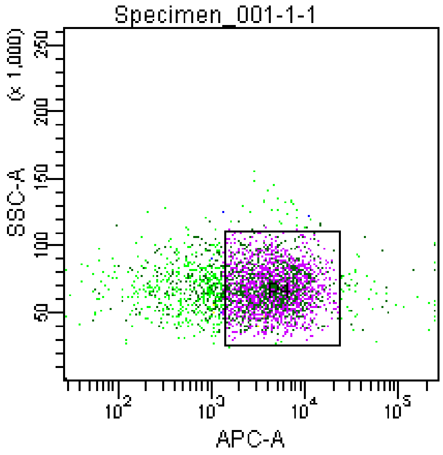

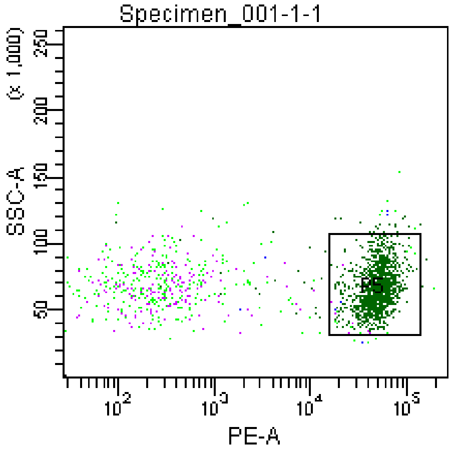

Experiment Name: Experiment_7740
Specimen Name: Specimen_001
Tube Name: 1-1
Record Date: Jan 10, 2022 8:31:01 PM
$OP: Administrator
GUID: 0c35e18f-3d2d-4cf1-8131-554d54c67593

Population	#Events	%Parent	SSC-A Mean	PE-Cy7-A Mean
P1	11,017	55.1	66,217	12,327
P2	7,120	64.6	66,409	18,006
P3	463	6.5	63,865	16,223
P5	439	94.8	63,839	16,239
P4	2,304	32.4	65,478	17,854
P6	2,055	28.9	67,521	17,452

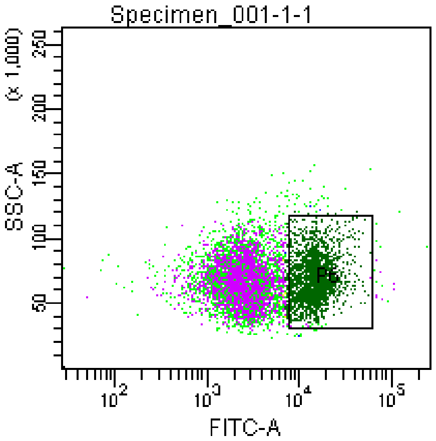

Supplement: S5 File — (ZIP) [file pone.0312147.s005.zip › Flow Cytometric Assessment/Global Sheet1_12052022164706.pdf]
