# Supplementary material for: Comparative analysis of the effects of cyclophosphamide and dexamethasone on intestinal immunity and microbiota in delayed hypersensitivity mice
Source: PLoS One. 2024 Oct 17;19(10):e0312147. doi: 10.1371/journal.pone.0312147 (PMC11486373; doi:10.1371/journal.pone.0312147)

FACSDiva Version 6.2

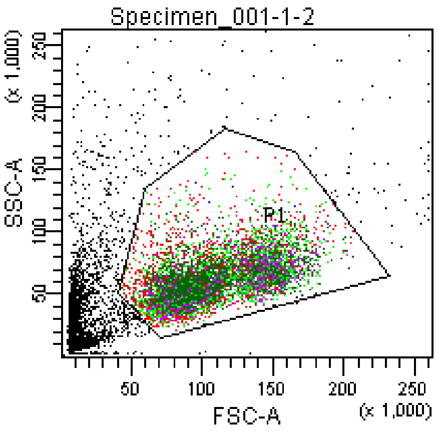

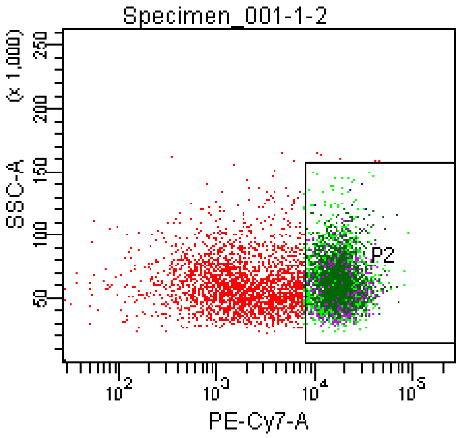

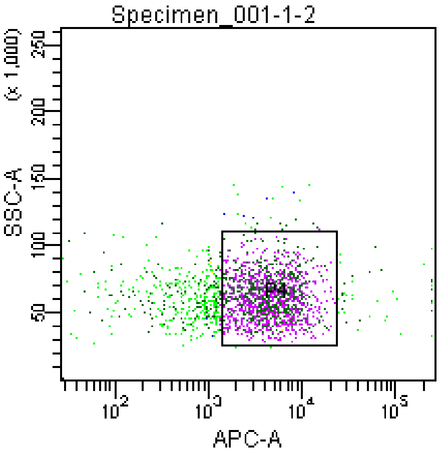

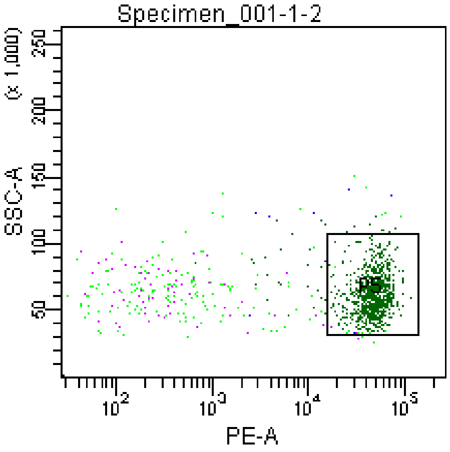

Experiment Name:	Experiment_7740
Specimen Name:	Specimen_001
Tube Name:	1-2
Record Date:	Jan 10, 2022 8:32:16 PM
$OP:	Administrator
GUID:	cfd9f243-db97-48a5-a366-1f139e3032f7

Population	#Events	%Parent	SSC-A Mean	PE-Cy7-A Mean
P1	7,000	70.0	60,294	13,521
P2	4,581	65.4	60,339	19,344
P3	285	6.2	60,313	18,480
P5	262	91.9	58,978	18,310
P4	1,388	30.3	59,441	19,460
P6	1,479	32.3	63,124	19,607

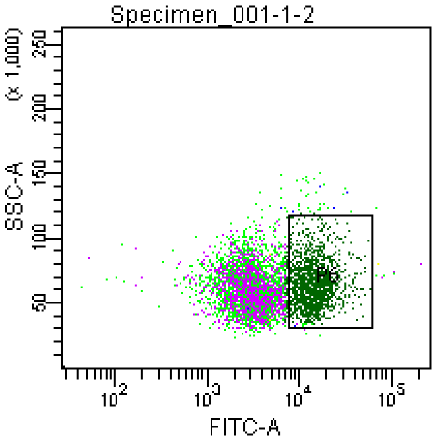

Supplement: S5 File — (ZIP) [file pone.0312147.s005.zip › Flow Cytometric Assessment/Global Sheet1_12052022164814.pdf]
