# Supplementary material for: Comparative analysis of the effects of cyclophosphamide and dexamethasone on intestinal immunity and microbiota in delayed hypersensitivity mice
Source: PLoS One. 2024 Oct 17;19(10):e0312147. doi: 10.1371/journal.pone.0312147 (PMC11486373; doi:10.1371/journal.pone.0312147)

FACSDiva Version 6.2

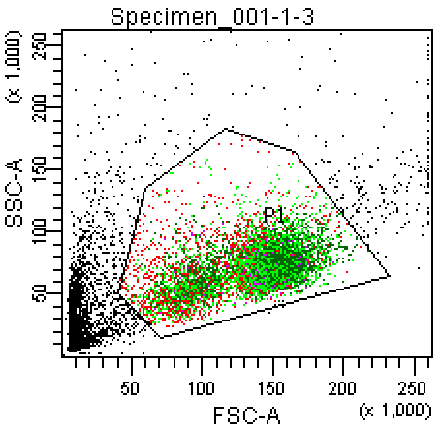

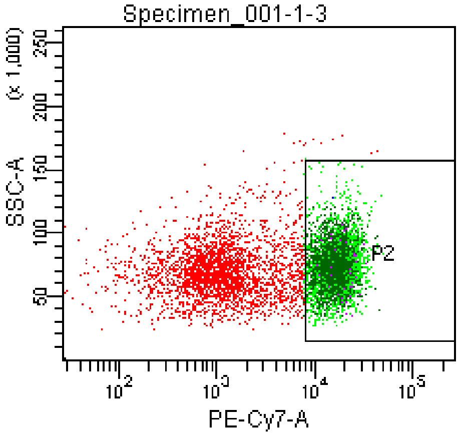

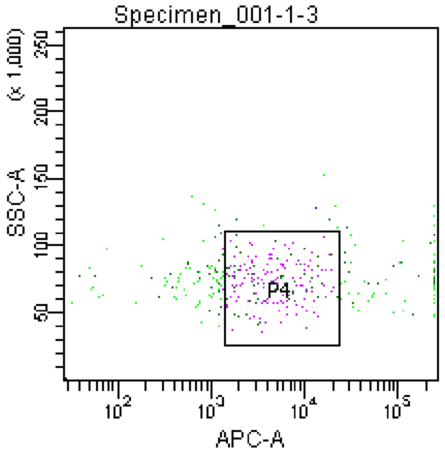

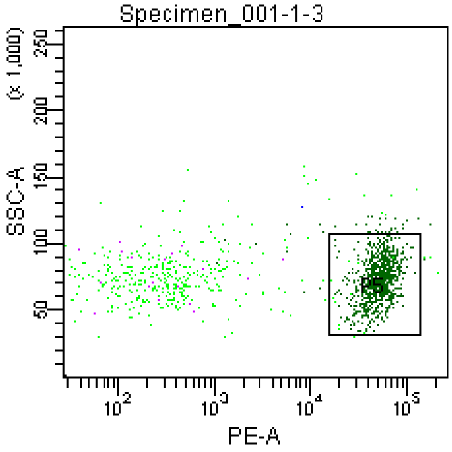

Experiment Name:	Experiment_7740
Specimen Name:	Specimen_001
Tube Name:	1-3
Record Date:	Jan 10, 2022 8:40:07 PM
$OP:	Administrator
GUID:	4cb2e3f6-d2cb-4992-8449-5134f4954bec

Population	#Events	%Parent	SSC-A Mean	PE-Cy7-A Mean
P1	7,082	70.8	70,424	11,804
P2	4,314	60.9	72,073	18,247
P3	53	1.2	75,633	14,798
P5	47	88.7	71,906	14,825
P4	188	4.4	71,567	17,851
P6	1,276	29.6	73,353	16,781

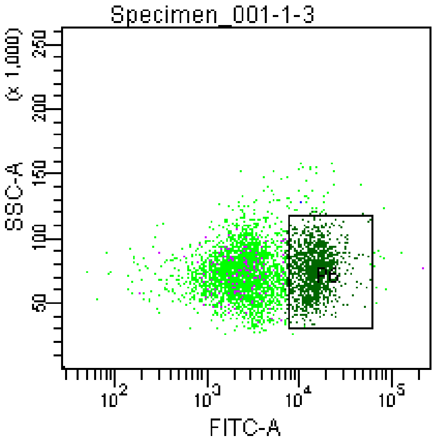

Supplement: S5 File — (ZIP) [file pone.0312147.s005.zip › Flow Cytometric Assessment/Global Sheet1_12052022164821.pdf]
