# Supplementary material for: Comparative analysis of the effects of cyclophosphamide and dexamethasone on intestinal immunity and microbiota in delayed hypersensitivity mice
Source: PLoS One. 2024 Oct 17;19(10):e0312147. doi: 10.1371/journal.pone.0312147 (PMC11486373; doi:10.1371/journal.pone.0312147)

# FACSDiva Version 6.2

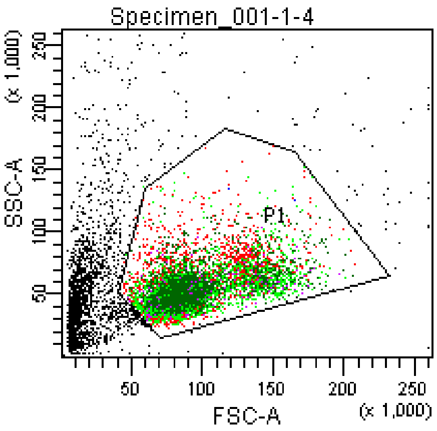


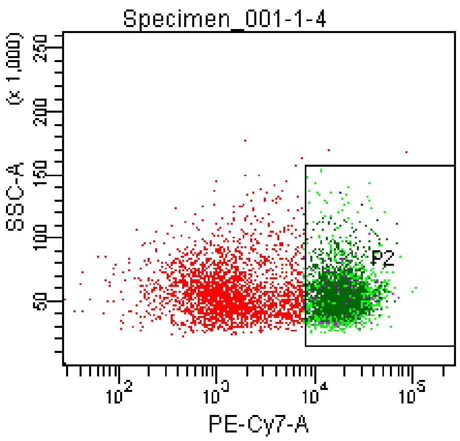


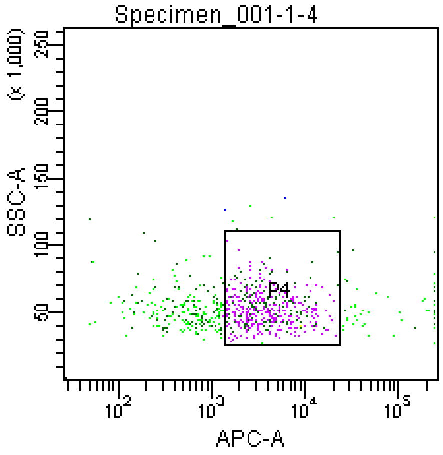


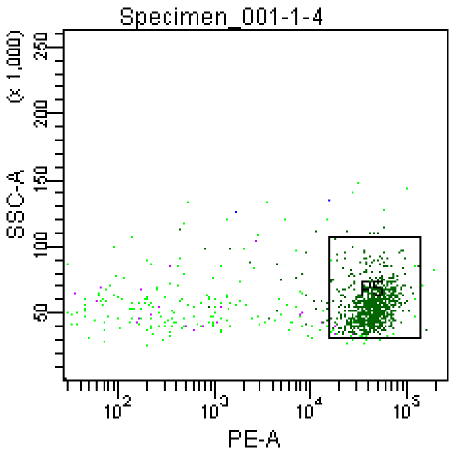


| | |
|---|---|
| Experiment Name: | Experiment_7740 |
| Specimen Name: | Specimen_001 |
| Tube Name: | 1-4 |
| Record Date: | Jan 10, 2022 8:41:58 PM |
| $OP: | Administrator |
| GUID: | 1d204c7c-bab4-477a-9ba4-c6985a74fc55 |

| Population | #Events | %Parent | SSC-A Mean | PE-Cy7-A Mean |
|---|---|---|---|---|
| P1 | 7,331 | 73.3 | 53,500 | 13,776 |
| P2 | 4,685 | 63.9 | 52,057 | 20,383 |
| P3 | 91 | 1.9 | 54,517 | 19,614 |
| P5 | 81 | 89.0 | 52,827 | 19,498 |
| P4 | 447 | 9.5 | 51,324 | 20,597 |
| P6 | 1,515 | 32.3 | 55,521 | 19,751 |

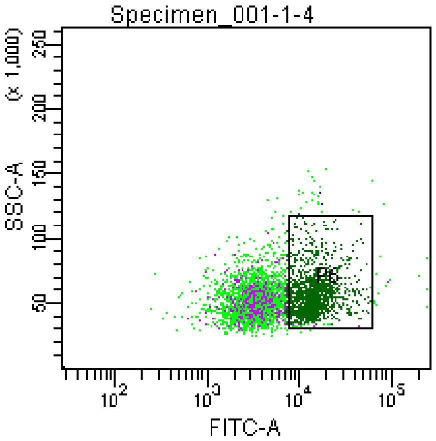

Supplement: S5 File — (ZIP) [file pone.0312147.s005.zip › Flow Cytometric Assessment/Global Sheet1_12052022164829.pdf]
