# Supplementary material for: Comparative analysis of the effects of cyclophosphamide and dexamethasone on intestinal immunity and microbiota in delayed hypersensitivity mice
Source: PLoS One. 2024 Oct 17;19(10):e0312147. doi: 10.1371/journal.pone.0312147 (PMC11486373; doi:10.1371/journal.pone.0312147)

FACSDiva Version 6.2

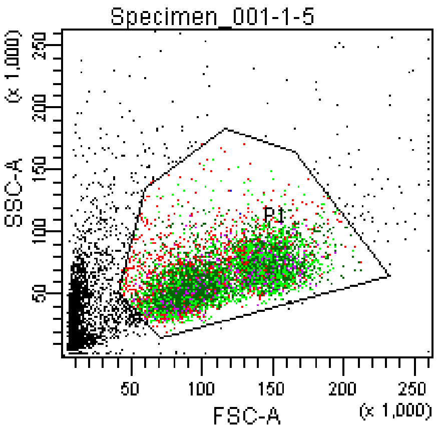

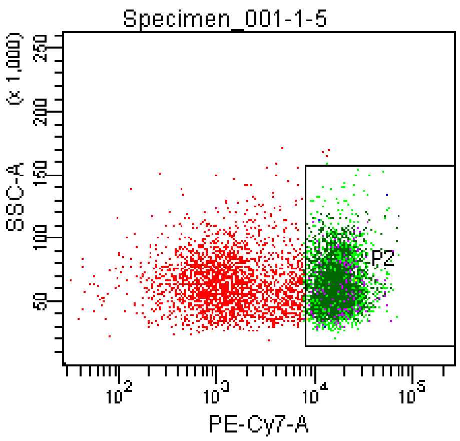

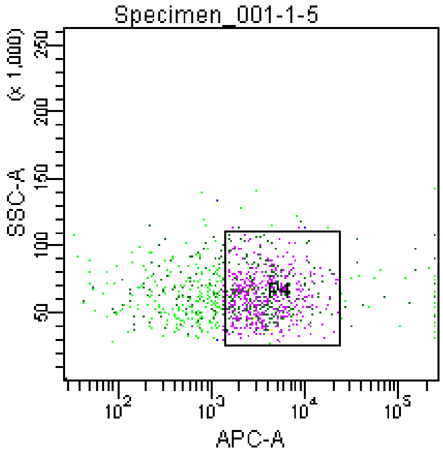

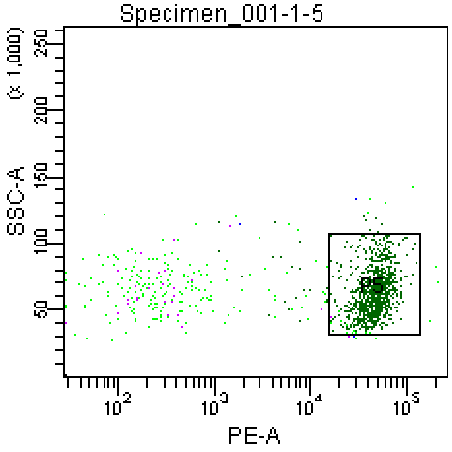

Experiment Name: Experiment_7740
Specimen Name: Specimen_001
Tube Name: 1-5
Record Date: Jan 10, 2022 8:42:57 PM
$OP: Administrator
GUID: a47b59cc-d73b-46d7-a50c-8e4383e22541

Population	#Events	%Parent	SSC-A Mean	PE-Cy7-A Mean
P1	7,267	72.7	62,281	13,127
P2	4,764	65.6	61,954	18,988
P3	176	3.7	61,564	17,425
P5	168	95.5	61,500	17,259
P4	684	14.4	61,128	19,207
P6	1,483	31.1	64,035	18,468

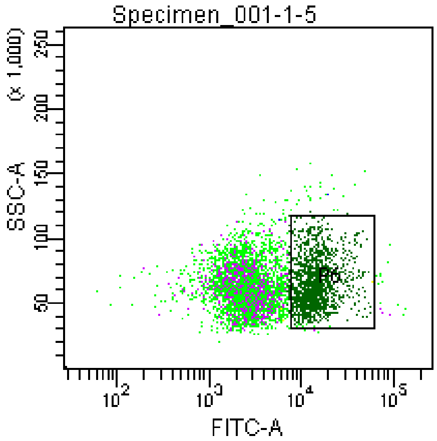

Supplement: S5 File — (ZIP) [file pone.0312147.s005.zip › Flow Cytometric Assessment/Global Sheet1_12052022164835.pdf]
