# Supplementary material for: Comparative analysis of the effects of cyclophosphamide and dexamethasone on intestinal immunity and microbiota in delayed hypersensitivity mice
Source: PLoS One. 2024 Oct 17;19(10):e0312147. doi: 10.1371/journal.pone.0312147 (PMC11486373; doi:10.1371/journal.pone.0312147)

FACSDiva Version 6.2

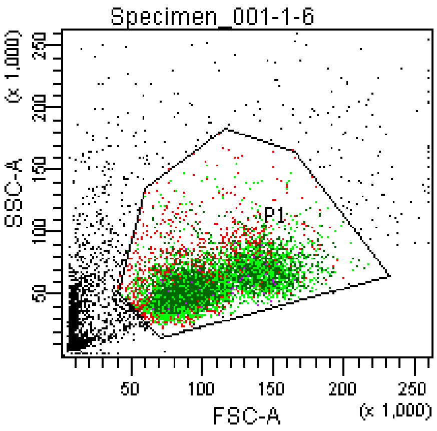

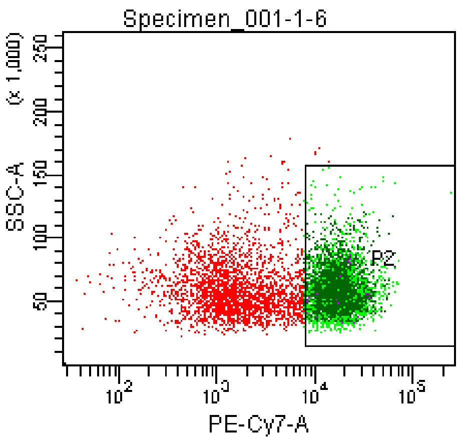

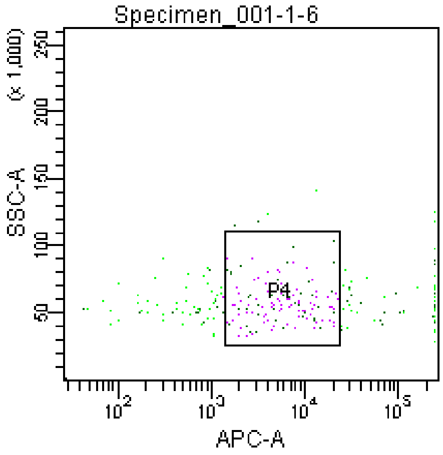

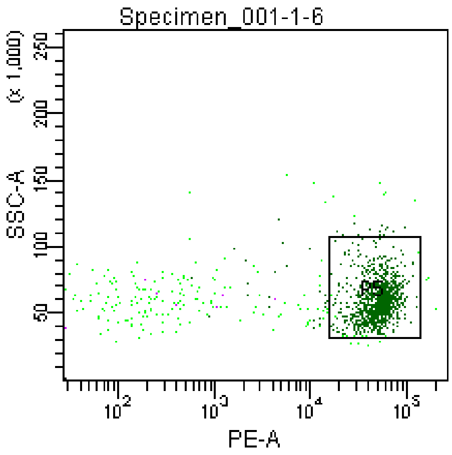

Experiment Name:	Experiment_7740
Specimen Name:	Specimen_001
Tube Name:	1-6
Record Date:	Jan 10, 2022 8:43:24 PM
$OP:	Administrator
GUID:	0dbb30cb-f18d-4106-a87c-65b5c3637155

Population	#Events	%Parent	SSC-A Mean	PE-Cy7-A Mean
P1	8,069	80.7	57,894	14,132
P2	5,494	68.1	57,503	19,729
P3	29	0.5	56,761	18,361
P5	27	93.1	57,451	18,679
P4	128	2.3	55,692	20,014
P6	1,697	30.9	60,010	19,179

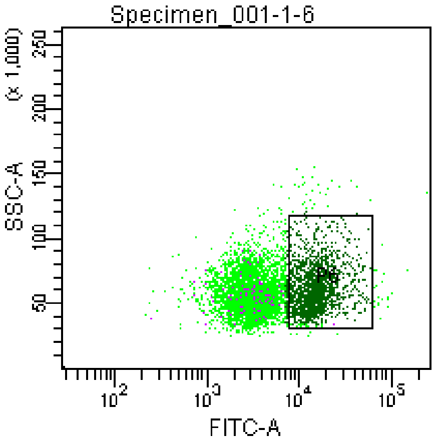

Supplement: S5 File — (ZIP) [file pone.0312147.s005.zip › Flow Cytometric Assessment/Global Sheet1_12052022164841.pdf]
