# Supplementary material for: Comparative analysis of the effects of cyclophosphamide and dexamethasone on intestinal immunity and microbiota in delayed hypersensitivity mice
Source: PLoS One. 2024 Oct 17;19(10):e0312147. doi: 10.1371/journal.pone.0312147 (PMC11486373; doi:10.1371/journal.pone.0312147)

FACSDiva Version 6.2

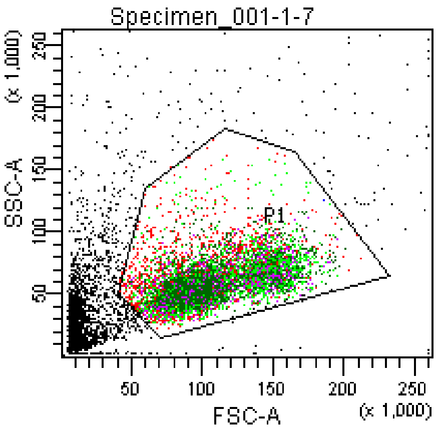

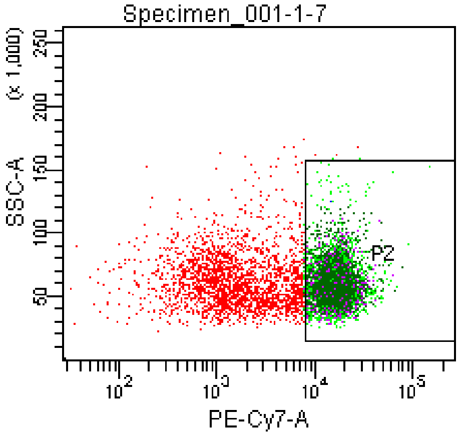

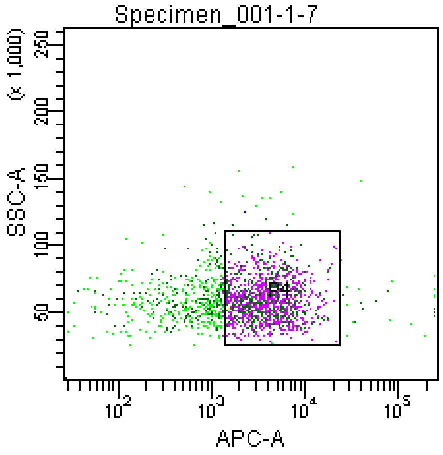

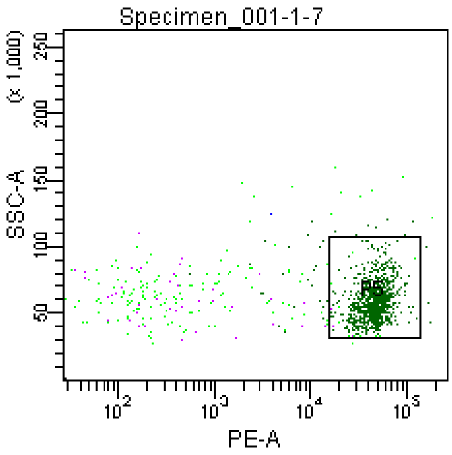

Experiment Name:	Experiment_7740
Specimen Name:	Specimen_001
Tube Name:	1-7
Record Date:	Jan 10, 2022 8:44:07 PM
$OP:	Administrator
GUID:	e7bf61b3-b938-4a08-83d4-6ddbf3023591

Population	#Events	%Parent	SSC-A Mean	PE-Cy7-A Mean
P1	7,217	72.2	58,985	12,958
P2	4,849	67.2	58,170	18,160
P3	250	5.2	57,534	16,796
P5	231	92.4	56,557	16,971
P4	1,160	23.9	57,480	18,129
P6	1,413	29.1	59,956	17,948

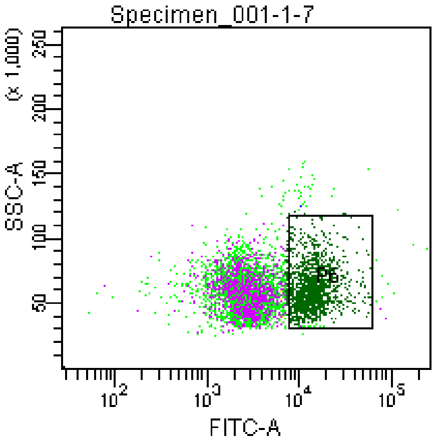

Supplement: S5 File — (ZIP) [file pone.0312147.s005.zip › Flow Cytometric Assessment/Global Sheet1_12052022164847.pdf]
