# Supplementary material for: Comparative analysis of the effects of cyclophosphamide and dexamethasone on intestinal immunity and microbiota in delayed hypersensitivity mice
Source: PLoS One. 2024 Oct 17;19(10):e0312147. doi: 10.1371/journal.pone.0312147 (PMC11486373; doi:10.1371/journal.pone.0312147)

FACSDiva Version 6.2

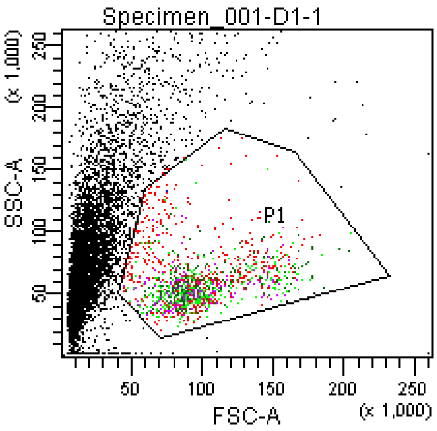

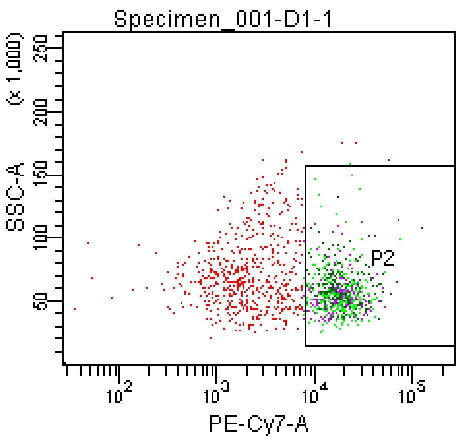

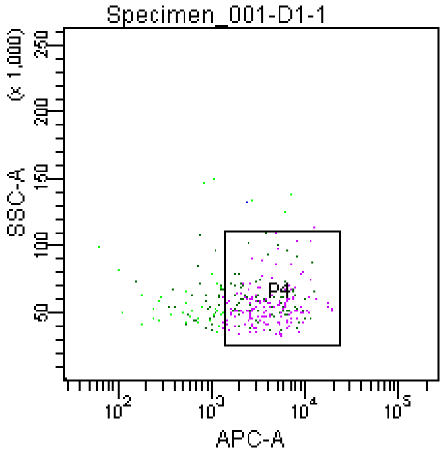

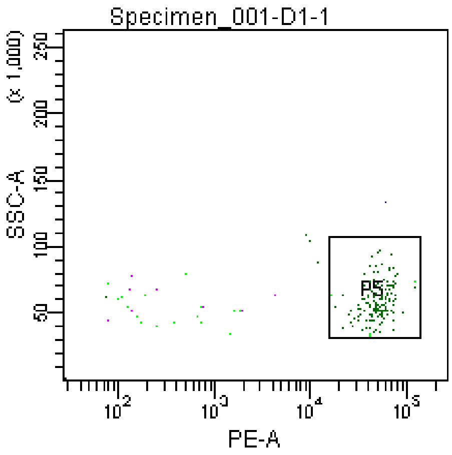

Experiment Name: Experiment_7740
Specimen Name: Specimen_001
Tube Name: D1-1
Record Date: Jan 10, 2022 8:45:15 PM
$OP: Administrator
GUID: b2f8fe5f-ca94-4a1c-a9d9-2c0f7f6aab11

Population	#Events	%Parent	SSC-A Mean	PE-Cy7-A Mean
P1	1,276	12.8	62,456	12,576
P2	699	54.8	55,569	20,645
P3	64	9.2	60,927	21,349
P5	59	92.2	58,718	20,069
P4	236	33.8	55,763	20,502
P6	221	31.6	58,560	21,965

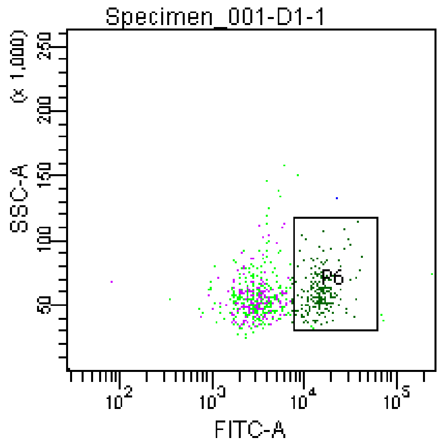

Supplement: S5 File — (ZIP) [file pone.0312147.s005.zip › Flow Cytometric Assessment/Global Sheet1_12052022164854.pdf]
