# Supplementary material for: Comparative analysis of the effects of cyclophosphamide and dexamethasone on intestinal immunity and microbiota in delayed hypersensitivity mice
Source: PLoS One. 2024 Oct 17;19(10):e0312147. doi: 10.1371/journal.pone.0312147 (PMC11486373; doi:10.1371/journal.pone.0312147)

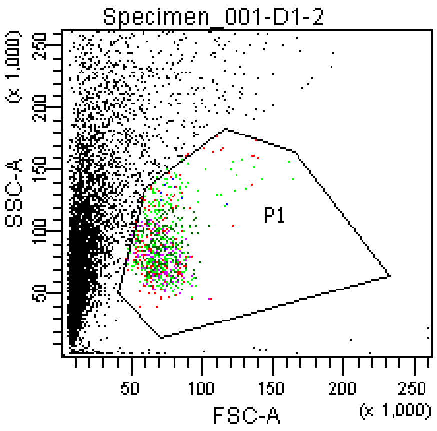

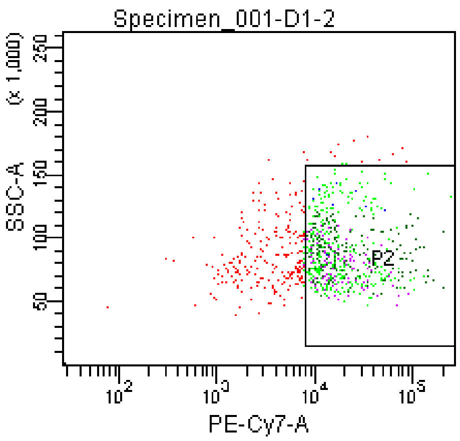

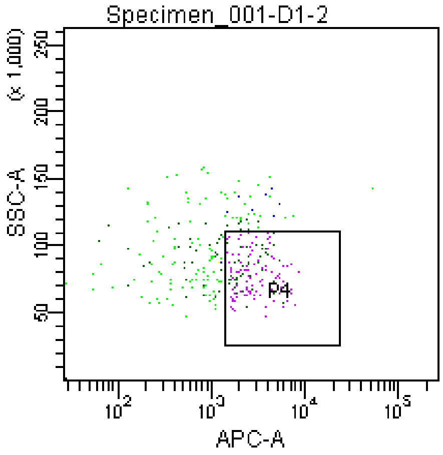

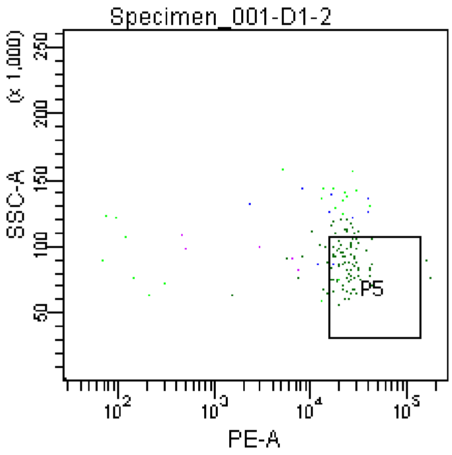

Experiment Name:	Experiment_7740
Specimen Name:	Specimen_001
Tube Name:	D1-2
Record Date:	Jan 10, 2022 8:47:32 PM
$OP:	Administrator
GUID:	a98c3ed9-fcee-4f96-9469-407cae868517

Population	#Events	%Parent	SSC-A Mean	PE-Cy7-A Mean
P1	850	8.5	87,743	20,992
P2	620	72.9	87,489	26,625
P3	48	7.7	89,465	20,373
P5	30	62.5	79,044	21,633
P4	148	23.9	78,455	23,099
P6	190	30.6	86,847	35,315

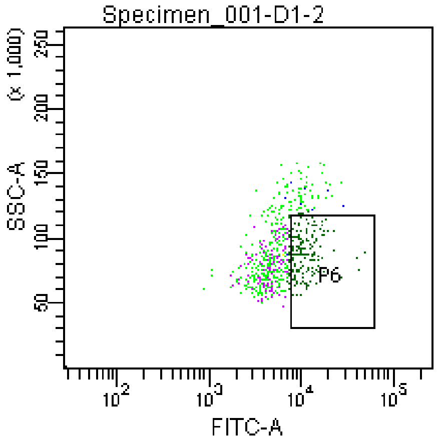

Supplement: S5 File — (ZIP) [file pone.0312147.s005.zip › Flow Cytometric Assessment/Global Sheet1_12052022164859.pdf]
