# Supplementary material for: Comparative analysis of the effects of cyclophosphamide and dexamethasone on intestinal immunity and microbiota in delayed hypersensitivity mice
Source: PLoS One. 2024 Oct 17;19(10):e0312147. doi: 10.1371/journal.pone.0312147 (PMC11486373; doi:10.1371/journal.pone.0312147)

FACSDiva Version 6.2

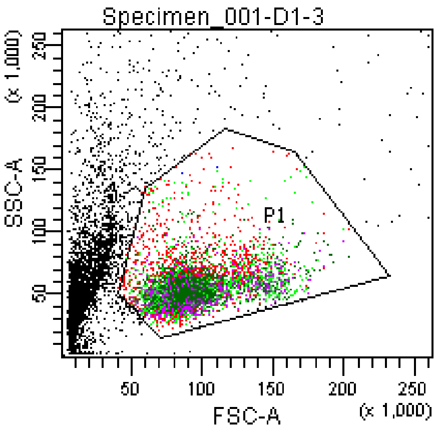

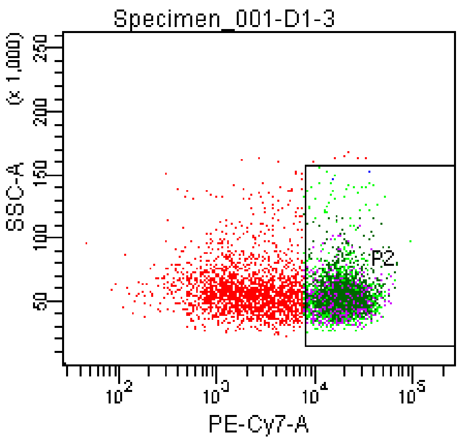

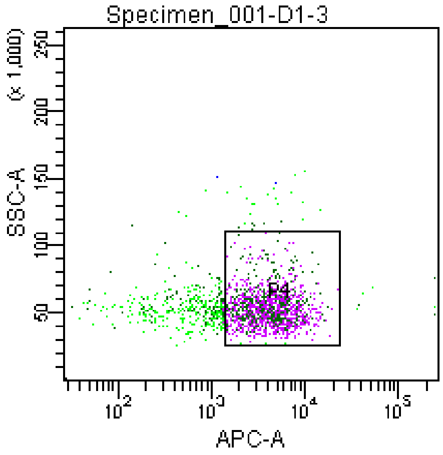

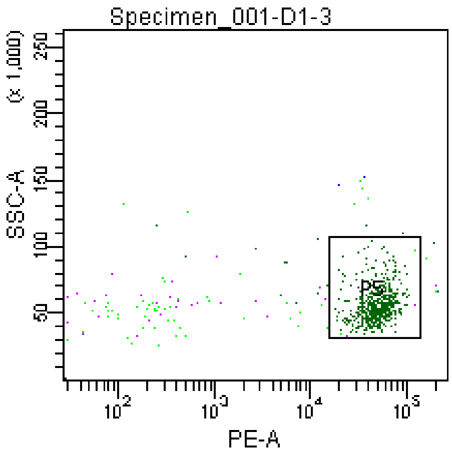

Experiment Name: Experiment_7740
Specimen Name: Specimen_001
Tube Name: D1-3
Record Date: Jan 10, 2022 8:49:42 PM
$OP: Administrator
GUID: 0c77bf87-42a0-4617-a178-963d72d9e08e

Population	#Events	%Parent	SSC-A Mean	PE-Cy7-A Mean
P1	5,669	56.7	54,481	14,563
P2	3,591	63.3	52,631	21,362
P3	246	6.9	55,165	21,480
P5	231	93.9	54,027	21,318
P4	1,235	34.4	51,597	21,402
P6	946	26.3	55,864	22,346

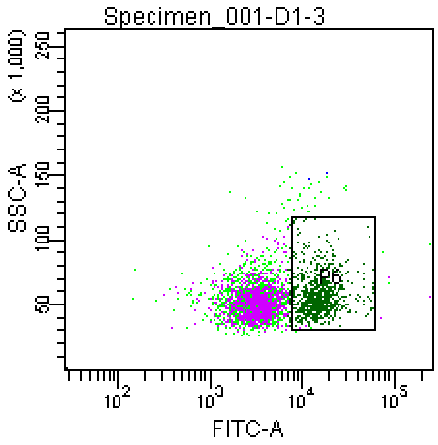

Supplement: S5 File — (ZIP) [file pone.0312147.s005.zip › Flow Cytometric Assessment/Global Sheet1_12052022164908.pdf]
