# Supplementary material for: Comparative analysis of the effects of cyclophosphamide and dexamethasone on intestinal immunity and microbiota in delayed hypersensitivity mice
Source: PLoS One. 2024 Oct 17;19(10):e0312147. doi: 10.1371/journal.pone.0312147 (PMC11486373; doi:10.1371/journal.pone.0312147)

FACSDiva Version 6.2

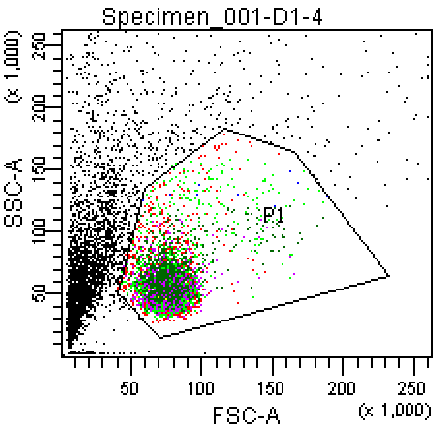

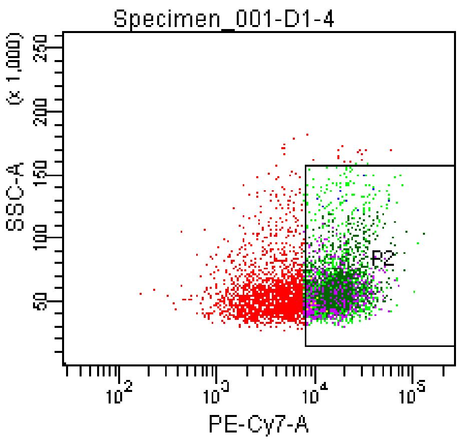

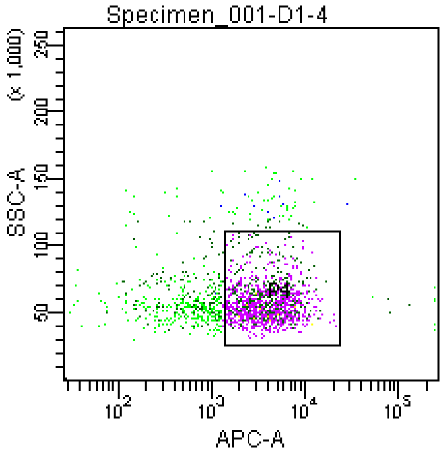

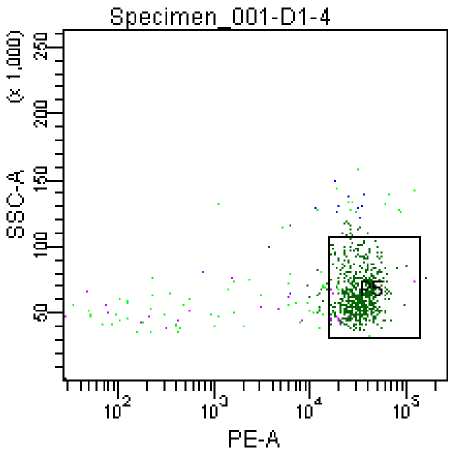

Experiment Name: Experiment_7740
Specimen Name: Specimen_001
Tube Name: D1-4
Record Date: Jan 10, 2022 8:50:50 PM
$OP: Administrator
GUID: 53503928-baaf-46d1-b5e9-b0846d1970a2

Population	#Events	%Parent	SSC-A Mean	PE-Cy7-A Mean
P1	5,718	57.2	58,091	13,768
P2	3,739	65.4	59,130	18,778
P3	278	7.4	65,728	20,568
P5	253	91.0	61,924	19,994
P4	1,241	33.2	56,172	18,459
P6	856	22.9	64,858	21,450

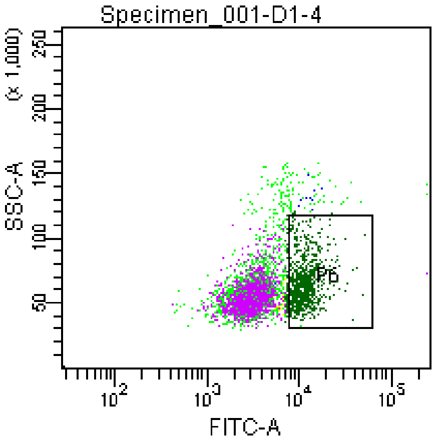

Supplement: S5 File — (ZIP) [file pone.0312147.s005.zip › Flow Cytometric Assessment/Global Sheet1_12052022164913.pdf]
