# Supplementary material for: Comparative analysis of the effects of cyclophosphamide and dexamethasone on intestinal immunity and microbiota in delayed hypersensitivity mice
Source: PLoS One. 2024 Oct 17;19(10):e0312147. doi: 10.1371/journal.pone.0312147 (PMC11486373; doi:10.1371/journal.pone.0312147)

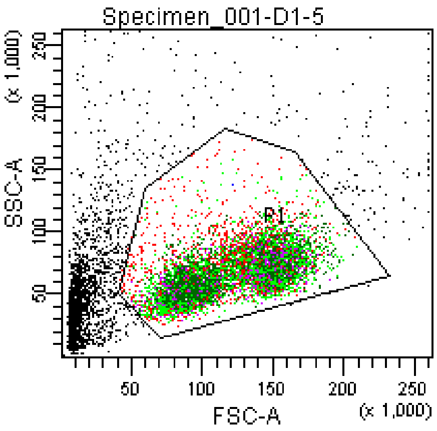

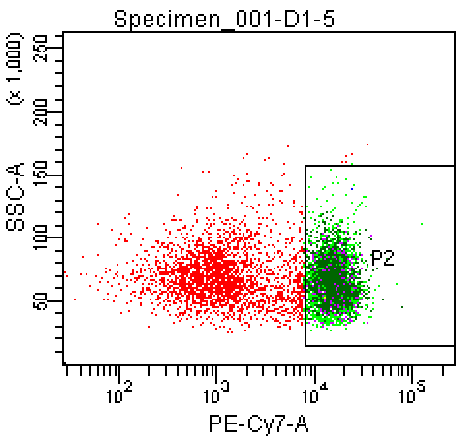

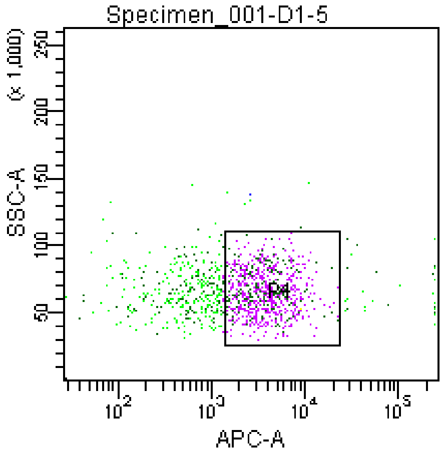

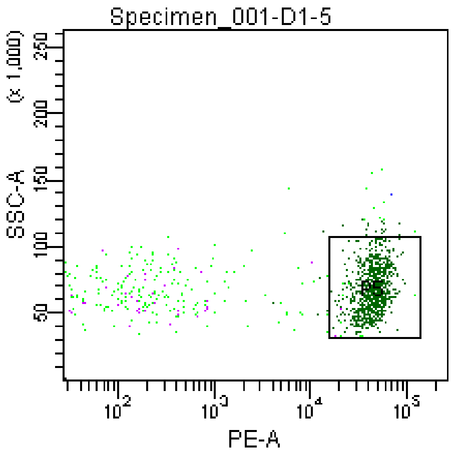

Experiment Name: Experiment_7740
Specimen Name: Specimen_001
Tube Name: D1-5
Record Date: Jan 10, 2022 8:51:28 PM
$OP: Administrator
GUID: b3f887be-40c2-4137-b3dd-6d1ab12f1919

Population	#Events	%Parent	SSC-A Mean	PE-Cy7-A Mean
P1	7,236	72.4	65,689	11,172
P2	4,496	62.1	63,913	16,920
P3	140	3.1	67,200	15,353
P5	136	97.1	66,488	15,323
P4	726	16.1	63,733	16,914
P6	1,148	25.5	66,280	16,815

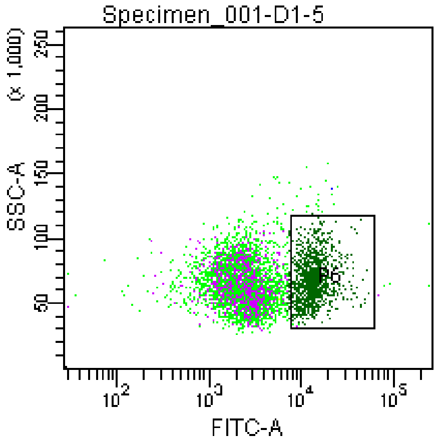

Supplement: S5 File — (ZIP) [file pone.0312147.s005.zip › Flow Cytometric Assessment/Global Sheet1_12052022164918.pdf]
