# Supplementary material for: Comparative analysis of the effects of cyclophosphamide and dexamethasone on intestinal immunity and microbiota in delayed hypersensitivity mice
Source: PLoS One. 2024 Oct 17;19(10):e0312147. doi: 10.1371/journal.pone.0312147 (PMC11486373; doi:10.1371/journal.pone.0312147)

FACSDiva Version 6.2

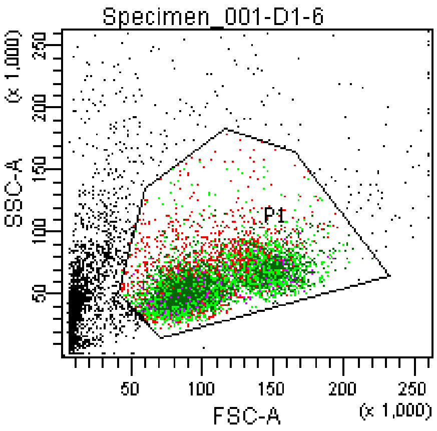

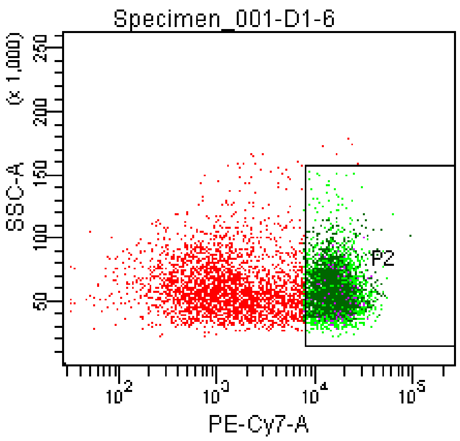

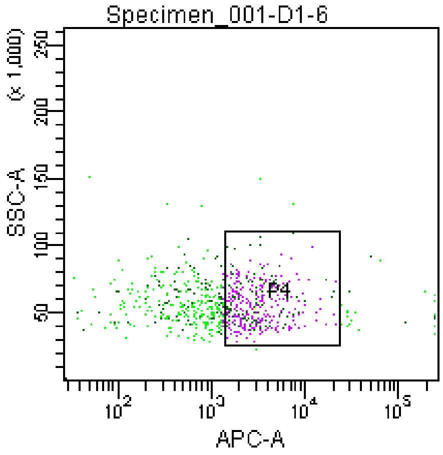

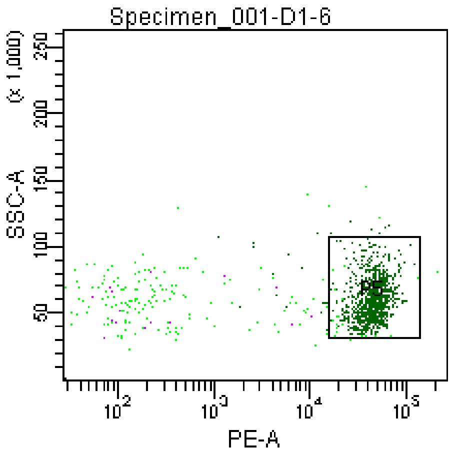

Experiment Name: Experiment_7740
Specimen Name: Specimen_001
Tube Name: D1-6
Record Date: Jan 10, 2022 8:52:04 PM
$OP: Administrator
GUID: 31bd137e-4cc4-4141-90c6-157e9766f2d2

Population	#Events	%Parent	SSC-A Mean	PE-Cy7-A Mean
P1	7,631	76.3	57,767	11,809
P2	4,788	62.7	56,247	17,648
P3	83	1.7	58,015	16,660
P5	80	96.4	58,283	16,798
P4	369	7.7	55,200	17,468
P6	1,265	26.4	59,883	17,129

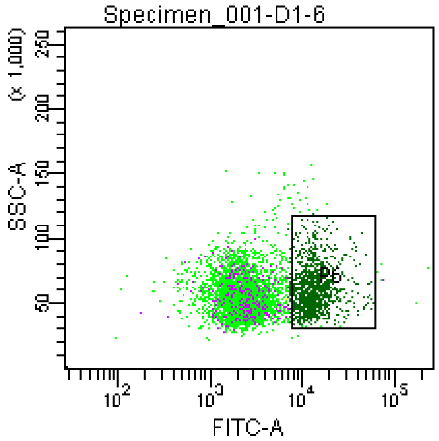

Supplement: S5 File — (ZIP) [file pone.0312147.s005.zip › Flow Cytometric Assessment/Global Sheet1_12052022164924.pdf]
