# Supplementary material for: Comparative analysis of the effects of cyclophosphamide and dexamethasone on intestinal immunity and microbiota in delayed hypersensitivity mice
Source: PLoS One. 2024 Oct 17;19(10):e0312147. doi: 10.1371/journal.pone.0312147 (PMC11486373; doi:10.1371/journal.pone.0312147)

FACSDiva Version 6.2

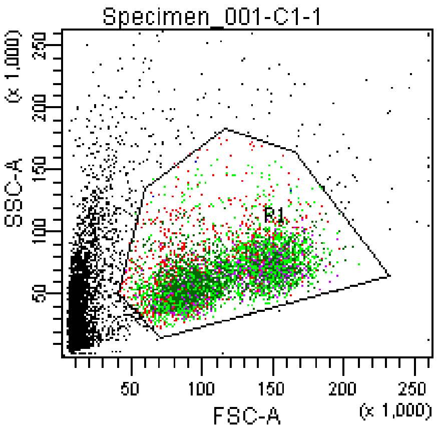

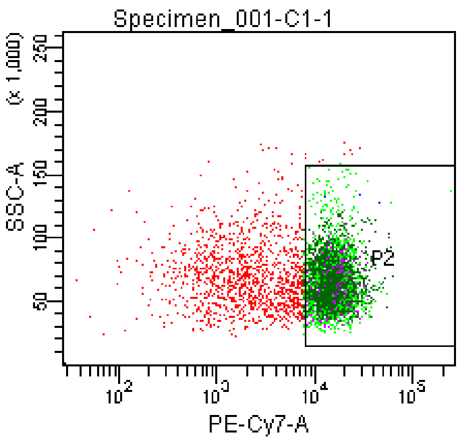

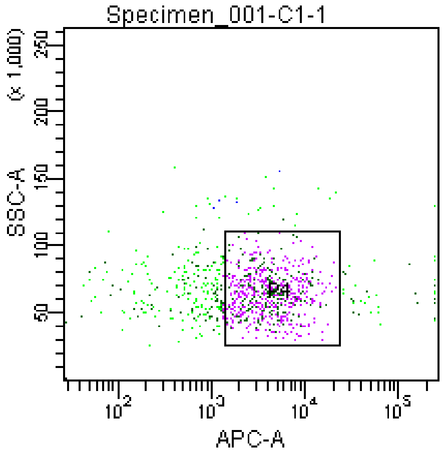

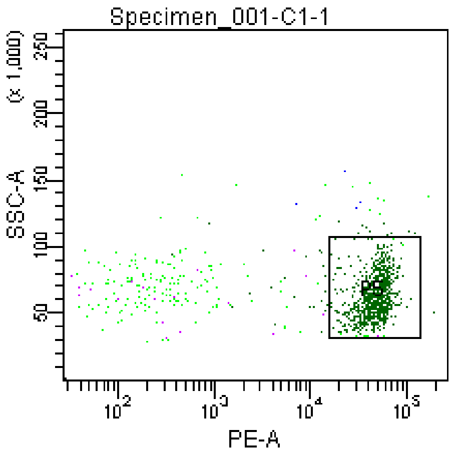

Experiment Name: Experiment_7740
Specimen Name: Specimen_001
Tube Name: C1-1
Record Date: Jan 10, 2022 8:52:38 PM
$OP: Administrator
GUID: 064e10b6-6e8a-4ad3-b00a-be1e8422f1b7

Population	#Events	%Parent	SSC-A Mean	PE-Cy7-A Mean
P1	5,525	55.2	65,206	13,818
P2	4,260	77.1	64,030	17,070
P3	154	3.6	62,006	16,643
P5	142	92.2	60,064	16,151
P4	619	14.5	62,801	17,119
P6	1,278	30.0	64,479	17,055

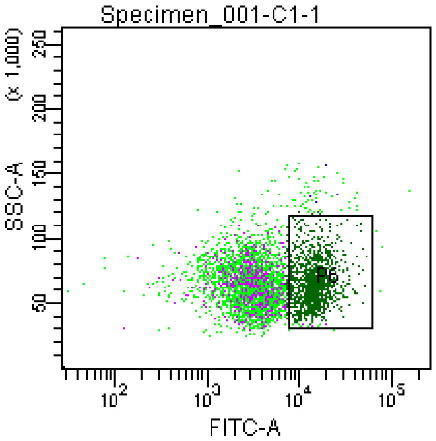

Supplement: S5 File — (ZIP) [file pone.0312147.s005.zip › Flow Cytometric Assessment/Global Sheet1_12052022164931.pdf]
