# Supplementary material for: Comparative analysis of the effects of cyclophosphamide and dexamethasone on intestinal immunity and microbiota in delayed hypersensitivity mice
Source: PLoS One. 2024 Oct 17;19(10):e0312147. doi: 10.1371/journal.pone.0312147 (PMC11486373; doi:10.1371/journal.pone.0312147)

FACSDiva Version 6.2

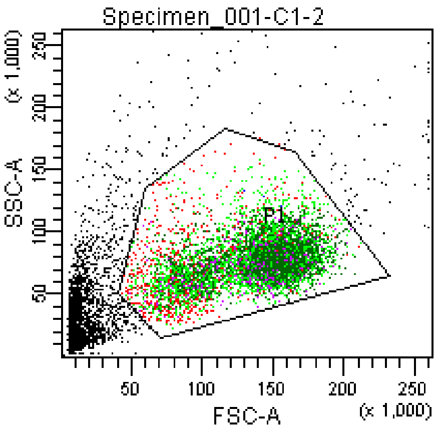

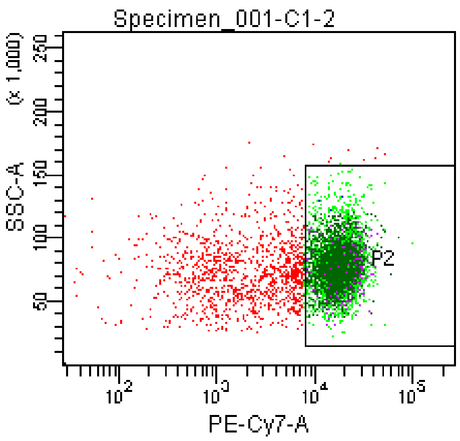

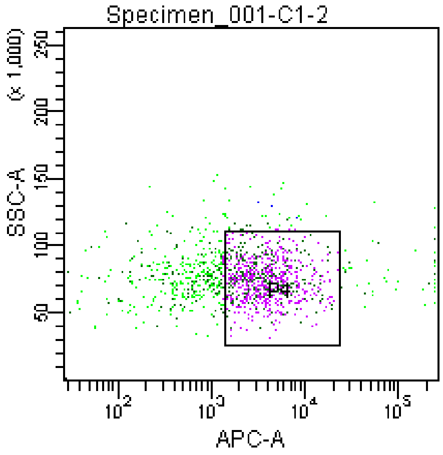

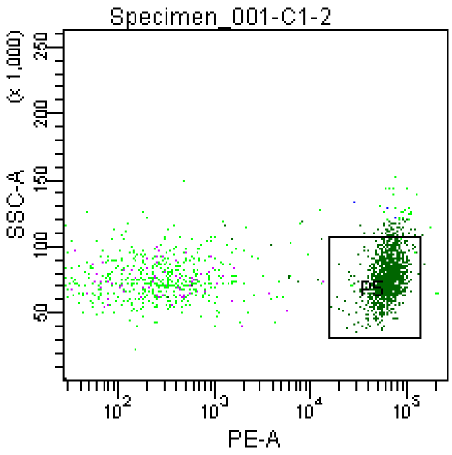

Experiment Name:	Experiment_7740
Specimen Name:	Specimen_001
Tube Name:	C1-2
Record Date:	Jan 10, 2022 8:53:10 PM
$OP:	Administrator
GUID:	0de27bc5-42a5-413c-81c7-1f89ec1968c5

Population	#Events	%Parent	SSC-A Mean	PE-Cy7-A Mean
P1	7,007	70.1	74,841	16,771
P2	5,821	83.1	75,267	19,579
P3	171	2.9	74,163	16,376
P5	156	91.2	72,307	16,268
P4	767	13.2	72,862	19,640
P6	1,626	27.9	77,037	18,003

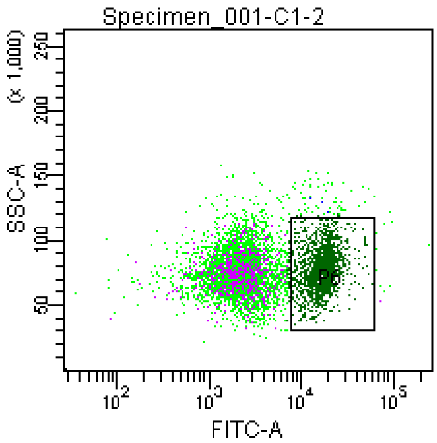

Supplement: S5 File — (ZIP) [file pone.0312147.s005.zip › Flow Cytometric Assessment/Global Sheet1_12052022164936.pdf]
