# Supplementary material for: Comparative analysis of the effects of cyclophosphamide and dexamethasone on intestinal immunity and microbiota in delayed hypersensitivity mice
Source: PLoS One. 2024 Oct 17;19(10):e0312147. doi: 10.1371/journal.pone.0312147 (PMC11486373; doi:10.1371/journal.pone.0312147)

FACSDiva Version 6.2

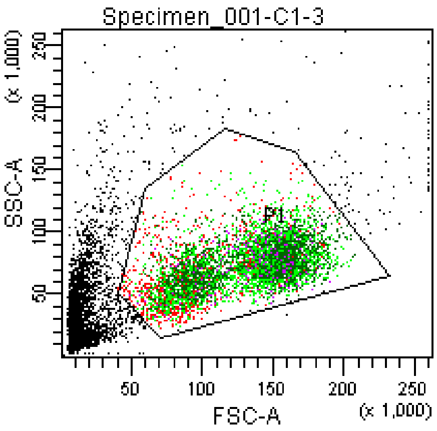

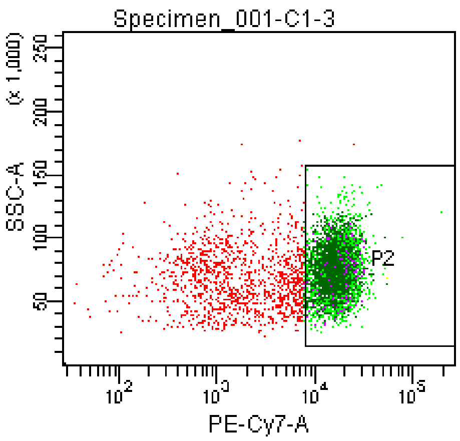

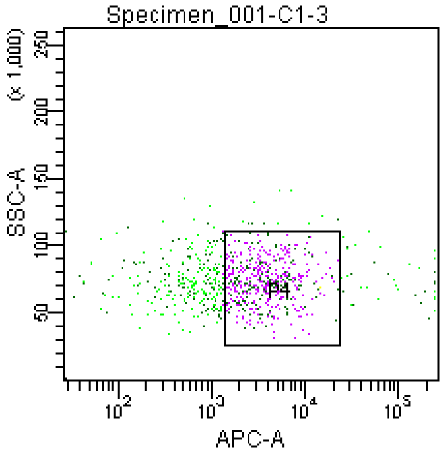

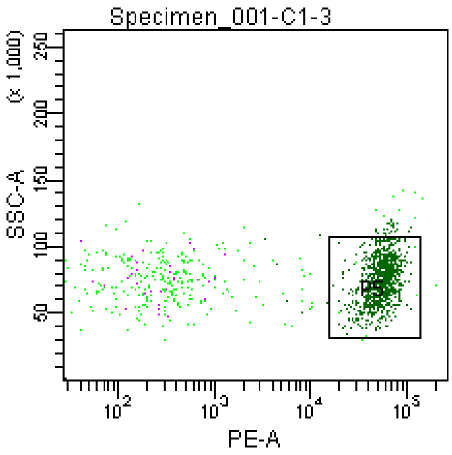

Experiment Name:	Experiment_7740
Specimen Name:	Specimen_001
Tube Name:	C1-3
Record Date:	Jan 10, 2022 8:53:49 PM
$OP:	Administrator
GUID:	65477284-2f77-4a9f-b92d-c37f1a463205

Population	#Events	%Parent	SSC-A Mean	PE-Cy7-A Mean
P1	5,853	58.5	70,916	14,856
P2	4,601	78.6	72,198	18,176
P3	128	2.8	68,879	15,815
P5	124	96.9	67,421	15,774
P4	498	10.8	71,889	18,274
P6	1,400	30.4	73,550	16,772

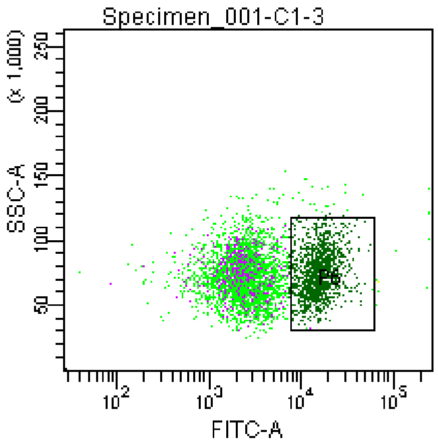

Supplement: S5 File — (ZIP) [file pone.0312147.s005.zip › Flow Cytometric Assessment/Global Sheet1_12052022164941.pdf]
