# Supplementary material for: Comparative analysis of the effects of cyclophosphamide and dexamethasone on intestinal immunity and microbiota in delayed hypersensitivity mice
Source: PLoS One. 2024 Oct 17;19(10):e0312147. doi: 10.1371/journal.pone.0312147 (PMC11486373; doi:10.1371/journal.pone.0312147)

FACSDiva Version 6.2

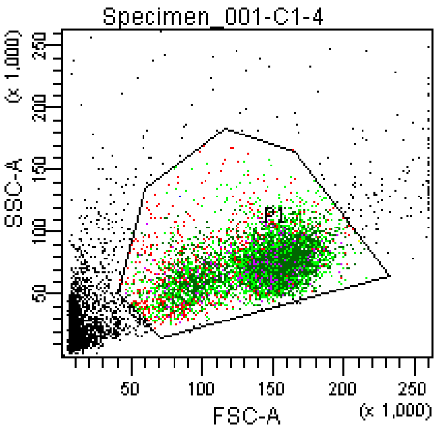

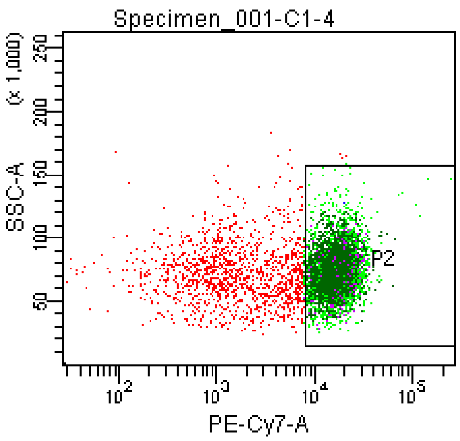

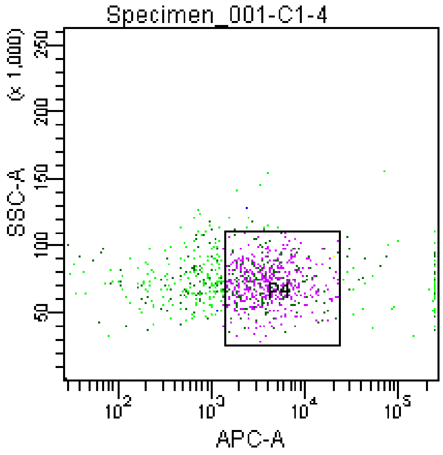

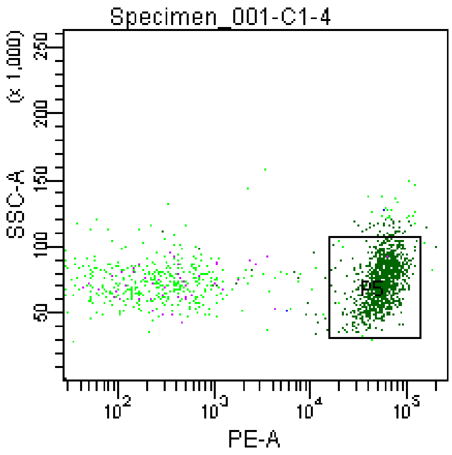

Experiment Name: Experiment_7740
Specimen Name: Specimen_001
Tube Name: C1-4
Record Date: Jan 10, 2022 8:54:13 PM
$OP: Administrator
GUID: 81332de9-7996-4a52-a85b-21c2dddbb62b

Population	#Events	%Parent	SSC-A Mean	PE-Cy7-A Mean
P1	7,304	73.0	71,704	15,432
P2	5,885	80.6	72,067	18,573
P3	115	2.0	70,273	15,707
P5	105	91.3	68,912	15,122
P4	602	10.2	70,383	18,480
P6	1,764	30.0	72,870	16,889

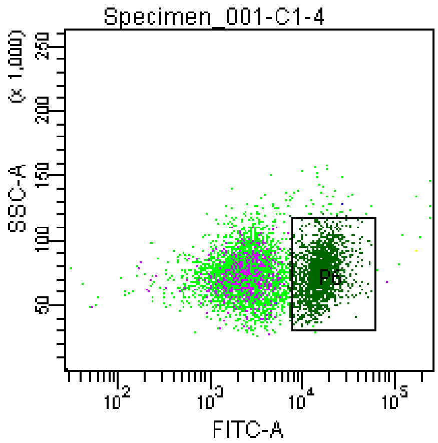

Supplement: S5 File — (ZIP) [file pone.0312147.s005.zip › Flow Cytometric Assessment/Global Sheet1_12052022164948.pdf]
