# Supplementary material for: Comparative analysis of the effects of cyclophosphamide and dexamethasone on intestinal immunity and microbiota in delayed hypersensitivity mice
Source: PLoS One. 2024 Oct 17;19(10):e0312147. doi: 10.1371/journal.pone.0312147 (PMC11486373; doi:10.1371/journal.pone.0312147)

FACSDiva Version 6.2

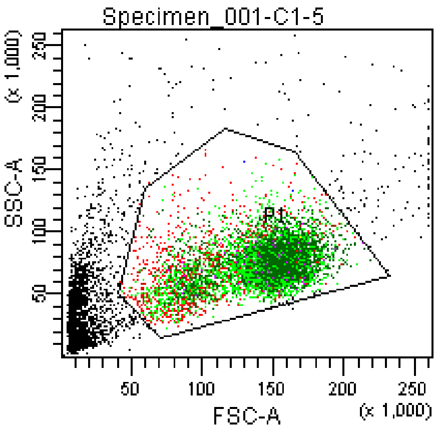

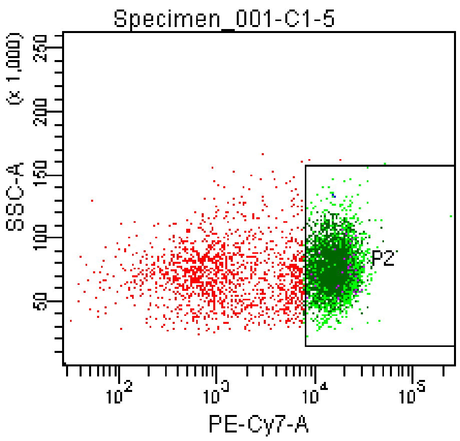

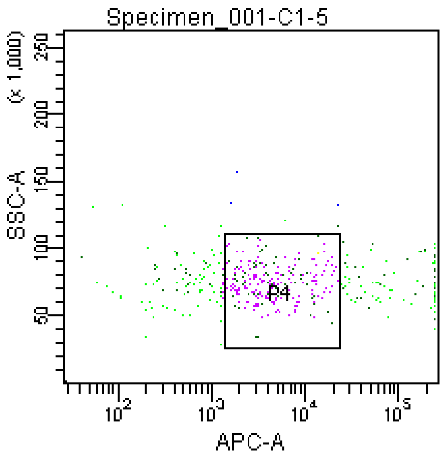

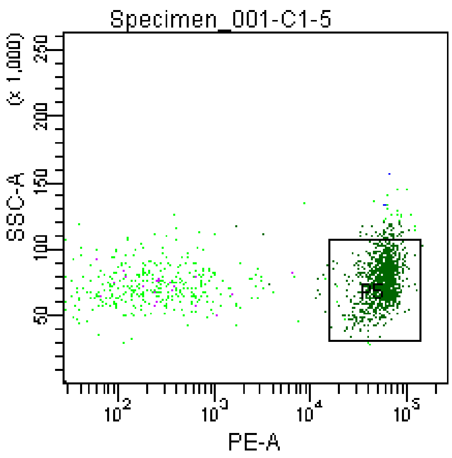

Experiment Name:	Experiment_7740
Specimen Name:	Specimen_001
Tube Name:	C1-5
Record Date:	Jan 10, 2022 8:54:42 PM
$OP:	Administrator
GUID:	607a71ee-e575-43c7-8a64-611cbe6abccc

Population	#Events	%Parent	SSC-A Mean	PE-Cy7-A Mean
P1	7,086	70.9	71,933	14,460
P2	5,556	78.4	72,614	17,869
P3	53	1.0	76,823	16,120
P5	47	88.7	72,144	15,892
P4	240	4.3	73,489	18,454
P6	1,625	29.2	75,682	16,248

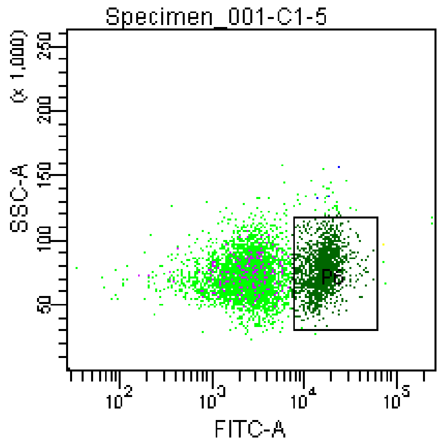

Supplement: S5 File — (ZIP) [file pone.0312147.s005.zip › Flow Cytometric Assessment/Global Sheet1_12052022164954.pdf]
