# Supplementary material for: Comparative analysis of the effects of cyclophosphamide and dexamethasone on intestinal immunity and microbiota in delayed hypersensitivity mice
Source: PLoS One. 2024 Oct 17;19(10):e0312147. doi: 10.1371/journal.pone.0312147 (PMC11486373; doi:10.1371/journal.pone.0312147)

FACSDiva Version 6.2

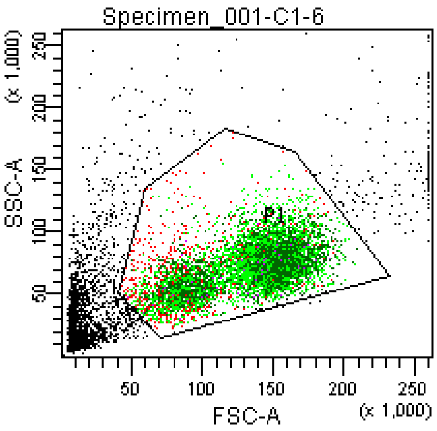

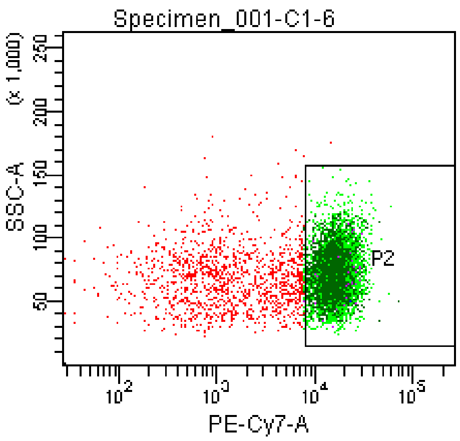

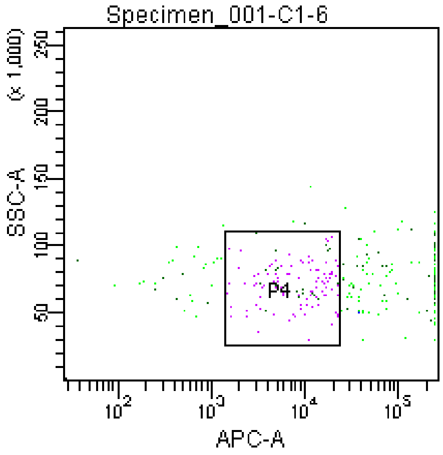

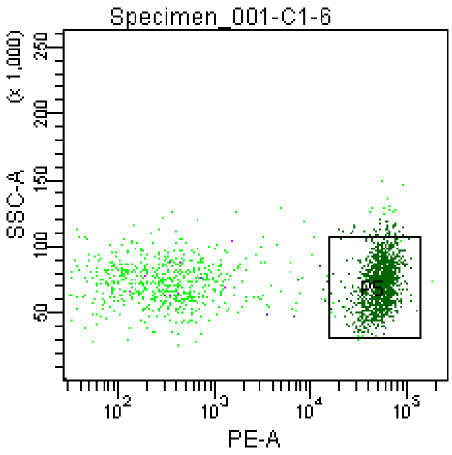

Experiment Name: Experiment_7740
Specimen Name: Specimen_001
Tube Name: C1-6
Record Date: Jan 10, 2022 8:55:00 PM
$OP: Administrator
GUID: d5cfcb24-42aa-48de-8309-a8863435213f

Population	#Events	%Parent	SSC-A Mean	PE-Cy7-A Mean
P1	7,420	74.2	69,897	15,114
P2	6,076	81.9	70,780	17,969
P3	28	0.5	77,086	15,831
P5	25	89.3	75,723	15,114
P4	122	2.0	70,191	18,012
P6	1,620	26.7	71,142	16,422

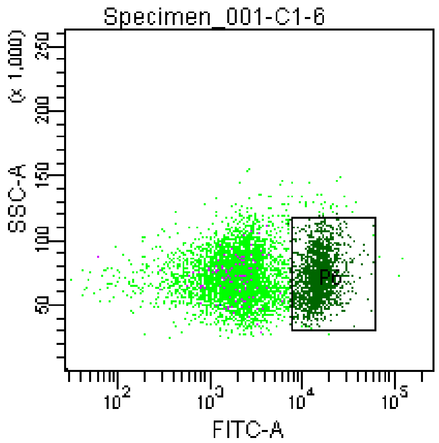

Supplement: S5 File — (ZIP) [file pone.0312147.s005.zip › Flow Cytometric Assessment/Global Sheet1_12052022164959.pdf]
