# Supplementary material for: Comparative analysis of the effects of cyclophosphamide and dexamethasone on intestinal immunity and microbiota in delayed hypersensitivity mice
Source: PLoS One. 2024 Oct 17;19(10):e0312147. doi: 10.1371/journal.pone.0312147 (PMC11486373; doi:10.1371/journal.pone.0312147)

FACSDiva Version 6.2

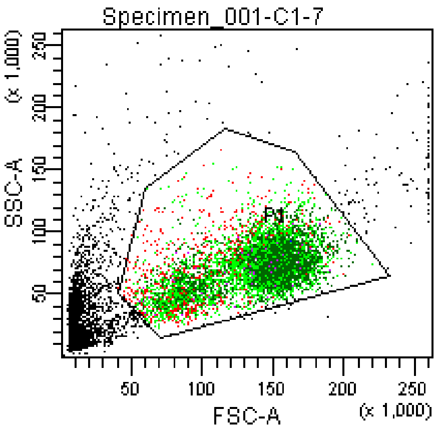

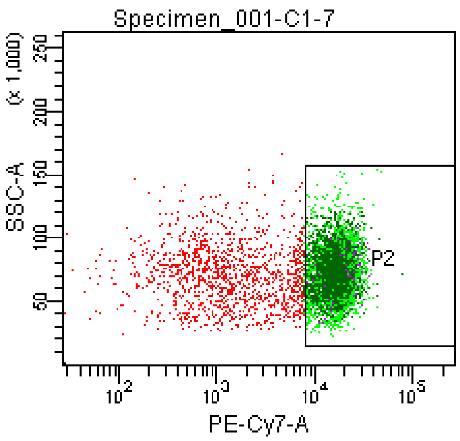

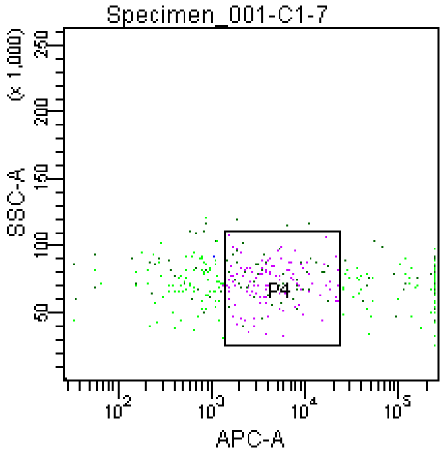

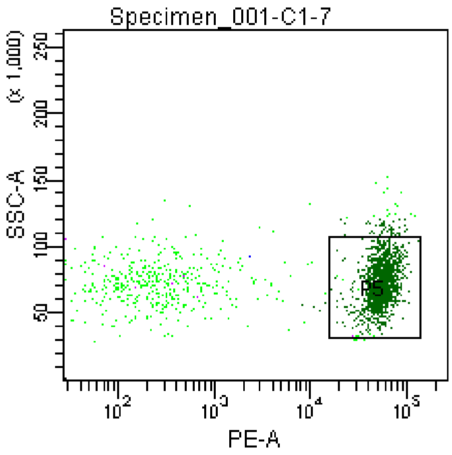

Experiment Name: Experiment_7740
Specimen Name: Specimen_001
Tube Name: C1-7
Record Date: Jan 10, 2022 8:55:47 PM
$OP: Administrator
GUID: 1a7b5150-4209-4888-801c-5b16e88ca5b5

Population	#Events	%Parent	SSC-A Mean	PE-Cy7-A Mean
P1	7,260	72.6	70,450	15,060
P2	5,862	80.7	70,787	18,132
P3	44	0.8	74,211	16,740
P5	39	88.6	73,288	15,860
P4	167	2.8	71,090	18,870
P6	1,659	28.3	72,097	16,553

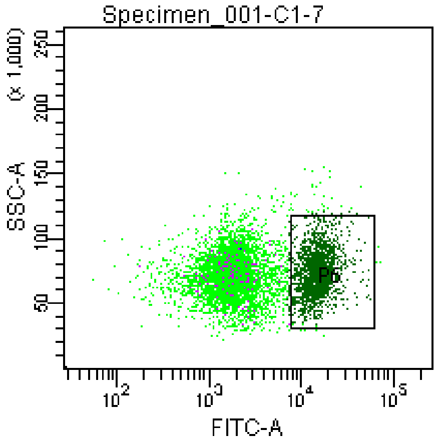

Supplement: S5 File — (ZIP) [file pone.0312147.s005.zip › Flow Cytometric Assessment/Global Sheet1_12052022165004.pdf]
