# Supplementary material for: Comparative analysis of the effects of cyclophosphamide and dexamethasone on intestinal immunity and microbiota in delayed hypersensitivity mice
Source: PLoS One. 2024 Oct 17;19(10):e0312147. doi: 10.1371/journal.pone.0312147 (PMC11486373; doi:10.1371/journal.pone.0312147)
Supplement: S5 File — (ZIP) [file pone.0312147.s005.zip › Flow Cytometric Assessment/Global Sheet1_12052022165223.pdf]

FACSDiva Version 6.2

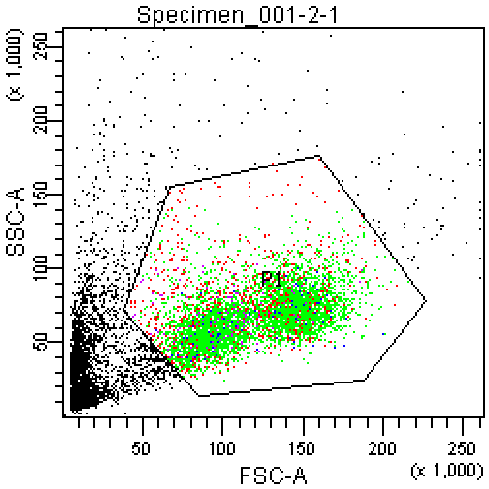

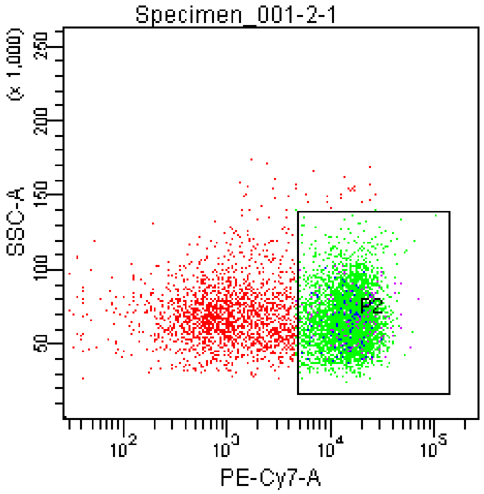

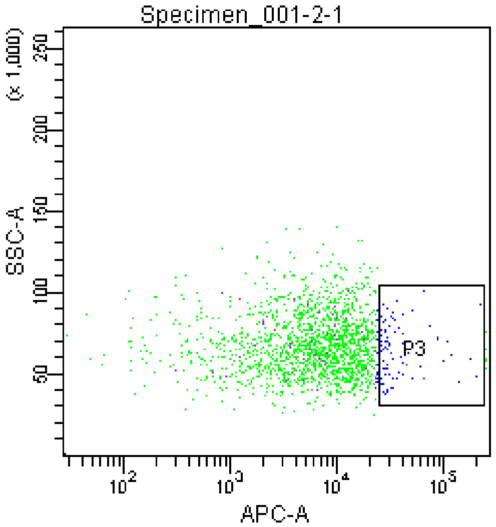

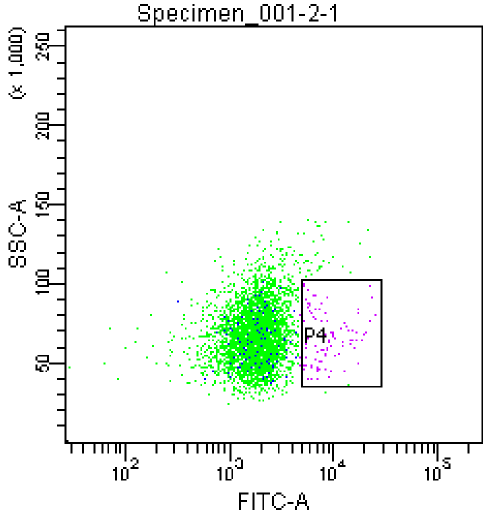

Experiment Name:	Experiment_7741
Specimen Name:	Specimen_001
Tube Name:	2-1
Record Date:	Jan 10, 2022 9:03:15 PM
$OP:	Administrator
GUID:	cbc6dc84-11f9-4e0e-858b-06e6e4548f38

Population	#Events	%Parent	SSC-A Mean	PE-Cy7-A Mean
P1	5,892	58.9	66,733	12,092
P2	4,044	68.6	65,634	16,905
P3	99	2.4	63,248	15,451
P4	114	2.8	66,853	21,408
