# Supplementary material for: Comparative analysis of the effects of cyclophosphamide and dexamethasone on intestinal immunity and microbiota in delayed hypersensitivity mice
Source: PLoS One. 2024 Oct 17;19(10):e0312147. doi: 10.1371/journal.pone.0312147 (PMC11486373; doi:10.1371/journal.pone.0312147)
Supplement: S5 File — (ZIP) [file pone.0312147.s005.zip › Flow Cytometric Assessment/Global Sheet1_12052022165232.pdf]

FACSDiva Version 6.2

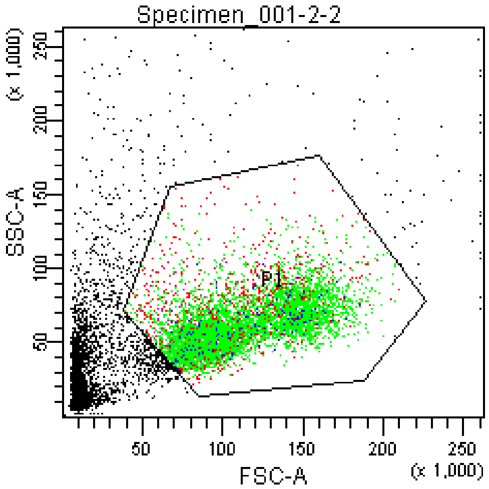

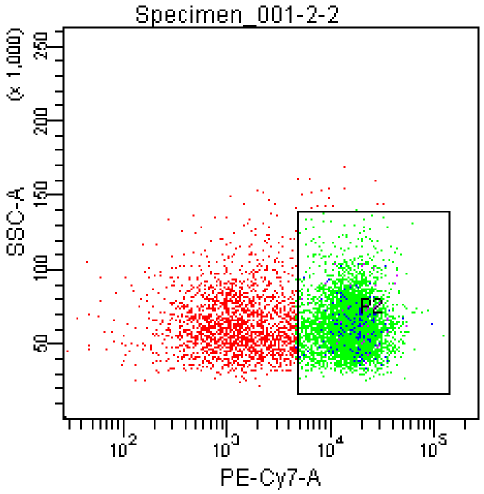

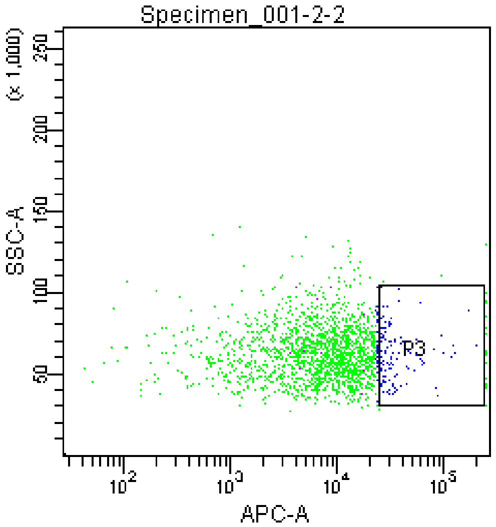

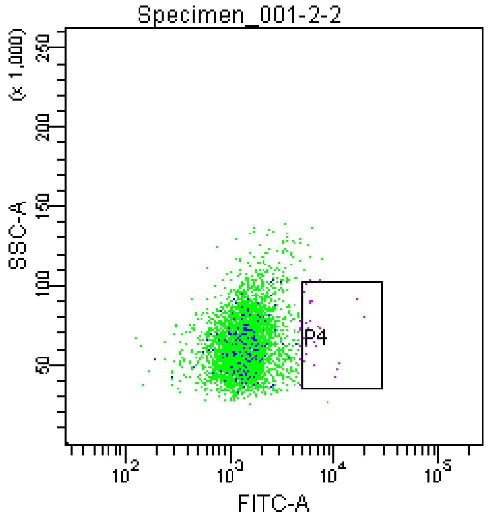

Experiment Name:	Experiment_7741
Specimen Name:	Specimen_001
Tube Name:	2-2
Record Date:	Jan 10, 2022 9:06:31 PM
$OP:	Administrator
GUID:	832e4b0b-60c3-47d7-b894-26a50f4eb46b

Population	#Events	%Parent	SSC-A Mean	PE-Cy7-A Mean
P1	6,601	66.0	61,915	13,277
P2	4,719	71.5	61,353	17,901
P3	158	3.3	60,790	19,131
P4	38	0.8	70,097	23,204
