# Supplementary material for: Comparative analysis of the effects of cyclophosphamide and dexamethasone on intestinal immunity and microbiota in delayed hypersensitivity mice
Source: PLoS One. 2024 Oct 17;19(10):e0312147. doi: 10.1371/journal.pone.0312147 (PMC11486373; doi:10.1371/journal.pone.0312147)
Supplement: S5 File — (ZIP) [file pone.0312147.s005.zip › Flow Cytometric Assessment/Global Sheet1_12052022165238.pdf]

FACSDiva Version 6.2

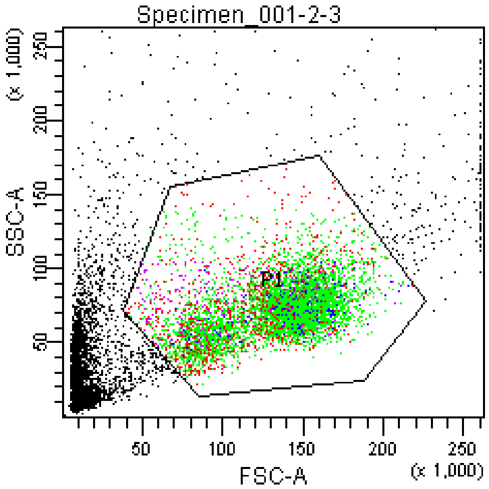

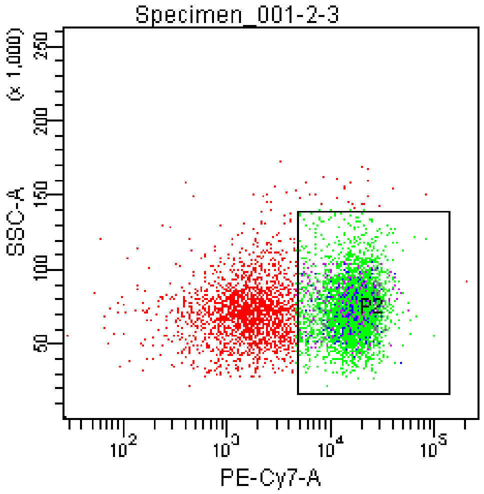

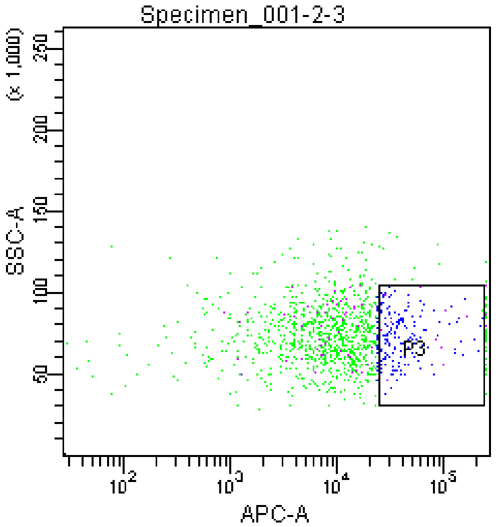

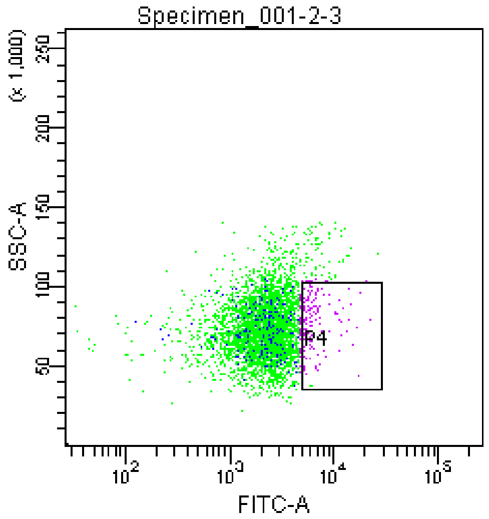

Experiment Name: Experiment_7741
Specimen Name: Specimen_001
Tube Name: 2-3
Record Date: Jan 10, 2022 9:06:53 PM
$OP: Administrator
GUID: d6a5659e-7319-4688-ba2c-388da419df2c

Population	#Events	%Parent	SSC-A Mean	PE-Cy7-A Mean
P1	6,232	62.3	71,760	12,920
P2	4,152	66.6	72,395	18,339
P3	200	4.8	71,870	18,055
P4	196	4.7	77,154	17,435
