# Supplementary material for: Comparative analysis of the effects of cyclophosphamide and dexamethasone on intestinal immunity and microbiota in delayed hypersensitivity mice
Source: PLoS One. 2024 Oct 17;19(10):e0312147. doi: 10.1371/journal.pone.0312147 (PMC11486373; doi:10.1371/journal.pone.0312147)
Supplement: S5 File — (ZIP) [file pone.0312147.s005.zip › Flow Cytometric Assessment/Global Sheet1_12052022165243.pdf]

FACSDiva Version 6.2

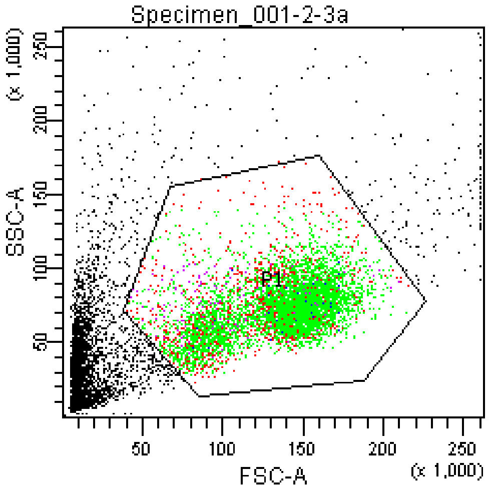

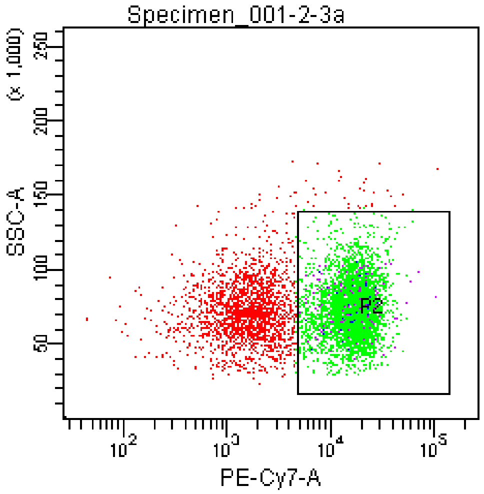

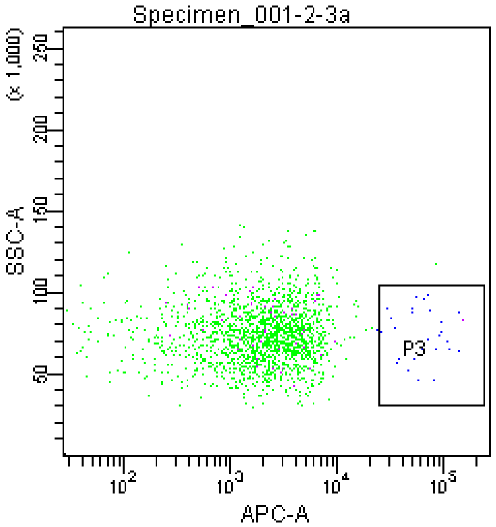

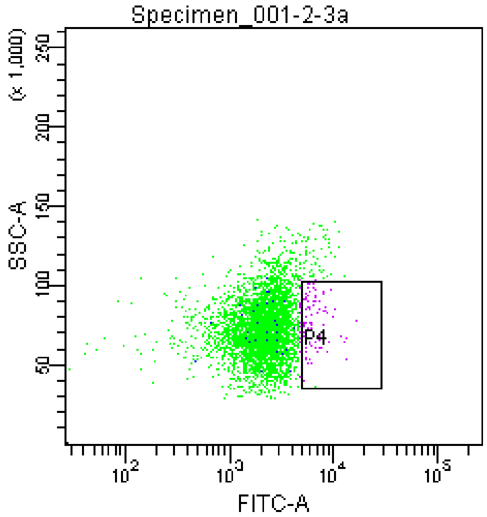

Experiment Name:	Experiment_7741
Specimen Name:	Specimen_001
Tube Name:	2-3a
Record Date:	Jan 10, 2022 9:07:48 PM
$OP:	Administrator
GUID:	afb7f5ec-a7ea-45b1-a497-bed993d80c22

Population	#Events	%Parent	SSC-A Mean	PE-Cy7-A Mean
P1	6,069	60.7	72,050	12,983
P2	4,118	67.9	72,299	18,138
P3	31	0.8	73,460	17,150
P4	113	2.7	76,323	19,841
