# Supplementary material for: Comparative analysis of the effects of cyclophosphamide and dexamethasone on intestinal immunity and microbiota in delayed hypersensitivity mice
Source: PLoS One. 2024 Oct 17;19(10):e0312147. doi: 10.1371/journal.pone.0312147 (PMC11486373; doi:10.1371/journal.pone.0312147)
Supplement: S5 File — (ZIP) [file pone.0312147.s005.zip › Flow Cytometric Assessment/Global Sheet1_12052022165248.pdf]

FACSDiva Version 6.2

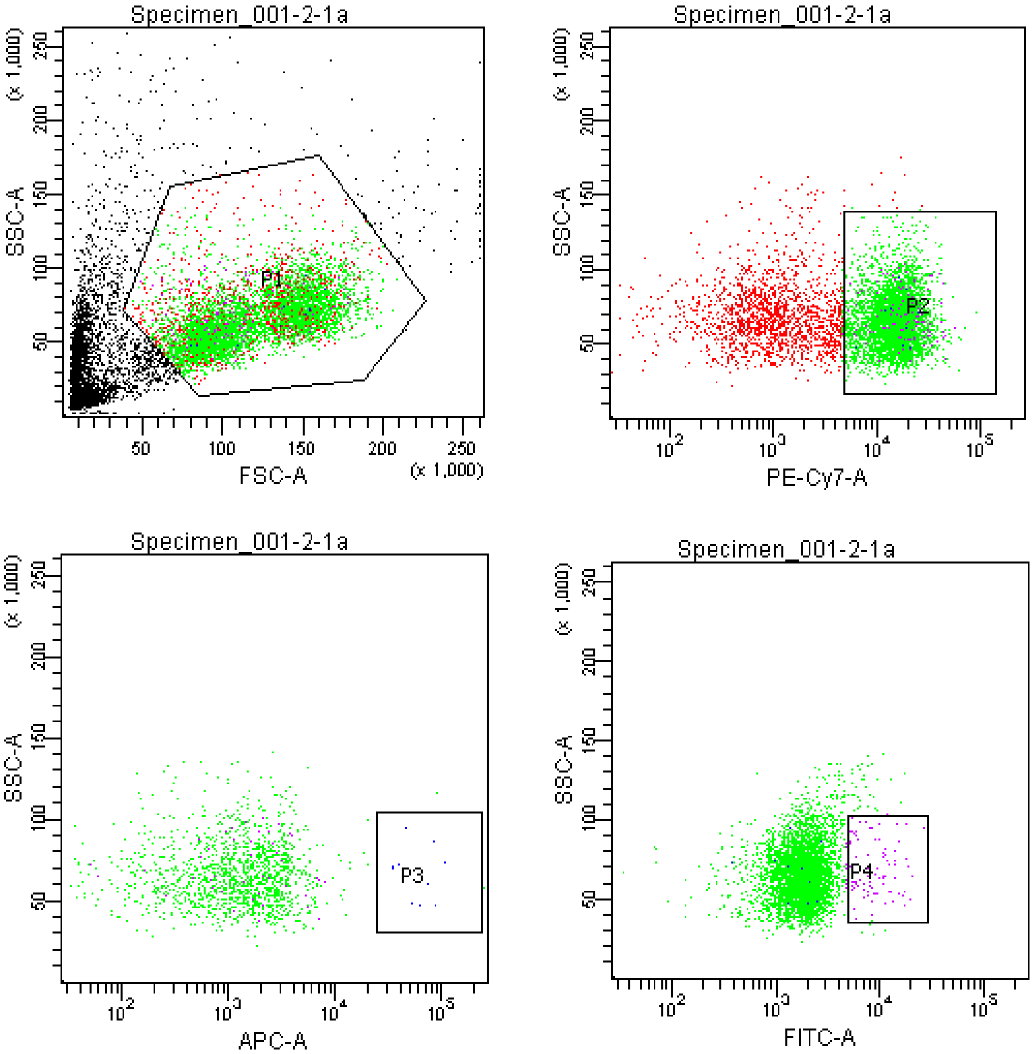

Experiment Name:	Experiment_7741
Specimen Name:	Specimen_001
Tube Name:	2-1a
Record Date:	Jan 10, 2022 9:09:56 PM
$OP:	Administrator
GUID:	0b9680cf-96c6-4339-8b0c-cfd1a9e066ec

Population	#Events	%Parent	SSC-A Mean	PE-Cy7-A Mean
P1	5,873	58.7	65,583	12,299
P2	4,072	69.3	64,505	17,087
P3	10	0.2	65,571	18,536
P4	104	2.6	68,135	21,764
