# Supplementary material for: Comparative analysis of the effects of cyclophosphamide and dexamethasone on intestinal immunity and microbiota in delayed hypersensitivity mice
Source: PLoS One. 2024 Oct 17;19(10):e0312147. doi: 10.1371/journal.pone.0312147 (PMC11486373; doi:10.1371/journal.pone.0312147)
Supplement: S5 File — (ZIP) [file pone.0312147.s005.zip › Flow Cytometric Assessment/Global Sheet1_12052022165253.pdf]

FACSDiva Version 6.2

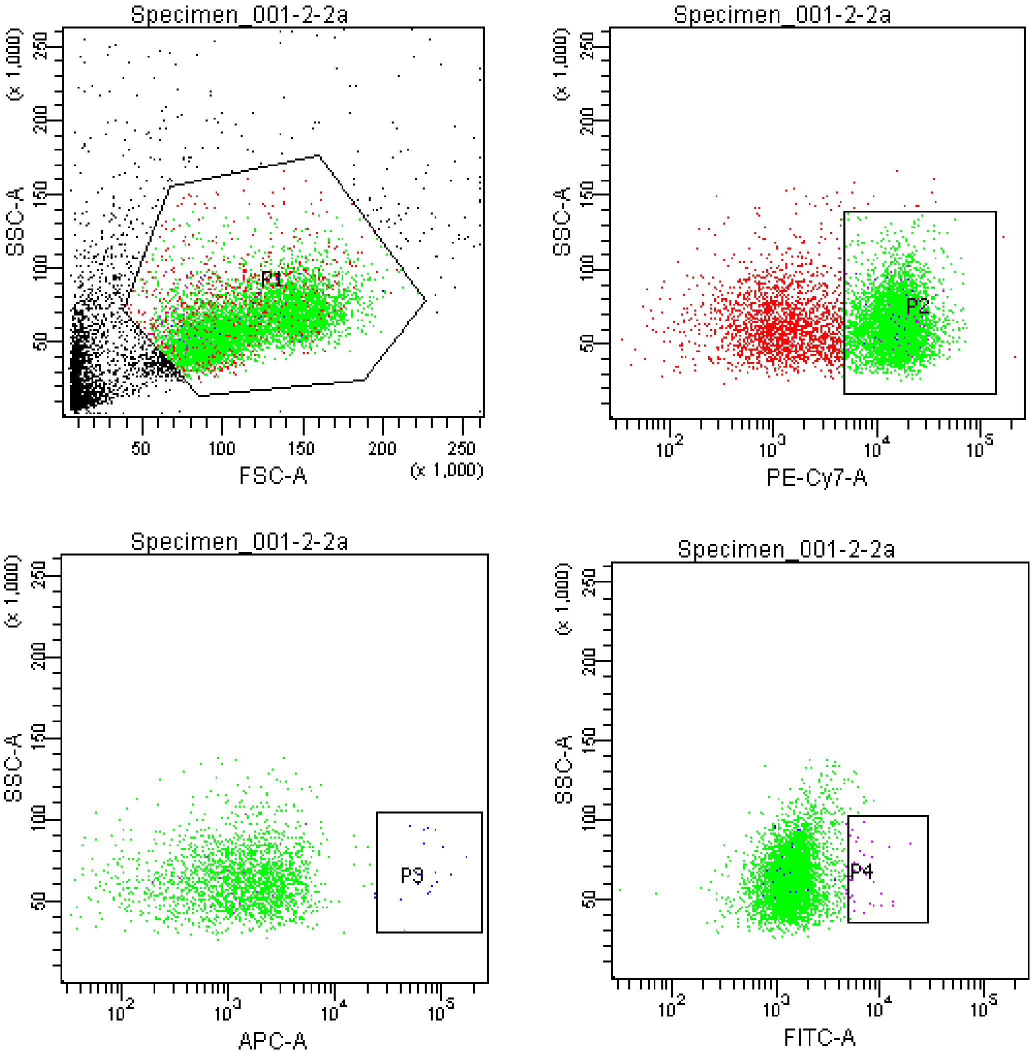

Experiment Name:	Experiment_7741
Specimen Name:	Specimen_001
Tube Name:	2-2a
Record Date:	Jan 10, 2022 9:10:33 PM
$OP:	Administrator
GUID:	af845d21-3a03-4a5f-8436-f64c0f51b36c

Population	#Events	%Parent	SSC-A Mean	PE-Cy7-A Mean
P1	7,116	71.2	62,280	13,044
P2	5,050	71.0	61,939	17,627
P3	20	0.4	68,931	16,209
P4	38	0.8	65,073	21,021
