# Supplementary material for: Comparative analysis of the effects of cyclophosphamide and dexamethasone on intestinal immunity and microbiota in delayed hypersensitivity mice
Source: PLoS One. 2024 Oct 17;19(10):e0312147. doi: 10.1371/journal.pone.0312147 (PMC11486373; doi:10.1371/journal.pone.0312147)
Supplement: S5 File — (ZIP) [file pone.0312147.s005.zip › Flow Cytometric Assessment/Global Sheet1_12052022165259.pdf]

FACSDiva Version 6.2

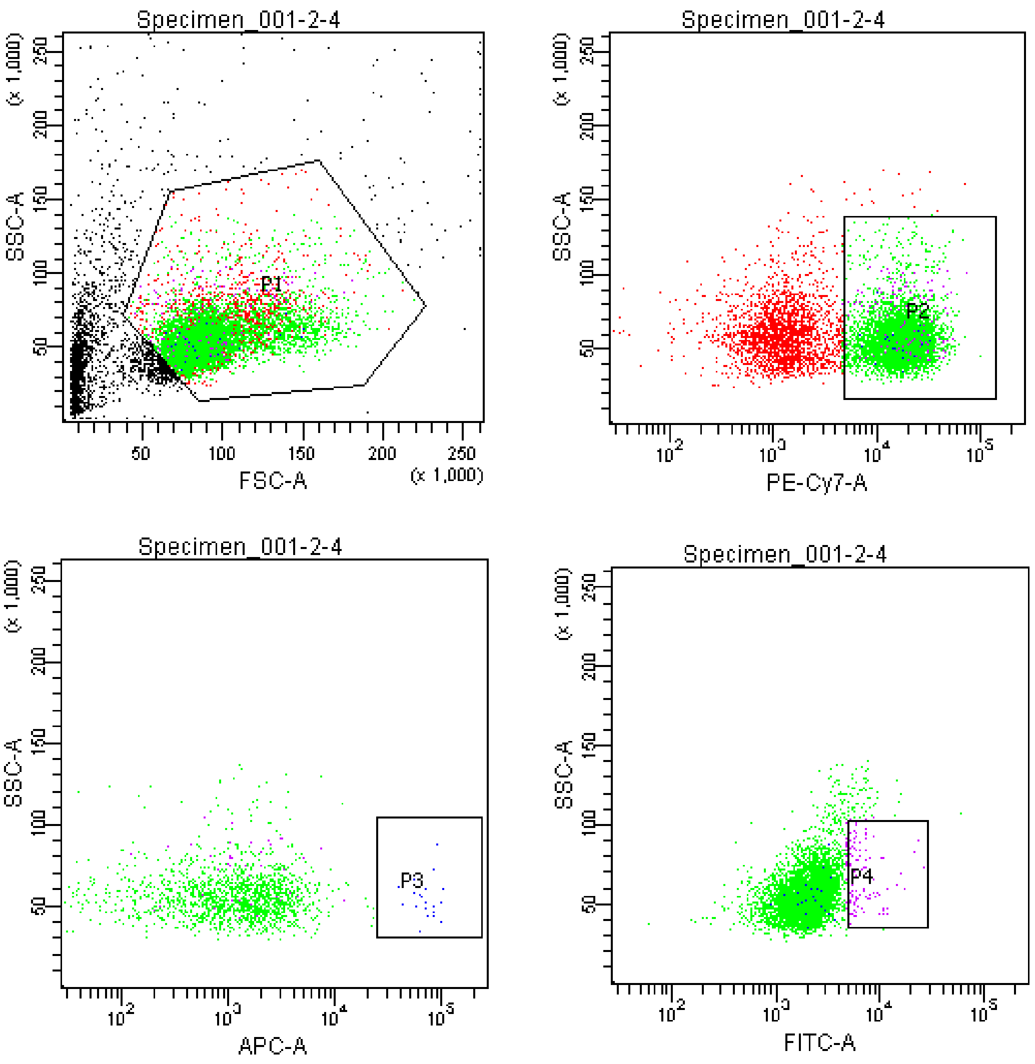

Experiment Name:	Experiment_7741
Specimen Name:	Specimen_001
Tube Name:	2-4
Record Date:	Jan 10, 2022 9:11:02 PM
$OP:	Administrator
GUID:	e90bcf81-a2c5-4949-8557-e2abc0b56eca

Population	#Events	%Parent	SSC-A Mean	PE-Cy7-A Mean
P1	7,593	75.9	56,477	14,198
P2	5,364	70.6	54,891	19,427
P3	21	0.4	52,832	19,139
P4	127	2.4	69,558	23,137
