# Supplementary material for: Comparative analysis of the effects of cyclophosphamide and dexamethasone on intestinal immunity and microbiota in delayed hypersensitivity mice
Source: PLoS One. 2024 Oct 17;19(10):e0312147. doi: 10.1371/journal.pone.0312147 (PMC11486373; doi:10.1371/journal.pone.0312147)
Supplement: S5 File — (ZIP) [file pone.0312147.s005.zip › Flow Cytometric Assessment/Global Sheet1_12052022165303.pdf]

FACSDiva Version 6.2

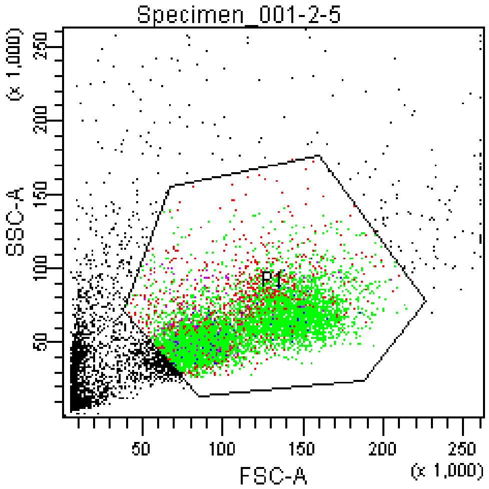

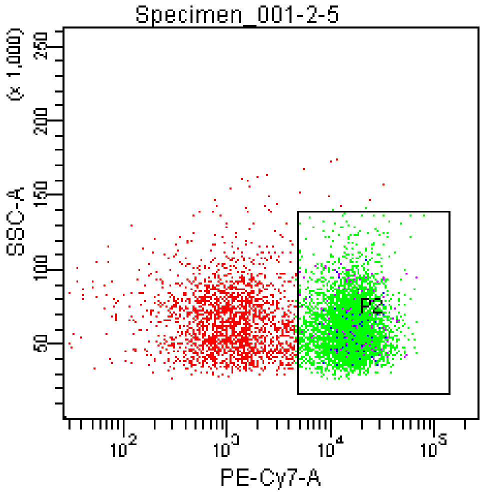

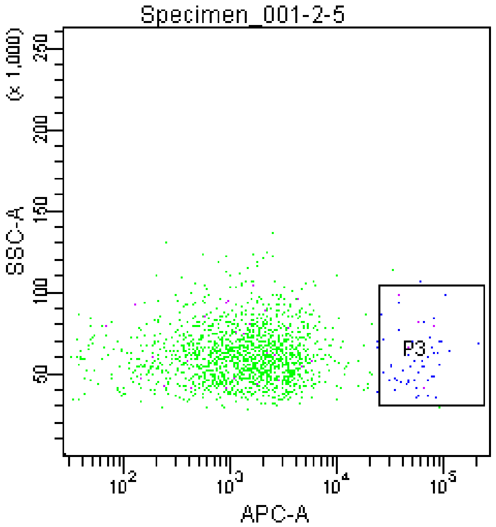

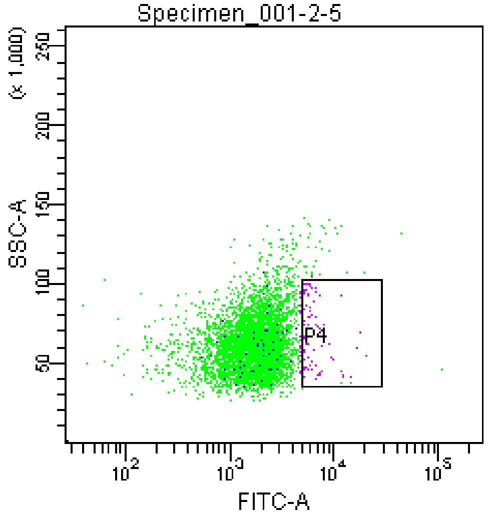

Experiment Name:	Experiment_7741
Specimen Name:	Specimen_001
Tube Name:	2-5
Record Date:	Jan 10, 2022 9:11:23 PM
$OP:	Administrator
GUID:	e58f271e-0d69-4402-af8c-c1ffec289464

Population	#Events	%Parent	SSC-A Mean	PE-Cy7-A Mean
P1	7,442	74.4	61,156	13,061
P2	5,267	70.8	59,953	17,865
P3	60	1.1	58,838	19,610
P4	97	1.8	68,785	21,896
