# Supplementary material for: Comparative analysis of the effects of cyclophosphamide and dexamethasone on intestinal immunity and microbiota in delayed hypersensitivity mice
Source: PLoS One. 2024 Oct 17;19(10):e0312147. doi: 10.1371/journal.pone.0312147 (PMC11486373; doi:10.1371/journal.pone.0312147)
Supplement: S5 File — (ZIP) [file pone.0312147.s005.zip › Flow Cytometric Assessment/Global Sheet1_12052022165308.pdf]

FACSDiva Version 6.2

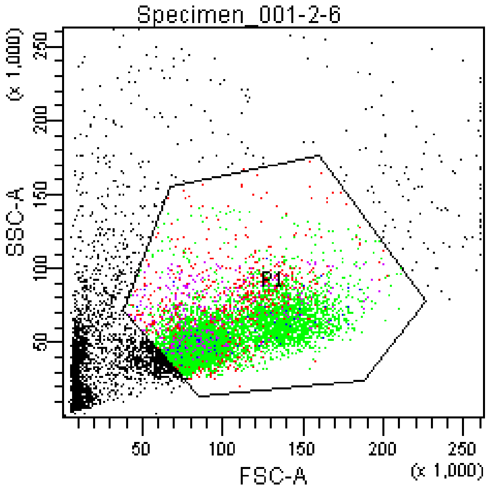

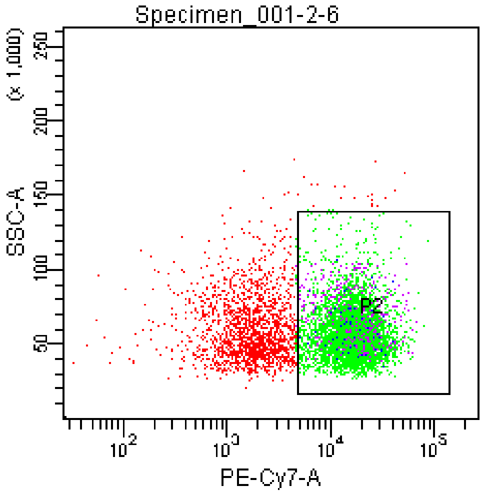

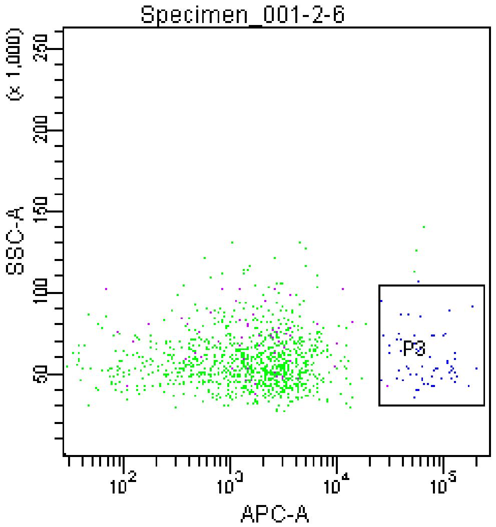

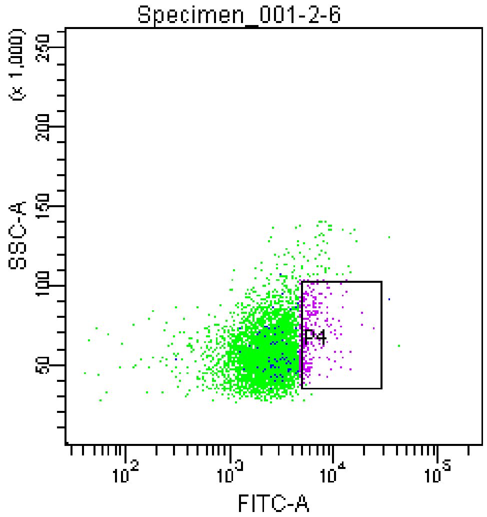

Experiment Name:	Experiment_7741
Specimen Name:	Specimen_001
Tube Name:	2-6
Record Date:	Jan 10, 2022 9:11:38 PM
$OP:	Administrator
GUID:	c47fd712-592a-4313-86f8-23f6e530366f

Population	#Events	%Parent	SSC-A Mean	PE-Cy7-A Mean
P1	7,079	70.8	57,240	14,960
P2	5,315	75.1	56,438	19,201
P3	68	1.3	57,591	19,841
P4	293	5.5	69,300	20,911
