# Supplementary material for: Comparative analysis of the effects of cyclophosphamide and dexamethasone on intestinal immunity and microbiota in delayed hypersensitivity mice
Source: PLoS One. 2024 Oct 17;19(10):e0312147. doi: 10.1371/journal.pone.0312147 (PMC11486373; doi:10.1371/journal.pone.0312147)
Supplement: S5 File — (ZIP) [file pone.0312147.s005.zip › Flow Cytometric Assessment/Global Sheet1_12052022165313.pdf]

FACSDiva Version 6.2

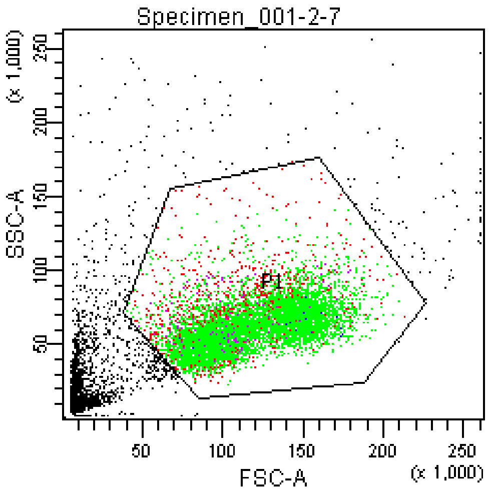

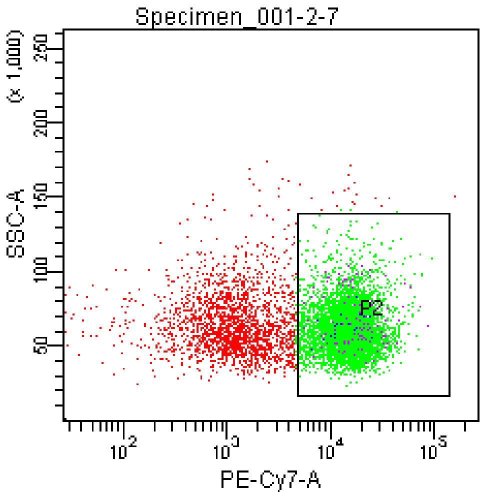

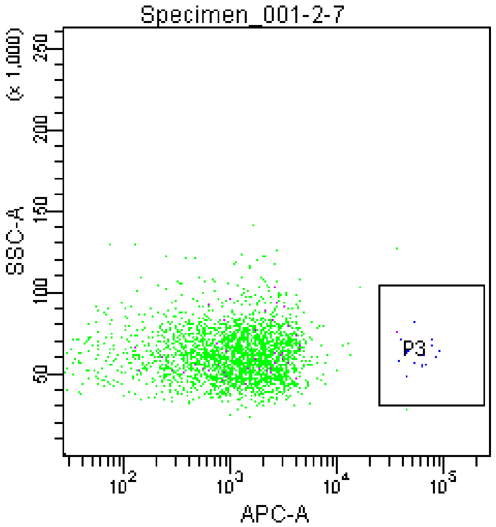

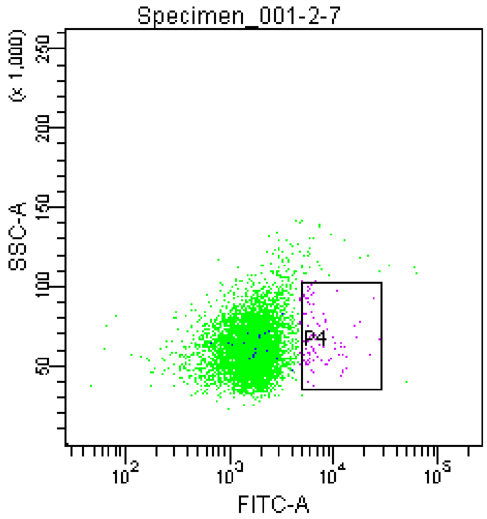

Experiment Name:	Experiment_7741
Specimen Name:	Specimen_001
Tube Name:	2-7
Record Date:	Jan 10, 2022 9:12:09 PM
$OP:	Administrator
GUID:	07c1e065-43b8-4761-bd71-d17e2044a1f8

Population	#Events	%Parent	SSC-A Mean	PE-Cy7-A Mean
P1	8,101	81.0	61,480	13,182
P2	5,979	73.8	60,523	17,249
P3	19	0.3	62,206	18,234
P4	102	1.7	67,195	21,894
