# Supplementary material for: Comparative analysis of the effects of cyclophosphamide and dexamethasone on intestinal immunity and microbiota in delayed hypersensitivity mice
Source: PLoS One. 2024 Oct 17;19(10):e0312147. doi: 10.1371/journal.pone.0312147 (PMC11486373; doi:10.1371/journal.pone.0312147)
Supplement: S5 File — (ZIP) [file pone.0312147.s005.zip › Flow Cytometric Assessment/Global Sheet1_12052022165317.pdf]

FACSDiva Version 6.2

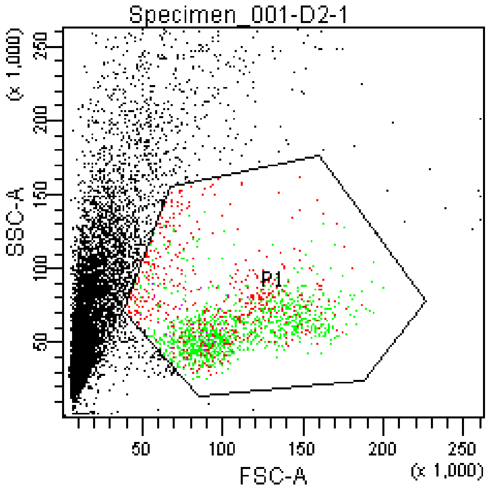

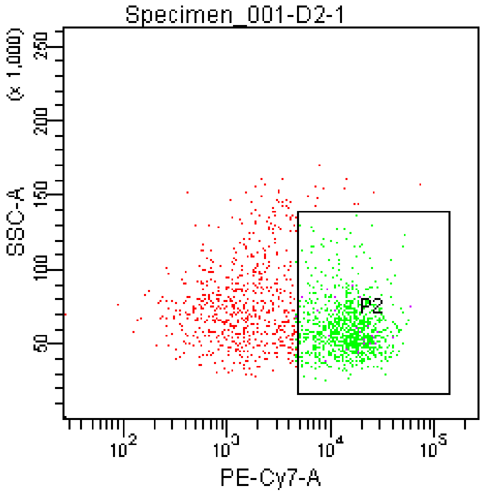

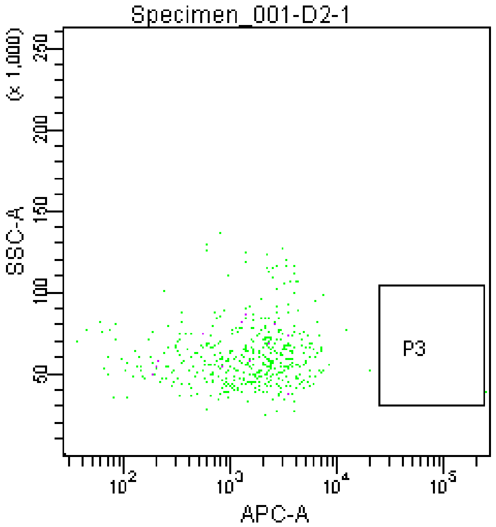

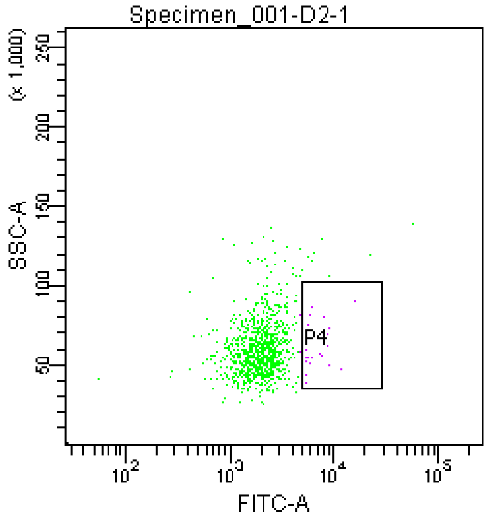

Experiment Name: Experiment_7741
Specimen Name: Specimen_001
Tube Name: D2-1
Record Date: Jan 10, 2022 9:13:09 PM
$OP: Administrator
GUID: bd831908-0371-4c07-890d-f844d7525561

Population	#Events	%Parent	SSC-A Mean	PE-Cy7-A Mean
P1	1,614	16.1	65,976	11,389
P2	977	60.5	59,110	17,462
P3	0	0.0	####	####
P4	25	2.6	60,177	20,387
