# Supplementary material for: Comparative analysis of the effects of cyclophosphamide and dexamethasone on intestinal immunity and microbiota in delayed hypersensitivity mice
Source: PLoS One. 2024 Oct 17;19(10):e0312147. doi: 10.1371/journal.pone.0312147 (PMC11486373; doi:10.1371/journal.pone.0312147)
Supplement: S5 File — (ZIP) [file pone.0312147.s005.zip › Flow Cytometric Assessment/Global Sheet1_12052022165321.pdf]

FACSDiva Version 6.2

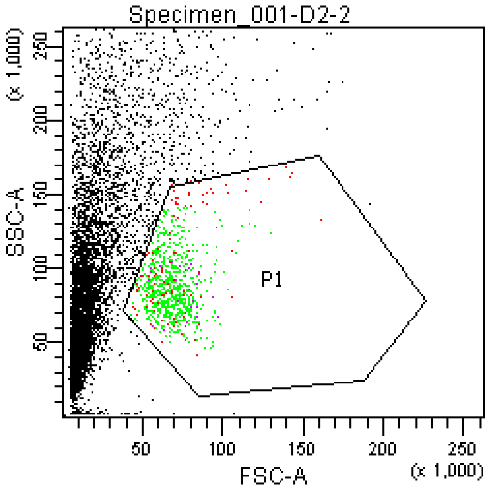

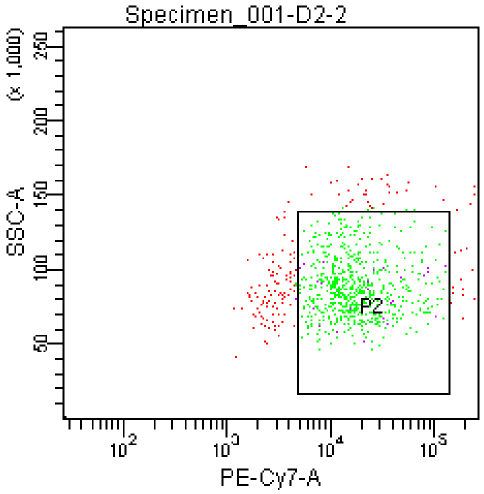

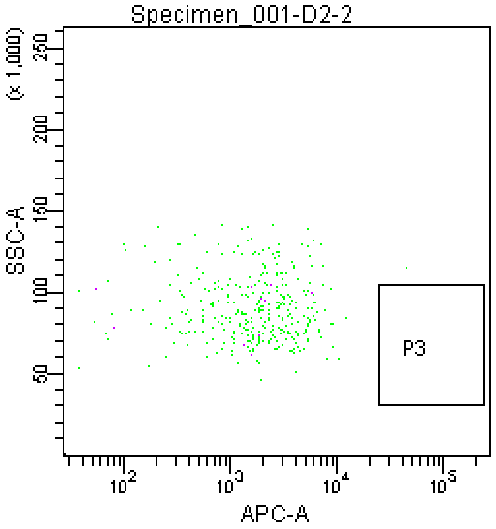

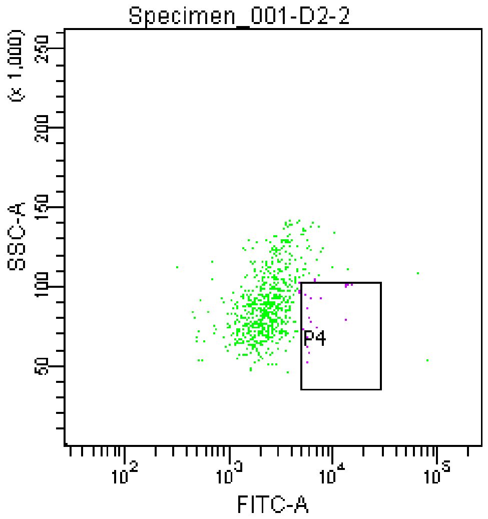

Experiment Name: Experiment_7741
Specimen Name: Specimen_001
Tube Name: D2-2
Record Date: Jan 10, 2022 9:15:04 PM
$OP: Administrator
GUID: 40981318-afaf-4748-b5aa-1b5ebb8fc443

Population	#Events	%Parent	SSC-A Mean	PE-Cy7-A Mean
P1	836	8.4	89,737	25,611
P2	689	82.4	87,270	24,159
P3	0	0.0	####	####
P4	25	3.6	83,234	37,287
