# Supplementary material for: Comparative analysis of the effects of cyclophosphamide and dexamethasone on intestinal immunity and microbiota in delayed hypersensitivity mice
Source: PLoS One. 2024 Oct 17;19(10):e0312147. doi: 10.1371/journal.pone.0312147 (PMC11486373; doi:10.1371/journal.pone.0312147)
Supplement: S5 File — (ZIP) [file pone.0312147.s005.zip › Flow Cytometric Assessment/Global Sheet1_12052022165326.pdf]

FACSDiva Version 6.2

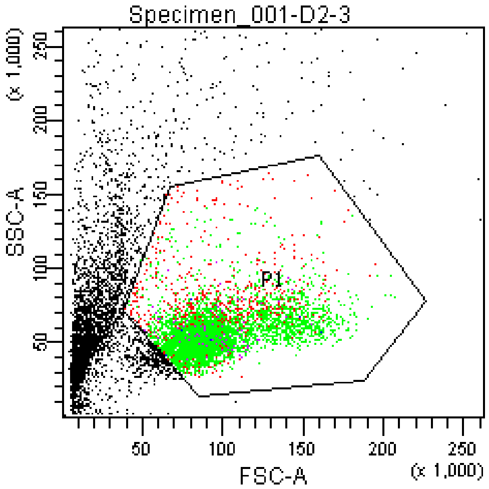

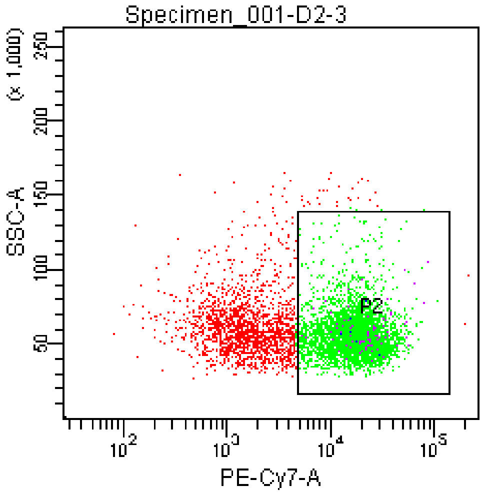

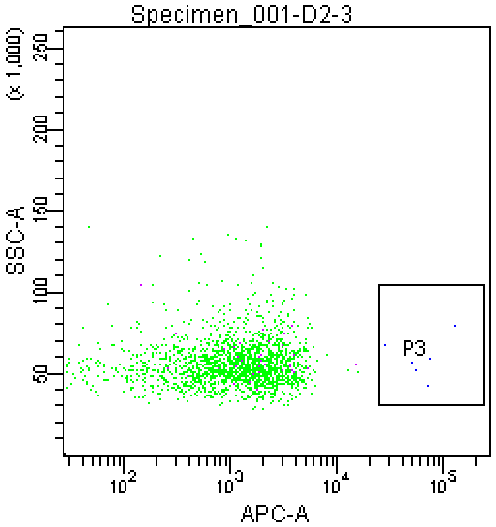

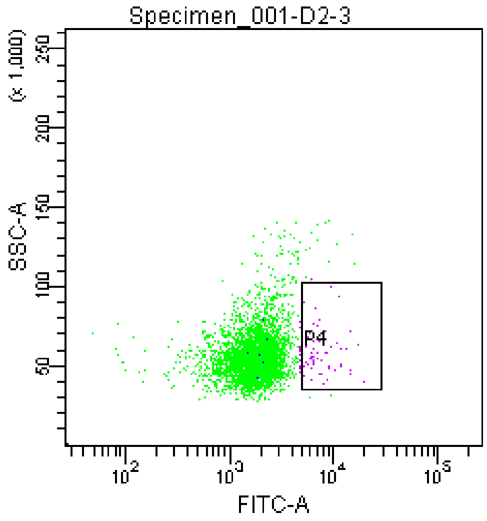

Experiment Name:	Experiment_7741
Specimen Name:	Specimen_001
Tube Name:	D2-3
Record Date:	Jan 10, 2022 9:16:23 PM
$OP:	Administrator
GUID:	b2ac2797-0f04-41c9-b705-76a9c0321fc1

Population	#Events	%Parent	SSC-A Mean	PE-Cy7-A Mean
P1	5,659	56.6	56,988	14,620
P2	3,948	69.8	54,917	19,941
P3	6	0.2	57,688	19,688
P4	76	1.9	57,941	27,602
