# Supplementary material for: Comparative analysis of the effects of cyclophosphamide and dexamethasone on intestinal immunity and microbiota in delayed hypersensitivity mice
Source: PLoS One. 2024 Oct 17;19(10):e0312147. doi: 10.1371/journal.pone.0312147 (PMC11486373; doi:10.1371/journal.pone.0312147)
Supplement: S5 File — (ZIP) [file pone.0312147.s005.zip › Flow Cytometric Assessment/Global Sheet1_12052022165331.pdf]

FACSDiva Version 6.2

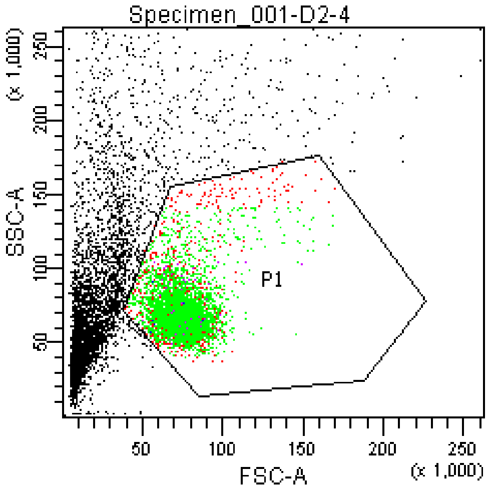

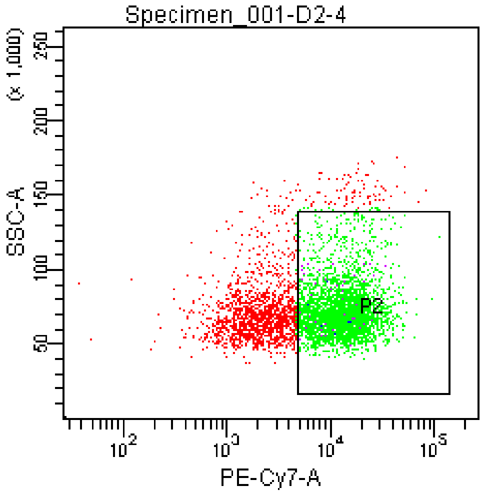

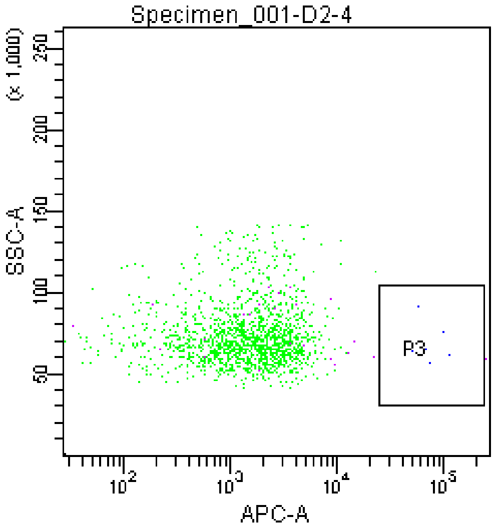

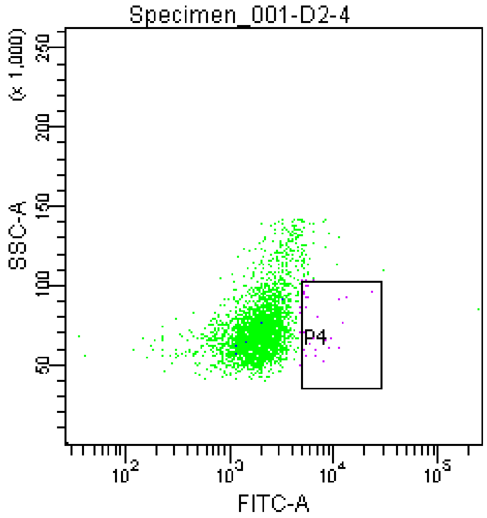

Experiment Name:	Experiment_7741
Specimen Name:	Specimen_001
Tube Name:	D2-4
Record Date:	Jan 10, 2022 9:17:46 PM
$OP:	Administrator
GUID:	c40fd444-dc02-4161-93f1-034fc6a4b164

Population	#Events	%Parent	SSC-A Mean	PE-Cy7-A Mean
P1	4,693	46.9	72,252	11,580
P2	3,169	67.5	71,440	15,515
P3	7	0.2	65,632	10,896
P4	40	1.3	78,759	13,243
