# Supplementary material for: Comparative analysis of the effects of cyclophosphamide and dexamethasone on intestinal immunity and microbiota in delayed hypersensitivity mice
Source: PLoS One. 2024 Oct 17;19(10):e0312147. doi: 10.1371/journal.pone.0312147 (PMC11486373; doi:10.1371/journal.pone.0312147)
Supplement: S5 File — (ZIP) [file pone.0312147.s005.zip › Flow Cytometric Assessment/Global Sheet1_12052022165336.pdf]

FACSDiva Version 6.2

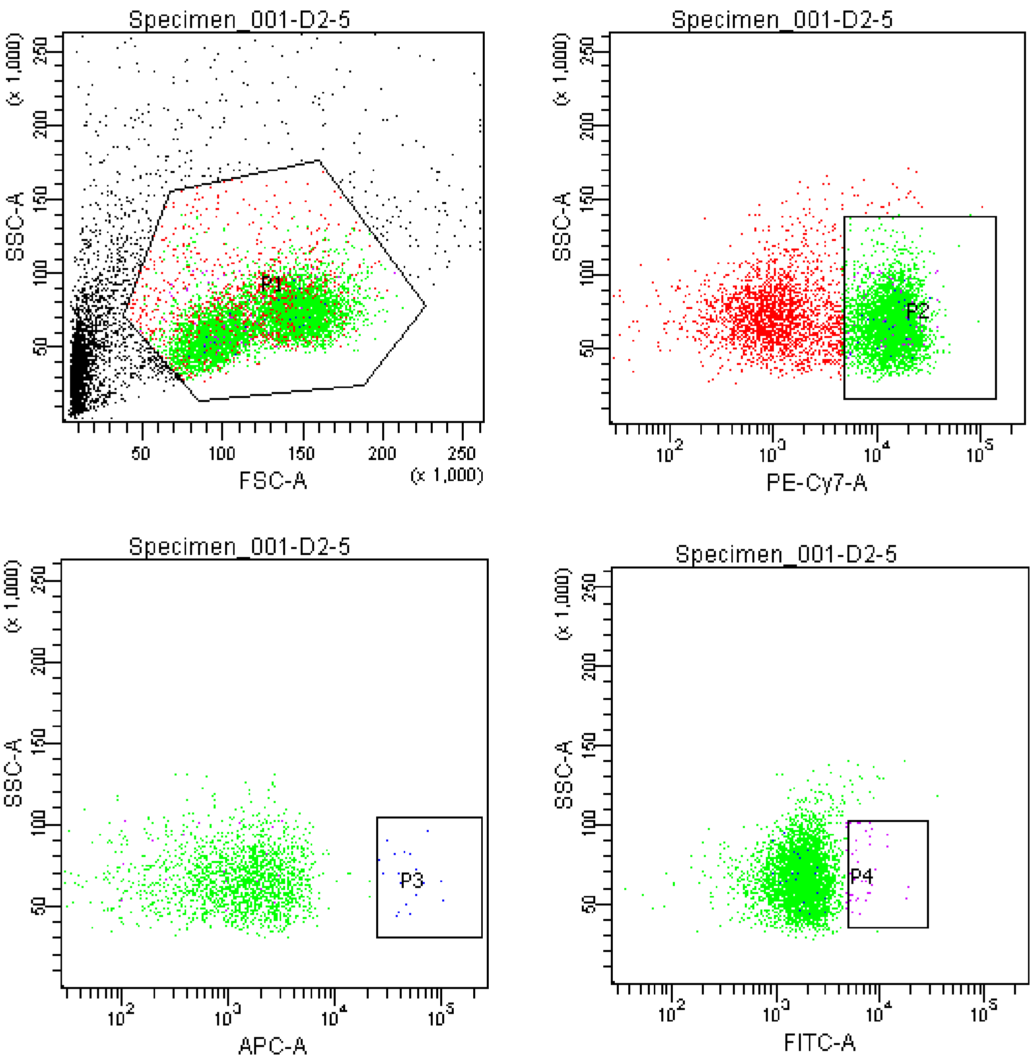

Experiment Name:	Experiment_7741
Specimen Name:	Specimen_001
Tube Name:	D2-5
Record Date:	Jan 10, 2022 9:18:32 PM
$OP:	Administrator
GUID:	b10277f2-235e-4fd2-a535-aff4b9d19dc8

Population	#Events	%Parent	SSC-A Mean	PE-Cy7-A Mean
P1	6,780	67.8	67,761	10,754
P2	4,409	65.0	65,369	15,699
P3	20	0.5	65,909	15,783
P4	48	1.1	72,315	17,616
