# Supplementary material for: Comparative analysis of the effects of cyclophosphamide and dexamethasone on intestinal immunity and microbiota in delayed hypersensitivity mice
Source: PLoS One. 2024 Oct 17;19(10):e0312147. doi: 10.1371/journal.pone.0312147 (PMC11486373; doi:10.1371/journal.pone.0312147)
Supplement: S5 File — (ZIP) [file pone.0312147.s005.zip › Flow Cytometric Assessment/Global Sheet1_12052022165340.pdf]

FACSDiva Version 6.2

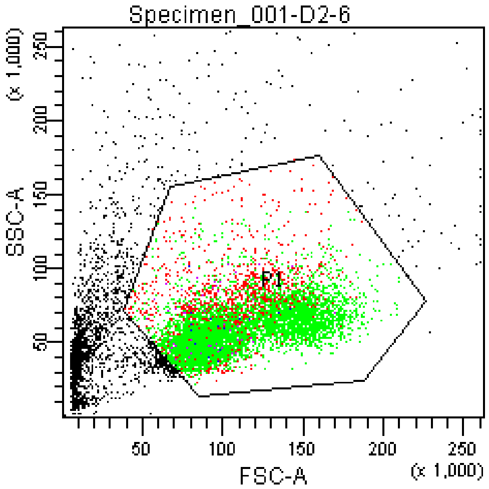

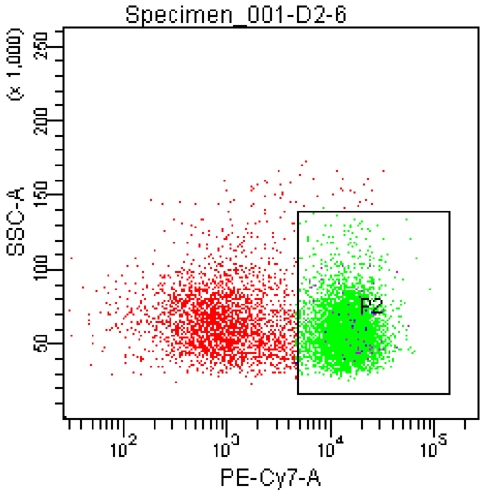

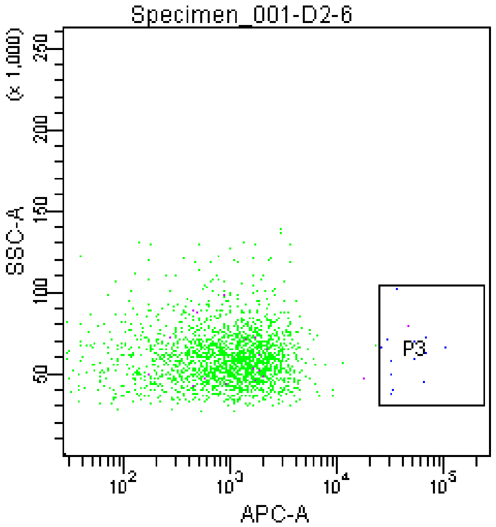

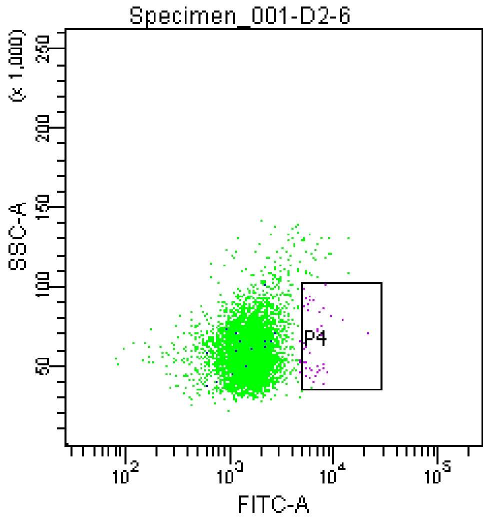

Experiment Name:	Experiment_7741
Specimen Name:	Specimen_001
Tube Name:	D2-6
Record Date:	Jan 10, 2022 9:19:08 PM
$OP:	Administrator
GUID:	9fe18e17-4229-4861-8070-b4186f644615

Population	#Events	%Parent	SSC-A Mean	PE-Cy7-A Mean
P1	7,703	77.0	60,674	11,473
P2	5,169	67.1	57,977	16,441
P3	16	0.3	61,169	17,533
P4	52	1.0	63,890	20,633
