# Supplementary material for: Comparative analysis of the effects of cyclophosphamide and dexamethasone on intestinal immunity and microbiota in delayed hypersensitivity mice
Source: PLoS One. 2024 Oct 17;19(10):e0312147. doi: 10.1371/journal.pone.0312147 (PMC11486373; doi:10.1371/journal.pone.0312147)
Supplement: S5 File — (ZIP) [file pone.0312147.s005.zip › Flow Cytometric Assessment/Global Sheet1_12052022165345.pdf]

FACSDiva Version 6.2

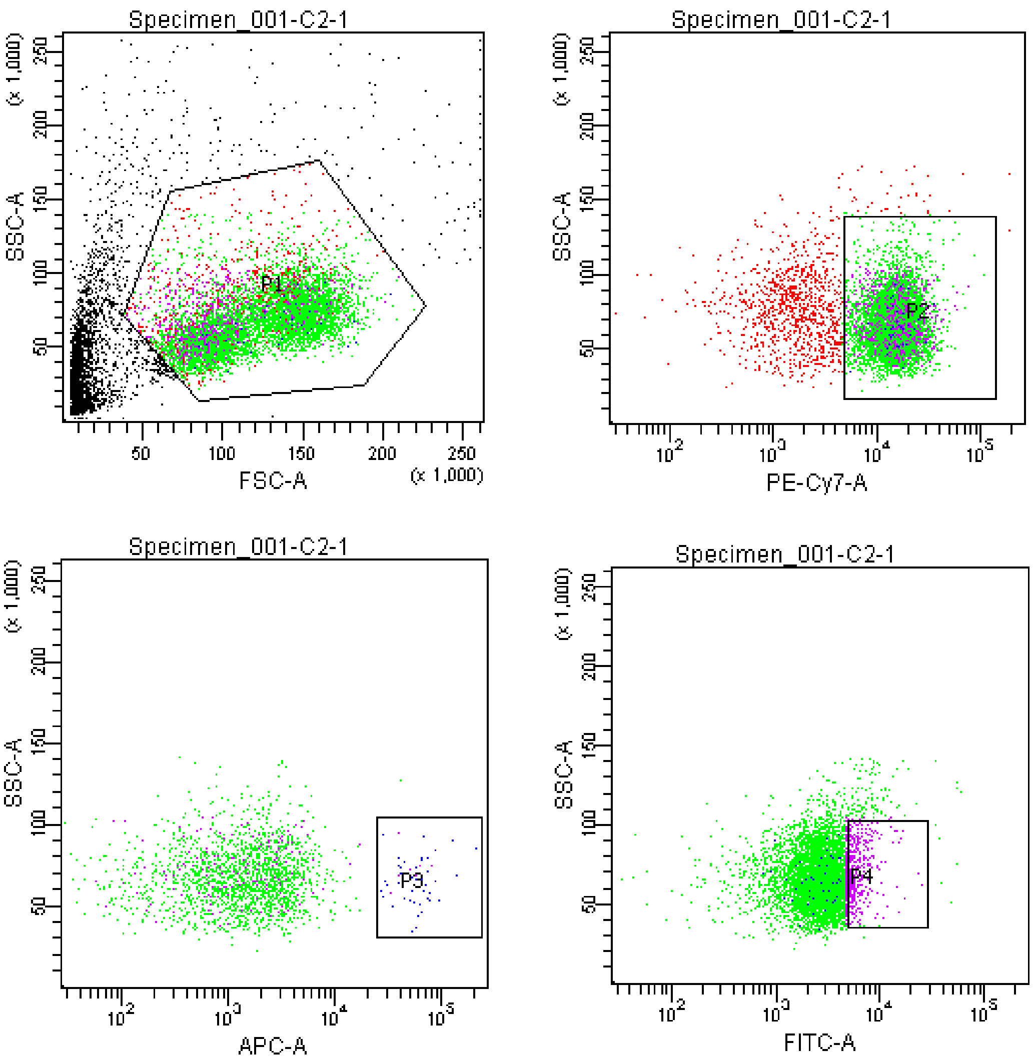

Experiment Name:	Experiment_7741
Specimen Name:	Specimen_001
Tube Name:	C2-1
Record Date:	Jan 10, 2022 9:19:44 PM
$OP:	Administrator
GUID:	aab95080-9c62-4e94-b8c4-1497181cde83

Population	#Events	%Parent	SSC-A Mean	PE-Cy7-A Mean
P1	6,905	69.0	67,498	14,754
P2	5,849	84.7	65,768	16,912
P3	52	0.9	64,372	17,115
P4	504	8.6	68,953	17,691
