# Supplementary material for: Comparative analysis of the effects of cyclophosphamide and dexamethasone on intestinal immunity and microbiota in delayed hypersensitivity mice
Source: PLoS One. 2024 Oct 17;19(10):e0312147. doi: 10.1371/journal.pone.0312147 (PMC11486373; doi:10.1371/journal.pone.0312147)
Supplement: S5 File — (ZIP) [file pone.0312147.s005.zip › Flow Cytometric Assessment/Global Sheet1_12052022165350.pdf]

FACSDiva Version 6.2

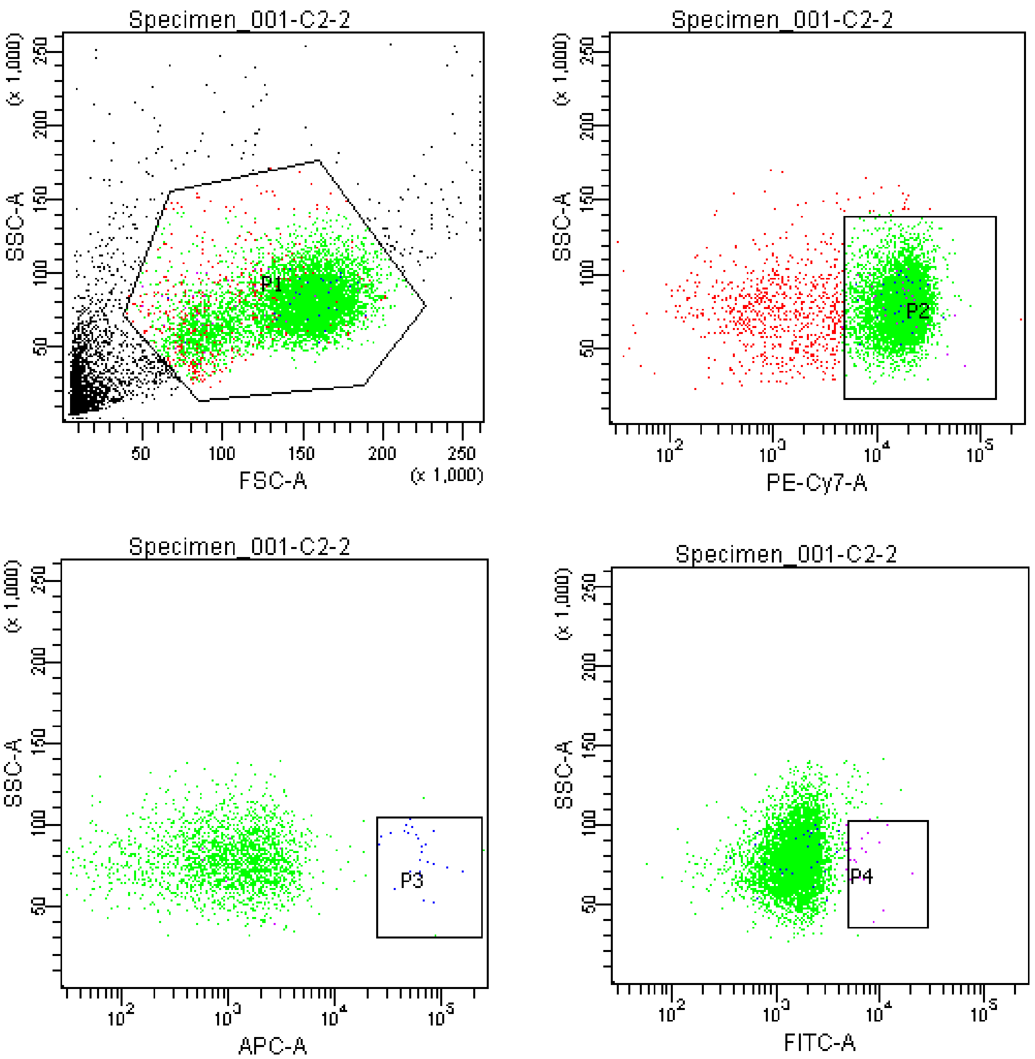

Experiment Name:	Experiment_7741
Specimen Name:	Specimen_001
Tube Name:	C2-2
Record Date:	Jan 10, 2022 9:20:22 PM
$OP:	Administrator
GUID:	212e5f14-93b7-40fd-a31c-1b25bb966f18

Population	#Events	%Parent	SSC-A Mean	PE-Cy7-A Mean
P1	7,293	72.9	77,439	17,202
P2	6,480	88.9	77,625	19,090
P3	27	0.4	80,641	18,465
P4	27	0.4	77,111	20,966
