# Supplementary material for: Comparative analysis of the effects of cyclophosphamide and dexamethasone on intestinal immunity and microbiota in delayed hypersensitivity mice
Source: PLoS One. 2024 Oct 17;19(10):e0312147. doi: 10.1371/journal.pone.0312147 (PMC11486373; doi:10.1371/journal.pone.0312147)
Supplement: S5 File — (ZIP) [file pone.0312147.s005.zip › Flow Cytometric Assessment/Global Sheet1_12052022165356.pdf]

FACSDiva Version 6.2

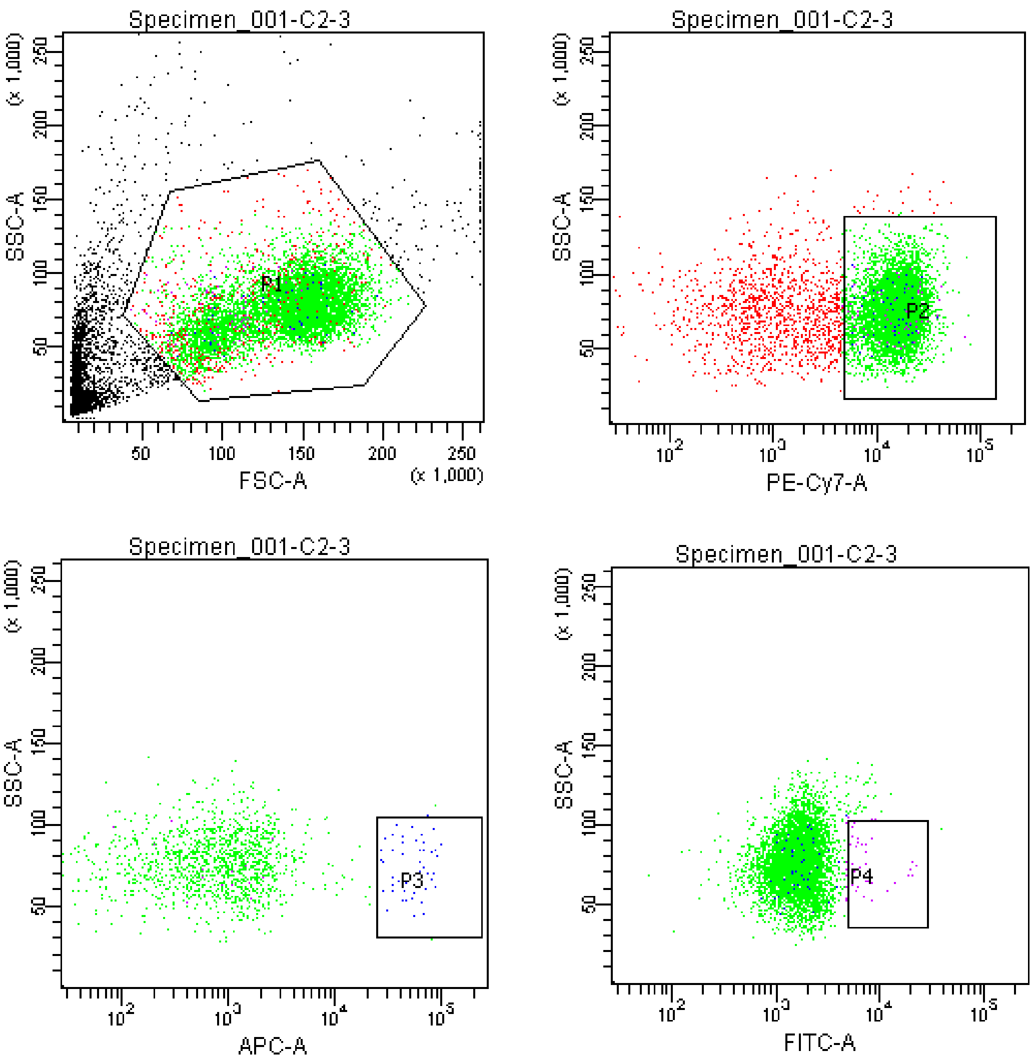

Experiment Name:	Experiment_7741
Specimen Name:	Specimen_001
Tube Name:	C2-3
Record Date:	Jan 10, 2022 9:20:46 PM
$OP:	Administrator
GUID:	15c51e2e-983d-4935-9faf-662e0693518e

Population	#Events	%Parent	SSC-A Mean	PE-Cy7-A Mean
P1	7,237	72.4	74,458	14,891
P2	5,986	82.7	74,568	17,621
P3	48	0.8	72,102	15,319
P4	54	0.9	73,520	18,531
