# Supplementary material for: Comparative analysis of the effects of cyclophosphamide and dexamethasone on intestinal immunity and microbiota in delayed hypersensitivity mice
Source: PLoS One. 2024 Oct 17;19(10):e0312147. doi: 10.1371/journal.pone.0312147 (PMC11486373; doi:10.1371/journal.pone.0312147)
Supplement: S5 File — (ZIP) [file pone.0312147.s005.zip › Flow Cytometric Assessment/Global Sheet1_12052022165400.pdf]

FACSDiva Version 6.2

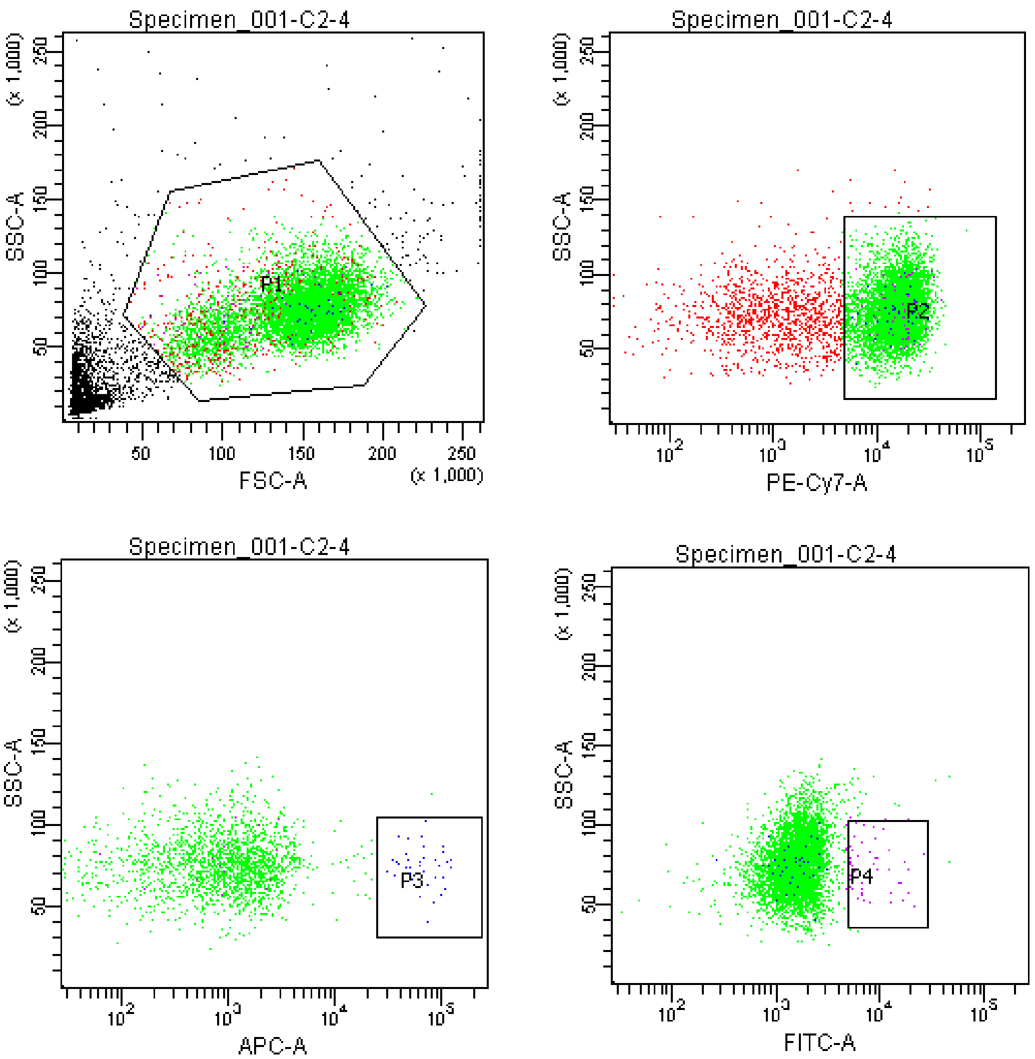

Experiment Name:	Experiment_7741
Specimen Name:	Specimen_001
Tube Name:	C2-4
Record Date:	Jan 10, 2022 9:21:14 PM
$OP:	Administrator
GUID:	4ca57b8c-edec-4c5b-9e92-ba0388141dae

Population	#Events	%Parent	SSC-A Mean	PE-Cy7-A Mean
P1	7,472	74.7	73,478	14,910
P2	6,233	83.4	73,922	17,553
P3	37	0.6	72,397	17,525
P4	55	0.9	74,279	18,767
