# Supplementary material for: Comparative analysis of the effects of cyclophosphamide and dexamethasone on intestinal immunity and microbiota in delayed hypersensitivity mice
Source: PLoS One. 2024 Oct 17;19(10):e0312147. doi: 10.1371/journal.pone.0312147 (PMC11486373; doi:10.1371/journal.pone.0312147)
Supplement: S5 File — (ZIP) [file pone.0312147.s005.zip › Flow Cytometric Assessment/Global Sheet1_12052022165404.pdf]

# FACSDiva Version 6.2

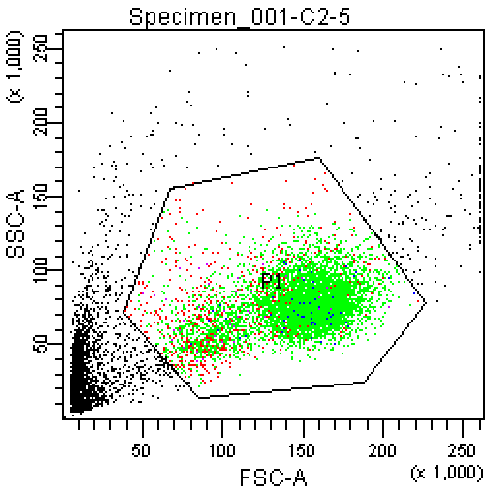


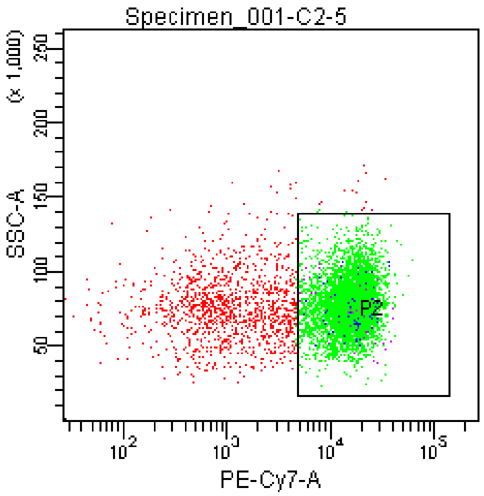


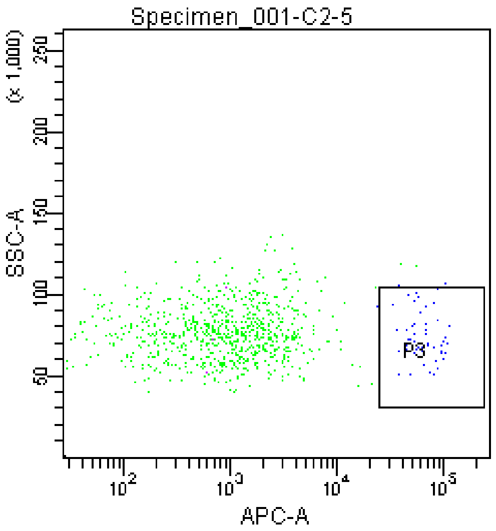


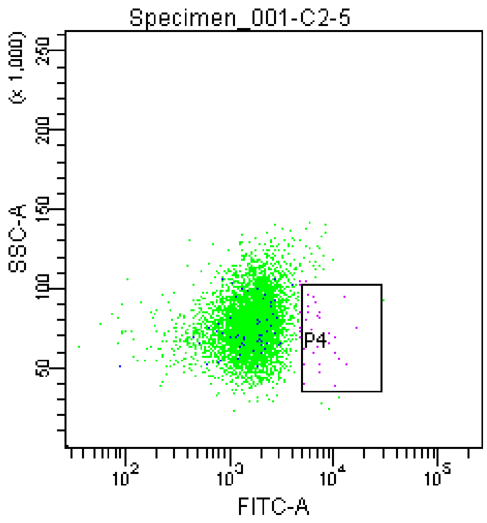


| | |
|---|---|
| Experiment Name: | Experiment_7741 |
| Specimen Name: | Specimen_001 |
| Tube Name: | C2-5 |
| Record Date: | Jan 10, 2022 9:21:42 PM |
| $OP: | Administrator |
| GUID: | 3a0d056d-d04e-46b4-ba30-0fcd90626cd8 |

| Population | #Events | %Parent | SSC-A Mean | PE-Cy7-A Mean |
|---|---|---|---|---|
| P1 | 7,150 | 71.5 | 75,447 | 14,453 |
| P2 | 5,818 | 81.4 | 75,617 | 17,384 |
| P3 | 57 | 1.0 | 72,896 | 18,340 |
| P4 | 38 | 0.7 | 71,515 | 19,583 |
