# Supplementary material for: Comparative analysis of the effects of cyclophosphamide and dexamethasone on intestinal immunity and microbiota in delayed hypersensitivity mice
Source: PLoS One. 2024 Oct 17;19(10):e0312147. doi: 10.1371/journal.pone.0312147 (PMC11486373; doi:10.1371/journal.pone.0312147)
Supplement: S5 File — (ZIP) [file pone.0312147.s005.zip › Flow Cytometric Assessment/Global Sheet1_12052022165409.pdf]

FACSDiva Version 6.2

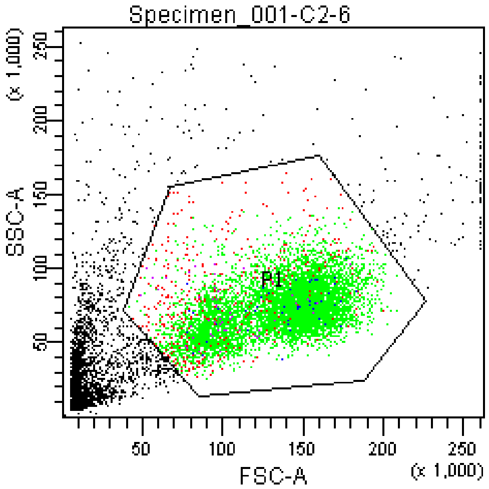

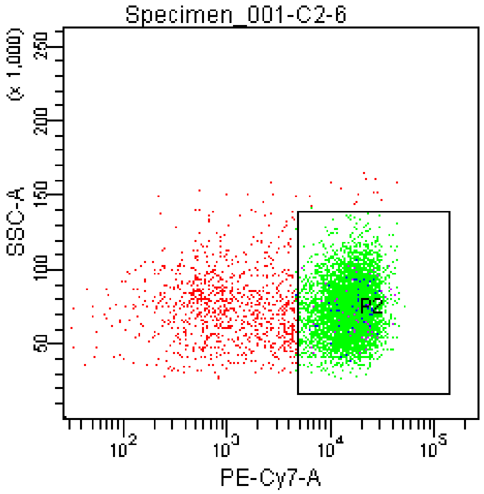

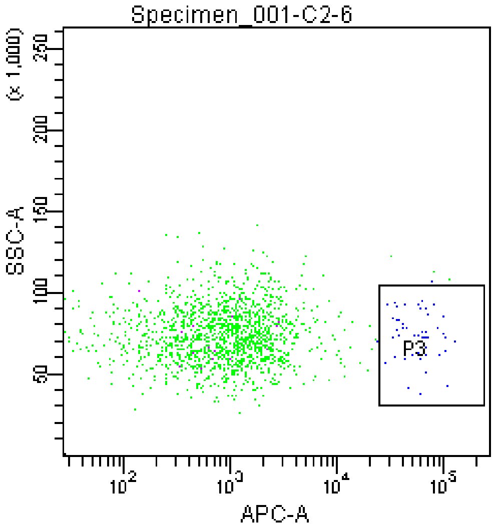

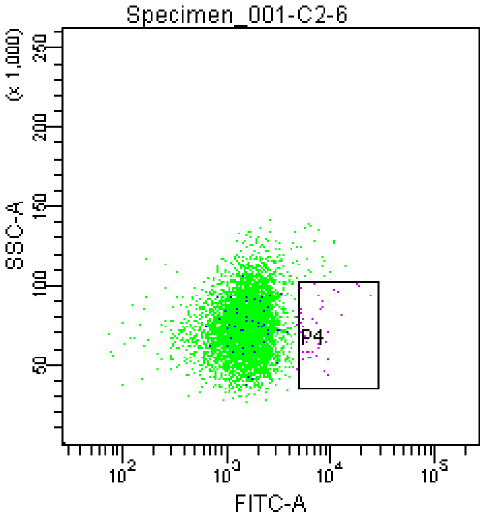

Experiment Name: Experiment_7741
Specimen Name: Specimen_001
Tube Name: C2-6
Record Date: Jan 10, 2022 9:22:14 PM
$OP: Administrator
GUID: 65aa14b7-65c6-4281-ad76-a5fcceddb5ac

Population	#Events	%Parent	SSC-A Mean	PE-Cy7-A Mean
P1	7,225	72.2	72,577	15,418
P2	6,244	86.4	72,270	17,568
P3	48	0.8	72,532	18,759
P4	50	0.8	71,179	20,666
