# Supplementary material for: Comparative analysis of the effects of cyclophosphamide and dexamethasone on intestinal immunity and microbiota in delayed hypersensitivity mice
Source: PLoS One. 2024 Oct 17;19(10):e0312147. doi: 10.1371/journal.pone.0312147 (PMC11486373; doi:10.1371/journal.pone.0312147)
Supplement: S5 File — (ZIP) [file pone.0312147.s005.zip › Flow Cytometric Assessment/Global Sheet1_12052022165414.pdf]

FACSDiva Version 6.2

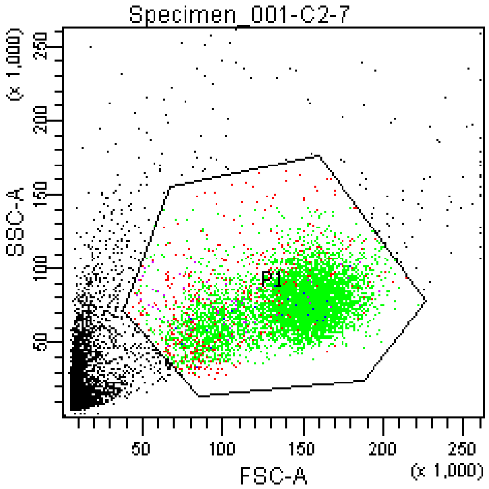

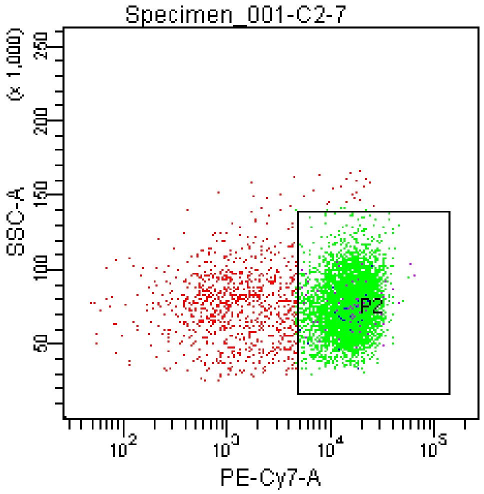

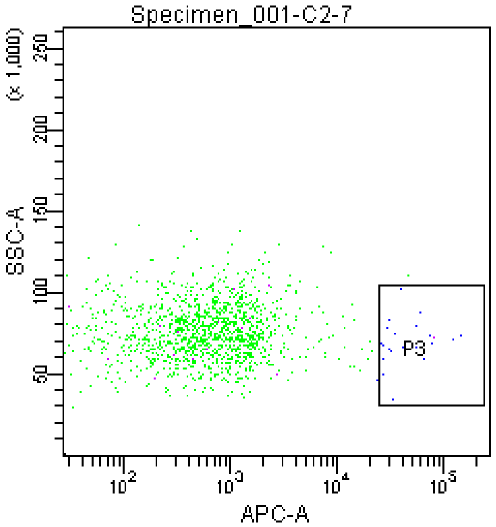

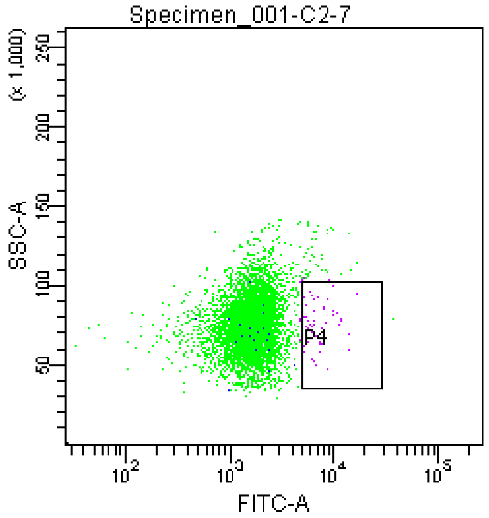

Experiment Name:	Experiment_7741
Specimen Name:	Specimen_001
Tube Name:	C2-7
Record Date:	Jan 10, 2022 9:22:51 PM
$OP:	Administrator
GUID:	27540182-5c59-4933-90e6-58d96fe64325

Population	#Events	%Parent	SSC-A Mean	PE-Cy7-A Mean
P1	6,534	65.3	74,109	15,003
P2	5,573	85.3	73,868	17,256
P3	22	0.4	66,995	15,927
P4	59	1.1	75,137	19,168
