# Supplementary material for: Comparative analysis of the effects of cyclophosphamide and dexamethasone on intestinal immunity and microbiota in delayed hypersensitivity mice
Source: PLoS One. 2024 Oct 17;19(10):e0312147. doi: 10.1371/journal.pone.0312147 (PMC11486373; doi:10.1371/journal.pone.0312147)
Supplement: Attachment — Submitted filename: Reply letter.docx [file pone.0312147.s008.docx]

Journal Requirements:

When submitting your revision, we need you to address these additional requirements.

1. Please ensure that your manuscript meets PLOS ONE's style requirements, including those for file naming. The PLOS ONE style templates can be found at

https://journals.plos.org/plosone/s/file?id=wjVg/PLOSOne_formatting_sample_main_body.pdf and https://journals.plos.org/plosone/s/file?id=ba62/PLOSOne_formatting_sample_title_authors_affiliations.pdf

Reply to Editor: Thank you for your guidance. We have reviewed and updated our manuscript to comply with PLOS ONE's style requirements, including adhering to the file naming conventions outlined in the provided templates.

2. To comply with PLOS ONE submissions requirements, in your Methods section, please provide additional information regarding the experiments involving animals and ensure you have included details on (1) methods of sacrifice, (2) methods of anesthesia and/or analgesia, and (3) efforts to alleviate suffering.

Reply to Editor: Thank you for your feedback. We have revised our Methods section to include comprehensive details regarding the animal experiments. Specifically, we have now provided explicit information on:

“Animals and Ethics

Female BALB/c mice, 6 weeks of age and weighing 20 ± 2 g, were obtained from the Guangdong Medical Experiment Animal Center, Guangzhou, China. They were housed in a specific pathogen-free (SPF) environment with a temperature range of 20~26 °C and were provided with food and water ad libitum. This study was carried out in strict accordance with the recommendations in the Guide for the Care and Use of Laboratory Animals of the National Institutes of Health. The protocol was approved by the Laboratory Animal Welfare and Ethics Committee of Guangxi University of Chinese Medicine (Approval No. DW20211205-101). All surgery was performed under sodium pentobarbital anesthesia, and all efforts were made to minimize suffering.

In instances where mice experienced a weight loss exceeding 20%, they were humanely euthanized with pentobarbital sodium at a dosage of 150 mg/kg body weight, administered intraperitoneally. This method ensures the maintenance of high ethical standards throughout the study.”

We have ensured that all aspects of animal care and experimentation are in full compliance with PLOS ONE's submission requirements.

3. Thank you for stating the following in the Acknowledgments Section of your manuscript: "This study was supported by Guangxi University of Chinese Medicine Doctoral Program (2020BS011 and 2021BS019), Guangxi Key Laboratory of Chinese Medicine Foundation Research Projects (22-065-53-02), Projects to improve the basic scientific research capacity of young and middle-aged people (2020KY07002) and Guangxi first-class discipline Clinical basis of Traditional Chinese Medicine (TCM) (0502300304)."

We note that you have provided funding information that is not currently declared in your Funding Statement. However, funding information should not appear in the Acknowledgments section or other areas of your manuscript. We will only publish funding information present in the Funding Statement section of the online submission form.

Please remove any funding-related text from the manuscript and let us know how you would like to update your Funding Statement. Currently, your Funding Statement reads as follows:

"This study was supported by Guangxi University of Chinese Medicine Doctoral Program（2020BS01 and 2021BS019), Guangxi Key Laboratory of Chinese Medicine Foundation Research Projects (22-065-53-02), Projects to improve the basic scientific research capacity of young and middle-aged people (2020KY07002) and Guangxi first-class discipline Clinical basis of Traditional Chinese Medicine (TCM) (0502300304). The funder were Guangxi University of Chinese Medicine and Department of Education of Guangxi. The funders did not play any role in the study design, data collection and analysis, decision to publish, or preparation of the manuscript."

Please include your amended statements within your cover letter; we will change the online submission form on your behalf.

Reply to Editor: Thank you for your instructions regarding the declaration of funding information in our manuscript. We have removed all references to funding sources from the Acknowledgments section of our manuscript as requested.

We would like to update our Funding Statement as follows:

This study was supported by Guangxi University of Chinese Medicine Doctoral Program (2020BS011 and 2021BS019), Guangxi Key Laboratory of Chinese Medicine Foundation Research Projects (22-065-53-02), Projects to improve the basic scientific research capacity of young and middle-aged people (2020KY07002) and Guangxi first-class discipline Clinical basis of Traditional Chinese Medicine (TCM) (0502300304). The funders had no role in study design, data collection and analysis, decision to publish, or preparation of the manuscript."

4. We note that your Data Availability Statement is currently as follows: All relevant data are within the manuscript and its Supporting Information files.

Please confirm at this time whether or not your submission contains all raw data required to replicate the results of your study. Authors must share the “minimal data set” for their submission. PLOS defines the minimal data set to consist of the data required to replicate all study findings reported in the article, as well as related metadata and methods (https://journals.plos.org/plosone/s/data-availability#loc-minimal-data-set-definition).

For example, authors should submit the following data:

- The values behind the means, standard deviations and other measures reported;

- The values used to build graphs;

- The points extracted from images for analysis.

Authors do not need to submit their entire data set if only a portion of the data was used in the reported study.

If your submission does not contain these data, please either upload them as Supporting Information files or deposit them to a stable, public repository and provide us with the relevant URLs, DOIs, or accession numbers. For a list of recommended repositories, please see https://journals.plos.org/plosone/s/recommended-repositories.

If there are ethical or legal restrictions on sharing a de-identified data set, please explain them in detail (e.g., data contain potentially sensitive information, data are owned by a third-party organization, etc.) and who has imposed them (e.g., an ethics committee). Please also provide contact information for a data access committee, ethics committee, or other institutional body to which data requests may be sent. If data are owned by a third party, please indicate how others may request data access.

Reply to Editor: Thank you for your request for clarification regarding the Data Availability Statement for our submission.

We confirm that our submission includes all raw data required to replicate the results of our study, as defined by PLOS ONE's guidelines for a minimal data set. Specifically, our submission contains:

- The underlying numerical values for all reported means, standard deviations, and other statistical measures;

- The data points utilized in the construction of all graphs presented in the study;

- The extracted data points derived from images used for analysis within the manuscript.

We have ensured that the data provided are sufficient to allow for the complete replication of our study's findings, adhering to the principles of transparency and reproducibility. The data are included within the manuscript and its Supporting Information files, and we have taken care to organize and present them in a manner that facilitates understanding and reuse.

Please do not hesitate to contact us if further clarification or additional information is needed.

[Note: HTML markup is below. Please do not edit.]

5 Review Comments to the Author

Please use the space provided to explain your answers to the questions above. You may also include additional comments for the author, including concerns about dual publication, research ethics, or publication ethics. (Please upload your review as an attachment if it exceeds 20,000 characters)

Reply to Editor: We would like to express our gratitude for the time and thorough review you and the reviewers have dedicated to our study. We have taken the reviewers' suggestions seriously and have made the following revisions to our manuscript:

1 Revision of Data and Analysis: Based on the reviewers' feedback, we have re-analyzed the data and provided a more detailed explanation and supporting data in the Results and Discussion sections.

2 Update to Methods: We have updated the Methods section to more clearly describe the experimental design, animal housing conditions, and sample collection process.

Precision in Conclusions: We have re-evaluated and revised the Conclusion section to ensure that the statements are accurate, data-supported, and avoid any ambiguous comparisons.

3 Figures and Statistical Analysis: We have added figures to visually present key results and conducted appropriate statistical tests to ensure the scientific and reliable nature of our findings.

4 Depth in Discussion: We have expanded the Discussion section to include a more in-depth analysis of the significance of our results and their contributions to the existing literature and future research directions.

5 Ethical Standards Compliance: We confirm that all research work was conducted in strict accordance with ethical standards, including animal welfare and research ethics.

We believe that these revisions have strengthened our study and provided valuable insights to the academic community. We look forward to your further guidance and feedback.

Reviewer #1: There are some issues with this manuscript that require further revisions.

1. It is better if a normal group was designed which does not undergo sensitization treatment. the control group is the model group according to the description in section

Reply to reviewer: First and foremost, we would like to express our sincere gratitude for your insightful comments and suggestions. We understand your concern regarding the inclusion of a normal control group in our experimental design. Allow us to clarify our approach and the rationale behind it.

Reasons for the Experimental Design:

1. Intra-individual Control Design: In our study, the right ear of each mouse was challenged with DNFB to induce the DTH response, while the left ear served as its own control by receiving only acetone. This design allows for a direct comparison of the DTH response between the treated right ear and the untreated left ear within the same animal, minimizing inter-individual variability.

2. Model Validation: The success of the DTH model is evidenced by the expected increase in swelling of the right ear post-challenge, contrasted with the absence of such a response in the left ear. This intra-individual difference provides a robust internal control to assess the effects of CY and DEX treatments.

3. Support from Scientific Literature: The use of an intra-individual control is a common practice in DTH research and is widely recognized and utilized in the scientific community.

4. Statistical Power: By employing an intra-individual control design, we enhance the sensitivity of our statistical analysis by reducing experimental error and increasing the likelihood of detecting treatment effects.

Response to Your Comments:

While we acknowledge the value of an independent normal control group in certain contexts, our study design, which employs an intra-individual control, was chosen for ethical considerations and efficient use of resources. This design not only aligns with the 3Rs principles (replacement, reduction, refinement) but also provides a powerful method for assessing the impact of CY and DEX on the DTH response by directly comparing the two ears of the same mouse.

We believe that by clearly describing this design in the methods section and thoroughly analyzing its advantages and limitations in the results and discussion sections, our study can offer valuable insights to the scientific community.

We appreciate the opportunity to further elucidate our experimental design and look forward to any additional guidance and feedback you may provide.

2. It is described that the effect of Cy performed better than DEX but there is no difference comparison between the Cy and DEX groups in results. It should be recommende to supplement.

Reply to reviewer: Thank you for your astute observation regarding the comparison between the effects of Cyclophosphamide (CY) and Dexamethasone (DEX) on the immune function of DNFB-induced sensitized mice. We have conducted a thorough reanalysis of our results and would like to provide the following clarification and additional comparative data:

Upon reevaluation, we identified significant differences in the immunosuppressive effects of CY and DEX. As shown in Figure 1, both CY and DEX significantly reduced the thymus and spleen indices, mesenteric lymph node enlargement, intestinal sIgA content, ear swelling degree, and the number of intestinal Peyer's patches compared to the control group. However, CY demonstrated a more pronounced reduction in these indices, suggesting a stronger immunosuppressive potential.

In particular, the CY group showed a significantly increased percentage of CD3+ total T cells and a non-significant effect on CD3+CD69+ activated T cells, whereas DEX increased the percentage of CD3+CD4+ helper T cells (Fig 3 and 4). CY treatment also resulted in a significant reduction in the percentage of CD3+TCRγ/δ+ T cells compared to DEX, which showed an opposite trend (Fig 7). These findings indicate that CY and DEX differentially modulate T cell subsets, with CY having a broader immunosuppressive effect.

Furthermore, our analysis of intestinal flora diversity and structure revealed that CY increased α diversity, while DEX decreased it (Fig 8B and 8C). At the genus level, CY significantly altered the composition of the gut microbiota, particularly increasing the proportion of Lachnospiraceae and decreasing Lactobacillus, which may be linked to its immunomodulatory effects (Fig 9B).

We have now included these comparative data and discussion in the revised manuscript to directly address the differences between CY and DEX, providing a clearer understanding of their comparative immunosuppressive effects.

We appreciate the opportunity to enhance our manuscript with this additional analysis and believe these revisions will significantly improve the clarity and impact of our findings.

3. Experimental procedures

It is necessary to explain clearly the administration method and time of Cy and DEX.

In addition, the time for receiving DNFB treatment also needs to be clearly explained, 12 hours, 24 hours, or 48 hours?

Reply to reviewer: Thank you for your request for additional clarification on the administration methods and timing of Cyclophosphamide (CY) and Dexamethasone (DEX), as well as the specifics of the DNFB treatment in our study.

In response to your query, we have made the following revisions to the methods section of our manuscript:

Administration of CY and DEX: Both CY and DEX were administered intraperitoneally. The administration took place on Day 4, 48 hours after the initial sensitization with DNFB. The dosages used were 100 mg/kg for CY and 40 mg/kg for DEX.

DNFB Treatment Timing: The challenge phase involved applying DNFB to the right ear on Day 6. The left ear received an equivalent volume of the vehicle (acetone) as a control. The ear swelling was measured 24 hours post-challenge, which corresponds to Day 7.

We believe these revisions provide the necessary details to understand the timing and method of drug administration and DNFB treatment in our study.

We appreciate your guidance and have made the necessary revisions to ensure the accuracy and clarity of our methods.

4. Results

①Table1 Missing unit. I think PP number and ear swelling are not suitable to appear here.

Reply to reviewer: Thank you for your comment on the presentation of our results in Table 1. We have taken your feedback into account and have converted Table 1 into a figure (Fig 1) to enhance clarity and visual representation of the data.

Fig 1 CY and DEX significantly inhibited the immune function of DNFB induced sensitized mice. (A-B) Thymus index and spleen index of mice in Contorl, CY and DEX groups. (C) Structural diagram of thymus and MLNs of mice in Contorl, CY and DEX groups. (D) Intestinal sIgA content of mice in Contorl group, CY group and DEX group. (E) Degree of ear swelling in mice in Contorl group, CY group and DEX group. (F) HE staining was used to detect the degree of infiltration of ear inflammatory cells in mice. (G) The number of intestinal PPs in mice in Contorl, CY and DEX groups. (H) HE staining was used to detect the degree of infiltration of intestinal PPs inflammatory cells in mice. Compared to the Control group, **P<0.01, ***P<0.001, Compared to the DEX group, ##P<0.01.

Regarding the inclusion of the PP number and ear swelling measurements, we respectfully disagree with the notion that they are not suitable for this section. We have retained these parameters for the following reasons:

1 PP Number: The number of Peyer's Patches (PPs) is a critical indicator of changes in the gut-associated lymphoid tissue, which is a significant aspect of the immune response. It provides valuable insights into the immunomodulatory effects of CY and DEX on the mucosal immune system.

2 Ear Swelling: Ear swelling is a direct and immediate measure of the delayed-type hypersensitivity (DTH) response, which is central to our study's focus on the immunosuppressive effects of CY and DEX. This parameter is essential for demonstrating the local inflammatory response and its modulation by the drugs.

We have ensured that the figure now includes the appropriate units for all measurements, and we believe that the inclusion of these parameters is justified and contributes to the comprehensiveness of our study.

We hope this addresses your concerns and that our revisions meet the journal's standards.

②Ear swelling

Figure1 lacks scale bar and requires more accurate arrow annotations or higher quality images. The arrow in Figure1B does not show inflammatory cells. In addition, provide a more detailed description of pathological changes

“Lymphocytes are the basis of the immune response, and the number of lymphocytes is an important index of immune function.” I think this description doesn’t appropriate here.

Reply to reviewer: Thank you for your detailed feedback on our figures. We have made the following revisions in response to your comments:

1 Scale Bar Addition: We have now included a scale bar in Figure 1 to provide a reference for the magnification used in the images.

2 Arrow Annotations: We have added clearer arrow annotations in Figure 1 to accurately indicate the areas of inflammatory cell infiltration.

3 Image Quality: We have replaced the images with higher quality versions to ensure that the details are more discernible.

4 Pathological Description: We have provided a more detailed description of the pathological changes observed in the ear tissue sections to better reflect the immunosuppressive effects of CY and DEX.

“CY and DEX significantly inhibited the immune function of DNFB-induced sensitized mice

To evaluate the specific effects of CY and DEX on immune function in DNFB-induced sensitized mice. The indexes of thymus and spleen index, mesenteric lymph node enlargement degree, intestinal sIgA content, ear swelling degree, and intestinal Peyer's patches were determined to determine the immunomodulatory effects of the two drugs and their potential comparative advantages. The results showed that both CY and DEX could effectively inhibit the immune response of DNFB-induced sensitized mice. Specifically, compared with the control group, the thymus and spleen indexes and the degree of mesenteric lymph node enlargement of mice treated with CY and DEX were significantly reduced (Fig 1A-C), indicating that both drugs had a significant shrinking effect on the volume of immune organs. The reduction of intestinal IgA content (Fig 1D) further confirms the inhibitory effect of CY and DEX on the immune response. In addition, a significant reduction in the degree of ear swelling (Fig 1E) and in the infiltration of ear inflammatory cells (Fig 1F) also demonstrated the effective control of local immune responses by CY and DEX. In terms of the number of intestinal Peyer's patches and the infiltration of inflammatory cells, CY and DEX treated mice also showed a decreasing and alleviating trend (Fig 1G-H). Of particular note, the CY group showed a more significant effect in suppressing immune function when compared to DEX, which may indicate that CY has a stronger immunosuppressive potential”

5 Removal of Inappropriate Description: The sentence "Lymphocytes are the basis of the immune response, and the number of lymphocytes is an important index of immune function." has been removed from the figure caption as it was deemed inappropriate for the context.I deleted “Lymphocytes are the basis of the immune response, and the number of lymphocytes is an important index of immune function.”in the paper.

③Figure2: The problems of Figure 2 are similar to Figure 1.

Reply to reviewer: Thank you for your feedback on Figure 2 of our manuscript. We have taken your comments seriously and have made comprehensive revisions to address the concerns raised. Specifically, we have:

1 Re-evaluated the content of Figure 2 and decided to integrate the relevant data into Figure 1 for a more coherent presentation.

2 Enhanced the clarity and resolution of the images within the revised Figure 1 to ensure that all annotations, including the scale bar and arrows indicating inflammatory cell infiltration, are clearly visible.

3 Removed any unnecessary descriptions and refined the figure caption to be more concise and relevant to the displayed data.

We believe that these modifications have resolved the issues previously present in Figure 2 and have improved the quality and integrity of our presentation.

④Table2: Lym dose not belong to tissue.

Reply to reviewer: Thank you for your comment on Table 2. We have decided to remove Table 2 from our manuscript for the following reasons:

1 Relevance and Focus: After re-evaluating the overall structure and focus of our study, we concluded that the data presented in Table 2, while interesting, are not central to the main findings of our research. We aim to ensure that the manuscript emphasizes the most critical aspects of our investigation.

2 Simplification of Results: We have streamlined our results to enhance readability and better convey the primary outcomes of our experiments. The removal of Table 2 contributes to a more concise presentation, avoiding information that might detract from the main messages of our study.

3 Avoidance of Redundancy: We identified some overlap between the data in Table 2 and other sections of the paper. To avoid redundancy and maintain the highest scientific standards, we chose to consolidate the information and present only the most pertinent findings.

Enhanced Data Presentation: We believe that the most impactful way to present our findings is through a revised Figure 1-9, which integrates the key data points in a clear and visually accessible format.

We have revised the manuscript accordingly and are confident that these changes have improved the clarity and impact of our presentation.

⑤Figure4B：As shown in Figure4B, the percentage of CD3+CD4+ T lymphocytes increased significantly in the DEX groups. This result is relatively rare and may require further analysis or discussion.

Reply to reviewer: Thank you for your insightful comment on Figure 4B, where you noted the significant increase in the percentage of CD3+CD4+ T lymphocytes in the DEX groups as being relatively rare. We appreciate the opportunity to further elucidate this finding.

1 Our results indicate that DEX treatment led to a significant increase in CD3+CD4+ helper T cells, which, while less commonly reported, is not without precedent and may be attributed to the intricate immunomodulatory effects of DEX. This increase could be related to DEX's role in promoting the Th2 type immune response, as suggested by previous studies [27, 28]. The promotion of CD4+ T cells by DEX might be due to its ability to influence the cytokine environment, leading to a preferential expansion of this subset.

27.	Q F, M X, Y Y Y, Y H, X M, Y X S, et al. High-dose dexamethasone or all-trans-retinoic acid restores the balance of macrophages towards M2 in immune thrombocytopenia. 2017;15(9). doi: 10.1111/jth.13767. PubMed PMID: 28682499.

28.	Yi J, Xu F, Xuehua Z, Chunxue B, Lei Z, Meiling J, et al. IL-13(+) Type 2 Innate Lymphoid Cells Correlate with Asthma Control Status and Treatment Response. 2016;55(5). doi: 10.1165/rcmb.2016-0099OC. PubMed PMID: 27314535.

2 We have expanded our discussion to consider the potential mechanisms behind this observation. DEX is known to have biphasic effects on immune cells, and its impact can vary depending on the timing and dosage of administration [21]. The increase in CD3+CD4+ T cells may reflect a shift towards a more regulatory or anti-inflammatory phenotype, which could be beneficial in certain contexts of immune modulation.

21.	Wang W, Jiang T, Zhu Z, Cui J, Zhu L, Ma Z. Dexamethasone suppresses allergic rhinitis and amplifies CD4(+) Foxp3(+) regulatory T cells in vitro. International forum of allergy & rhinology. 2015;5(10):900-6. Epub 2015/06/19. doi: 10.1002/alr.21579. PubMed PMID: 26086746.

3 Additionally, we have reviewed the literature and found that the immunosuppressive actions of DEX can be complex and context-dependent. For instance, DEX has been shown to induce apoptosis in certain T cell populations while sparing others, potentially leading to the observed increase in CD3+CD4+ T cells [21]. This finding underscores the need for a nuanced understanding of DEX's effects on immune cell dynamics.

We trust that our additional analysis and discussion provide a clearer context for the observed increase in CD3+CD4+ T lymphocytes with DEX treatment and address the concerns raised.

⑥3.8 Structure analysis of intestinal bacterial flora

The analysis of intestinal bacterial flora is too simple and needs further investigation, such as supplementation α Diversity analysis, species between different groups, and association analysis with immune cells, etc

Suggest changing the group of Figure6 and Figure7 to “Control、Cy and DEX”

Reply to reviewer: Thank you for your insightful suggestions regarding the structural analysis of the intestinal bacterial flora in our study. In response to your comments, we have made the following revisions:

1 α-Diversity Analysis: We have included additional α-diversity analysis with the Shannon and Chao indices for a more comprehensive assessment of the richness and evenness of the microbial communities across different treatment groups.

2 Comparison of Species Between Groups: We have conducted a comparative analysis of the microbial species present in the different groups, providing insights into the taxonomic changes induced by the treatments.

3 Association Analysis with Immune Cells: We acknowledge that the current study does not encompass an association analysis between the gut microbiota and the host's immune system. We recognize the significance of this analysis for a deeper understanding of the immunomodulatory effects of CY and DEX and will incorporate it into our future research.

“While this study provides preliminary insights into the effects of CY and DEX on the diversity and structure of the gut microbiota in mice, there are limitations. The research primarily focused on the direct impact of the drugs on the composition of the gut microbiota, and the specific mechanisms by which the drugs indirectly affect host immunity through the flora are not yet clear. Moreover, the study was conducted solely in a mouse model, and its clinical relevance needs further validation in human studies. Future research will require an association analysis between the gut microbiota and the host's immune system, as well as studies on the personalized use of CY and DEX in different populations. This will contribute to a more comprehensive understanding of the immunomodulatory mechanisms of CY and DEX and provide a scientific basis for the development of new therapeutic strategies.”

4 Figure Legend Modification: As per your suggestion, we have updated the group labels in the previously mentioned Figures 6 and 7 to "Control, CY, and DEX," and we have noted that these changes are now reflected in the new Figures 8 and 9 to enhance clarity and consistency.

Fig 8 Analysis results of intestinal flora diversity in mice. (A) Venn diagram was used to show the common and unique operational taxa (OTU) of intestinal samples from different treatment groups. (B) and (C) showed the Shannon and Chao indices of intestinal microflora in control group, CY group and DEX group, respectively, to assess α diversity.

Fig 9 Analysis of intestinal flora structure in mice. (A) Relative abundance of phylum-grade dominant bacteria in fecal samples of mice in control group, CY treatment group and DEX treatment group. (B) Analysis of intestinal flora composition at genus level in fecal samples of mice in control group, CY treatment group and DEX treatment group.

We have clearly stated these revisions in the manuscript and discussed their significance in the discussion section. We look forward to your further feedback.

5、There are many issues with the manuscript writing, and it is necessary to make professional revisions

①A space is required between numbers and units，for example“24h,48h,72h”

Answer: Thank you so much for your valuable suggestion. I have added space for each.

②Many symbol descriptions are inconsistent，for example“p<0.01”、“(P < 0.001; Table 1).”、“*means: p<0.05;**means”、“**p < 0.01.”“P<0.05”

Reply to reviewer: We greatly appreciate your meticulous review and valuable suggestions regarding the manuscript writing. We have carefully inspected and corrected all inconsistencies in the use of symbols throughout the text to ensure uniformity and professionalism. Here is the status of our revisions in response to the issues you raised:

Space Between Numbers and Units: We have added spaces in all time indications, such as "24 h, 48 h, 72 h," to adhere to the standard formatting.

Consistency of Symbol Descriptions: We have standardized the representation of all statistical significance symbols to ensure consistency as follows:

All p-value notations are now uniformly presented as "p < 0.05," "p < 0.01," and "p < 0.001."

We have conducted a comprehensive proofreading of the manuscript to ensure the professionalism and accuracy of all content. We believe these revisions will enhance the quality of our submission and meet the journal's standards. We look forward to your further feedback.

③The serial number of the title is duplicated. 2.7 Intestinal SIgA. and 2.7 Determination of gut microbiota abundance and diversity.

Reply to reviewer: Thank you for pointing out the duplication of section numbers in our manuscript. We sincerely apologize for this oversight and have made the following corrections:

We have reviewed the entire document to ensure that all section numbers are unique and accurately reflect the order of the sections.

The duplicated section number "2.7" has been corrected. The sections previously labeled as "2.7" for "Intestinal SIgA" and "Determination of gut microbiota abundance and diversity" have been renumbered to avoid any confusion.

We have conducted a thorough proofreading of the manuscript to prevent such errors from occurring again and to ensure the accuracy and professionalism of the submission. We look forward to your further feedback.

Reviewer #2: Li et al. describe their work on profiling the immune responses and gut microbiome changes to common immunosuppression agents, Cy and DEX, in a DNFB-mediated DTH female mouse model. In their study, they characterize the immune suppression elicited by Cy and DEX through flow cytometry analysis of peyer's patches, Lym, and MLN. They additionally showed decreased sIgA levels in both treatment groups and differences in their gut microbiome compositions across groups. Overall, the authors generated potentially interesting immune response and microbiome datasets, but I am uncertain of the study's significance and encourage the authors to better convey the motivation behind their study in the introduction of the paper for the broad readership of PLOS ONE. I have some additional recommendations below.

We would like to express our sincere gratitude for your recognition of our research work and the valuable suggestions you have provided during the review process. Your positive evaluation serves as an encouragement and offers guidance on how to better articulate the significance and motivation behind our study for the broad readership of PLOS ONE.

- I understand that Cy and DEX are two commonly used drugs but it is not totally clear from the intro why the authors were interested in comparing Cy vs. DEX. Are there indications in which either Cy and DEX could be used for treatment and is that directly related to the DTH model? The authors should clarify this point in the introduction or not make comparisons between the two treatment arms and only with the control arm.

Reply to reviewer: Thank you for your interest in our study and the specific questions regarding the rationale and relevance of comparing Cy and DEX. In response to your feedback, we have made the following specific revisions to the introduction:

1 In the first paragraph, we have added a description of the importance of the DTH model in understanding the role of memory T cells in cellular immunity.

2 In the second paragraph, we clarified the role of CY and DEX as immunosuppressive agents in treating autoimmune and inflammatory diseases and highlighted their specific impacts in the DTH model that have not been fully explored.

3 In the third paragraph, we emphasized the role of the gastrointestinal tract microbiota in immune homeostasis and the potential impact of dysbiosis caused by immunosuppressive drugs on systemic immune responses.

4 In the fourth paragraph, building on previous research, we articulated the aim of our study to dissect the comparative impact of Cy and DEX on intestinal immunity and microbiota in a DTH mouse model.

5 Finally, in the fifth paragraph, we stated the purpose of this research to determine the capacity of these agents to induce immunosuppression in both systemic and intestinal compartments and how they modulate intestinal mucosal immunity and microbiota, which could have significant implications for therapeutic strategies and the development of targeted immunotherapies.

We believe these revisions directly address your questions and clearly convey the motivation and scientific significance of our study. We look forward to your further feedback.

- The introduction's opening sentence also is misleading since the authors do not talk about oral vaccines. I recommend that the authors heavily rewrite their introduction to better set up and motivate their study.

Reply to reviewer: Thank you for pointing out the potentially misleading opening sentence in the introduction. Following your recommendation, we have thoroughly rewritten the introduction to better set up and motivate our study.

Specifically, we have removed the opening sentence that may not be directly related to our research and have included the following to strengthen the introduction:

1 We introduce the importance of delayed-type hypersensitivity (DTH) in understanding cellular immunity as the starting point of our study.

2 We emphasize the role of immunosuppressive agents in treating autoimmune and inflammatory diseases and their potential impact in the DTH model.

3 We provide a detailed description of the role of the gastrointestinal microbiota in immune homeostasis and the impact of dysbiosis caused by immunosuppressive agents on systemic immune responses.

4 We clarify the objectives of our study, which is to dissect the comparative effects of CY and DEX on intestinal immunity and microbiota in a DTH mouse model.

We believe these revisions will help readers better understand the relevance and significance of our research. We look forward to your feedback and appreciate your continued support of our work.

- Relatedly, all statistics appeared to be performed between control and treatment conditions and none were performed comparing Cy vs. DEX. If the authors want to make statements such as one treatment being better than the other, then the authors should perform the appropriate statistical tests between treatment arms.

Reply to reviewer: Thank you for highlighting the need for statistical comparisons between the treatment conditions in our study. We have taken your advice to heart and have re-analyzed our data, with a particular focus on comparing the effects of CY and DEX.

During this re-analysis, we performed the appropriate statistical tests to compare the two treatment arms. Based on these new results, we have updated the description and discussion sections to ensure that our conclusions are well-supported and justified.

We have made sure that all comparisons are backed by statistical evidence and have provided a detailed explanation of the different effects of CY and DEX in the discussion. Furthermore, we have explored potential reasons for any observed differences and discussed the implications of these differences for clinical practice and future research in our conclusions.

We believe these revisions have strengthened the rigor of our study and provided deeper insights. We look forward to your further feedback and appreciate your valuable comments on our research.

- The authors should strengthen their discussion around the motivation for looking at microbiome changes.

Reply to reviewer: Thank you for your interest in our study and your suggestion to strengthen the discussion around the motivation for examining changes in the microbiome. In response to your feedback, we have enhanced our discussion regarding the analysis of gut microbiota changes and made the following revisions to the discussion section:

1 We have elaborated on the impact of CY and DEX on the diversity of the gut microbiota in mice, as revealed by 16S rRNA sequencing technology, and how they significantly modulate the α diversity of the gut microbiota. (Fig 8)

2 We analyzed the effects of CY and DEX on the structure of the gut microbiota, particularly how they alter the relative abundance of specific flora and how these changes are reflected in the composition of the gut microbial community. (Fig 9)

3 We emphasized the significant regulatory effects of CY and DEX on the composition of the gut microbiota and the profound impact these modulations may have on the host's metabolism and immune status. (The 10th paragraph of the discussion)

4 Given the limited literature on the interplay between the gut microbiota, sIgA, and B cells, we have made every effort to cite relevant literature in support of our findings and to point out directions for future research. (The 10th paragraph of the discussion)

We acknowledge that, despite the limited literature, our findings provide new perspectives for understanding the interactions between drugs and the gut microbiota and lay the groundwork for further research.

We believe that these revisions not only address your suggestions but also deepen and broaden the scope of our study. We look forward to your further feedback.

- The authors say that IgA increased in the abstract - I believe this was a typo and that the data show IgA decreased in the treatment arms compared to the control arm.

Reply to reviewer: Thank you for identifying the error regarding the intestinal sIgA content in our abstract. We have made the necessary corrections to the abstract and the results section to ensure consistency with our experimental data.

In the Results section, we have clarified that treatment with CY and DEX actually led to a significant decrease in intestinal sIgA content compared to the control group. This finding has been corrected and emphasized in the text to ensure the accuracy of the statement.

“Results

CY and DEX significantly reduced the immune function in DNFB-induced sensitized mice, as indicated by decreased thymus and spleen indices, MLN enlargement, intestinal sIgA content, and ear swelling degree. ”

We apologize for the typo in the abstract and have updated the content to align with the description in the results section. We believe this revision addresses the issue you raised.

We look forward to your further feedback and appreciate your attention to our research.

- It is not obvious how many mice were used within each treatment arm. Also, the authors did not mention whether the mice across treatment groups were housed together or separately as this is an important point in any microbiome study. A schematic of the treatment regimen may also be helpful to include in the supplement or as part of figure 1.

Reply to reviewer: Thank you for your insightful comments and suggestions regarding the housing conditions in our microbiome study. Thank you for your attention to the details regarding the number of mice used in each treatment arm and their housing conditions in our microbiome study. In our methods section, we have clearly stated that there were 6 mice per group, with a total of 18 mice evenly distributed across the three treatment groups. Moreover, we have provided a detailed description of the group housing of mice, which allows for natural social interactions and is critical for reflecting more natural conditions for microbiome development. (The first and second paragraphs of the method)

We have revised the methods section to clearly articulate our housing strategy and to provide a compelling rationale for our choice of group housing for the following reasons:

1 Natural Social Interactions: Group housing allows mice to engage in natural social interactions, which are crucial for their behavioral and physiological health. This is particularly important as social behavior can influence microbiome dynamics.

2 Ethological Relevance: Housing mice in groups better reflects natural conditions and is more representative of the ecological context in which the gut microbiome has co-evolved with the host, potentially leading to more robust and generalizable findings.

3 Controlled Environment: Despite group housing, we ensured that all groups were maintained under identical and controlled environmental conditions to minimize variability not related to the treatments.

4 Minimized Cross-Contamination: Each treatment group was housed in separate cages to prevent cross-contamination of microbiota while still allowing for the necessary social interactions within groups.

5 Ethical Considerations: Our approach aligns with ethical guidelines that prioritize the welfare of the animals, ensuring that their housing conditions support both scientific rigor and animal well-being.

We trust that these revisions and the rationale provided will address your concerns and demonstrate our commitment to both scientific excellence and animal welfare in our research.

Regarding your suggestion for a schematic of the treatment regimen, while we understand that this could be helpful, we believe it is not necessary for our study. Our methods section already provides an exhaustive description of the experimental design, including mouse grouping, treatment, sample collection, and analysis methods. Additionally, our data and results are presented in a clear and logical manner in the text, allowing readers to easily follow the experimental process and outcomes.

We believe that through clear textual descriptions and detailed methodological explanations, we have provided sufficient information to understand our experimental design and results without the need for additional schematics. Our aim is to maintain the simplicity of the report, avoiding unnecessary complexity while ensuring that our study is transparent and replicable.

We look forward to your further feedback and appreciate your ongoing support of our research.

- A circos plot should not be used in Figure 6. Instead the authors should perform statistical tests on the differences in taxa group relative abundances between groups. The authors should show stacked abundance plots for each mouse/stool sample that was sequenced. The authors may also consider a PCoA plot.

Reply to reviewer: Thank you for your valuable feedback regarding the use of a circos plot in Fig 6 of our study. In response to your suggestion, we have re-analyzed the data and presented our results using more appropriate statistical methods and visualization techniques.

We performed statistical tests to assess the differences in taxa group relative abundances between groups and presented the microbiota composition of each mouse/stool sample using stacked abundance plots. Additionally, we included a Principal Coordinate Analysis (PCoA) plot to illustrate the differences in microbial community structure among the treatment groups.

Specifically, we used a Venn diagram to show the distribution of OTUs among different samples, revealing the common and unique microbial groups in the intestinal flora of mice in each group (Fig 8A). α-diversity analysis indicated that CY treatment significantly increased α diversity in the mice intestinal flora, while DEX treatment significantly decreased it (Fig 8B and 8C). These results suggest that CY and DEX have significant regulatory effects on the diversity of the intestinal flora in mice, with opposite trends.

Fig 8 Analysis results of intestinal flora diversity in mice. (A) Venn diagram was used to show the common and unique operational taxa (OTU) of intestinal samples from different treatment groups. (B) and (C) showed the Shannon and Chao indices of intestinal microflora in control group, CY group and DEX group, respectively, to assess α diversity.

In terms of gut microbiota structure analysis, we presented the distribution of dominant bacterial communities at the phylum level in the fecal samples of the three groups of mice (Fig 9A), as well as the composition of the gut microbiota at the genus level (Fig 9B). These results indicate that CY and DEX significantly affect the structure and function of the gut microbiome by altering the relative abundance of specific flora.

Fig 9 Analysis of intestinal flora structure in mice. (A) Relative abundance of phylum-grade dominant bacteria in fecal samples of mice in control group, CY treatment group and DEX treatment group. (B) Analysis of intestinal flora composition at genus level in fecal samples of mice in control group, CY treatment group and DEX treatment group.

We believe that these revisions meet your requirements and present our results in a more scientific and accurate manner. We look forward to your further feedback.

- The authors should more clearly discuss reasons for the differences in immune cell subpopulations between the treatment groups and differences between up and down regulation of these population across compartments.

Reply to reviewer: Thank you for your insightful comments on the analysis of immune cell subpopulation differences in our study. We have carefully considered your suggestions and have made the following detailed revisions:

1 Quantitative Data Measurement: We conducted a quantitative analysis of various T cell subsets in the mesenteric lymph nodes (MLNs), including the percentages of total CD3+ T cells, activated T cells, helper T cells, cytotoxic T cells, and T cells with different T cell receptors (TCRs).

2 Regulatory Effects of CY and DEX: Our results show that CY treatment significantly increased the percentage of total CD3+ T cells, with no significant effect on activated T cells, while DEX treatment significantly increased the percentage of helper T cells, and CY treatment reduced this proportion.

“**Effects of CY and DEX on T cell subsets of MLNs**

In the in-depth analysis, we quantitatively measured the percentage of total CD3+T cells, CD3+CD69+ activated T cells, CD3+CD4+ helper T cells, CD3+CD8+ cytotoxic T cells, CD3+TCRβ+ T cells, and CD3+TCRγ/δ+ T cells. To investigate the regulatory effects of CY and DEX on the distribution of T cell subsets in mesenteric lymph nodes of sensitized mice. The results showed that compared with the control group, the percentage of CD3+ total T cells in CY group was significantly increased, and the percentage of CD3+CD69+ activated T cells had no significant effect. Compared with the DEX treatment group, the percentage of CD3+ total T cells and CD3+CD69+ activated T cells in MLNs after CY treatment was significantly increased (Fig 3). Further analysis showed that CY treatment significantly reduced the percentage of CD3+CD4+ helper T cells in MLNs, while DEX treatment significantly increased the percentage (Fig 4). For CD3+CD8+ cytotoxic T cells, neither CY nor DEX treatments had a significant effect compared to controls (Fig 5). In addition, compared with the control group, there was no significant change in the percentage of CD3+TCRβ+ T cells in the CY treatment group (Fig 6), but the percentage of CD3+TCRγ/δ+ T cells was significantly reduced (Fig 7). Compared with DEX, the percentage of CD3+TCRβ+ T cells was significantly increased and the percentage of CD3+TCRγ/δ+ T cells was significantly decreased after CY treatment. These results suggest that CY and DEX have a significant regulatory effect on the distribution of T cell subpopulations in the sensitized mice mesenteric lymph nodes, in which CY promotes the increase of the proportion of total T cells while inhibiting the proportion of activated T cells and γδ T cells, while DEX is more effective in increasing the proportion of helper T cells. These differential regulatory effects may indicate the different emphasis of CY and DEX on immunosuppressive mechanism, and provide an important reference for clinical application.”

3 Distribution Differences of Immune Cell Subpopulations: We have thoroughly discussed how CY and DEX regulate the immune response by affecting the proportions of different T cell subpopulations, which may reflect different emphases in their immunosuppressive mechanisms.

In-depth Mechanism Discussion: We have added a discussion on the potential mechanisms of action of CY and DEX, including how they regulate T cell subsets by affecting T cell receptor signaling pathways and promoting specific types of immune responses. (Paragraphs 4-9 of the discussion)

4 Clinical Application Implications: We have emphasized the importance of these findings for clinical applications, especially regarding the impact of drug dosage and administration timing on the modulation of immune responses. (Paragraphs 4-9 of the discussion)

5 Figures and Statistical Analysis: To more clearly present the results, we have added figures to visually represent the differences between treatment groups and have conducted appropriate statistical tests to support our conclusions.

We believe that through these detailed revisions and additions, we have not only fully addressed your comments but also enhanced the depth and rigor of our study. We look forward to your further feedback.

- I am not sure that the vague statement of Cy is better than DEX is well supported in the conclusion.

Reply to reviewer: Dear Reviewer,

Thank you for your scrutiny of the comparison between CY and DEX in the conclusion of our study. We understand your uncertainty regarding the vague statement that "CY is better than DEX" and agree that scientific conclusions must be well-supported by data. Therefore, we have made significant revisions to our conclusion to ensure accuracy and justification.

1 Data Support: We have supported our conclusions with solid experimental data, including analyses of gut microbiota diversity and structure, changes in immune cell subpopulations, and the regulatory effects of the drugs on T cell subset distribution.

2 Statistical Analysis: We have conducted rigorous statistical tests to compare differences between the CY and DEX treatment groups and ensured that all conclusions are based on the results of these analyses.

3 Depth of Discussion: In the discussion section, we have delved into the different impacts of CY and DEX on the immune response, including their regulatory roles in gut microbiota and specific changes in immune cell subpopulations.

4 Precision of Conclusions: We have revisited the conclusion section to avoid vague statements like "CY is superior to DEX," instead specifying in what aspects CY and DEX show different effects and the potential significance of these differences.

5 Clinical Implications: We have emphasized the clinical relevance of the study findings, especially how CY and DEX can be applied in different therapeutic scenarios based on their unique mechanisms of action.

6 Future Research Directions: We have suggested directions for future research, including further exploration of the molecular mechanisms by which CY and DEX affect the immune system and their potential applications in various disease states.

We believe that with these revisions, our conclusions are more precise and persuasive, and are fully supported by experimental data and scientific analysis. We look forward to your further feedback.
