# Supplementary material for: Comparative analysis of the effects of cyclophosphamide and dexamethasone on intestinal immunity and microbiota in delayed hypersensitivity mice
Source: PLoS One. 2024 Oct 17;19(10):e0312147. doi: 10.1371/journal.pone.0312147 (PMC11486373; doi:10.1371/journal.pone.0312147)
Supplement: Attachment — Submitted filename: plos one.docx [file pone.0312147.s009.docx]

The paper by Xiangling Li and authors given the increased use of immunosuppressants for the most diverse autoimmune diseases. The paper is interesting and reveals important information about the link between gut flora and systemic hypersensitivity reaction.This is an interesting paper due to the results obtained, but some considerations about the methodology and writing should be implemented.

1. In the introduction, it is recommended to search for other references on the immunosuppressive dosage of cyclophosphamide (CY) and Dexamethasone (DEX). The dosage of CY used in different literature reports is not the same, why the study used 100mg/kg CY and 40mg/kg DEX ? It is recommended to add a detailed explanation CY and evidence in the introduction section.
2. Another important consideration is the role of CY on the B cell response, which is not reported in the paper. However, the paper had about the detection of SIgA, in general, we believe that the secretion of antibodies is related to B lymphocyte. Assessment of B lymphocyte populations is essential in this situation.
3. The changes of T cell subsets and intestinal flora were detected in the paper. However, the relationship between intestinal flora and T cell subpopulation changes was not discussed in depth in the discussion section. Although the two changes may be the result of the separate action of CY and DEX, whether they affect each other was not mentioned.
4. The figures of intestinal bacterial flora is unclear, especially in Figure 9, the name of intestinal bacterial flora is not clear, it is suggested to change the picture or increase the color contrast.
5. The manuscript needs language and Grammar editing.The abbreviations before and after the text should be consistent, and the abbreviations appeared in the previous text should be directly followed by abbreviations, such as line 232, line 246‘mesenteric lymph nodes’, which can be directly represented by‘MLN’. In addition, in all image annotations, the comparison after the comma is capitalized, such as lines 255,259,268,273‘Compared to the Control group, **P<0.01, Compared to the DEX group’. There is no superscript of + in the whole paper.
