# Supplementary material for: Comparative analysis of the effects of cyclophosphamide and dexamethasone on intestinal immunity and microbiota in delayed hypersensitivity mice
Source: PLoS One. 2024 Oct 17;19(10):e0312147. doi: 10.1371/journal.pone.0312147 (PMC11486373; doi:10.1371/journal.pone.0312147)
Supplement: Attachment — Submitted filename: Response to Reviewers.doc [file pone.0312147.s010.doc]

Reply to Editor:

I will consider the minor revisions satisfied with the following points addressed:

(1)Clarification of experimental timing for euthanasia and when (post- or ante-mortem) tissue was collected.

Reply to Editor: Thank you for your feedback. We have revised our Methods section to include comprehensive details regarding the animal experiments. Specifically, we have now provided explicit information on :

“The following day, Day 7, fecal samples were gathered through direct anal extraction to analysis the gut microbiota , after that，ear tissue samples were collected post-euthanasia using an 8.5 mm punch to measure the degree of ear swelling. The spleen and thymus were removed, weighed, and their organ indices calculated. The mesenteric lymph nodes (MLNs) were extracted to evaluate proliferation and cell subpopulations of lymphocyte. The small intestine was harvested to analysis the intestinal Peyer's Patches(PPs) and determination of Intestinal Secretory IgA.The ear tissue samples were fixed, embedded, sectioned, and stained with hematoxylin and eosin for histological examination.”

In this section, we added the time of tissue collection from various parts of the mouse to clarify the experimental process.

We appreciate your guidance and have made the necessary revisions to ensure the accuracy and clarity of our methods.

(2)Clarification around the proposed immunomoduatory effects of Cy and Dex on T cell populations and tempering of the claims of direct effects without mechanistic study.

Reply to Editor: Thank you for your feedback. In the discussion section, we have clearly explained the effects of CY and DEX on T cells and their subpopulations. In the article, we have also made it clear that:

CY treatment has been shown to significantly increase the percentage of CD3+ total T cells in the MLNs of sensitized mice. Despite the increase in total T cells, CY treatment decreases the percentage of CD3+CD69+ activated T cells, suggesting an inhibition of T cell activation. CY also reduces the percentage of CD3+CD4+ helper T cells and CD3+TCRγ/δ+ T cells, indicating a modulation of specific T cell subsets.

DEX treatment leads to a significant increase in the percentage of CD3+CD4+ helper T cells, which could be linked to its promotion of the Th2 type immune response.Unlike CY, DEX does not decrease the percentage of CD3+CD69+ activated T cells. DEX decreases the percentage of CD3+TCRβ+ T lymphocytes compared to the control group.

The immune system is a complex network, and the effects of immunosuppressive drugs like CY and DEX are likely to be influenced by multiple factors, including dosage, administration timing, and the balance of lymphocyte sub-populations.

Further studies are needed to investigate the mechanisms underlying the immunomodulatory effects of Cy and Dex, including their impact on T cell migration, adhesion molecule expression, and T cell receptor signaling.

As the mean while,we have rewrote the conclusion section:

In summary, CY and DEX have significant regulatory effects on the immune organ index, distribution of T cell subsets, and diversity and structure of gut microbiota. These results not only deepen our understanding of the mechanisms of action of these two drugs, but also provide scientific basis for their application in animal models and subsequent research on the mechanism of immune regulation and individualized application in clinical treatment.

We appreciate the opportunity to enhance our manuscript with this additional analysis and believe these revisions will significantly improve the clarity and impact of our findings.

(3)Explanation of the dosing chosen with literature support.

Reply to Editor: Thank you for your attention about the dose of CY and DEX. In the introduction, we made modifications to the drug dosage and added relevant references and our previous experimental data to support the dosing chosen. In response to your query, we have made the following revisions to the introduction section of our manuscript:

“Both CY and DEX can successfully induce immunosuppression in various models [12-13], and a dose of 80 mg/kg CY used for 5 consecutive days can successfully induce immunodeficiency in mice [14].Based on our previous time（24h,48h,72h）and dosage (DEX: 200mg/kg，120mg/kg，40mg/kg，24mg/kg or Cy: 300mg/kg，200mg/kg，100mg/kg，50mg/kg) studies of Cy and DEX[15], the current study aims to dissect the comparative impact of a single high dose of CY (100 mg/kg) and DEX (40 mg/kg) on intestinal immunity and microbiota in a DTH mouse model.”

We appreciate your guidance and have added literature to to ensure the accuracy and clarity of our methods.

(4) Addition of discussion around the effects of Cy and Dex on B cells. Text additions will be sufficient.

Reply to Editor: Thank you for your request for the effects of Cy and Dex on B cells. We have added the effects of CY and DEX on B cells in discussion as follow:

“Plasma cells in the lamina propria contribute the most to secretory IgA (sIgA) secretion, CY exhibited a selective and differential suppressive effect on various stages of the B cell cycle, including activation, proliferation, and differentiation, leading to reduced immunoglobulin secretion by B cells following in vitro stimulation[23]. Similarly, DEX also inhibited antibody production by B cells[24].”

We appreciate the opportunity to further elucidate our discussion and look forward to any additional guidance and feedback you may provide.

(5) Clarification in the figure of flora identifiers, or inclusion of supplemental material with a table listing the flora.

Reply to Editor: Thank you for your guidance on the figures. We have added the names of the bacterial groups to the legend of Fig. 9. Specifically, we have now provided explicit information on :

**“Fig 9 Analysis of intestinal flora structure in mice.** (A) Relative abundance of phylum-grade dominant bacteria in fecal samples of mice in control group, CY treatment group and DEX treatment group.From bottom to top in order are: *P_Bacteroidota, p_Firmicutes, p_Campylobacterota, p_Deferribacterota, P_Patescibacteria, p_Proteobacteria, p_Desulfobacterota* and *other.* (B) Analysis of intestinal flora composition at genus level in fecal samples of mice in control group, CY treatment group and DEX treatment group. The top ten, are as follows:*g_Bacteroides, g_norank_Muribaculaceae, g_Lachnospiraceae_NK4A136_group, g_Helicobacter, g_Alloprevotella, g_Alistipes, g_unclassified_Lachnospiraceae, g_norank_Oscillospiraceae, g_Parabacteroides* and *g_norank_Clostridia_UCG-014”*

We are extremely grateful for your guidance and believe that these modifications will significantly enhance the clarity of the changes in bacterial groups depicted in our figures, making the altered bacterial groups more explicit.

(6) Please do another grammatical check of the paper.

Reply to Editor: Thank you for your request for grammatical check of the paper. We confirm that, after numerous revisions, the grammar in our manuscript is now nearly perfect. In this revision, we have specifically addressed the grammatical issues raised by the reviewer# 1 and have marked these changes within the text.At the same time, we revised and refined the format of the references in the revised manuscript.

Please do not hesitate to contact us if further revisions are needed.

Reply to Reviewers:

Reviewer #1: The paper is interesting and reveals important information about the link between gut flora and systemic hypersensitivity reaction. This is an interesting paper due to the results obtained, but some considerations about the methodology and writing should be implemented.

1. In the introduction, it is recommended to search for other references on the immunosuppressive dosage of cyclophosphamide (CY) and Dexamethasone (DEX). The dosage of CY used in different literature reports is not the same, why the study used 100mg/kg CY and 40mg/kg DEX ? It is recommended to add a detailed explanation CY and evidence in the introduction section.

Reply to reviewer: First and foremost, we would like to express our sincere gratitude for your insightful comments and suggestions.

This research is carried out on the basis of our laboratory, we saw the report that different administration ways, different drug doses, different animal sampling time, the results of immunosuppressive is different, so we tested the DEX 64 mg/kg, 40 mg/kg, 13.2 mg/kg, 8 mg/kg with 6 animals in each group administered for 3 days . On the 4th day, the systemic immune status of mice was detected. After that a single dose of DEX 200mg/kg ,120mg/kg 40mg/kg;24mg/kg was administered to detect the systemic immune status of mice. Also a single dose of 300mg/kg, 200mg/kg, 100mg/kg, and 50mg/kg of CY were taken to the mice. All these experiments were tested three timed for 24h,48h, and 72h to observe the suppression of the immune system. Based on this, the dose and administration time of DEX and CY in this study were determined. At the same time, it taken 48 hours after intraperitoneal injection of DEX or CY may also avoid the rebound effect with an increase in their circulation.

we have now provided explicit information on:

“Both CY and DEX can successfully induce immunosuppression in various models [12-13], and a dose of 50 mg/kg CY used for 2 consecutive days can successfully induce immunodeficiency in mice [14]. Based on our previous time(24h,48h,72h)and dosage (DEX: 200mg/kg，120mg/kg，40mg/kg，24mg/kg or Cy: 300mg/kg，200mg/kg，100mg/kg，50mg/kg) studies of Cy and DEX[15], ”

We appreciate your guidance and have made the necessary revisions to ensure the accuracy and clarity of our methods.

2. Another important consideration is the role of CY on the B cell response, which is not reported in the paper. However, the paper had about the detection of SIgA, in general, we believe that the secretion of antibodies is related to B lymphocyte. Assessment of B lymphocyte populations is essential in this situation.

Reply to Reviewer:Thank you so much for your valuable comments.

Because this experiment used a DTH animal model, which is mainly used to study T cell immune responses, no data on B cells were provided in this study. In addition, SIgA was detected in this study because of the relationship between SIgA and intestinal flora. Based on your suggestion, we have added content discussing the effects of CY and DEX on B cells in the discussion section:“Plasma cells in the lamina propria contribute the most to secretory IgA (sIgA) secretion, CY exhibited a selective and differential suppressive effect on various stages of the B cell cycle, including activation, proliferation, and differentiation, leading to reduced immunoglobulin secretion by B cells following in vitro stimulation[23]. Similarly, DEX also inhibited antibody production by B cells[24].”

Meanwhile, in the following study, we will conduct further detection of B cells and B cell-related factors in various parts.

3. The changes of T cell subsets and intestinal flora were detected in the paper. However, the relationship between intestinal flora and T cell subpopulation changes was not discussed in depth in the discussion section. Although the two changes may be the result of the separate action of CY and DEX, whether they affect each other was not mentioned.

Reply to Reviewer:First and foremost, we would like to express our sincere gratitude for your insightful comments and suggestions. When addressing the relationship between intestinal flora and T cell subpopulation changes after CY and DEX administration, it's important to note that while our study detected alterations in both, a detailed exploration of their interplay wasn't our primary focus. However, based on literature and our results, we can discuss potential interactions.	The gut microbiota plays a crucial role in modulating the immune system, including T cell responses. Changes in microbiota composition, as observed in our study, could impact T cell subpopulations. For instance, certain bacterial species promote regulatory T cells (Tregs), while dysbiosis can lead to immune-mediated disorders.

In our study, CY and DEX induced distinct changes in gut microbiota diversity and structure, likely contributing to observed changes in T cell subsets. Although we didn't directly investigate the causal relationship, the alterations suggest potential interactions. Future studies could further explore this interplay using targeted approaches, such as fecal microbiota transplantation or germ-free mice, and investigate specific bacterial strains and metabolites involved.

In conclusion, while our study detected alterations in T cell subsets and intestinal flora, a detailed exploration of their interplay wasn't our focus. However, based on literature and our results, it's plausible that changes in microbiota composition could contribute to the observed alterations in T cell subpopulations. Future studies are needed to elucidate the causal relationship.

4. The figures of intestinal bacterial flora is unclear, especially in Figure 9, the name of intestinal bacterial flora is not clear, it is suggested to change the picture or increase the color contrast.

Reply to Reviewer：Thank you so much for your valuable comments and suggestions. We have added the name of the intestinal bacterial flora in the legend of the figure 9.Just as follow:

**Fig 9 Analysis of intestinal flora structure in mice.** (A) Relative abundance of phylum-grade dominant bacteria in fecal samples of mice in control group, CY treatment group and DEX treatment group.From bottom to top in order are: *P_Bacteroidota, p_Firmicutes, p_Campylobacterota, p_Deferribacterota, P_Patescibacteria, p_Proteobacteria, p_Desulfobacterota* and *other.* (B) Analysis of intestinal flora composition at genus level in fecal samples of mice in control group, CY treatment group and DEX treatment group. The top ten, are as follows:*g_Bacteroides, g_norank_Muribaculaceae, g_Lachnospiraceae_NK4A136_group, g_Helicobacter, g_Alloprevotella, g_Alistipes, g_unclassified_Lachnospiraceae, g_norank_Oscillospiraceae, g_Parabacteroides* and *g_norank_Clostridia_UCG-014*

We are grateful for your suggestion and believe that these modifications will significantly enhance the clarity of the changes in bacterial groups depicted in our figures, making the altered bacterial groups more explicit.

5. The manuscript needs language and Grammar editing.The abbreviations before and after the text should be consistent, and the abbreviations appeared in the previous text should be directly followed by abbreviations, such as line 232, line 246‘mesenteric lymph nodes’, which can be directly represented by‘MLN’. In addition, in all image annotations, the comparison after the comma is capitalized, such as lines 255,259,268,273‘Compared to the Control group, **P<0.01, Compared to the DEX group’. There is no superscript of + in the whole paper.

Reply to reviewer: We greatly appreciate your meticulous review and valuable suggestions regarding the manuscript writing. We have carefully inspected and corrected all inconsistencies in the use of symbols throughout the text to ensure uniformity and professionalism. Here is the status of our revisions in response to the issues you raised:

About abbreviations: We have changed ‘mesenteric lymph nodes’ to MLN all in the paper.

Consistency of Symbol Descriptions: We have standardized comma to period to ensure consistency as follows: “Compared to the Control group, **P<0.01. Compared to the DEX group”

We have conducted a comprehensive proofreading of the manuscript to ensure the professionalism and accuracy of all content. We believe these revisions will enhance the quality of our submission and meet the journal's standards. We look forward to your further feedback.

Reviewer #3: When describing your study design, there was no mention of when the mice were euthanised and which tissue samples were collected pre and post mortem. This needs to be properly addressed as it is confusing.

Reply to Reviewer: Thank you for your feedback.The mice were euthanized on Day 7, immediately after the collection of fecal samples through ante-mortem direct anal extraction for gut microbiota analysis. Tissue Samples Collected Post-mortem: Ear Tissue: After euthanasia, ear tissue samples were collected using an 8.5 mm punch to measure the degree of ear swelling. Spleen and Thymus: The spleen and thymus were removed, weighed, and their organ indices were calculated. Mesenteric Lymph Nodes (MLNs): The MLNs were extracted to evaluate proliferation and cell subpopulations of lymphocyte. Small Intestine: The small intestine was harvested for analysis of intestinal Peyer's Patches (PPs) and determination of Intestinal Secretory IgA.

We have revised our Methods section to include comprehensive details regarding the animal experiments. Specifically, we have now provided explicit information on :

“The following day, Day 7, fecal samples were gathered through ante-mortem direct anal extraction to analysis the gut microbiota , after that ear tissue samples were collected post-euthanasia using an 8.5 mm punch to measure the degree of ear swelling. The spleen and thymus were removed, weighed, and their organ indices calculated. The mesenteric lymph nodes (MLNs) were extracted to evaluate proliferation and cell subpopulations of lymphocyte. The small intestine was harvested to analysis the intestinal Peyer's Patches(PPs) and determination of Intestinal Secretory IgA.The ear tissue samples were fixed, embedded, sectioned, and stained with hematoxylin and eosin for histological examination.”

Could baseline fecal samples have been collected to establish the microflora composition prior to administration of the test compounds and if so, why was this analysis not performed?

Reply to reviewer: Thank you very much for your valuable comments.It is reasonable to consider collecting baseline fecal samples to establish the microflora composition prior to the administration of the test compounds, Cyclophosphamide (CY) and Dexamethasone (DEX). The rationale for collecting baseline samples stems from the need to understand the initial microbial community structure and diversity within each mouse. This information is essential for accurately interpreting any shifts in microbiota composition that occur during the course of the study.

However, there are several potential reasons why this analysis may not have been performed in the current study:

**Study Design Constraints:** The study have been designed primarily to focus on the immediate effects of CY and DEX on intestinal immunity and microbiota following their administration on DTH mice. In addition to our previous experiments that investigated the effects of varying doses and administration times of CY and DEX on mice (blank mice without any treatment), we did not conduct further tests on blank mice in the current experiment.

**Focus on Comparative Analysis:** The study aimed to compare the effects of CY and DEX on intestinal immunity and microbiota. Therefore, the primary interest may have been in the differential impact of these drugs rather than the absolute changes from baseline.

**Ethical Considerations:** While collecting fecal samples is generally non-invasive and low-risk, minimizing the number of procedures performed on the animals is important to adhere to ethical guidelines and ensure their well-being.

Despite these reasons, incorporating baseline fecal sample collection in future studies would significantly enhance the ability to interpret changes in the gut microbiota accurately. Such data would provide a more comprehensive understanding of the drugs' effects on the microbial community and contribute to a more nuanced interpretation of their immunomodulatory mechanisms.

I am not satisfied with your conclusion that CY and DEX exhibit immunomodulatory effects by regulating T cell subsets, diversity of gut microflora etc. as you have not provided evidence for a regulatory effect. You have describes some alterations to immune celular parameters, without demonstrating a causal relationship or mechanism. Your conclusions should reflect this.

Reply to reviewer: Dear Reviewer,

Thank you for your scrutiny of the relationship or mechanism of immune celular parameters caused by CY or DEX in the conclusion of our study. We understand your uncertainty regarding the vague statement that "immunomodulatory effects" and agree that scientific conclusions must be well-supported by data. Therefore, we have made significant revisions to our conclusion to ensure accuracy and justification.

We have revisited the conclusion section to avoid vague statements like "CY and DEX have significant regulatory effects on the immune organ index, distribution of T cell subsets, and diversity and structure of gut microbiota." instead of “CY and DEX exhibit significant immunomodulatory effects by regulating the immune organ index”.to describe the alterations of immune celular parameters.

We have suggested directions for future research, including further exploration of the molecular mechanisms by which CY and DEX affect the immune system and their potential applications in various disease states.

We believe that with these revisions, our conclusions are more precise and persuasive, and are fully supported by experimental data and scientific analysis. We look forward to your further feedback.
